# Synthesis of biological based hennotannic acid-based salts over porous bismuth coordination polymer with phosphorous acid tags†

**DOI:** 10.1039/d0ra06674e

**Published:** 2021-01-07

**Authors:** Saeed Babaee, Mahmoud Zarei, Mohammad Ali Zolfigol, Sadegh Khazalpour, Masoumeh Hasani, Uwe Rinner, Romana Schirhagl, Neda Norouzi, Sadegh Rostamnia

**Affiliations:** Department of Organic Chemistry, Faculty of Chemistry, Bu-Ali Sina University PO Box 6517838683 Hamedan Iran mahmoud8103@yahoo.com zolfi@basu.ac.ir mzolfigol@yahoo.com saeed.babai@yahoo.com +988138380709 +988138282807; Department of Analytical Chemistry, Faculty of Chemistry, Bu-Ali Sina University Hamedan Iran khazalpour@yahoo.com hasani@basu.ac.ir; Department of Life Sciences, IMC University of Applied Sciences Piaristengasse 1, 3500 Krems Austria uwe.rinner@fh-krems.ac.at; University Medical Center Groningen, Groningen University Antonius Deusinglaan 1, 9713 AV Groningen Netherlands romana.schirhagl@gmail.com n.norouzi2018@gmail.com; Organic and Nano Group (ONG), Department of Chemistry, Faculty of Science, University of Maragheh PO Box 55181-83111 Maragheh Iran rostamnia@maragheh.ac.ir

## Abstract

In this paper, a novel porous polymer capable of coordinating to bismuth (PCPs-Bi) was synthesized. The Bi-PCPs was then reacted with phosphorous acid to produce a novel polymer PCPs(Bi)N(CH_2_PO_3_H_2_)_2_ which is shown to act as an efficient and recyclable catalyst. The mentioned catalyst was applied for the efficient synthesis of new mono and bis naphthoquinone-based salts of piperidine and/or piperazine *via* the reaction of hennotannic acid with various aldehydes, piperidine and/or piperazine, respectively. The structure of the resulting mono and bis substituted piperazine or piperidine-based naphthoquinone salts was thoroughly characterized spectroscopically. The electrochemical behavior of the products was also investigated. The presented protocol has the advantages of excellent yields (82–95%), short reaction times (4–30 min) and simple work-up.

## Abstract 2

In this paper, a novel porous polymer capable of coordinating to bismuth (PCPs-Bi) was synthesized.

## Graphical abstract

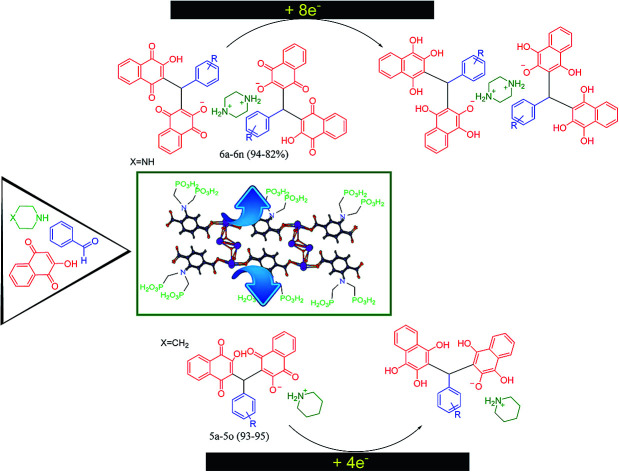

## 1. Introduction

Nowadays, porous material frameworks such as porous coordination polymers (PCPs) and metal–organic frameworks (MOFs), a combination of organic ligands and metal, are commonly used in catalysts, adsorption, gas storage, *etc*^1–10^. To the best of our knowledge, most of the bismuth salts are safe and this metal is known to be non-toxic and stable.^11–14^ Due to its high stability and non-toxicity it is used in cosmetics and pigments and has been used in equipment for drinking water. Additionally, it is added to multi-drug systems and as an alternative to lead in industry.^15,16^ In addition, bismuth-based porous material frameworks have been used for fluorescence-based detection.^17^ On the other hand, the emphasis of science and technology has shifted to design heterogeneous selective catalysts based on renewable, nanoporous, organic–inorganic hybrid and neutral materials. These often show lower toxicity and thus produce less hazardous waste. They are reusable, and have higher turnover numbers (TON) and turnover frequencies (TOF). In this regard, porous coordination polymers (PCPs) have been reported as porous catalysts which have high surface area and thermal stability.^18,19^

Phosphorus functional groups are essential linkers within the molecular structural of living cells and organisms.^20^ Acidic phosphorous derivatives are made from mineral phosphorus derivatives, which are naturally found in the body. One of their calcium salts contributes to the formation of healthy bones and teeth.^21,22^ These materials are a common additive for preserving flavors and bacteria in many foods processing routines.^23^ It also plays an important role in photosynthesis, cell division, respiration and energy transfer.^24^ Recently we have reported glycoluril, SBA-15, En/MIL-100(Cr) and melamine with phosphorous acid tags for the synthesis of organic compounds with biological properties.^25–28^

2-Hydroxynaphthalene-1,4-dione (hennotannic acid) or lawsone with a red-orange color is extracted from the leaves of the henna plant.^29^ Henna is used for dyeing of skin, hair, fabrics, fingernails, silk, wool and leather. Lawsone has been also used for the preparation of azo dyes,^30^ xanthenes and bis-coumarins,^31^ chromene derivatives,^32^ spirocompounds,^33^ and leuco-dyes.^34^

Naphthoquinone derivatives are main constituents of the lawson interims (Hana) found in abundance in leaves and bark.^35^ These structures have some medicinal and healing properties. They are antioxidant,^36^ antibacterial,^37^ antifungal,^38,39^ hepatoprotective,^39^ anti-inflammatory,^40^ antiviral, anticancer,^41^ protein glycation inhibiting^42^ and antiparasitic.^43^ Very recently, we have studied the electrochemical reduction of lawsone based xanthenes *via* chronoamperometry, cyclic voltammetry, and differential pulse voltammetry at a glassy carbon electrode.^44^ The above said properties have led to the synthesis of various naphthoquinone-based compounds which exhibited a variety of biological properties.^45,46^

**Scheme 1:**
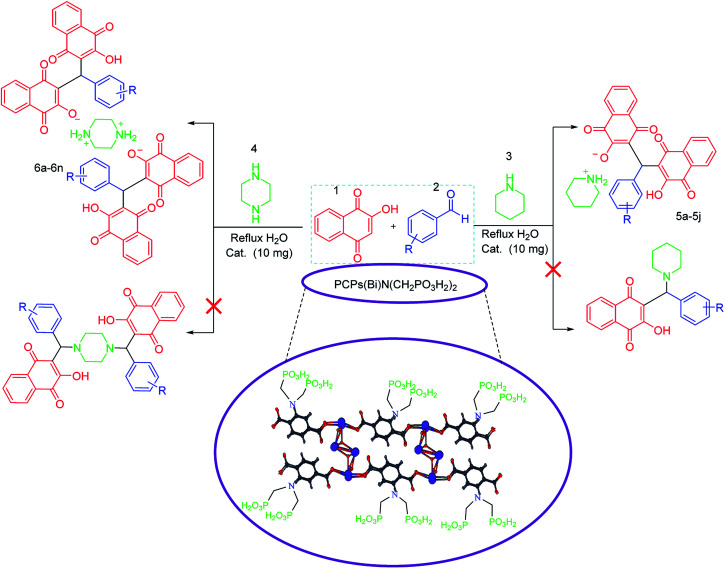
Synthesis of mono and bis naphthoquinone based salts of piperidine and/or piperazine using PCPs(Bi)N(CH_2_PO_3_H_2_)_2_.

Since that bismuth is the only one of that group to be non-toxic, we decided to apply it for synthesis of its corresponding porous coordination polymer with phosphorous acid tags. Therefore, herein, a novel porous coordination polymer containing bismuth (Bi) with phosphorous acid tags PCPs(Bi)N(CH_2_PO_3_H_2_)_2_ was synthesized. We used this material as a nano-porous catalyst for the synthesis of new naphthoquinone derivatives. Mono [piperidin-1-ium-1,4-dihydronaphthalen-2-olate] (5a–5j) and bis [piperazine-1,4-diium-1,4-dihydronaphthalen-2-olate] (6a–6n) henna-based salts of piperidine and/or piperazine were synthesized respectively, from the reaction of 2-hydroxynaphthalene-1,4-dione (henna), various aldehydes and piperazine or piperidine under reflux water condition (Scheme 1). As presented in the Scheme 1 the second reactions paths were not occurred.

## 2. Experimental

### 2.1. Materials and methods

The materials such as 2-aminoterephthalic acid (BDC-NH_2_) (Sigma-Aldrich, 99%), bismuth(iii) nitrate pentahydrate (Bi(NO_3_)·5H_2_O) (Merck, 95%), formic acid (HCOOH) (Merck, 37%), ethanol (C_2_H_5_OH) (Merck, 99%), lithium perchlorate (LiClO_4_) (Merck, 99%), 2-hydroxynaphthalene-1,4-dione (Sigma-Aldrich, 99%), piperidine and piperazine (Sigma-Aldrich, 99%) and other materials (Merck) were reagent-grade materials and used as received without further purification.

### 2.2. Instrumental measurements

From the model of the BRUKER Ultrashield FT-NMR spectrometer (*δ* in ppm) were recorded ^1^H NMR (600 or 400 MHz), ^13^C NMR (151 or 101 MHz) ^13^C NMR (DEPT-135), ^1^H^1^H Cosy, ^1^H^13^C HSQC, and ^1^H^13^C HMBC. Recorded on a Büchi B-545 apparatus in open capillary tubes were melting points. The PerkinElmer PE-1600-FTIR device was recorded for infrared spectra of compounds. SEM was performed using a scanning electron microscope for field publishing made by TE-SCAN. Thermal gravimetry (TG), differential thermal gravimetric (DTG) and differential thermal (DTA) were analyzed by a Perkin Elmer (Model: Pyris 1). BET and BJH were analyzed by BELSORP-mini ii high precision surface area and pore size. XRD was analyzed by ITAL STRUCTURE APD2000.

### 2.3. Electrochemical test

Cyclic voltammetry was performed using an Autolab model PGSTAT204 potentiostat/galvanostat. The working electrode used in the voltammetry experiments was a glassy carbon disc (3.2 mm^2^ area) and a platinum wire was used as a counter electrode. An Ag wire electrode was used as a reference electrode for all experiments and all potential values were reported with reference to ferrocene/ferrocenium couple (Fc/Fc^+^) as an internal standard. Tetrabutylammonium perchlorate was reagent-grade materials from Aldrich. Cyclic voltammetric measurements of each solution was carried out in a tetrabutylammonium perchlorate solution as the supporting electrolyte. A saturated solution of some product (5b, 6k, 5c, 6j, 5a and 6m) and tetrabutylammonium perchlorate (0.1 M) in DMSO, was put into the voltammetric cell and was deoxygenated with high-purity nitrogen (99.999%) for about 25 min. The background voltammograms were obtained by scanning the potential from 1.00 V to about −2.2 V.^47^

### 2.4. Catalytic tests

The mentioned naphthoquinone compounds were synthesized in the presence of PCPs(Bi)N(CH_2_PO_3_H_2_)_2_ as catalyst *via* one-pot reaction of 2-hydroxynaphthalene-1,4-dione (4 mmol or 2 mmol), piperazine or piperidine (1 mmol), aryl aldehyde (2 mmol or 1 mmol). The condensation of 2-hydroxynaphthalene-1,4-dione (2 mmol, 0.348 g), piperidine (1 mmol, 0.085 g) and benzaldehyde (1 mmol, 0.106 g) was determined as model reaction to optimize the reaction conditions (Table 1).

**Table 1:** Effect of different amounts of catalysts, temperature and solvent (5 mL) in the synthesis of naphthoquinone derivatives based on piperazine and piperidine

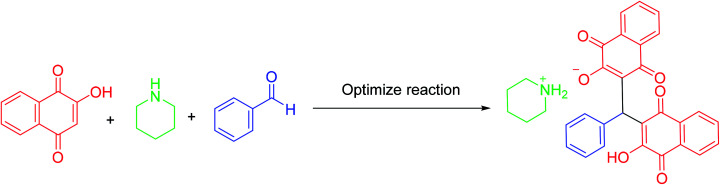					
Entry	Catalyst (mg)	Temp. (°C)	Solvent	Time (min)	Yield (%)
1	—	Reflux	H_2_O	90	Trace
2	1	Reflux	H_2_O	60	35
3	3	Reflux	H_2_O	45	50
4	5	Reflux	H_2_O	33	57
5	7	Reflux	H_2_O	15	78
**6**	**10**	**Reflux**	**H**_**2**_ **O**	**5**	**92**
7	15	Reflux	H_2_O	5	92
8	10	80	H_2_O	25	75
9	10	50	H_2_O	40	45
10	10	Rt	H_2_O	60	32
11	10	—	100	25	70
12	10	100	DMF	20	67
13	10	Reflux	EtOH	15	64
14	10	Reflux	CHCl_3_	35	35
15	10	Reflux	EtOAc	30	58
16	10	Reflux	CH_3_CN	20	60
17	10	Reflux	Toluene	90	Trace

### 2.5. Electrochemical test

**Fig. 1:**
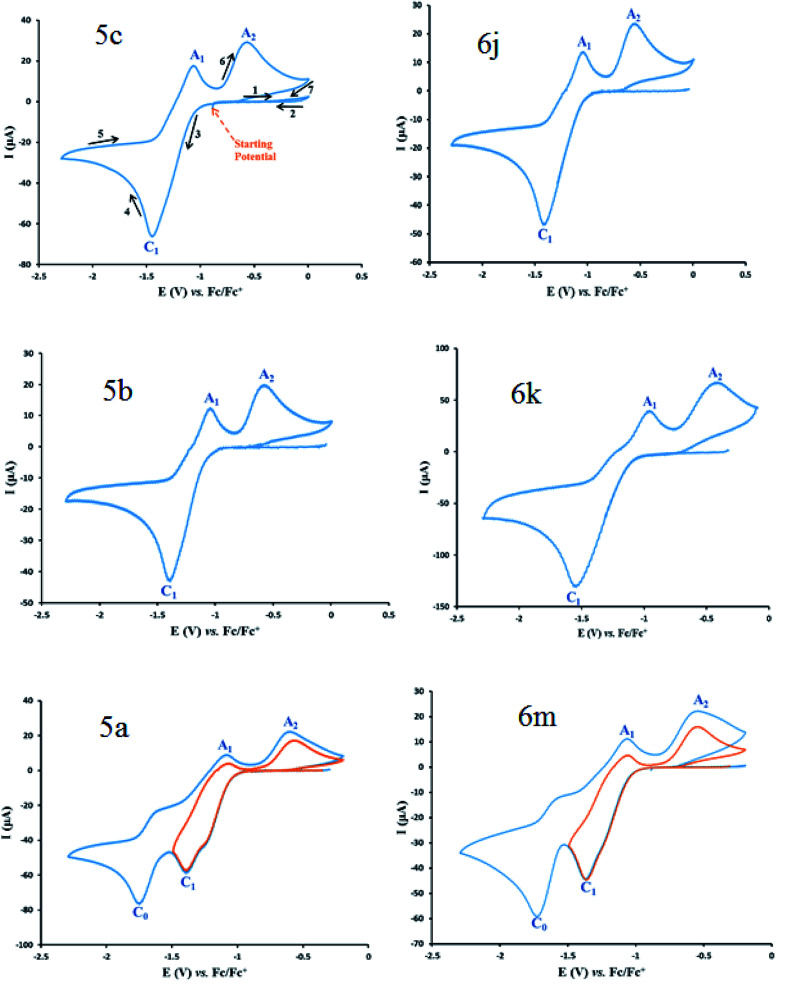
Cyclic voltammograms of a saturated solution of the product (5b, 6k, 5c, 6j, 5a and 6m) in DMSO containing tetra-*n*-butylammonium perchlorate (0.1 M), at glassy carbon electrode. Sweeping direction: oxidation of the obtained products at the first stage, reduction at the second stage and oxidation at the third stage. Scan rate: 100 mV s^−1^. Temperature = 25 ± 1 °C. Vectors show the sweeping direction.

**Scheme 2:**
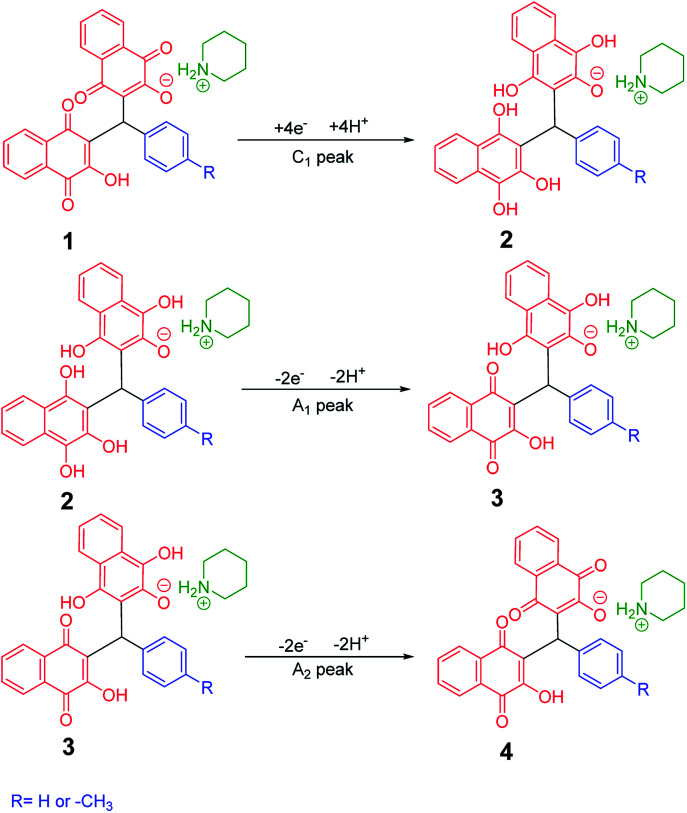
Proposed mechanism of electrochemical process in 5b and 5c cyclic voltammograms in Fig. 1.

**Scheme 3:**
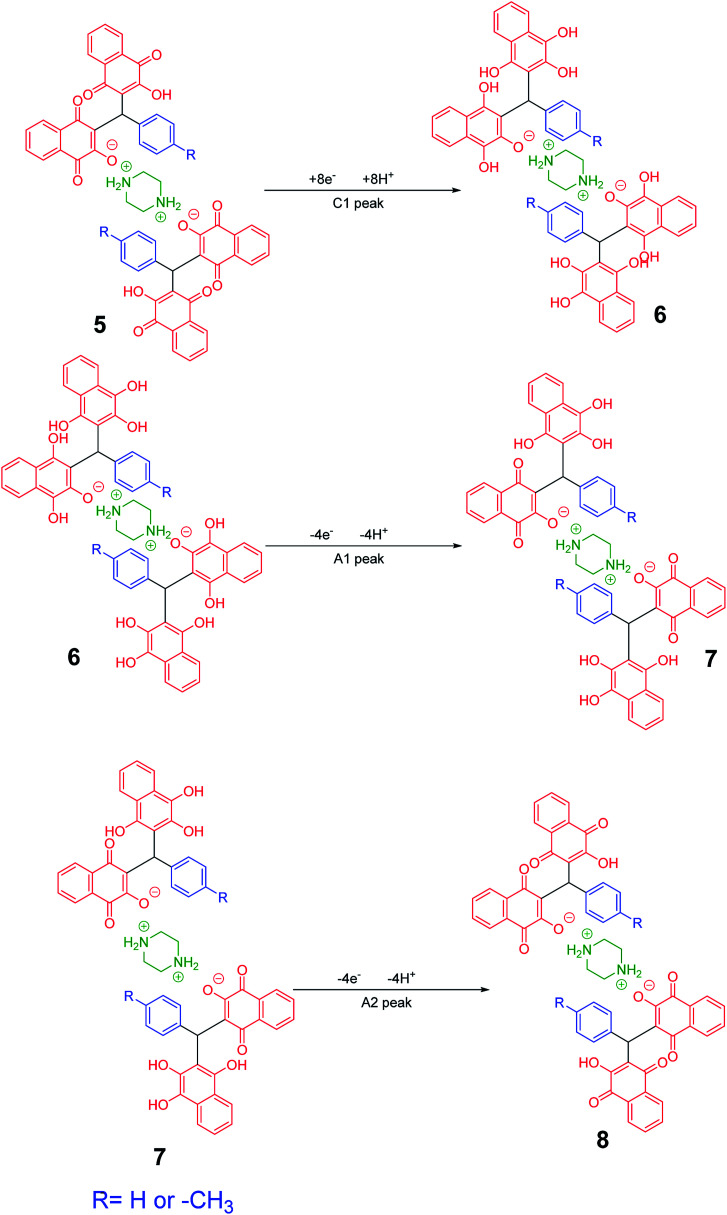
Proposed mechanism of electrochemical process in 6k and 6j cyclic voltammograms in Fig. 1.

Cyclic voltammograms of saturated solutions of 5b, 6k, 5c, 6j, 5a and 6m in DMSO are shown in Fig. 1. When the potential was scanned from −1.00 V to 0.00 V *vs.* Fc/Fc^+^, the cyclic voltammograms do not show any oxidation or reduction, but upon scanning the electrode potential from −1.00 V to a more negative voltage (−2.00 V), the cyclic voltammogram exhibit one (C_1_ for 5b, 6k, 5c, 6j, 5a) or two (C_0_ and C_1_ for 5a and 6m) cathodic and two anodic peaks (A_1_ and A_2_). In the cyclic voltammograms of 5b and 5c, the C_1_ peak is related to the reduction of 1 to 2 within a four-electron/four-proton process. The A_1_ and A_2_ peaks are related to the oxidation of 2 to 3 and 3 to 4 within a two-electron/two-proton process (Scheme 2).^48^ In the cyclic voltammograms of 6k and 6j, the C_1_ peak is related to the reduction of 5 to 6 within an eight-electron/eight-proton process, A_1_ and A_2_ peaks are related to the oxidation of 6 to 7 and 7 to 8 within a four-electron/four-proton process (Scheme 3).

**Scheme 4:**
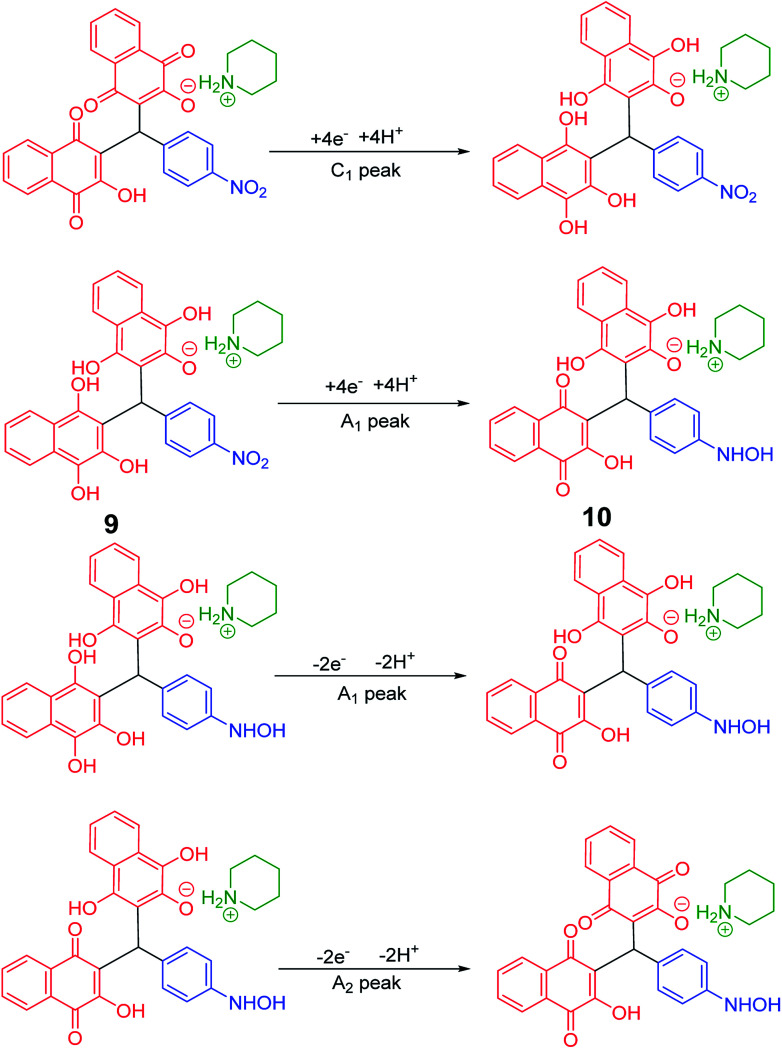
Proposed mechanism of the electrochemical process of 5a cyclic voltammogram in the Fig. 1.

**Scheme 5:**
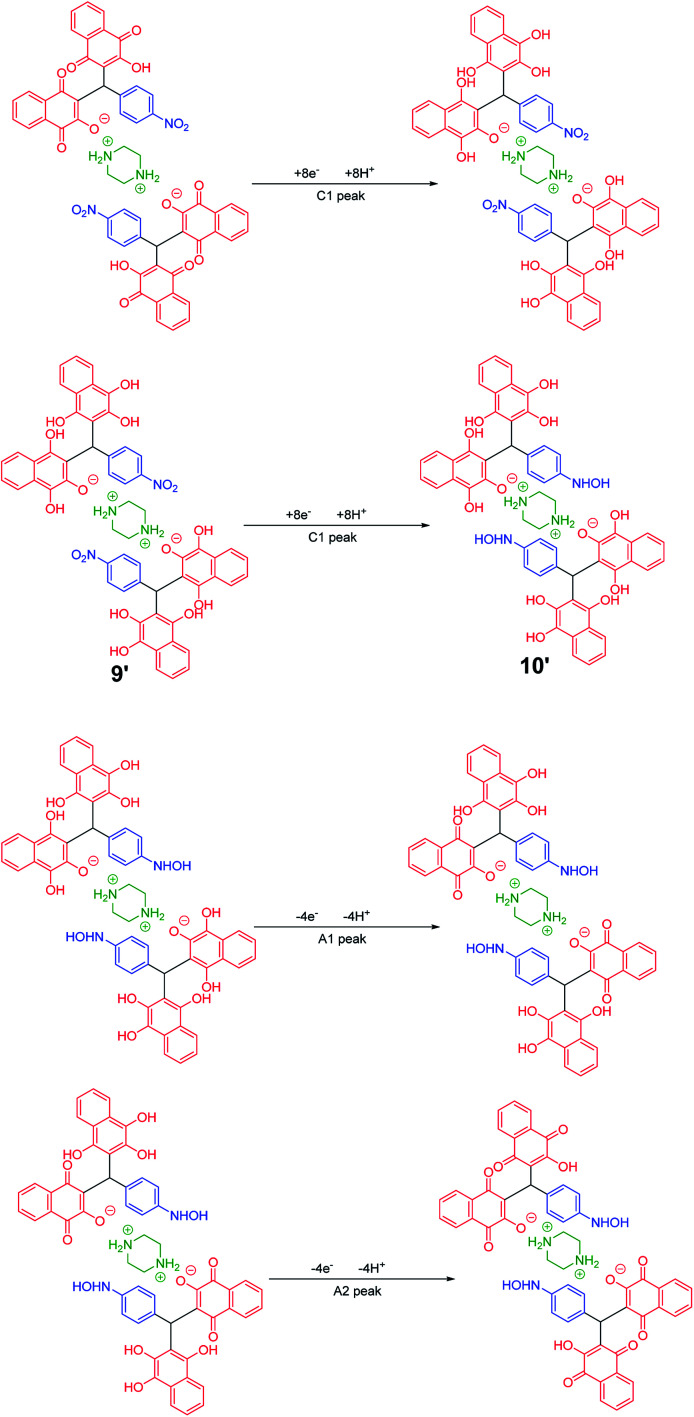
Proposed mechanism of the electrochemical process in 6m cyclic voltammogram in Fig. 1.

In the cyclic voltammograms of 5a and 6m, a new irreversible cathodic peak (C_0_) corresponding to the reduction of –NO_2_ to –NHOH functional group (9 and 9′ to 10 and 10′), appears at a more negative potential (Fig. 1) (Schemes 4 and 5).^49^

## 3. Result and discussion

In continuation of our investigation, PCPs(Bi)N(CH_2_PO_3_H_2_)_2_ was tested as nano heterogeneous catalyst for the synthesis of naphthoquinone derivatives based on piperazine and piperidine. As shown in Table 1, the best amount of catalyst was 10 mg PCPs(Bi)N(CH_2_PO_3_H_2_)_2_ and reflux in water lead to the best yield (Table 1, entry 6). Different amounts of catalyst and different temperatures did not improve the yield or reaction time (Table 1 entries 1–10). To investigate the solvent effect on the reaction improvement, several solvents such as H_2_O, DMF, EtOH, CHCl_3_, EtOAc, CH_3_CN, toluene (5 mL) and a solvent free reaction were tested and compared with reflux in water in the presence of 10 mg PCPs(Bi)N(CH_2_PO_3_H_2_)_2_ (Table 1, entries 11–17). The results are summarized in Table 1.

**Scheme 6:**
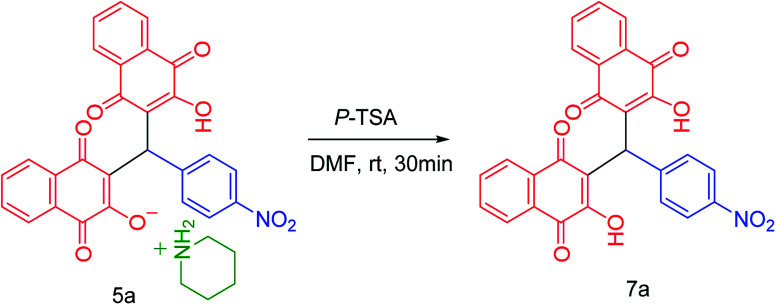
Synthesis of 7a*via* reaction of 5a.

**Fig. 2:**
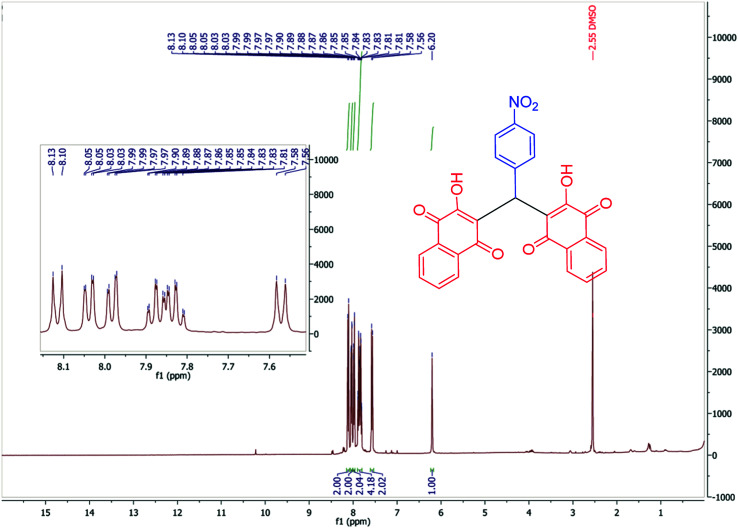
^1^H NMR spectrum of product 7a.

**Fig. 3:**
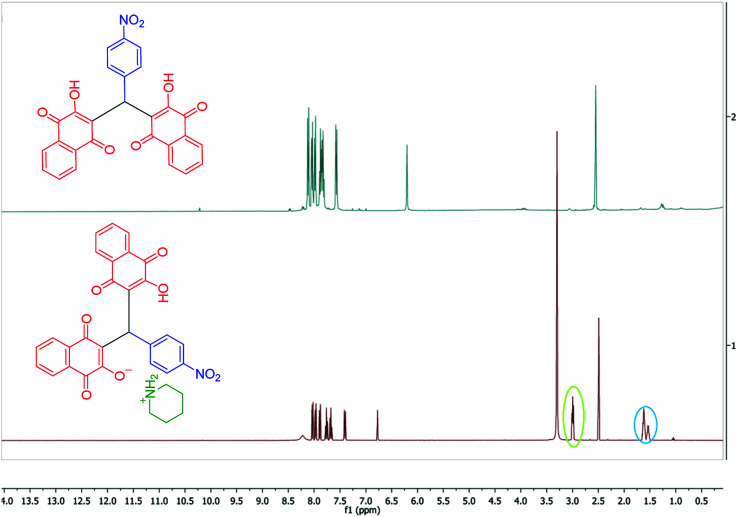
Comparison of the ^1^H NMR spectra of product 7a and 5a.

To confirm of the structure product 5a, the product was dissolved in ethanol in the present of *p*-TSA (1.0 eq.) which was stirred for 30 minutes (Scheme 6). After completing the reaction, the product was separated by filtration. By comparing the initial product 5a and the reaction product 7a we confirmed the salt structure of 5a. The structure of product 7a was confirmed by ^1^H NMR (Fig. 2 and 3) and by comparing its obtained physical data with those reported in the literature. We find, that the signals at 1.52–165 and 2.98–3.01 related to piperidinium cation is absent (Fig. 3).

### 3.1. Analytical data of compound 5a

**Scheme 7:**
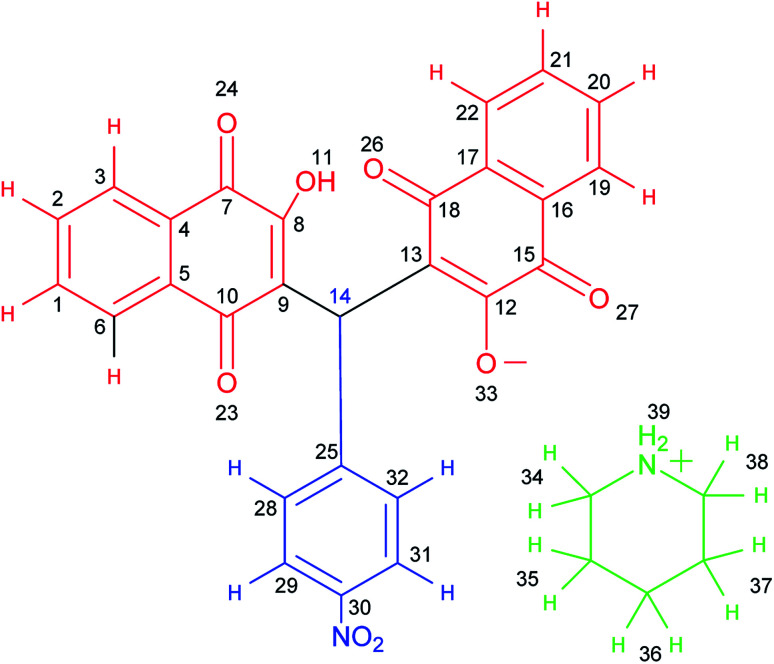
Structure of compound 5a.

After purification, the structures of the desired products (5a–5j) and (6a–6n) were fully characterized using various techniques such as FT-IR (Fig. S1†), ^1^H NMR (Fig. S2†), ^13^C NMR (DEPT-135) (Fig. S3†), ^1^H^1^H, COSY-NMR (Fig. S4–S6†), ^1^H^13^C, HSQC-NMR (Fig. S7 and S8†) and ^1^H^13^C, HMBC-NMR (Fig. S9–S12†). The structure of the compounds was presented in Scheme 7.

Orange solid; mp: 249–250 °C; IR (KBr): *υ* (cm^−1^) = 3433, 3216 (NH_2_), 3173 (C–H aromatic), 2929, 2854 (C–H aliphatic), 1676, 1638 (C=O), 1595, 1573, 1512, 1344 (NO_2_). ^1^H NMR (400 MHz, DMSO-*d*_6_) *δ* 8.22 (s, 2H), 8.03 (d, *J* = 8.8 Hz, 2H), 7.97 (d, *J* = 7.6 Hz, 2H), 7.89 (d, *J* = 7.4 Hz, 2H), 7.76 (t, *J* = 7.4 Hz, 2H), 7.68 (t, *J* = 7.4 Hz, 2H), 7.41 (d, *J* = 8.6 Hz, 2H), 6.77 (s, 1H), 3.02–2.97 (m, 4H), 1.62 (p, *J* = 5.7 Hz, 4H), 1.54 (q, *J* = 5.3 Hz, 2H). ^13^C NMR (DEPT-135) (100 MHz, DMSO-*d*_6_) *δ* 183.4, 182.2, 164.7, 150.8, 145.2, 133.9, 133.2, 132.0, 131.0, 128.1, 125.8, 125.3, 123.1, 121.6, 43.8, 33.7, 22.2, 21.6. ^1^H^1^H COSY-NMR ((400, 400) MHz, DMSO-*d*_6_) *δ* (8.04–8.02), (8.04–7.41), (8.02–7.41), (8.02–8.03), (7.98–7.97), (7.98–7.76), (7.96–7.76), (7.96–7.97), (7.90–7.90), (7.90–7.69), (7.88–7.69), (7.88–7.89), (7.78–7.77), (7.78–7.68), (7.78–7.98), (7.76–7.77), (7.74–7.98), (7.74–7.69), (7.74–7.77), (7.70–7.69), (7.70–7.76), (7.70–7.90), (7.68–7.69), (7.66–7.77), (7.66–7.90), (7.42–7.42), (7.42–8.04), (7.40–8.04), (7.39–7.40), (6.77–6.78), (3.30–3.31), (3.00–2.99), (2.98–1.63), (2.49–2.50), (1.61–1.54), (1.61–3.00), (1.60–1.62), (1.53–1.53), (1.51–1.63). ^1^H^13^C HSQC-NMR ((400, 100) MHz, DMSO-*d*_6_) *δ* (8.03–123.03), (7.97–125.81), (7.89–125.26), (7.76–133.92), (7.68–132.07), (7.41–128.15), (6.77–33.81), (2.99–43.87), (2.49–39.86), (1.62–22.28), (1.53–21.77). ^1^H^13^C, HMBC-NMR ((400, 100) MHz, DMSO-*d*_6_) *δ* (8.24–123.25), (8.11–125.68), (8.03–145.41), (8.03–151.00), (8.03–123.20), (7.97–131.61), (7.97–182.45), (7.90–134.43), (7.90–183.50), (7.76–133.36), (7.71–125.88), (7.70–125.73), (7.68–131.23), (7.41–128.33), (7.41–33.87), (7.41–145.40), (6.78–128.40), (6.78–182.43), (6.78–164.88), (6.78–151.01), (6.78–121.80), (3.00–44.04), (3.00–21.46), (3.00–23.01), (2.50–39.90), (1.63–44.12), (1.61–21.58), (1.54–22.43), (1.52–43.90).

## 4. Synthesis of porous coordination polymer (PCPs) (Bi)–BDC-NH_2_

**Scheme 8:**
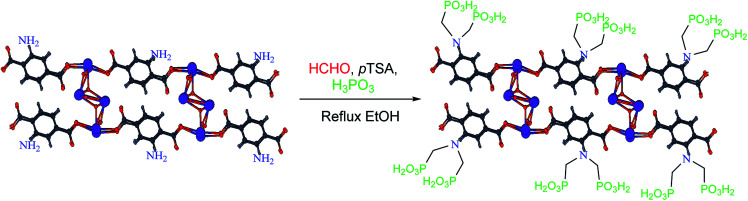
Synthesis of PCPs(Bi)–BDC-NH_2_ containing phosphorous acid functional groups.

In a Teflon-lined bomb, a mixture of Bi(NO_3_)·5H_2_O (1.0 mmol, 0.485 g) and 2-aminoterephthalic acid (2.0 mmol, 0.332 g) was putted in the oven at 180 °C for 5 days. Then the mixture was slowly cooled to room temperature. Then the porous coordination polymer containing bismuth formed a creamy precipitate which was washed with DMF to remove the unreacted ligand. The PCPs (Bi)–BDC-NH_2_ was isolated by vacuum filtration.^13^ Based on the data obtained from XRD, the structure and morphology of PCPs(Bi)–BDC-NH_2_ is similar to what was observed for PCPs(Bi)–BDC-NO_2_.^13^ After verifying the structure, the polymer was applied for the synthesis of PCPs(Bi)N(CH_2_PO_3_H_2_)_2_. In a 50 mL round-bottomed flask PCPs(Bi)–BDC-NH_2_ (1.0 g), paraformaldehyde (8.0 mmol, 0.54 g), phosphorous acid (8.0 mmol, 1.476 g), *p*-TSA (1.0 mmol, 0.172 g) and ethanol (15 mL) were mixed and refluxed for 18 hours. After this time, a yellow precipitate was isolated by centrifugation (1000 rpm, 10 min). The residue was dried under vacuum to obtain PCPs(Bi)N(CH_2_PO_3_H_2_)_2_ (1.65 g) (Scheme 8).

In continuation our investigations for developing of the knowledge of catalytic systems, catalytic systems and organic methodology, herein we wish to expand the porous catalysts. With this aim, we have prepared a novel functionalized porous coordination polymer (PCPs) linked to phosphorous acid groups in the presence *p*-TSA and paraformaldehyde under ethanol reflux (Scheme 8). PCPs(Bi)N(CH_2_PO_3_H_2_)_2_ was characterized by FT-IR and XRD spectroscopy, elemental mapping analysis (DEX), the scanning electron microscopy (SEM), N_2_ adsorption–desorption isotherm (BET), thermal gravimetric (TG), derivative thermal gravimetric (DTG), differential thermal (DTA), transmission electron microcopy (TEM).

**Fig. 4:**
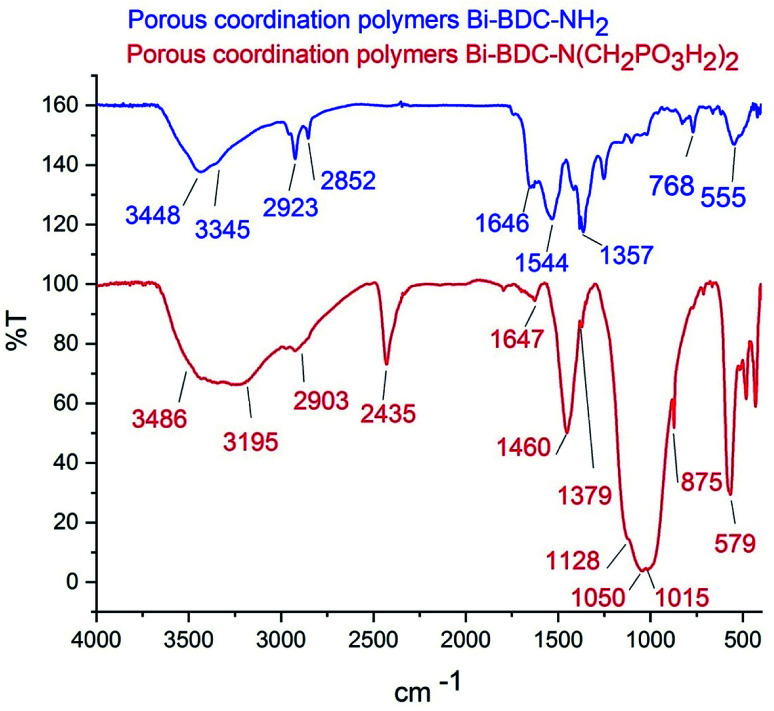
FT-IR spectra of PCPs(Bi)BDC-NH_2_ and PCPs(Bi)N(CH_2_PO_3_H_2_)_2_ in KBr.

The FT-IR spectra of PCPs(Bi)BDC-NH_2_ and PCPs(Bi)N(CH_2_PO_3_H_2_)_2_ were compared in Fig. 4. The broad peak 2600–3500 cm^−1^ is related to OH of PO_3_H_2_ functional groups. The aromatic C–H and C=C stretches bands are respectively at 2923 and 1647 cm^−1^. The absorption bands at 1015 and 1050 cm^−1^ are related to P–O bond stretching and the band at 1128 cm^−1^ is related to P=O.^50^ Furthermore, peaks of Bi–O of octahedral BiO_6_ appeared at 875 and 579 cm^−1^ respectively^51^ (Fig. 4). The FT-IR spectrum difference between PCPs(Bi)BDC-NH_2_ and PCPs(Bi)N(CH_2_PO_3_H_2_)_2_ verified the structure of the catalyst.

**Fig. 5:**
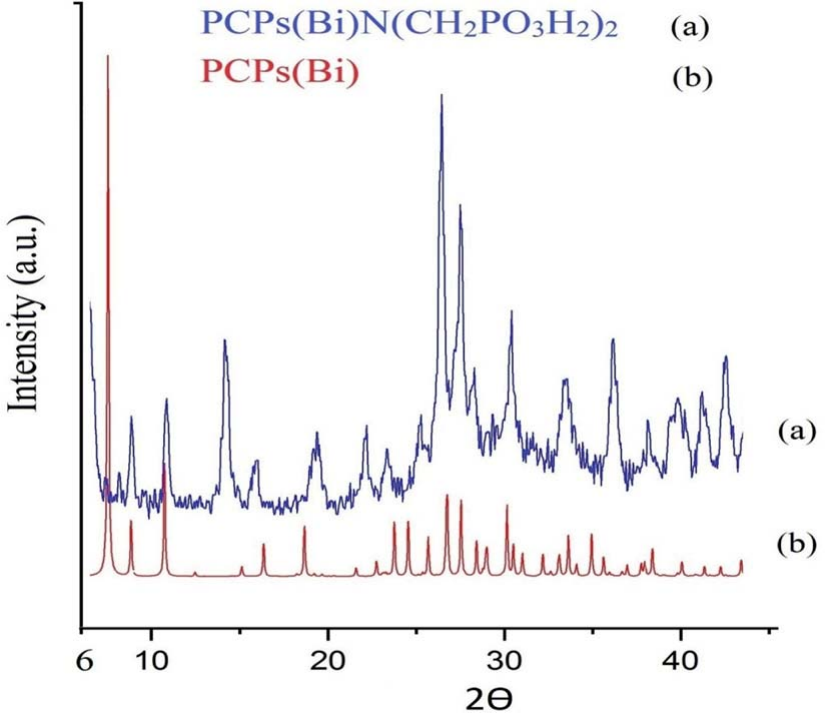
Comparison XRD patterns of the PCPs(Bi) and PCPs(Bi)N(CH_2_PO_3_H_2_).

**Fig. 6:**
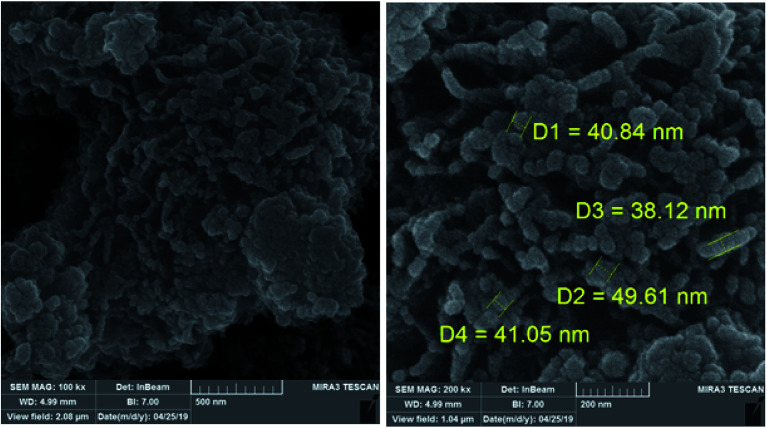
SEM of PCPs(Bi)N(CH_2_PO_3_H_2_)_2_.

The morphology and structure of the of PCPs(Bi)N(CH_2_PO_3_H_2_) was studied using XRD (Fig. 5). PCPs(Bi)^13^ and PCPs(Bi)N(CH_2_PO_3_H_2_) were compared in the range of 6–44° using XRD as shown in Fig. 5. The peaks of Bi–O of PCPs(Bi) in 2*θ* = 8.8, 10.76, 18.6, 26.8 and 27.6 and PO_3_H_2_ in 2*θ* = 23.4, 30.2, 33.7 and 36.2 are observed in the XRD pattern, which is consistent with previously paper.^52,53^ The SEM images of the PCPs(Bi)N(CH_2_PO_3_H_2_)_2_ catalyst reveal that the particles shape resembles cauliflowers and the particle size is within the range of the 38–49 nm (Fig. 6).

**Fig. 7:**
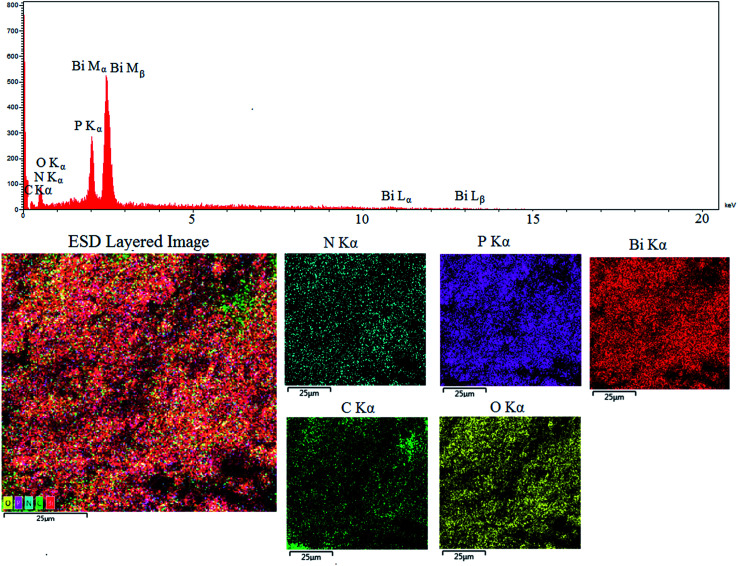
Energy-dispersive X-ray spectroscopy (EDX), elemental mapping analysis of PCPs(Bi)N(CH_2_PO_3_H_2_)_2_.

The present elements of PCPs(Bi)N(CH_2_PO_3_H_2_)_2_ can be seen using energy-dispersive X-ray spectroscopy (EDX) and elemental mapping analysis and in the energy-dispersive X-ray spectroscopy (EDX) mode in scanning electron microscopy (SEM). In EDX elemental mapping analysis, bismuth (61.82%), carbon (8.56%), nitrogen (2.62%), oxygen (17.24%) and phosphor (9.77%) were confirmed in the structure of PCPs(Bi)N(CH_2_PO_3_H_2_)_2_ (Fig. 7).

**Fig. 8:**
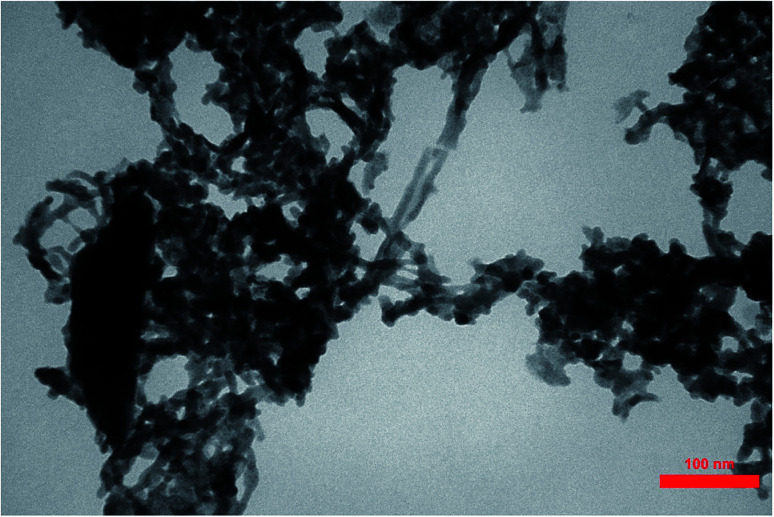
TEM of PCPs(Bi)N(CH_2_PO_3_H_2_)_2_.

Transmission Electron Microcopy (TEM) images of PCPs(Bi)N(CH_2_PO_3_H_2_)_2_ are presented in Fig. 8. In agreement with SEM a cross-linked nanostructure of PCPs-catalyst was confirmed. The particles of PCPs(Bi)N(CH_2_PO_3_H_2_)_2_ are approximately 30–50 nm with a narrow size distribution.

Nitrogen adsorption–desorption isotherm after Brunauer Emmett and Teller (BET) of PCPs(Bi)N(CH_2_PO_3_H_2_)_2_ was also studied in Fig. S13.† Mean pore diameter, BET surface area, and total pore volume of PCPs(Bi)N(CH_2_PO_3_H_2_)_2_ are 38.8 nm, 22 m^2^ g^−1^ and 0.2 cm^3^ g^−1^.

**Fig. 9:**
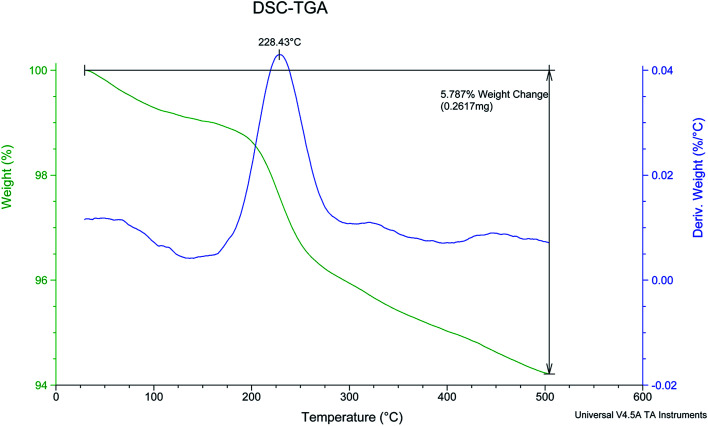
TG and DTG analysis of PCPs(Bi)N(CH_2_PO_3_H_2_)_2_.

Thermal Gravimetric (TG) and derivative thermal gravimetric (DTG) analysis of PCPs(Bi)N(CH_2_PO_3_H_2_)_2_ are shown in the Fig. 9. Two decline stages were observed for PCPs(Bi)N(CH_2_PO_3_H_2_)_2_ in the Fig. 9. The weight loss was related to evaporation of solvent (organic and water). Based on previously work, the weight loss of the first step in TG patterns is related to the exit of solvents used in the course of synthesis of described catalyst.^54–56^ The results show that the catalyst can be used up to 228 °C. At this temperature to the polymer exhausts PO_3_H_2_ groups and the structure of PCPs(Bi)N(CH_2_PO_3_H_2_)_2_ is decomposed (Fig. 9).

## 5. Catalytic properties of (PCPs) (Bi)–BDC-NH_2_

### 5.1. Synthesis of mono and bis naphthoquinone based on salts of piperidine

In a 25 mL round-bottomed flask, a mixture of 2-hydroxynaphthalene-1,4-dione (2.0 mmol, 0.348 g), piperidine (1.0 mmol, 0.085 g), aryl aldehyde (1.0 mmol) and PCPs(Bi)N(CH_2_PO_3_H_2_)_2_ (10 mg) as catalyst were stirred in an aqueous solution under reflux condition. After completion of the reaction which was detected by thin layer chromatography (TLC) (*n*-hexane/EtOAc; 1 : 2), the reaction mixture was cooled to room temperature. Then, warm acetone (10 mL) was added to the mixture for separation of the catalyst by centrifugation (1000 rpm, 10 min). Then, the obtained product was washed with water/ethanol (1 : 1) Scheme 1.

### 5.2. Synthesis of mono and bis naphthoquinone based on salts of piperazine

In a 25 mL round-bottomed flask, a mixture of 2-hydroxynaphthalene-1,4-dione (4.0 mmol, 0.696 g), piperazine (1.0 mmol, 0.086 g), aryl aldehyde (2.0 mmol) and PCPs(Bi)N(CH_2_PO_3_H_2_)_2_ (10 mg) as catalyst were stirred in an aqueous solution under reflux condition. After completion of the reaction which was detected by thin layer chromatography (TLC) (*n*-hexane/EtOAc; 1 : 2), the reaction mixture was cooled to room temperature. Then, warm acetone (10 mL) was added to the mixture for separation of the catalyst by centrifugation (1000 rpm, 10 min). Then, the obtained product was washed with water/ethanol (1 : 1) Scheme 1.

### 5.3. Catalytic potential of PCPs(Bi)N(CH_2_PO_3_H_2_)_2_

We further investigated the efficiency of PCPs(Bi)N(CH_2_PO_3_H_2_)_2_ for the synthesis of 5b for the reaction of 2-hydroxynaphthalene-1,4-dione (2.0 mmol, 0.348 g), piperidine (1.0 mmol, 0.085 g) and benzaldehyde (1.0 mmol, 0.106 g) under the above mentioned optimized reaction conditions. Various organic and inorganic acid catalysts for the above reaction were also tested for comparison (Table 2). As Table 2 indicates, PCPs(Bi)N(CH_2_PO_3_H_2_)_2_ is the best catalyst for the synthesis of naphthoquinone derivatives as salts of piperazine or piperidine, due to the shorter reaction times, higher yields and amount of applied catalyst.

**Table 2:** Evaluation of various catalyst for the synthesis of 5b in comparison with PCPs(Bi)N(CH_2_PO_3_H_2_)_2_ under water reflux

Entry	Catalyst	(mol% or mg)	Time (h)	Yield (%)	TON	TOF
**1**	[Py-SO_3_H]Cl^57^	10	1.5	62	620	413.3
**2**	NH_4_NO_3_	10	5	—	—	—
**3**	CF_3_SO_3_H	10	5	Trace	—	—
**4**	Al(HSO_4_)_3_	10	5	37	370	74
**5**	H_3_[p(W_3_O_10_)_4_]·*X*H_2_O	10	4	30	300	75
**6**	Mg(NO_3_)_2_·6H_2_O	10	4	—	—	
**7**	GTBSA^58^	10	2	68	680	340
**8**	*p*-TSA	10	5	Trace	—	—
**9**	Trichloroisocyanuric acid	10	5	32	320	64
**10**	H_3_PO_3_	10	1.5	73	730	486
**11**	[Fe_3_O_4_@SiO_2_@Pr-DABCO-SO_3_H]Cl_2_ (ref. 59)	10	2	72	—	—
**12**	APVPB^60^	10 mg	4	58	—	—
**13**	Fe_3_O_4_	10 mg	5	—	—	—
**14**	SBISAC^61^	10 mg	2	55	—	—
**15**	SSA^62^	10 mg	1.5	45	—	—
**16**	[PVI-SO_3_H]Cl^63^	10 mg	1	56	—	—
**17**	PCPs(Bi)BDC-NH_2_	10 mg	4	24	—	—
**18**	**PCPs(Bi)N(CH**_**2**_ **PO**_**3**_ **H**_**2**_ **)**_**2**_	**10 mg**	**5 (min)**	**95**	—	—

Then, PCPs(Bi)N(CH_2_PO_3_H_2_)_2_ was tested as heterogeneous catalyst. The efficiency and applicability were studied for the reaction of hydroxynaphthalene-1,4-dione (4 mmol or 2 mmol), piperazine or piperidine (1.0 mmol), aryl aldehyde (2.0 mmol or 1.0 mmol). As shown in Table 2, this method is suitable for the synthesis of naphthoquinone derivatives based on piperazine and piperidine (5a–5j) and (6a–6n) (Table 3) in high to excellent yields (82–95%) with in relatively short reaction times (4–30 min). As shown in Table 3, all aldehydes including benzaldehyde as well as other aromatic aldehydes possessing electron-releasing substituents, electron withdrawing substituents, basic groups or halogens afforded the desired naphthoquinone compounds as salts of piperazine or piperidine.

**Table 3:** Synthesis of piperidin-1-ium 3-((3-hydroxy-1,4-dioxo-1,4-dihydronaphthalen-2-yl)(phenyl)methyl)-1,4-dioxo-1,4-dihydronaphthalen-2-olate (5a–5j) and piperazine-1,4-diium 3-((1,4-dioxo-1,4-dihydronaphthalen-2-yl)(phenyl)methyl)-1,4-dioxo-1,4-dihydronaphthalen-2-olate derivatives in the presence of PCPs(Bi)N(CH_2_PO_3_H_2_)_2_

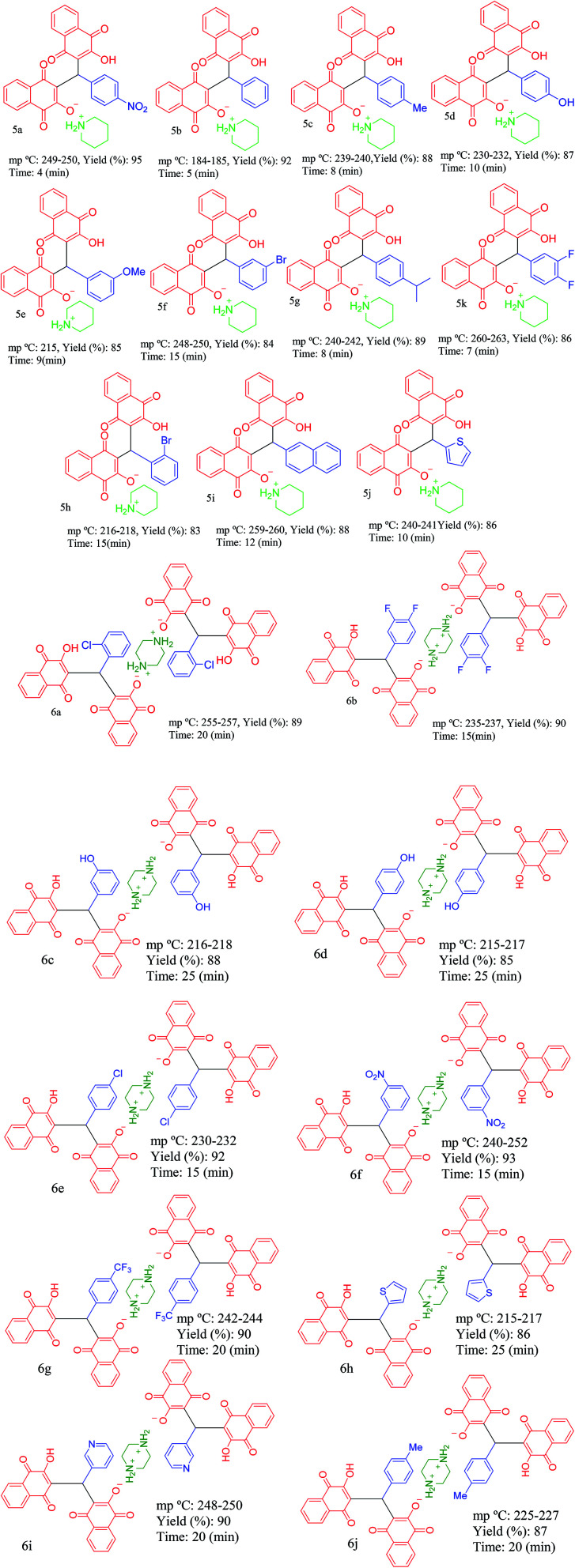

**Fig. 10:**
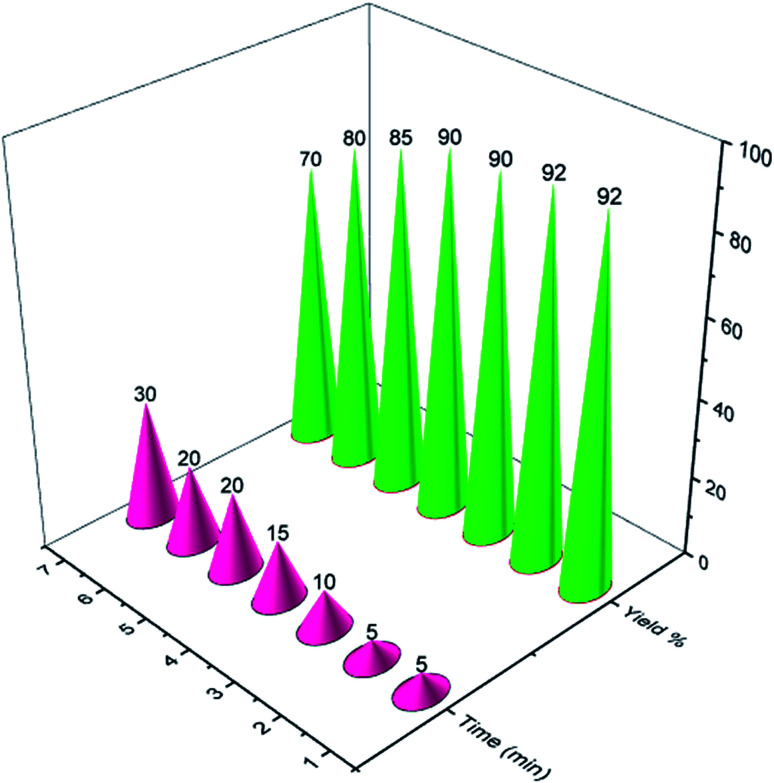
Recyclability of PCPs(Bi)N(CH_2_PO_3_H_2_)_2_ for the synthesis naphthoquinone derivatives.

Finally, for checking the reusability of the described catalyst for the synthesis of naphthoquinone derivatives, PCPs(Bi)N(CH_2_PO_3_H_2_)_2_ was examined in a model reaction. We used 2-hydroxynaphthalene-1,4-dione (2.0 mmol, 0.348 g), piperidine (1.0 mmol, 0.085 g) and benzaldehyde (1.0 mmol, 0.106 g). The results show that the catalyst has the potential to be reused up to 6 times without significant yield reduction (Fig. 10).

## 6. Conclusion

In summary, a porous coordinated polymer (PCPs) was designed, synthesized and applied as a novel, efficient and functionalized phosphorus acid PCPs(Bi)N(CH_2_PO_3_H_2_)_2_ catalyst. PCPs(Bi)N(CH_2_PO_3_H_2_)_2_ was prepared using a reaction of PCPs(Bi)BDC-NH_2_, paraformaldehyde and phosphorus acid (H_3_PO_3_). The described coordinated polymer PCPs(Bi)N(CH_2_PO_3_H_2_)_2_ was also tested as a porous catalyst for the synthesis of new mono and bis-naphthoquinone-based salts were characterized with ^1^H, ^13^C NMR, ^13^C NMR (DEPT-135), ^1^H^1^H Cosy, ^1^H^13^C HSQC, ^1^H^13^C HMBC, FT-IR and HR-Mass. Since that henna-based salts compounds are good candidates for electrochemical investigation, the electrochemical action of the obtained products was also investigated and presented. The present work can open up a new perspective in the course of rational design, synthesis and applications of task-specific biological based hennotannic acid (2-hydroxynaphthalene-1,4-dione or henna) based molecules. Some of the major advantages of this work are high yield of products, short reaction times, facile workup and reusability of the presented catalyst.

## Conflicts of interest

There are no conflicts to declare.

## Supplementary Material

RA-011-D0RA06674E-s001
