# Supplementary material for: Synthesis of biological based hennotannic acid-based salts over porous bismuth coordination polymer with phosphorous acid tags
Source: RSC Adv. 2021 Jan 7;11(4):2141–57. doi: 10.1039/d0ra06674e (PMC8693640; doi:10.1039/d0ra06674e)
Supplement: RA-011-D0RA06674E-s001 [file RA-011-D0RA06674E-s001.pdf]

Electronic Supplementary Material (ESI) for RSC Advances.
This journal is © The Royal Society of Chemistry 2020

Supporting information

Synthesis of biological based hennotannic acid-based salts over porous bismuth coordination polymer with phosphorous acid tags

Saeed Babaee,[a] Mahmoud Zarei,*[a] Mohammad Ali Zolfigol,*[a] Sadegh Khazalpour,[b] Masoumeh Hasani,[b] Uwe Rinner,[c] Romana Schirhagl,[d] Neda Norouzi[d], Sadegh Rostamnia[e]

[a] Department of Organic Chemistry, Faculty of Chemistry, Bu-Ali Sina University, PO Box 6517838683, Hamedan, Iran. Tel: +988138282807, Fax: +988138380709 Iran. E-Mail: mahmoud8103@yahoo.com (M. Zarei), zolfi@basu.ac.ir, mzolfigol@yahoo.com (M. A. Zolfigol) & saeed.babai@yahoo.com (Saeed Babaee).

[b] Department of Analytical Chemistry, Faculty of Chemistry, Bu-Ali Sina University, Hamedan, Iran. khazalpour@yahoo.com (Sadegh Khazalpour) & hasani@basu.ac.ir (Masoumeh Hasani).

[c] Department of Life Sciences, IMC University of Applied Sciences, Piaristengasse 1,3500 Krems, Austria. E-Mail: uwe.rinner@fh-krems.ac.at

[d] University Medical Center Groningen, Groningen University, Antonius Deusinglaan 1, 9713 AV Groningen, Netherlands. romana.schirhagl@gmail.com (Romana Schirhagl) & n.norouzi2018@gmail.com (Neda Norouzi)

[b] Organic and Nano Group (ONG), Department of Chemistry, Faculty of Science, University of Maragheh, PO Box 55181-83111, Maragheh, Iran. E-Mail: rostamnia@maragheh.ac.ir (Sadegh Rostamnia).

Table of Contents

Figure S1: FT-IR spectrum of compound 5a S12

Figure S2: ^{1}H NMR spectrum of compound 5a S12

Figure S3: ^{13}C NMR (DEPT-135) spectrum of compound 5a S13

Figure S4: ^{1}H^{1}H, COSY-NMR spectrum of compound 5a S13

Figure S5: ^{1}H^{1}H, COSY-NMR expand spectrum of compound 5a S14

Figure S6: ^{1}H^{1}H, COSY-NMR expand spectrum of compound 5a S14

Figure S7: ^{1}H^{13}C HSQC-NMR spectrum of compound 5a S15

Figure S8: ^{1}H^{13}C, HSQC-NMR expand spectrum of compound 5a S15

Figure S9: ^{1}H^{13}C HMBC-NMR spectrum of compound 5a S16

Figure S10: ^{1}H^{13}C HMBC-NMR expand spectrum of compound 5a S16

Figure S11: ^{1}H^{13}C HMBC-NMR expand spectrum of compound 5a S17

Figure S12: ^{1}H^{13}C HMBC-NMR expand spectrum of compound 5a S17

Figure S13: Nitrogen adsorption-desorption isotherm of PCPs(Bi)N($CH_2PO_3H_2$)$_2$ S18

Suggested mechanism for the synthesis of napthoquinone derivatives using PCPs(Bi)N($CH_2PO_3H_2$)$_2$ S18

Scheme S1: Proposed mechanism for the synthesis napthoquinone derivatives using PCPs(Bi)N($CH_2PO_3H_2$)$_2$ S19

Piperidin-1-ium 3-((3-hydroxy-1,4-dioxo-1,4-dihydronaphthalen-2-yl)(4-nitrophenyl)methyl)-1,4-dioxo-1,4-dihydronaphthalen S20

Piperidin-1-ium 3-((3-hydroxy-1,4-dioxo-1,4-dihydronaphthalen-2-yl)(phenyl)methyl)-1,4-dioxo-1,4-dihydronaphthalen-2-olate S21

Piperidin-1-ium 3-((3-hydroxy-1,4-dioxo-1,4-dihydronaphthalen-2-yl)(p-tolyl)methyl)-1,4-dioxo-1,4-dihydronaphthalen-2-olate S21

Piperidin-1-ium 3-((3-hydroxy-1,4-dioxo-1,4-dihydronaphthalen-2-yl)(4-hydroxyphenyl)methyl)-1,4-dioxo-1,4-dihydronaphthalen-2-olate S22

Piperidin-1-ium 3-((3-hydroxy-1,4-dioxo-1,4-dihydronaphthalen-2-yl)(3-methoxyphenyl)methyl)-1,4-dioxo-1,4-dihydronaphthalen-2-olate 23

Piperidin-1-ium 3-((3-bromophenyl)(3-hydroxy-1,4-dioxo-1,4-dihydronaphthalen-2-yl)methyl)-1,4-dioxo-1,4-dihydronaphthalen-2-olate S24

Piperidin-1-ium 3-((3-hydroxy-1,4-dioxo-1,4-dihydronaphthalen-2-yl)(4-isopropylphenyl)methyl)-1,4-dioxo-1,4-dihydronaphthalen-2-olate 25

Piperidin-1-ium 3-((3,4-difluorophenyl)(3-hydroxy-1,4-dioxo-1,4-dihydronaphthalen-2-yl)methyl)-1,4-dioxo-1,4-dihydronaphthalen-2-olate S26

Piperidin-1-ium 3-((2-bromophenyl)(3-hydroxy-1,4-dioxo-1,4-dihydronaphthalen-2-yl)methyl)-1,4-dioxo-1,4-dihydronaphthalen-2-olate 27

Piperidin-1-ium 3-((3-hydroxy-1,4-dioxo-1,4-dihydronaphthalen-2-yl)(naphthalen-2-yl)methyl)-1,4-dioxo-1,4-dihydronaphthalen-2-olate S28

Piperidin-1-ium 3-((3-hydroxy-1,4-dioxo-1,4-dihydronaphthalen-2-yl)(thiophen-2-yl)methyl)-1,4-dioxo-1,4-dihydronaphthalen-2-olate S29

Piperazine-1,4-diium 3-((2-chlorophenyl)(3-hydroxy-1,4-dioxo-1,4-dihydronaphthalen-2-yl)methyl)-1,4-dioxo-1,4-dihydronaphthalen-2-olate S30

Piperazine-1,4-diium 3-((3,4-difluorophenyl)(3-hydroxy-1,4-dioxo-1,4-dihydronaphthalen-2-yl)methyl)-1,4-dioxo-1,4-dihydronaphthalen-2-olate S31

Piperazine-1,4-diium 3-((3-hydroxy-1,4-dioxo-1,4-dihydronaphthalen-2-yl)(3-hydroxyphenyl)methyl)-1,4-dioxo-1,4-dihydronaphthalen-2-olate S31

Piperazine-1,4-diium 3-((3-hydroxy-1,4-dioxo-1,4-dihydronaphthalen-2-yl)(4-hydroxyphenyl)methyl)-1,4-dioxo-1,4-dihydronaphthalen-2-olate S32

Piperazine-1,4-diium 3-((4-chlorophenyl)(3-hydroxy-1,4-dioxo-1,4-dihydronaphthalen-2-yl)methyl)-1,4-dioxo-1,4-dihydronaphthalen-2-olate S32

Piperazine-1,4-diium 3-((3-hydroxy-1,4-dioxo-1,4-dihydronaphthalen-2-yl)(3-nitrophenyl)methyl)-1,4-dioxo-1,4-dihydronaphthalen-2-olate S32

Piperazine-1,4-diium 3-((3-hydroxy-1,4-dioxo-1,4-dihydronaphthalen-2-yl)(4-(trifluoromethyl)phenyl)methyl)-1,4-dioxo-1,4-dihydronaphthalen-2-olate........ S33

Piperazine-1,4-diium 3-((3-hydroxy-1,4-dioxo-1,4-dihydronaphthalen-2-yl)(thiophen-2-yl)methyl)-1,4-dioxo-1,4-dihydronaphthalen-2-olate S33

Piperazine-1,4-diium 3-((3-hydroxy-1,4-dioxo-1,4-dihydronaphthalen-2-yl)(pyridin-3-yl)methyl)-1,4-dioxo-1,4-dihydronaphthalen-2-olate S34

Piperazine-1,4-diium 3-((3-hydroxy-1,4-dioxo-1,4-dihydronaphthalen-2-yl)(p-tolyl)methyl)-1,4-dioxo-1,4-dihydronaphthalen-2-olate........ S34

Piperazine-1,4-diium 3-((3-hydroxy-1,4-dioxo-1,4-dihydronaphthalen-2-yl)(phenyl)methyl)-1,4-dioxo-1,4-dihydronaphthalen-2-olate........ S35

Piperazine-1,4-diium 3-((3,4-dimethoxyphenyl)(3-hydroxy-1,4-dioxo-1,4-dihydronaphthalen-2-yl)methyl)-1,4-dioxo-1,4-dihydronaphthalen-2-olate........ S35

Piperazine-1,4-diium 3-((3-hydroxy-1,4-dioxo-1,4-dihydronaphthalen-2-yl)(4-nitrophenyl)methyl)-1,4-dioxo-1,4-dihydronaphthalen-2-olate S35

Piperazine-1,4-diium 3-((3-hydroxy-1,4-dioxo-1,4-dihydronaphthalen-2-yl)(4-isopropylphenyl)methyl)-1,4-dioxo-1,4-dihydronaphthalen-2-olate........ S36

FT-IR spectrum of piperidin-1-ium 3-((3-hydroxy-1,4-dioxo-1,4-dihydronaphthalen-2-yl)(phenyl)methyl)-1,4-dioxo-1,4-dihydronaphthalen-2-olate S37

^{1}H NMR spectrum of piperidin-1-ium 3-((3-hydroxy-1,4-dioxo-1,4-dihydronaphthalen-2-yl)(phenyl)methyl)-1,4-dioxo-1,4-dihydronaphthalen-2-olate S38

^{13}C NMR (DEPT-135) spectrum of piperidin-1-ium 3-((3-hydroxy-1,4-dioxo-1,4-dihydronaphthalen-2-yl)(phenyl)methyl)-1,4-dioxo-1,4-dihydronaphthalen-2-olate...... S39

^{1}H^{1}H, COSY-NMR spectrum of piperidin-1-ium 3-((3-hydroxy-1,4-dioxo-1,4-dihydronaphthalen-2-yl)(phenyl)methyl)-1,4-dioxo-1,4-dihydronaphthalen-2-olate...... S40

^{1}H^{13}C, HSQC-NMR spectrum of piperidin-1-ium 3-((3-hydroxy-1,4-dioxo-1,4-dihydronaphthalen-2-yl)(phenyl)methyl)-1,4-dioxo-1,4-dihydronaphthalen-2-olate...... S41

$^{1}H^{13}C$, HMBC-NMR spectrum of piperidin-1-ium 3-((3-hydroxy-1,4-dioxo-1,4-dihydronaphthalen-2-yl)(phenyl)methyl)-1,4-dioxo-1,4-dihydronaphthalen-2-olate...... S42

FT-IR spectrum of piperidin-1-ium 3-((3-hydroxy-1,4-dioxo-1,4-dihydronaphthalen-2-yl)(phenyl)methyl)-1,4-dioxo-1,4-dihydronaphthalen-2-olate S43

^{1}H NMR spectrum of piperidin-1-ium 3-((3-hydroxy-1,4-dioxo-1,4-dihydronaphthalen-2-yl)(phenyl)methyl)-1,4-dioxo-1,4-dihydronaphthalen-2-olate S44

^{13}C NMR (DEPT-135) spectrum of piperidin-1-ium 3-((3-hydroxy-1,4-dioxo-1,4-dihydronaphthalen-2-yl)(phenyl)methyl)-1,4-dioxo-1,4-dihydronaphthalen-2-olate...... S45

$^{1}H^{1}H$, COSY-NMR spectrum of piperidin-1-ium 3-((3-hydroxy-1,4-dioxo-1,4-dihydronaphthalen-2-yl)(phenyl)methyl)-1,4-dioxo-1,4-dihydronaphthalen-2-olate...... S46

$^{1}H^{13}C$, HSQC-NMR spectrum piperidin-1-ium 3-((3-hydroxy-1,4-dioxo-1,4-dihydronaphthalen-2-yl)(phenyl)methyl)-1,4-dioxo-1,4-dihydronaphthalen-2-olate...... S47

$^{1}H^{13}C$, HMBC-NMR spectrum of piperidin-1-ium 3-((3-hydroxy-1,4-dioxo-1,4-dihydronaphthalen-2-yl)(phenyl)methyl)-1,4-dioxo-1,4-dihydronaphthalen-2-olate...... S48

^{1}H NMR spectrum of piperidin-1-ium 3-((3-hydroxy-1,4-dioxo-1,4-dihydronaphthalen-2-yl)(p-tolyl)methyl)-1,4-dioxo-1,4-dihydronaphthalen-2-olate S49

^{13}C NMR (DEPT-135) spectrum of piperidin-1-ium 3-((3-hydroxy-1,4-dioxo-1,4-dihydronaphthalen-2-yl)(p-tolyl)methyl)-1,4-dioxo-1,4-dihydronaphthalen-2-olate...... S50

$^{1}H^{13}C$, HSQC-NMR spectrum piperidin-1-ium 3-((3-hydroxy-1,4-dioxo-1,4-dihydronaphthalen-2-yl)(p-tolyl)methyl)-1,4-dioxo-1,4-dihydronaphthalen-2-olate...... S51

$^{1}H^{1}H$, COSY-NMR spectrum of piperidin-1-ium 3-((3-hydroxy-1,4-dioxo-1,4-dihydronaphthalen-2-yl)(p-tolyl)methyl)-1,4-dioxo-1,4-dihydronaphthalen-2-olate...... S52

$^{1}H^{13}C$, HMBC-NMR spectrum of piperidin-1-ium 3-((3-hydroxy-1,4-dioxo-1,4-dihydronaphthalen-2-yl)(p-tolyl)methyl)-1,4-dioxo-1,4-dihydronaphthalen-2-olate...... S53

FT-IR spectrum of piperidin-1-ium 3-((3-hydroxy-1,4-dioxo-1,4-dihydronaphthalen-2-yl)(4-hydroxyphenyl)methyl)-1,4-dioxo-1,4-dihydronaphthalen-2-olate....................... S54

^{1}H NMR spectrum of piperidin-1-ium 3-((3-hydroxy-1,4-dioxo-1,4-dihydronaphthalen-2-yl)(4-hydroxyphenyl)methyl)-1,4-dioxo-1,4-dihydronaphthalen-2-olate....................... S55

$^{1}H^{1}H$, COSY-NMR spectrum of piperidin-1-ium 3-((3-hydroxy-1,4-dioxo-1,4-dihydronaphthalen-2-yl)(4-hydroxyphenyl)methyl)-1,4-dioxo-1,4-dihydronaphthalen-2-olate.. S56

$^{1}H^{13}C$, HSQC-NMR spectrum piperidin-1-ium 3-((3-hydroxy-1,4-dioxo-1,4-dihydronaphthalen-2-yl)(4-hydroxyphenyl)methyl)-1,4-dioxo-1,4-dihydronaphthalen-2-olate.. S57

$^{1}H^{13}C$, HMBC-NMR spectrum of piperidin-1-ium 3-((3-hydroxy-1,4-dioxo-1,4-dihydronaphthalen-2-yl)(4-hydroxyphenyl)methyl)-1,4-dioxo-1,4-dihydronaphthalen-2-olate.. S58

FT-IR spectrum of piperidin-1-ium 3-((3-hydroxy-1,4-dioxo-1,4-dihydronaphthalen-2-yl)(3-methoxyphenyl)methyl)-1,4-dioxo-1,4-dihydronaphthalen-2-olate....................... S59

^{1}H NMR spectrum of piperidin-1-ium 3-((3-hydroxy-1,4-dioxo-1,4-dihydronaphthalen-2-yl)(3-methoxyphenyl)methyl)-1,4-dioxo-1,4-dihydronaphthalen-2-olate....................... S60

^{13}C NMR (DEPT-135) spectrum of piperidin-1-ium 3-((3-hydroxy-1,4-dioxo-1,4-dihydronaphthalen-2-yl)(3-methoxyphenyl)methyl)-1,4-dioxo-1,4-dihydronaphthalen-2-olate S61

^{1}H^{1}H, COSY-NMR spectrum of piperidin-1-ium 3-((3-hydroxy-1,4-dioxo-1,4-dihydronaphthalen-2-yl)(3-methoxyphenyl)methyl)-1,4-dioxo-1,4-dihydronaphthalen-2-olate S62

^{1}H^{13}C, HSQC-NMR spectrum piperidin-1-ium 3-((3-hydroxy-1,4-dioxo-1,4-dihydronaphthalen-2-yl)(3-methoxyphenyl)methyl)-1,4-dioxo-1,4-dihydronaphthalen-2-olate S63

^{1}H^{13}C, HMBC-NMR spectrum of piperidin-1-ium 3-((3-hydroxy-1,4-dioxo-1,4-dihydronaphthalen-2-yl)(3-methoxyphenyl)methyl)-1,4-dioxo-1,4-dihydronaphthalen-2-olate S64

FT-IR spectrum of piperidin-1-ium 3-((3-bromophenyl)(3-hydroxy-1,4-dioxo-1,4-dihydronaphthalen-2-yl)methyl)-1,4-dioxo-1,4-dihydronaphthalen-2-olate S65

^{1}H NMR spectrum of piperidin-1-ium 3-((3-bromophenyl)(3-hydroxy-1,4-dioxo-1,4-dihydronaphthalen-2-yl)methyl)-1,4-dioxo-1,4-dihydronaphthalen-2-olate S66

^{13}C NMR (DEPT-135) spectrum of piperidin-1-ium 3-((3-bromophenyl)(3-hydroxy-1,4-dioxo-1,4-dihydronaphthalen-2-yl)methyl)-1,4-dioxo-1,4-dihydronaphthalen-2-olate .. S67

^{1}H^{1}H, COSY-NMR spectrum of piperidin-1-ium 3-((3-bromophenyl)(3-hydroxy-1,4-dioxo-1,4-dihydronaphthalen-2-yl)methyl)-1,4-dioxo-1,4-dihydronaphthalen-2-olate .. S68

^{1}H^{13}C, HSQC-NMR spectrum piperidin-1-ium 3-((3-bromophenyl)(3-hydroxy-1,4-dioxo-1,4-dihydronaphthalen-2-yl)methyl)-1,4-dioxo-1,4-dihydronaphthalen-2-olate S69

^{1}H^{13}C, HMBC-NMR spectrum of piperidin-1-ium 3-((3-bromophenyl)(3-hydroxy-1,4-dioxo-1,4-dihydronaphthalen-2-yl)methyl)-1,4-dioxo-1,4-dihydronaphthalen-2-olate .. S70

FT-IR spectrum of piperidin-1-ium 3-((3-hydroxy-1,4-dioxo-1,4-dihydronaphthalen-2-yl)(4-isopropylphenyl)methyl)-1,4-dioxo-1,4-dihydronaphthalen-2-olate S71

^{1}H NMR spectrum of piperidin-1-ium 3-((3-hydroxy-1,4-dioxo-1,4-dihydronaphthalen-2-yl)(4-isopropylphenyl)methyl)-1,4-dioxo-1,4-dihydronaphthalen-2-olate S72

^{1}H^{1}H, COSY-NMR spectrum of piperidin-1-ium 3-((3-hydroxy-1,4-dioxo-1,4-dihydronaphthalen-2-yl)(4-isopropylphenyl)methyl)-1,4-dioxo-1,4-dihydronaphthalen-2-olate S73

^{1}H^{13}C, HSQC-NMR spectrum piperidin-1-ium 3-((3-hydroxy-1,4-dioxo-1,4-dihydronaphthalen-2-yl)(4-isopropylphenyl)methyl)-1,4-dioxo-1,4-dihydronaphthalen-2-olate S74

^{1}H^{13}C, HMBC-NMR spectrum of piperidin-1-ium 3-((3-hydroxy-1,4-dioxo-1,4-dihydronaphthalen-2-yl)(4-isopropylphenyl)methyl)-1,4-dioxo-1,4-dihydronaphthalen-2-olate S75

FT-IR spectrum of piperidin-1-ium 3-((3,4-difluorophenyl)(3-hydroxy-1,4-dioxo-1,4-dihydronaphthalen-2-yl)methyl)-1,4-dioxo-1,4-dihydronaphthalen-2-olate S76

^{1}H NMR spectrum of piperidin-1-ium 3-((3,4-difluorophenyl)(3-hydroxy-1,4-dioxo-1,4-dihydronaphthalen-2-yl)methyl)-1,4-dioxo-1,4-dihydronaphthalen-2-olate S77

^{13}C NMR (DEPT-135) spectrum of piperidin-1-ium 3-((3,4-difluorophenyl)(3-hydroxy-1,4-dioxo-1,4-dihydronaphthalen-2-yl)methyl)-1,4-dioxo-1,4-dihydronaphthalen-2-olate S78

$^{1}H^{1}H$, COSY-NMR spectrum of piperidin-1-ium 3-((3,4-difluorophenyl)(3-hydroxy-1,4-dioxo-1,4-dihydronaphthalen-2-yl)methyl)-1,4-dioxo-1,4-dihydronaphthalen-2-olate .. S79

$^{1}H^{13}C$, HSQC-NMR spectrum piperidin-1-ium 3-((3,4-difluorophenyl)(3-hydroxy-1,4-dioxo-1,4-dihydronaphthalen-2-yl)methyl)-1,4-dioxo-1,4-dihydronaphthalen-2-olate .. S80

$^{1}H^{13}C$, HMBC-NMR spectrum of piperidin-1-ium 3-((3,4-difluorophenyl)(3-hydroxy-1,4-dioxo-1,4-dihydronaphthalen-2-yl)methyl)-1,4-dioxo-1,4-dihydronaphthalen-2-olate .. S81

^{19}F NMR (spin-spin coupling) piperidin-1-ium 3-((3,4-difluorophenyl)(3-hydroxy-1,4-dioxo-1,4-dihydronaphthalen-2-yl)methyl)-1,4-dioxo-1,4-dihydronaphthalen-2-olate .. S82

^{19}F NMR piperidin-1-ium 3-((3,4-difluorophenyl)(3-hydroxy-1,4-dioxo-1,4-dihydronaphthalen-2-yl)methyl)-1,4-dioxo-1,4-dihydronaphthalen-2-olate S83

FT-IR spectrum of piperidin-1-ium 3-((2-bromophenyl)(3-hydroxy-1,4-dioxo-1,4-dihydronaphthalen-2-yl)methyl)-1,4-dioxo-1,4-dihydronaphthalen-2-olate S84

^{1}H NMR spectrum of piperidin-1-ium 3-((2-bromophenyl)(3-hydroxy-1,4-dioxo-1,4-dihydronaphthalen-2-yl)methyl)-1,4-dioxo-1,4-dihydronaphthalen-2-olate S85

^{13}C NMR (DEPT-135) spectrum of piperidin-1-ium 3-((2-bromophenyl)(3-hydroxy-1,4-dioxo-1,4-dihydronaphthalen-2-yl)methyl)-1,4-dioxo-1,4-dihydronaphthalen-2-olate .. S86

$^{1}H^{1}H$, COSY-NMR spectrum of piperidin-1-ium 3-((2-bromophenyl)(3-hydroxy-1,4-dioxo-1,4-dihydronaphthalen-2-yl)methyl)-1,4-dioxo-1,4-dihydronaphthalen-2-olate .. S87

$^{1}H^{13}C$, HSQC-NMR spectrum of -1-ium 3-((2-bromophenyl)(3-hydroxy-1,4-dioxo-1,4-dihydronaphthalen-2-yl)methyl)-1,4-dioxo-1,4-dihydronaphthalen-2-olate S88

$^{1}H^{13}C$, HMBC-NMR spectrum of piperidin-1-ium 3-((2-bromophenyl)(3-hydroxy-1,4-dioxo-1,4-dihydronaphthalen-2-yl)methyl)-1,4-dioxo-1,4-dihydronaphthalen-2-olate .. S89

FT-IR spectrum of piperidin-1-ium 3-((3-hydroxy-1,4-dioxo-1,4-dihydronaphthalen-2-yl)(naphthalen-2-yl)methyl)-1,4-dioxo-1,4-dihydronaphthalen-2-olate S90

^{1}H NMR spectrum of piperidin-1-ium 3-((3-hydroxy-1,4-dioxo-1,4-dihydronaphthalen-2-yl)(naphthalen-2-yl)methyl)-1,4-dioxo-1,4-dihydronaphthalen-2-olate S91

^{13}C NMR (DEPT-135) spectrum of piperidin-1-ium 3-((3-hydroxy-1,4-dioxo-1,4-dihydronaphthalen-2-yl)(naphthalen-2-yl)methyl)-1,4-dioxo-1,4-dihydronaphthalen-2-olate S92

$^{1}H^{1}H$, COSY-NMR spectrum of piperidin-1-ium 3-((3-hydroxy-1,4-dioxo-1,4-dihydronaphthalen-2-yl)(naphthalen-2-yl)methyl)-1,4-dioxo-1,4-dihydronaphthalen-2-olate S93

$^{1}H^{13}C$, HSQC-NMR spectrum of piperidin-1-ium 3-((3-hydroxy-1,4-dioxo-1,4-dihydronaphthalen-2-yl)(naphthalen-2-yl)methyl)-1,4-dioxo-1,4-dihydronaphthalen-2-olate S94

$^{1}H^{13}C$, HMBC-NMR spectrum of piperidin-1-ium 3-((3-hydroxy-1,4-dioxo-1,4-dihydronaphthalen-2-yl)(naphthalen-2-yl)methyl)-1,4-dioxo-1,4-dihydronaphthalen-2-olate S95

^{1}H NMR spectrum of piperidin-1-ium 3-((3-hydroxy-1,4-dioxo-1,4-dihydronaphthalen-2-yl)(thiophen-2-yl)methyl)-1,4-dioxo-1,4-dihydronaphthalen-2-olate S96

^{13}C NMR (DEPT-135) spectrum of piperidin-1-ium 3-((3-hydroxy-1,4-dioxo-1,4-dihydronaphthalen-2-yl)(thiophen-2-yl)methyl)-1,4-dioxo-1,4-dihydronaphthalen-2-olate S97

^{1}H^{1}H, COSY-NMR spectrum of piperidin-1-ium 3-((3-hydroxy-1,4-dioxo-1,4-dihydronaphthalen-2-yl)(thiophen-2-yl)methyl)-1,4-dioxo-1,4-dihydronaphthalen-2-olate S98

^{1}H^{13}C, HSQC-NMR spectrum of piperidin-1-ium 3-((3-hydroxy-1,4-dioxo-1,4-dihydronaphthalen-2-yl)(thiophen-2-yl)methyl)-1,4-dioxo-1,4-dihydronaphthalen-2-olate S99

^{1}H^{13}C, HMBC-NMR spectrum of piperidin-1-ium 3-((3-hydroxy-1,4-dioxo-1,4-dihydronaphthalen-2-yl)(thiophen-2-yl)methyl)-1,4-dioxo-1,4-dihydronaphthalen-2-olate S100

FT-IR spectrum of piperazine-1,4-diium 3-((2-chlorophenyl)(3-hydroxy-1,4-dioxo-1,4-dihydronaphthalen-2-yl)methyl)-1,4-dioxo-1,4-dihydronaphthalen-2-olate S101

^{1}H NMR spectrum of piperazine-1,4-diium 3-((2-chlorophenyl)(3-hydroxy-1,4-dioxo-1,4-dihydronaphthalen-2-yl)methyl)-1,4-dioxo-1,4-dihydronaphthalen-2-olate S102

^{13}C NMR spectrum of piperazine-1,4-diium 3-((2-chlorophenyl)(3-hydroxy-1,4-dioxo-1,4-dihydronaphthalen-2-yl)methyl)-1,4-dioxo-1,4-dihydronaphthalen-2-olate S103

^{1}H^{13}C, HSQC-NMR spectrum of piperazine-1,4-diium 3-((2-chlorophenyl)(3-hydroxy-1,4-dioxo-1,4-dihydronaphthalen-2-yl)methyl)-1,4-dioxo-1,4-dihydronaphthalen-2-olate S104

HR-Mass spectrum of piperazine-1,4-diium 3-((2-chlorophenyl)(3-hydroxy-1,4-dioxo-1,4-dihydronaphthalen-2-yl)methyl)-1,4-dioxo-1,4-dihydronaphthalen-2-olate S105

FT-IR spectrum of piperazine-1,4-diium 3-((3,4-difluorophenyl)(1,4-dioxo-1,4-dihydronaphthalen-2-yl)methyl)-1,4-dioxo-1,4-dihydronaphthalen-2-olate S106

^{1}H NMR spectrum of piperazine-1,4-diium 3-((3,4-difluorophenyl)(1,4-dioxo-1,4-dihydronaphthalen-2-yl)methyl)-1,4-dioxo-1,4-dihydronaphthalen-2-olate S107

^{13}C NMR spectrum of piperazine-1,4-diium 3-((3,4-difluorophenyl)(1,4-dioxo-1,4-dihydronaphthalen-2-yl)methyl)-1,4-dioxo-1,4-dihydronaphthalen-2-olate S108

^{1}H^{13}C, HSQC-NMR spectrum of piperazine-1,4-diium 3-((3,4-difluorophenyl)(1,4-dioxo-1,4-dihydronaphthalen-2-yl)methyl)-1,4-dioxo-1,4-dihydronaphthalen-2-olate S109

HR-Mass spectrum of piperazine-1,4-diium 3-((3,4-difluorophenyl)(1,4-dioxo-1,4-dihydronaphthalen-2-yl)methyl)-1,4-dioxo-1,4-dihydronaphthalen-2-olate S110

FT-IR spectrum of piperazine-1,4-diium 3-((1,4-dioxo-1,4-dihydronaphthalen-2-yl)(3-hydroxyphenyl)methyl)-1,4-dioxo-1,4-dihydronaphthalen-2-olate S111

^{1}H NMR spectrum of piperazine-1,4-diium 3-((1,4-dioxo-1,4-dihydronaphthalen-2-yl)(3-hydroxyphenyl)methyl)-1,4-dioxo-1,4-dihydronaphthalen-2-olate S112

^{13}C NMR spectrum of piperazine-1,4-diium 3-((1,4-dioxo-1,4-dihydronaphthalen-2-yl)(3-hydroxyphenyl)methyl)-1,4-dioxo-1,4-dihydronaphthalen-2-olate S113

$^{1}H^{13}C$, HSQC-NMR spectrum of piperazine-1,4-diium 3-((1,4-dioxo-1,4-dihydronaphthalen-2-yl)(3-hydroxyphenyl)methyl)-1,4-dioxo-1,4-dihydronaphthalen-2-olate S114

HR-Mass spectrum of piperazine-1,4-diium 3-((1,4-dioxo-1,4-dihydronaphthalen-2-yl)(3-hydroxyphenyl)methyl)-1,4-dioxo-1,4-dihydronaphthalen-2-olate S115

FT-IR spectrum ofpiperazine-1,4-diium 3-((1,4-dioxo-1,4-dihydronaphthalen-2-yl)(4-hydroxyphenyl)methyl)-1,4-dioxo-1,4-dihydronaphthalen-2-olate S116

^{1}HNMR spectrum of piperazine-1,4-diium 3-((1,4-dioxo-1,4-dihydronaphthalen-2-yl)(4-hydroxyphenyl)methyl)-1,4-dioxo-1,4-dihydronaphthalen-2-olate S117

^{13}C NMR spectrum of piperazine-1,4-diium 3-((1,4-dioxo-1,4-dihydronaphthalen-2-yl)(4-hydroxyphenyl)methyl)-1,4-dioxo-1,4-dihydronaphthalen-2-olate S118

$^{1}H^{13}C$, HSQC-NMR spectrum of piperazine-1,4-diium 3-((1,4-dioxo-1,4-dihydronaphthalen-2-yl)(4-hydroxyphenyl)methyl)-1,4-dioxo-1,4-dihydronaphthalen-2-olate S119

HR-Mass spectrum of piperazine-1,4-diium 3-((1,4-dioxo-1,4-dihydronaphthalen-2-yl)(4-hydroxyphenyl)methyl)-1,4-dioxo-1,4-dihydronaphthalen-2-olate S120

FT-IR spectrum of piperazine-1,4-diium 3-((4-chlorophenyl)(1,4-dioxo-1,4-dihydronaphthalen-2-yl)methyl)-1,4-dioxo-1,4-dihydronaphthalen-2-olate S121

^{1}H NMR spectrum of piperazine-1,4-diium 3-((4-chlorophenyl)(1,4-dioxo-1,4-dihydronaphthalen-2-yl)methyl)-1,4-dioxo-1,4-dihydronaphthalen-2-olate S122

^{13}C NMR spectrum of piperazine-1,4-diium 3-((4-chlorophenyl)(1,4-dioxo-1,4-dihydronaphthalen-2-yl)methyl)-1,4-dioxo-1,4-dihydronaphthalen-2-olate S123

$^{1}H^{13}C$, HSQC-NMR spectrum of piperazine-1,4-diium 3-((4-chlorophenyl)(1,4-dioxo-1,4-dihydronaphthalen-2-yl)methyl)-1,4-dioxo-1,4-dihydronaphthalen-2-olate S124

HR-Mass spectrum of piperazine-1,4-diium 3-((4-chlorophenyl)(1,4-dioxo-1,4-dihydronaphthalen-2-yl)methyl)-1,4-dioxo-1,4-dihydronaphthalen-2-olate S125

FT-IR spectrum of piperazine-1,4-diium 3-((1,4-dioxo-1,4-dihydronaphthalen-2-yl)(3-nitrophenyl)methyl)-1,4-dioxo-1,4-dihydronaphthalen-2-olate S126

^{1}H NMR spectrum of piperazine-1,4-diium 3-((1,4-dioxo-1,4-dihydronaphthalen-2-yl)(3-nitrophenyl)methyl)-1,4-dioxo-1,4-dihydronaphthalen-2-olate S127

^{13}C NMR spectrum of piperazine-1,4-diium 3-((1,4-dioxo-1,4-dihydronaphthalen-2-yl)(3-nitrophenyl)methyl)-1,4-dioxo-1,4-dihydronaphthalen-2-olate S128

$^{1}H^{13}C$, HSQC-NMR spectrum of piperazine-1,4-diium 3-((1,4-dioxo-1,4-dihydronaphthalen-2-yl)(3-nitrophenyl)methyl)-1,4-dioxo-1,4-dihydronaphthalen-2-olate S129

HR-Mass spectrum of piperazine-1,4-diium 3-((1,4-dioxo-1,4-dihydronaphthalen-2-yl)(3-nitrophenyl)methyl)-1,4-dioxo-1,4-dihydronaphthalen-2-olate S130

FT-IR spectrum of piperazine-1,4-diium 3-((1,4-dioxo-1,4-dihydronaphthalen-2-yl)(4-(trifluoromethyl)phenyl)methyl)-1,4-dioxo-1,4-dihydronaphthalen-2-olate S131

^{1}H NMR spectrum of piperazine-1,4-diium 3-((1,4-dioxo-1,4-dihydronaphthalen-2-yl)(4-(trifluoromethyl)phenyl)methyl)-1,4-dioxo-1,4-dihydronaphthalen-2-olate S132

^{13}C NMR spectrum of piperazine-1,4-diium 3-((1,4-dioxo-1,4-dihydronaphthalen-2-yl)(4-(trifluoromethyl)phenyl)methyl)-1,4-dioxo-1,4-dihydronaphthalen-2-olate S133

$^{1}H^{13}C$, HSQC-NMR spectrum of piperazine-1,4-diium 3-((1,4-dioxo-1,4-dihydronaphthalen-2-yl)(4-(trifluoromethyl)phenyl)methyl)-1,4-dioxo-1,4-dihydronaphthalen-2-olate S134

HR-Mass spectrum of piperazine-1,4-diium 3-((1,4-dioxo-1,4-dihydronaphthalen-2-yl)(4-(trifluoromethyl)phenyl)methyl)-1,4-dioxo-1,4-dihydronaphthalen-2-olate S135

FT-IR spectrum ofpiperazine-1,4-diium 3-((1,4-dioxo-1,4-dihydronaphthalen-2-yl)(thiophen-2-yl)methyl)-1,4-dioxo-1,4-dihydronaphthalen-2-olate S136

^{1}H NMR spectrum of piperazine-1,4-diium 3-((1,4-dioxo-1,4-dihydronaphthalen-2-yl)(thiophen-2-yl)methyl)-1,4-dioxo-1,4-dihydronaphthalen-2-olate S137

^{13}C NMR spectrum of piperazine-1,4-diium 3-((1,4-dioxo-1,4-dihydronaphthalen-2-yl)(thiophen-2-yl)methyl)-1,4-dioxo-1,4-dihydronaphthalen-2-olate S138

$^{1}H^{13}C$, HSQC-NMR spectrum of piperazine-1,4-diium 3-((1,4-dioxo-1,4-dihydronaphthalen-2-yl)(thiophen-2-yl)methyl)-1,4-dioxo-1,4-dihydronaphthalen-2-olate S139

HR-Mass spectrum of piperazine-1,4-diium 3-((1,4-dioxo-1,4-dihydronaphthalen-2-yl)(thiophen-2-yl)methyl)-1,4-dioxo-1,4-dihydronaphthalen-2-olate S140

FT-IR spectrum of piperazine-1,4-diium 3-((1,4-dioxo-1,4-dihydronaphthalen-2-yl)(pyridin-3-yl)methyl)-1,4-dioxo-1,4-dihydronaphthalen-2-olate S141

^{1}H NMR spectrum of piperazine-1,4-diium 3-((1,4-dioxo-1,4-dihydronaphthalen-2-yl)(pyridin-3-yl)methyl)-1,4-dioxo-1,4-dihydronaphthalen-2-olate S142

^{13}C NMR spectrum of piperazine-1,4-diium 3-((1,4-dioxo-1,4-dihydronaphthalen-2-yl)(pyridin-3-yl)methyl)-1,4-dioxo-1,4-dihydronaphthalen-2-olate S143

$^{1}H^{13}C$, HSQC-NMR spectrum of piperazine-1,4-diium 3-((1,4-dioxo-1,4-dihydronaphthalen-2-yl)(pyridin-3-yl)methyl)-1,4-dioxo-1,4-dihydronaphthalen-2-olate S144

HR-Mass spectrum of piperazine-1,4-diium 3-((1,4-dioxo-1,4-dihydronaphthalen-2-yl)(pyridin-3-yl)methyl)-1,4-dioxo-1,4-dihydronaphthalen-2-olate S145

FT-IR spectrum of piperazine-1,4-diium 3-((3-hydroxy-1,4-dioxo-1,4-dihydronaphthalen-2-yl)(p-tolyl)methyl)-1,4-dioxo-1,4-dihydronaphthalen-2-olate.... S146

^{1}H NMR spectrum of piperazine-1,4-diium 3-((3-hydroxy-1,4-dioxo-1,4-dihydronaphthalen-2-yl)(p-tolyl)methyl)-1,4-dioxo-1,4-dihydronaphthalen-2-olate.... S147

^{13}CNMR spectrum of piperazine-1,4-diium 3-((3-hydroxy-1,4-dioxo-1,4-dihydronaphthalen-2-yl)(p-tolyl)methyl)-1,4-dioxo-1,4-dihydronaphthalen-2-olate.... S148

$^{1}H^{13}C$, HSQC-NMR piperazine-1,4-diium 3-((3-hydroxy-1,4-dioxo-1,4-dihydronaphthalen-2-yl)(p-tolyl)methyl)-1,4-dioxo-1,4-dihydronaphthalen-2-olate.... S149

HR-Mass spectrum of piperazine-1,4-diium 3-((3-hydroxy-1,4-dioxo-1,4-dihydronaphthalen-2-yl)(p-tolyl)methyl)-1,4-dioxo-1,4-dihydronaphthalen-2-olate.... S150

FT-IR spectrum of piperazine-1,4-diium 3-((3-hydroxy-1,4-dioxo-1,4-dihydronaphthalen-2-yl)(phenyl)methyl)-1,4-dioxo-1,4-dihydronaphthalen-2-olate S151

^{1}H NMR spectrum of piperazine-1,4-diium 3-((3-hydroxy-1,4-dioxo-1,4-dihydronaphthalen-2-yl)(phenyl)methyl)-1,4-dioxo-1,4-dihydronaphthalen-2-olate.... S152

^{13}C NMR spectrum of piperazine-1,4-diium 3-((3-hydroxy-1,4-dioxo-1,4-dihydronaphthalen-2-yl)(phenyl)methyl)-1,4-dioxo-1,4-dihydronaphthalen-2-olate.... S153

$^{1}H^{13}C$, HSQC-NMR spectrum of piperazine-1,4-diium 3-((3-hydroxy-1,4-dioxo-1,4-dihydronaphthalen-2-yl)(phenyl)methyl)-1,4-dioxo-1,4-dihydronaphthalen-2-olate.... S154

HR-Mass spectrum of piperazine-1,4-diium 3-((3-hydroxy-1,4-dioxo-1,4-dihydronaphthalen-2-yl)(phenyl)methyl)-1,4-dioxo-1,4-dihydronaphthalen-2-olate.... S155

FT-IR spectrum of piperazine-1,4-diium 3-((3,4-dimethoxyphenyl)(3-hydroxy-1,4-dioxo-1,4-dihydronaphthalen-2-yl)methyl)-1,4-dioxo-1,4-dihydronaphthalen-2-olate........... S156

^{1}H NMR spectrum of piperazine-1,4-diium 3-((3,4-dimethoxyphenyl)(3-hydroxy-1,4-dioxo-1,4-dihydronaphthalen-2-yl)methyl)-1,4-dioxo-1,4-dihydronaphthalen-2-olate.. .... S157

^{13}C NMR spectrum of piperazine-1,4-diium 3-((3,4-dimethoxyphenyl)(3-hydroxy-1,4-dioxo-1,4-dihydronaphthalen-2-yl)methyl)-1,4-dioxo-1,4-dihydronaphthalen-2-olate S158

$^{1}H^{13}C$, HSQC-NMR spectrum of piperazine-1,4-diium 3-((3,4-dimethoxyphenyl)(3-hydroxy-1,4-dioxo-1,4-dihydronaphthalen-2-yl)methyl)-1,4-dioxo-1,4-dihydronaphthalen-2-olate S159

FT-IR spectrum of piperazine-1,4-diium 3-((3-hydroxy-1,4-dioxo-1,4-dihydronaphthalen-2-yl)(4-nitrophenyl)methyl)-1,4-dioxo-1,4-dihydronaphthalen-2-olate S160

^{1}H NMR spectrum of piperazine-1,4-diium 3-((3-hydroxy-1,4-dioxo-1,4-dihydronaphthalen-2-yl)(4-nitrophenyl)methyl)-1,4-dioxo-1,4-dihydronaphthalen-2-olate S161

^{13}C NMR spectrum of piperazine-1,4-diium 3-((3-hydroxy-1,4-dioxo-1,4-dihydronaphthalen-2-yl)(4-nitrophenyl)methyl)-1,4-dioxo-1,4-dihydronaphthalen-2-olate S162

$^{1}H^{13}C$, HSQC-NMR spectrum of piperazine-1,4-diium 3-((3-hydroxy-1,4-dioxo-1,4-dihydronaphthalen-2-yl)(4-nitrophenyl)methyl)-1,4-dioxo-1,4-dihydronaphthalen-2-olate S163

HR-Mass spectrum of piperazine-1,4-diium 3-((3-hydroxy-1,4-dioxo-1,4-dihydronaphthalen-2-yl)(4-nitrophenyl)methyl)-1,4-dioxo-1,4-dihydronaphthalen-2-olate S164

FT-IR spectrum of piperazine-1,4-diium 3-((3-hydroxy-1,4-dioxo-1,4-dihydronaphthalen-2-yl)(4-isopropylphenyl)methyl)-1,4-dioxo-1,4-dihydronaphthalen-2-olate S165

^{1}H NMR spectrum of piperazine-1,4-diium 3-((3-hydroxy-1,4-dioxo-1,4-dihydronaphthalen-2-yl)(4-isopropylphenyl)methyl)-1,4-dioxo-1,4-dihydronaphthalen-2-olate S166

^{13}C NMR spectrum of piperazine-1,4-diium 3-((3-hydroxy-1,4-dioxo-1,4-dihydronaphthalen-2-yl)(4-isopropylphenyl)methyl)-1,4-dioxo-1,4-dihydronaphthalen-2-olate S167

$^{1}H^{13}C$, HSQC-NMR spectrum of piperazine-1,4-diium 3-((3-hydroxy-1,4-dioxo-1,4-dihydronaphthalen-2-yl)(4-isopropylphenyl)methyl)-1,4-dioxo-1,4-dihydronaphthalen-2-olate S168

HR-Mass spectrum of piperazine-1,4-diium 3-((3-hydroxy-1,4-dioxo-1,4-dihydronaphthalen-2-yl)(4-isopropylphenyl)methyl)-1,4-dioxo-1,4-dihydronaphthalen-2-olate S169

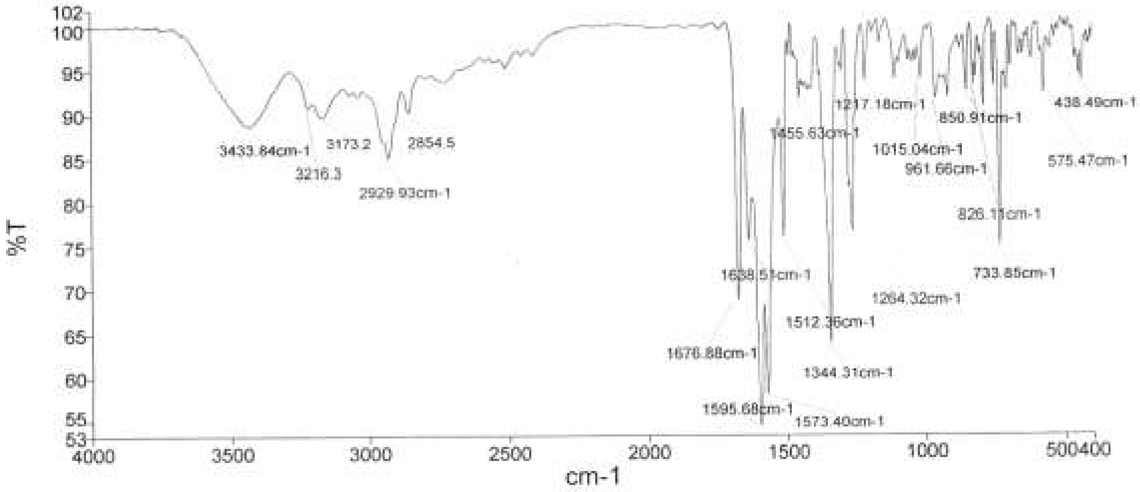

Figure S1: FT-IR spectrum of compound 5a

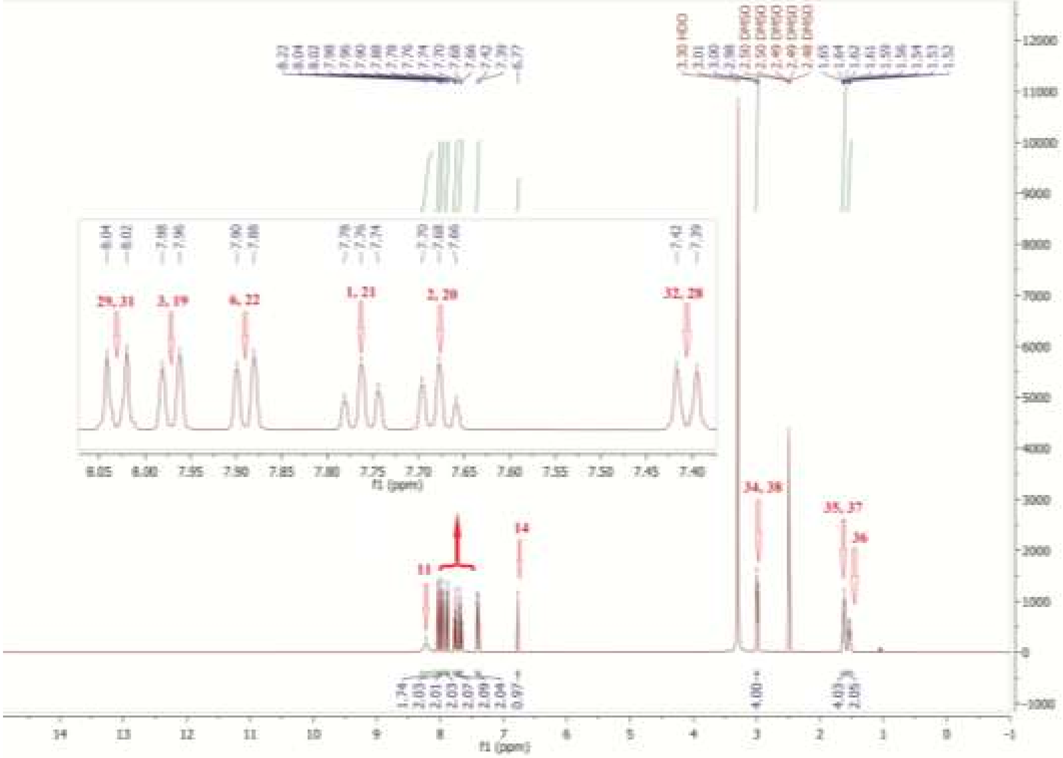

Figure S2: ^{1}H NMR spectrum of compound 5a

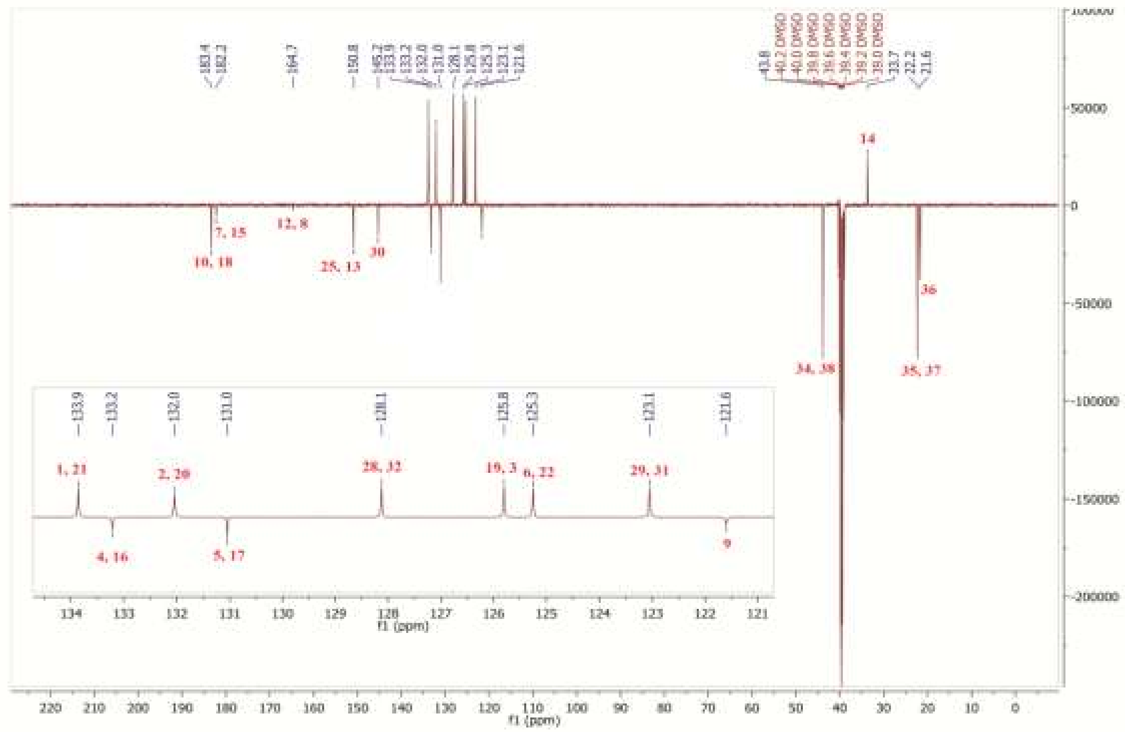

Figure S3: ^{13}C NMR (DEPT-135) spectrum of compound 5a

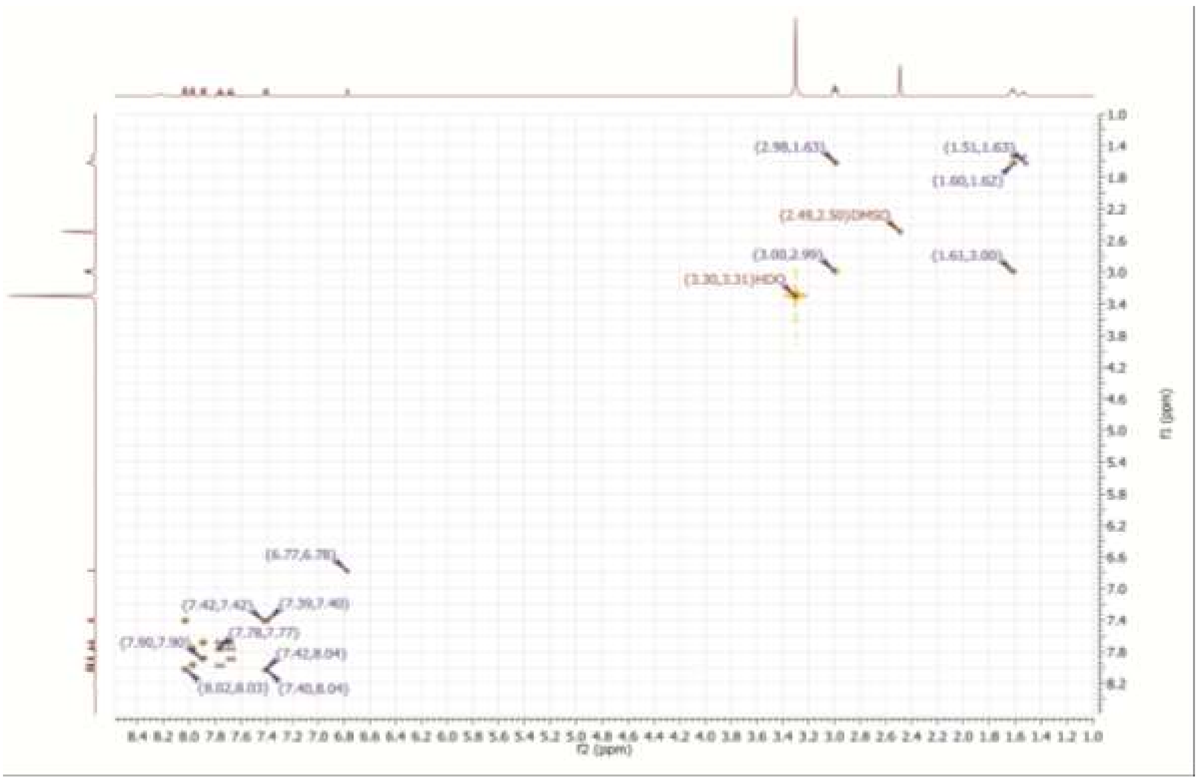

Figure S4: ^{1}H^{1}H, COSY-NMR spectrum of compound 5a

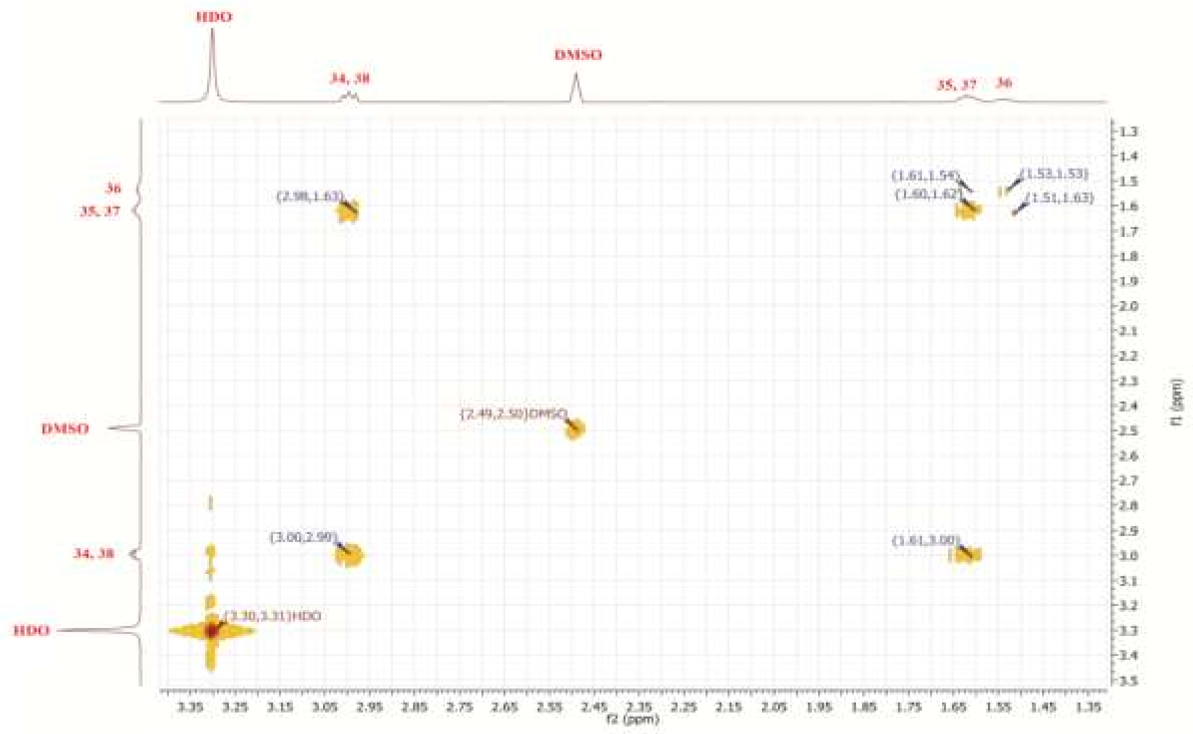

Figure S5: ^{1}H^{1}H, COSY-NMR expand spectrum of compound 5a

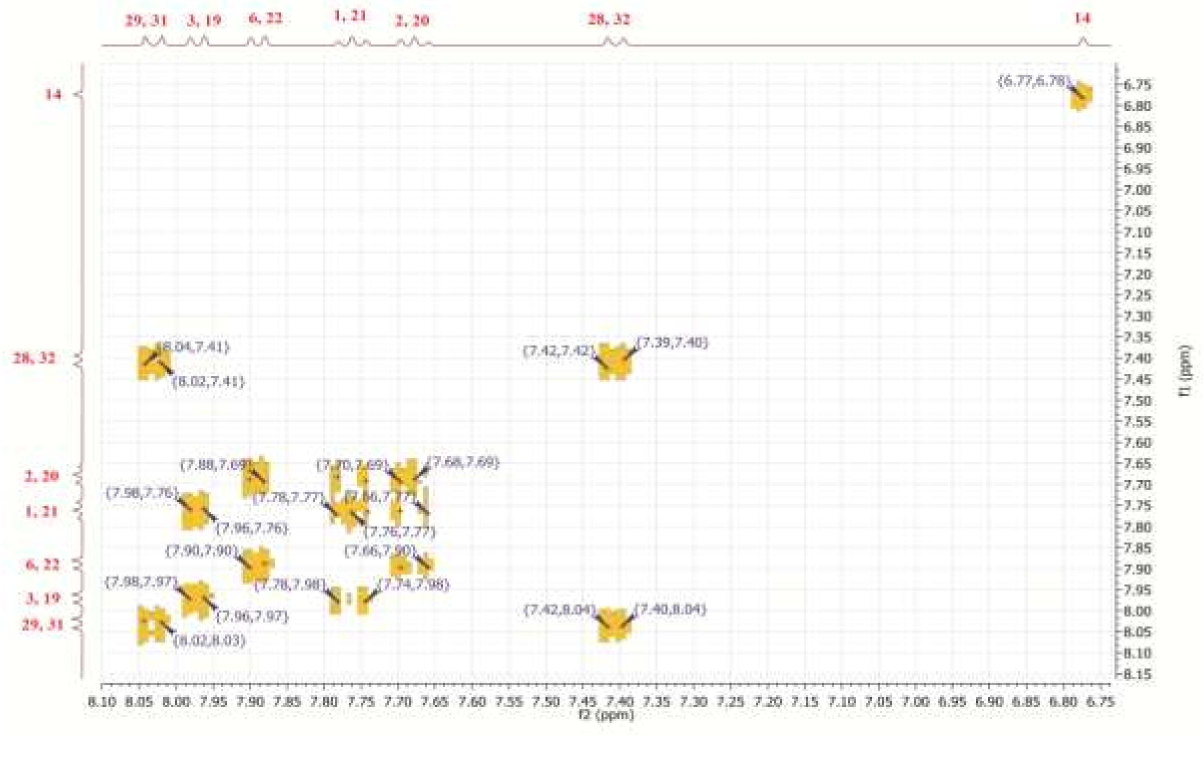

Figure S6: ^{1}H^{1}H, COSY-NMR expand spectrum of compound 5a

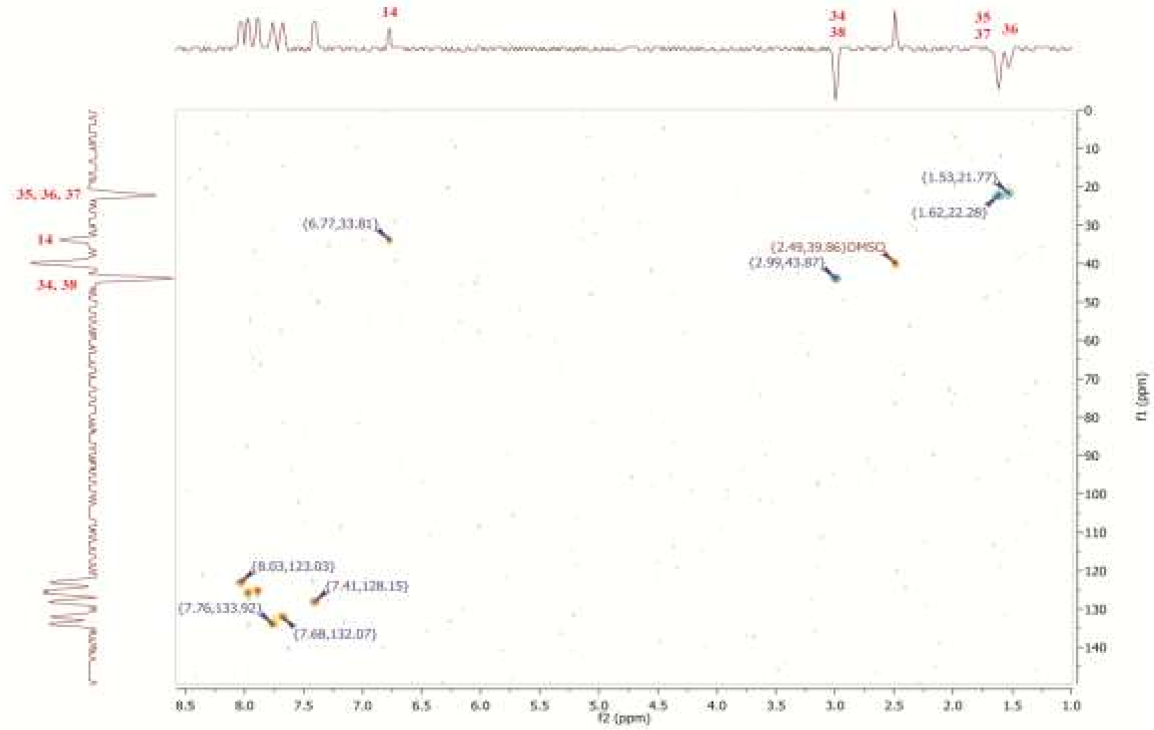

Figure S7: $^{1}H^{13}C$ HSQC-NMR spectrum of compound 5a

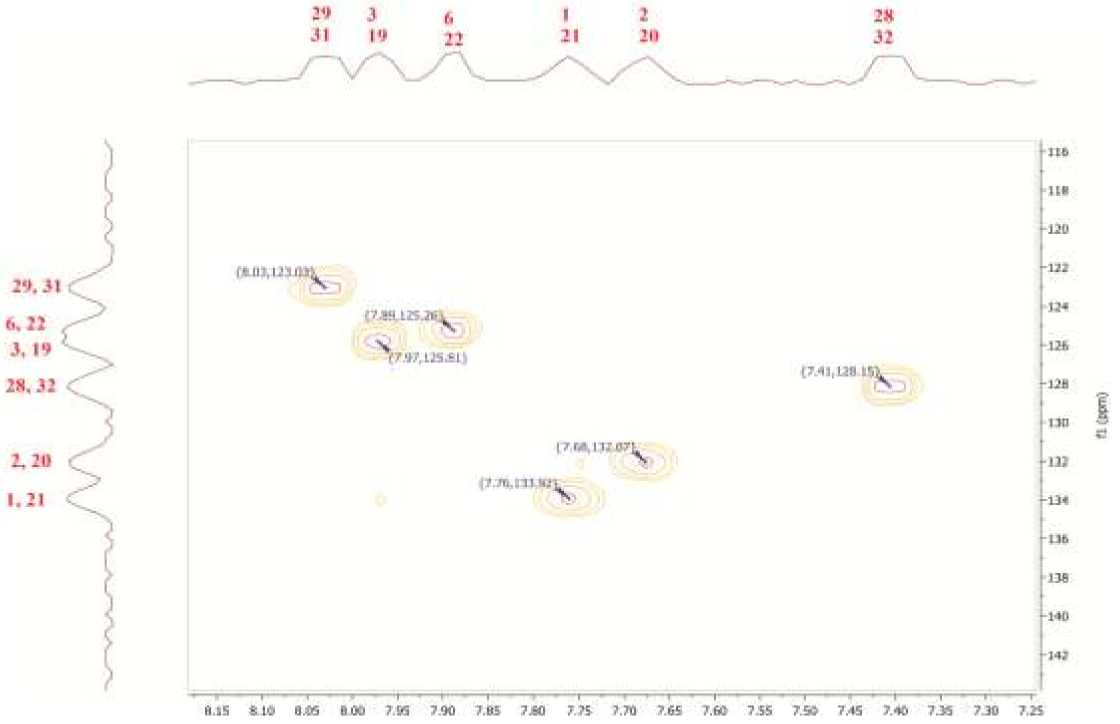

Figure S8: $^{1}H^{13}C$, HSQC-NMR expand spectrum of compound 5a

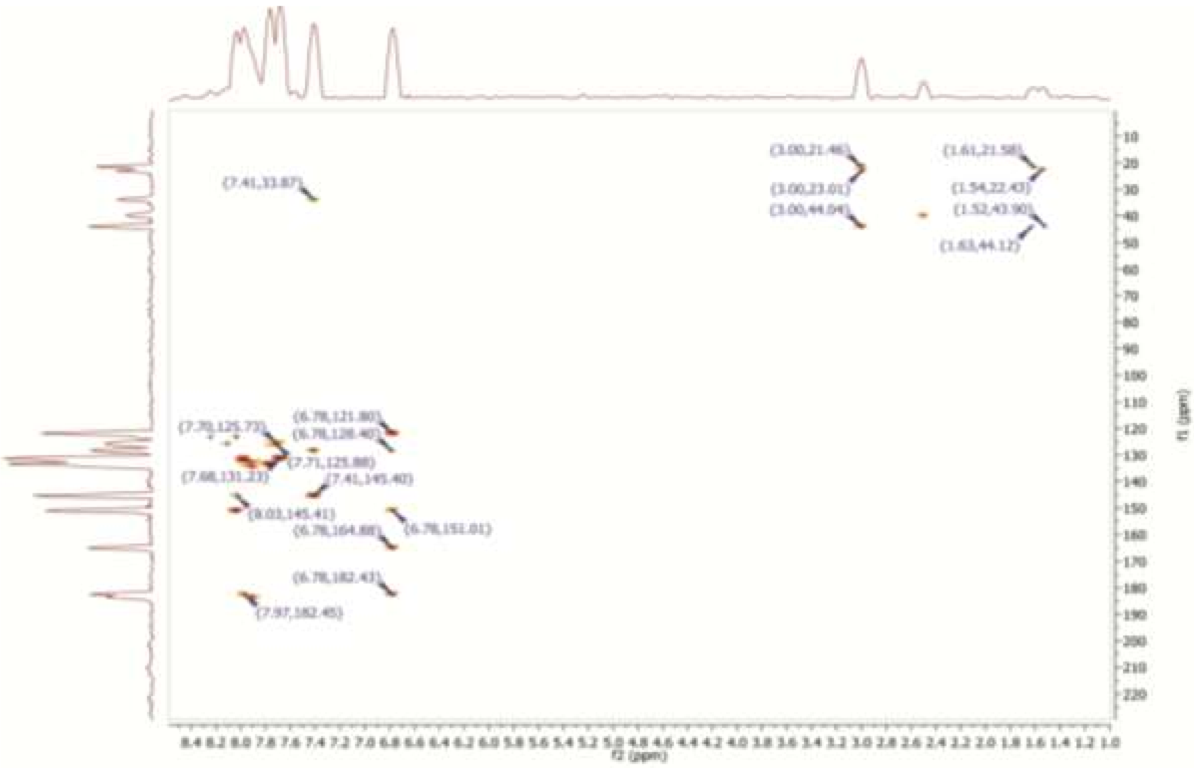

Figure S9: $^{1}H^{13}C$ HMBC-NMR spectrum of compound 5a

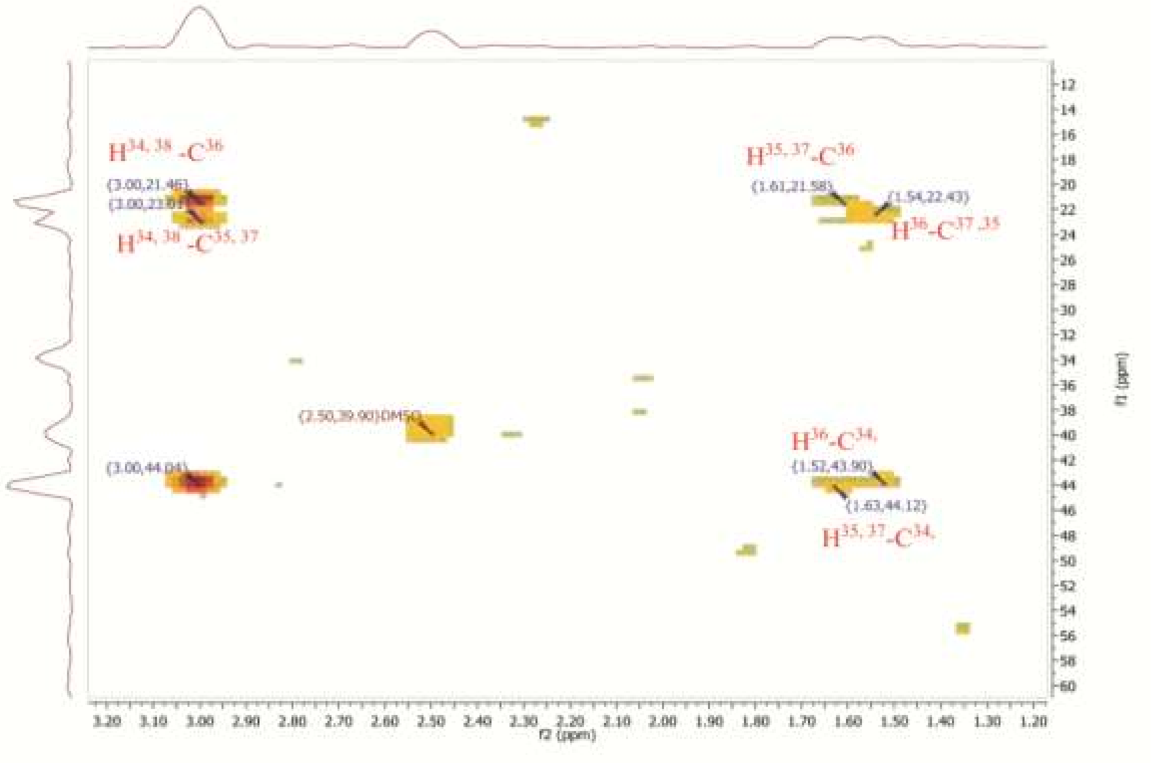

Figure S10: $^{1}H^{13}C$ HMBC-NMR expand spectrum of compound 5a

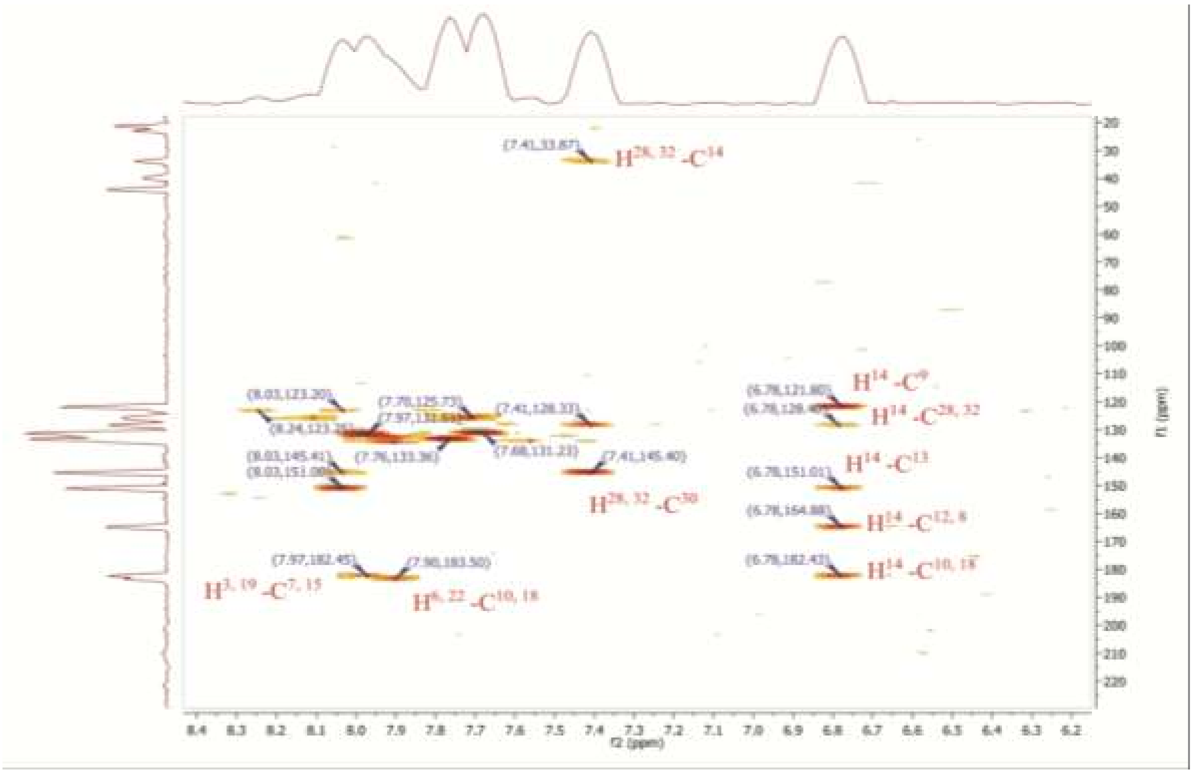

Figure S11: $^{1}H^{13}C$ HMBC-NMR expand spectrum of compound 5a

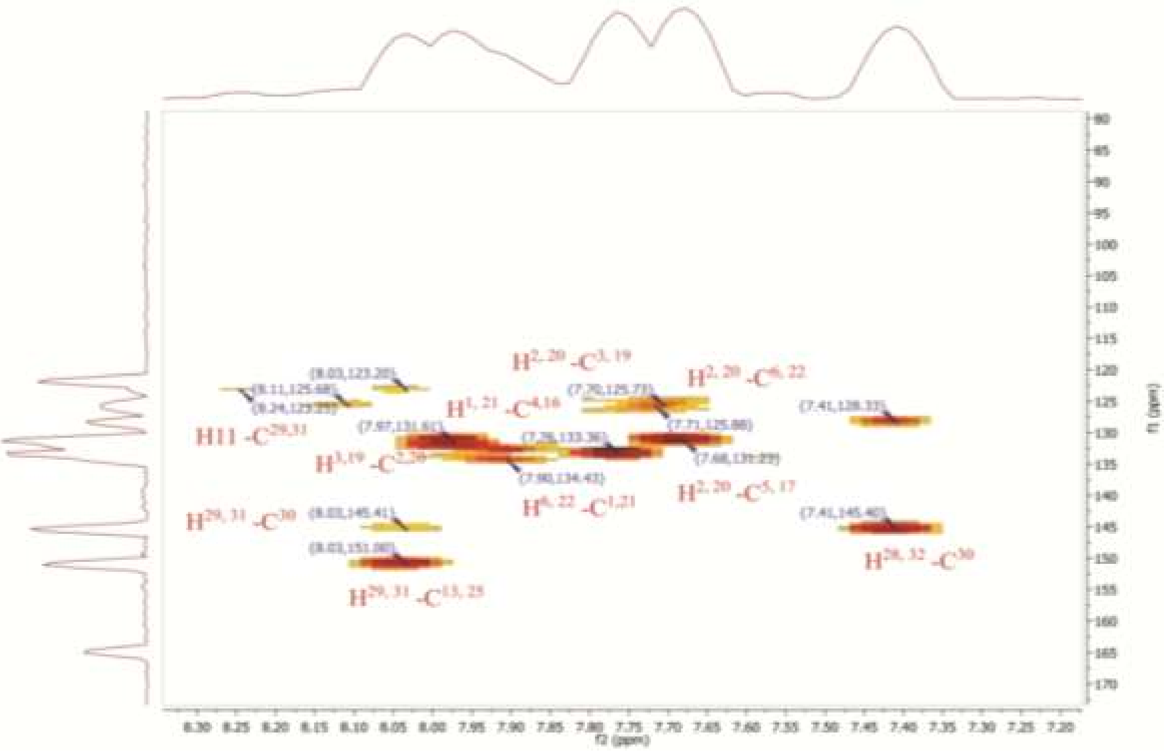

Figure S12: $^{1}H^{13}C$ HMBC-NMR expand spectrum of compound 5a

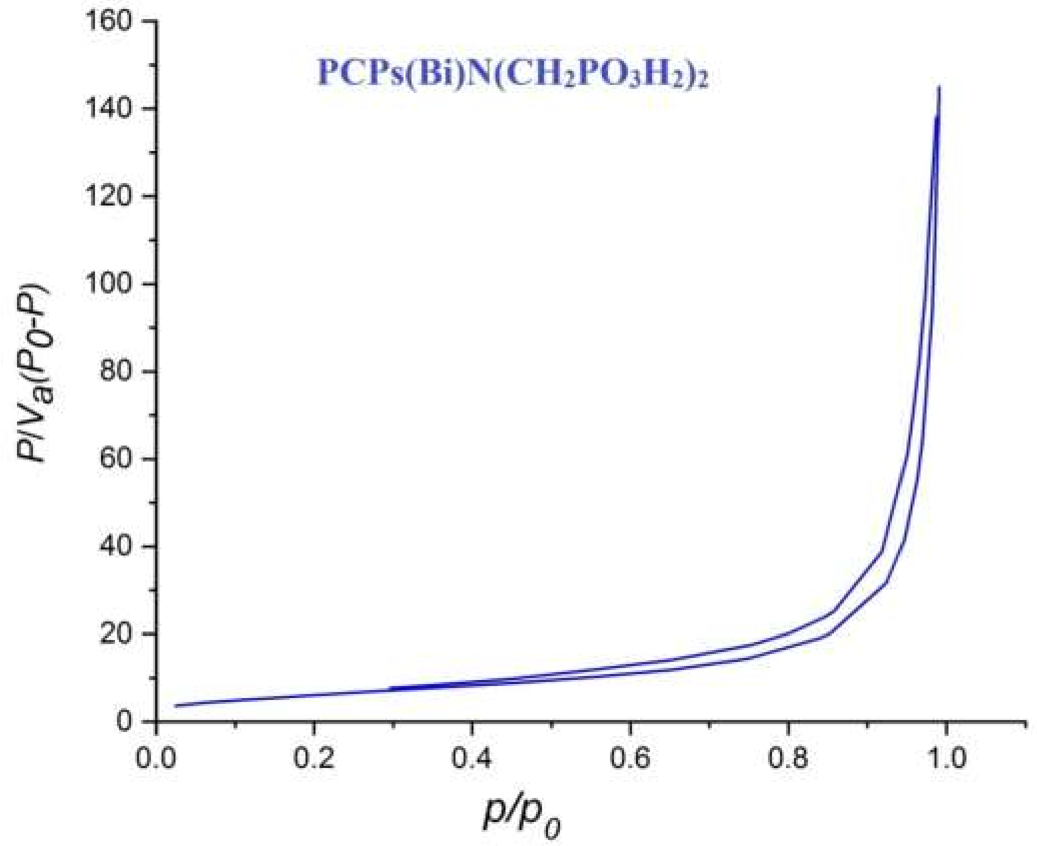

Figure S13: Nitrogen adsorption-desorption isotherm of PCPs(Bi)N($CH_2PO_3H_2$)$_2$

Suggested mechanism for the synthesis of napthoquinone derivatives using PCPs(Bi)N($CH_2PO_3H_2$)$_2$

A suggested mechanism for the synthesis of napthoquinone derivatives as salts of piperazine or piperidine **5a-5j** and **6a-6n** using PCPs(Bi)N($CH_2PO_3H_2$)$_2$ has been summarized in scheme 7. Initially, the aldehyde is activated by the acidic sites of the catalyst, in which 2-hydroxynaphthalene-1,4-dione reacts with the aldehyde, to afford intermediate (**I**). Then, intermediate (**I**) reacts with another 2-hydroxynaphthalene-1,4-dione by removing one water molecule and to afford intermediate (**II**). Intermediate (**II**), is converted to intermediate (**III**) via losing its proton. Piperazine receives (H^+) from the catalyst and becomes a piperazinium cation at the intermediate **(IV)**. Two intermediates **(III)** and (**IV)** come together. Finally, by repeating this process, the napthoquinone derivatives of piperazine are synthesized (Scheme S1).

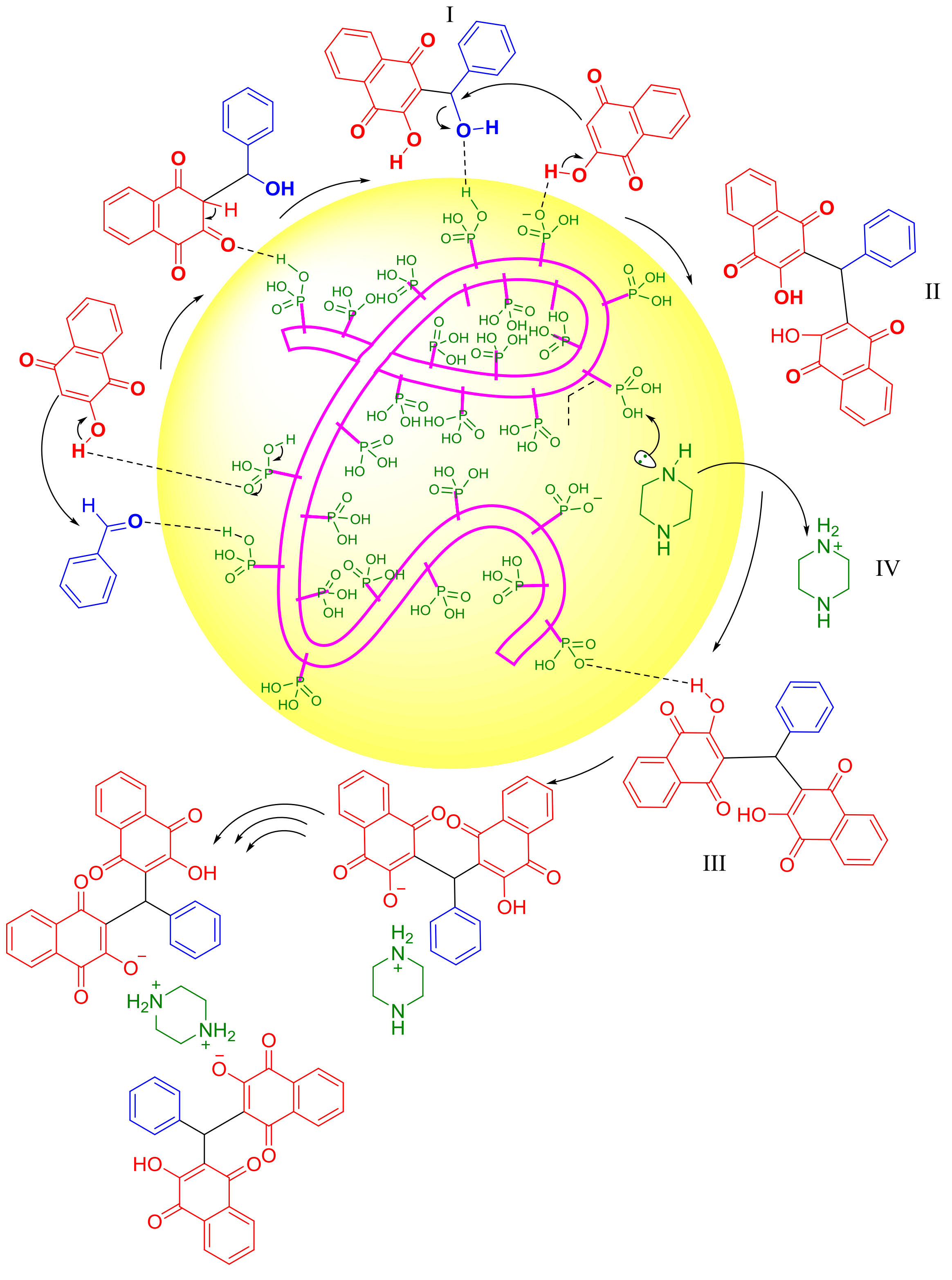

Scheme S1: Proposed mechanism for the synthesis napthoquinone derivatives using $PCPs(Bi)N(CH_2PO_3H_2)_2$

Piperidin-1-ium 3-((3-hydroxy-1,4-dioxo-1,4-dihydronaphthalen-2-yl)(4-nitrophenyl)methyl)-1,4-dioxo-1,4-dihydronaphthalen-2-olate

Orange solid; Mp: 249-250 °C; IR (KBr): υ (cm^{-1}) = 3433, 3216, 3173, 2929,2854, 1676, 1638, 1595, 1573, 1512, 1344. ^{1}H NMR (400 MHz, DMSO-*d6*) δ 8.22 (s, 2H), 8.03 (d, *J* = 8.8 Hz, 2H), 7.97 (d, *J* = 7.6 Hz, 2H), 7.89 (d, *J* = 7.4 Hz, 2H), 7.76 (t, *J* = 7.4 Hz, 2H), 7.68 (t, *J* = 7.4 Hz, 2H), 7.41 (d, *J* = 8.6 Hz, 2H), 6.77 (s, 1H), 3.02 – 2.97 (m, 4H), 1.62 (p, *J* = 5.7 Hz, 4H), 1.54 (q, *J* = 5.3 Hz, 2H). ^{13}C NMR (DEPT-135) (100 MHz, DMSO-*d6*) δ 183.4, 182.2, 164.7, 150.8, 145.2, 133.9, 133.2, 132.0, 131.0, 128.1, 125.8, 125.3, 123.1, 121.6, 43.8, 33.7, 22.2, 21.6. $^{1}H^{1}H$ COSY-NMR ((400, 400) MHz, DMSO-*d6*) δ (8.04 8.02), (8.04 7.41), (8.02 7.41), (8.02 8.03), (7.98 7.97), (7.98 7.76), (7.96 7.76), (7.96 7.97), (7.90 7.90), (7.90 7.69), (7.88 7.69), (7.88 7.89), (7.78 7.77), (7.78 7.68), (7.78 7.98), (7.76 7.77), (7.74 7.98), (7.74 7.69), (7.74 7.77), (7.70 7.69), (7.70 7.76), (7.70 7.90), (7.68 7.69), (7.66 7.77), (7.66 7.90), (7.42 7.42), (7.42 8.04), (7.40 8.04), (7.39 7.40), (6.77 6.78), (3.00 2.99), (2.98 1.63), (1.61 1.54), (1.61 3.00), (1.60 1.62), (1.53 1.53), (1.51 1.63). $^{1}H^{13}C$ HSQC-NMR ((400, 100) MHz, DMSO) δ (8.03 123.03), (7.97 125.81), (7.89 125.26), (7.76 133.92), (7.68 132.07), (7.41 128.15), (6.77 33.81), (2.99 43.87), (1.62 22.28), (1.53 21.77). $^{1}H^{13}C$ HMBC-NMR ((400, 100) MHz, DMSO) δ (8.24 123.25), (8.11 125.68), (8.03 145.41), (8.03 151.00), (8.03 123.20), (7.97 131.61), (7.97 182.45), (7.92 183.22), (7.90 134.43), (7.90 183.50), (7.76 133.36), (7.71 125.88), (7.70 125.73), (7.68 131.23), (7.59 131.29), (7.56 134.04), (7.41 128.33), (7.41 33.87), (7.41 145.40), (6.78 128.40), (6.78 182.43), (6.78 164.88), (6.78 151.01), (6.78 121.80), (3.00 44.04), (3.00 21.46), (3.00 23.01), (1.63 44.12), (1.63 21.49), (1.61 21.58), (1.54 22.43), (1.52 43.90).

Piperidin-1-ium 3-((3-hydroxy-1,4-dioxo-1,4-dihydronaphthalen-2-yl)(phenyl)methyl)-1,4-dioxo-1,4-dihydronaphthalen-2-olate

Orange solid; Mp: 184-185 °C; IR (KBr): υ (cm^{-1}) = 3443, 3253, 3181, 3024, 2936, 2860, 1672, 598, 1570. ^{1}H NMR (400 MHz, DMSO-*d6*) δ 8.25 (s, 1H), 7.96 (dd, *J* = 7.7, 1.2 Hz, 2H), 7.87 (dd, *J* = 7.6, 1.3 Hz, 2H), 7.75 (td, *J* = 7.5, 1.4 Hz, 2H), 7.66 (td, *J* = 7.5, 1.3 Hz, 2H), 7.18 – 7.09 (m, 4H), 7.06 (t, *J* = 7.0 Hz, 1H), 6.69 (s, 1H), 2.98 (t, *J* = 5.6 Hz, 4H), 1.61 (p, *J* = 5.7 Hz, 4H), 1.52 (qd, *J* = 7.6, 2.3 Hz, 2H). ^{13}C NMR (DEPT-135) (100 MHz, DMSO-*d6*) δ 183.7, 182.4, 164.6, 141.5, 133.8, 133.3, 131.9, 131.0, 127.7, 126.8, 125.8, 125.1, 124.8, 122.5, 43.8, 33.0, 22.2, 21.6. ^{1}H^{1}H COSY-NMR ((400, 400) MHz, DMSO-*d6*) δ (7.97 7.96), (7.97 7.75), (7.95 7.96), (7.89 7.87), (7.88 7.67), (7.87 7.68), (7.87 7.87), (7.77 7.97), (7.77 7.66), (7.75 7.75), (7.73 7.97), (7.73 7.68), (7.73 7.74), (7.68 7.68), (7.68 7.75), (7.68 7.88), (7.66 7.67), (7.64 7.76), (7.64 7.88), (7.17 7.11), (7.15 7.15), (7.13 7.08), (7.12 7.13), (7.10 7.16), (7.07 7.13), (7.06 7.06), (6.69 6.70), (3.00 1.61), (2.99 3.00), (1.62 1.53), (1.61 1.61), (1.60 2.99), (1.53 1.62), (1.52 1.53). ^{1}H^{13}C HSQC-NMR ((400, 100) MHz, DMSO-*d6*) δ (7.96 125.82), (7.87 125.17), (7.74 133.91), (7.66 131.98), (7.15 127.83), (7.13 124.72), (7.12 127.21), (7.06 124.83), (6.69 33.05), (2.98 43.82),, (1.61 22.18), (1.52 21.70). ^{1}H^{13}C HMBC-NMR ((400, 100) MHz, DMSO-*d6*) δ (8.16 125.95), (8.07 125.36), (7.96 134.00), (7.88 133.79), (7.88 183.85), (7.76 124.86), (7.75 133.54), (7.75 126.34), (7.69 125.92), (7.66 131.27), (7.66 125.04), (7.65 126.71), (7.55 133.94), (7.54 133.79), (7.47 132.12), (7.16 127.87), (7.15 141.65), (7.11 127.14), (7.11 33.28), (7.11 124.86), (7.06 126.99), (6.95 137.56), (6.70 141.65), (6.70 126.93), (6.70 164.71), (6.70 122.72), (6.70 182.61), (2.99 21.32), (2.99 23.06), (2.98 44.10), (1.62 43.94), (1.61 21.70), (1.52 44.11), (1.52 22.56).

Piperidin-1-ium 3-((3-hydroxy-1,4-dioxo-1,4-dihydronaphthalen-2-yl)(p-tolyl)methyl)-1,4-dioxo-1,4-dihydronaphthalen-2-olate

Orange solid; Mp: 239-240 °C; IR (KBr): υ (cm^{-1}) = ^{1}H NMR (400 MHz, DMSO-*d6*) δ 8.28 (s, 1H), 7.96 (dd, *J* = 7.7, 1.2 Hz, 2H), 7.87 (dd, *J* = 7.5, 1.3 Hz, 2H),

7.74 (td, *J* = 7.5, 1.4 Hz, 2H), 7.66 (td, *J* = 7.5, 1.3 Hz, 2H), 6.99 (d, *J* = 8.1 Hz, 2H), 6.95 (d, *J* = 8.2 Hz, 2H), 6.64 (s, 1H), 3.01 – 2.95 (m, 4H), 2.21 (s, 3H), 1.60 (p, *J* = 5.6 Hz, 4H), 1.51 (qt, *J* = 5.5, 3.7, 3.1 Hz, 2H). ^{13}C NMR (DEPT-135) (100 MHz, DMSO-*d6*) δ 183.7, 182.4, 164.7, 138.3, 133.8, 133.5, 133.4, 131.9, 131.0, 128.3, 126.7, 125.7, 125.1, 122.6, 43.8, 32.7, 22.2, 21.6, 20.5. ^{1}H^{1}H COSY-NMR ((400, 400) MHz, DMSO-*d6*) δ (7.97 7.96), (7.97 7.74), (7.95 7.74), (7.95 7.95), (7.88 7.87), (7.88 7.67), (7.86 7.67), (7.86 7.87), (7.76 7.96), (7.76 7.66), (7.75 7.75), (7.73 7.96), (7.73 7.67), (7.73 7.74), (7.68 7.67), (7.68 7.74), (7.68 7.87), (7.66 7.66), (7.64 7.76), (7.64 7.87), (7.64 7.66), (7.00 6.95), (6.98 6.98), (6.96 6.97), (6.94 7.00), (6.64 6.64), (2.98 2.97), (2.97 1.60), (2.21 2.21), (1.62 2.98), (1.61 1.51), (1.60 1.60), (1.51 1.52), (1.50 1.61). ^{1}H^{13}C HSQC-NMR ((400, 100) MHz, DMSO-*d6*) δ (7.96 125.89), (7.96 124.44), (7.87 125.24), (7.87 123.79), (7.76 132.54), (7.75 133.98), (7.66 132.03), (7.66 130.56), (7.01 128.43), (6.98 126.92), (6.98 125.52), (6.96 128.54), (6.93 127.00), (6.64 32.74), (2.98 43.87), (2.21 20.60), (1.60 22.24), (1.52 21.71). ^{1}H^{13}C HMBC-NMR ((400, 100) MHz, DMSO-*d6*) δ (8.15 125.83), (8.06 125.12), (7.96 182.49), (7.96 131.18), (7.95 133.74), (7.87 183.68), (7.87 133.61), (7.75 133.35), (7.75 126.12), (7.66 130.98), (7.66 124.86), (7.65 133.52), (7.55 133.69), (7.46 131.94), (6.99 126.74), (6.99 133.49), (6.98 32.72), (6.95 20.52), (6.95 138.32), (6.94 128.36), (6.64 126.75), (6.64 182.42), (6.64 164.50), (6.64 122.65), (6.64 138.33), (2.99 21.31), (2.98 43.87), (2.97 22.80), (2.37 20.55), (2.21 128.36), (2.21 133.48), (2.05 20.56), (1.60 21.49), (1.60 43.89), (1.52 43.80), (1.51 22.27).

Piperidin-1-ium 3-((3-hydroxy-1,4-dioxo-1,4-dihydronaphthalen-2-yl)(4-hydroxyphenyl)methyl)-1,4-dioxo-1,4-dihydronaphthalen-2-olate

Orange solid; Mp: 230-232 °C; IR (KBr): υ (cm^{-1}) = 3447, 3243, 3072, 2947, 2866, 1675, 1645, 1598. ^{1}H NMR (400 MHz, DMSO-*d6*) δ 8.28 (s, 1H), 7.95 (dd, *J* = 7.7, 1.2 Hz, 2H), 7.87 (dd, *J* = 7.6, 1.3 Hz, 2H), 7.76 (td, *J* = 7.5, 1.4 Hz, 2H), 7.67 (td, *J* = 7.5, 1.3 Hz, 2H), 7.57 (dd, *J* = 7.9, 1.3 Hz, 1H), 7.48 (td, *J* = 7.6, 1.4 Hz,

1H), 7.44 – 7.39 (m, 1H), 7.35 (t, *J* = 7.7 Hz, 1H), 6.85 (s, 1H), 2.98 (t, *J* = 5.6 Hz, 4H), 1.60 (p, *J* = 5.7 Hz, 4H), 1.51 (qd, *J* = 6.7, 5.4, 3.1 Hz, 2H). $^{1}H^{1}H$ COSY-NMR ((400, 400) MHz, DMSO-*d6*) δ (8.28 8.29), (7.96 7.95), (7.96 7.75), (7.94 7.76), (7.94 7.94), (7.88 7.68), (7.88 7.87), (7.86 7.68), (7.86 7.87), (7.78 7.77), (7.78 7.68), (7.78 7.95), (7.76 7.85), (7.76 7.76), (7.76 7.95), (7.74 7.96), (7.74 7.68), (7.73 7.76), (7.69 7.95), (7.69 7.76), (7.69 7.69), (7.69 7.88), (7.67 7.94), (7.67 7.68), (7.65 7.94), (7.65 7.77), (7.65 7.88), (7.59 7.36), (7.59 7.57), (7.57 7.36), (7.56 7.57), (7.50 7.42), (7.48 7.49), (7.47 7.43), (7.43 7.44), (7.41 7.49), (7.37 7.47), (7.37 7.37), (7.37 7.58), (7.35 7.36), (7.33 7.58), (7.33 7.48), (7.33 7.36), (6.85 7.43), (6.85 6.86), (2.97 1.60), (2.96 2.99), (1.61 1.52), (1.60 1.61), (1.57 2.99), (1.51 1.53), (1.50 1.61). $^{1}H^{13}C$ HSQC-NMR ((400, 100) MHz, DMSO-*d6*) δ (7.95 127.56), (7.95 126.17), (7.87 125.65), (7.87 127.12), (7.75 134.34), (7.67 132.48), (7.66 133.91), (7.57 124.33), (7.57 125.76), (7.48 133.44), (7.48 132.05), (7.42 130.44), (7.40 131.91), (7.35 127.05), (6.85 31.90), (2.98 44.24), (1.60 22.62), (1.52 22.10). $^{1}H^{13}C$ HMBC-NMR ((400, 100) MHz, DMSO-*d6*) δ (8.15 126.18), (8.07 125.75), (7.95 182.40), (7.95 131.93), (7.87 183.50), (7.87 134.01), (7.77 125.63), (7.76 133.40), (7.67 131.53), (7.67 126.42), (7.58 150.09), (7.58 132.09), (7.58 134.95), (7.49 124.44), (7.49 134.84), (7.45 132.49), (7.41 127.05), (7.41 150.02), (7.41 31.89), (7.36 124.36), (7.35 130.44), (7.35 150.00), (6.86 164.69), (6.86 182.36), (6.85 150.04), (6.85 134.83), (6.85 121.25), (2.99 21.90), (2.98 44.28), (2.98 23.21), (1.60 21.96), (1.60 44.43), (1.52 44.24), (1.52 22.62).

Piperidin-1-ium 3-((3-hydroxy-1,4-dioxo-1,4-dihydronaphthalen-2-yl)(3-methoxyphenyl)methyl)-1,4-dioxo-1,4-dihydronaphthalen-2-olate

Orange solid; Mp: 218-220 °C; IR (KBr): υ (cm^{-1}) = 3404, 3132, 3072, 2948, 2840, 1669, 1636, 1597. ^{1}H NMR (400 MHz, DMSO-*d6*) δ 8.32 (s, 1H), 7.93 (dd, *J* = 7.7, 1.2 Hz, 2H), 7.85 (dd, *J* = 7.6, 1.3 Hz, 2H), 7.72 (td, *J* = 7.5, 1.4 Hz, 2H), 7.63 (td, *J* = 7.5, 1.3 Hz, 2H), 6.73 – 6.67 (m, 2H), 6.58 – 6.52 (m, 2H), 3.68 (s, 3H),

2.98 (t, J = 5.6 Hz, 4H), 1.61 (p, J = 5.8 Hz, 4H), 1.52 (q, J = 6.2, 5.7 Hz, 2H). ^{13}C NMR (DEPT-135) (100 MHz, DMSO-*d6*) δ 183.9, 182.2, 164.0, 146.9, 143.9, 133.6, 133.4, 131.7, 131.1, 128.3, 125.6, 125.0, 123.1, 121.6, 117.3, 109.1, 55.8, 43.8, 30.3, 22.2, 21.7. ^{1}H^{1}H COSY-NMR ((400, 400) MHz, DMSO-*d6*) δ (7.94 7.93), (7.94 7.72), (7.92 7.72), (7.92 7.92), (7.86 7.85), (7.86 7.65), (7.84 7.65), (7.84 7.85), (7.74 7.93), (7.72 7.73), (7.70 7.93), (7.70 7.64), (7.70 7.72), (7.66 7.72), (7.65 7.85), (7.63 7.64), (7.62 7.73), (7.62 7.85), (6.72 6.57), (6.72 6.71), (6.70 6.57), (6.70 6.71), (6.68 6.56), (6.68 6.69), (6.57 6.56), (6.57 6.71), (6.55 6.71), (6.55 6.55), (6.53 6.71), (6.53 6.56), (3.68 3.68), (2.98 3.00), (2.97 1.61), (1.62 1.53), (1.61 1.61), (1.60 2.99), (1.52 1.62), (1.52 1.53). ^{1}H^{13}C HSQC-NMR ((400, 100) MHz, DMSO-*d6*) δ (7.94 125.75), (7.85 125.06), (7.85 123.63), (7.73 132.46), (7.73 133.81), (7.65 130.43), (7.64 131.87), (6.72 120.38), (6.72 121.80), (6.70 109.23), (6.56 30.36), (6.56 117.38), (3.69 55.83), (2.99 43.87), (1.61 22.23), (1.53 21.78). ^{1}H^{13}C HMBC-NMR ((400, 100) MHz, DMSO-*d6*) δ (8.12 125.73), (8.04 125.10), (7.93 132.29), (7.93 182.31), (7.93 130.81), (7.92 133.76), (7.85 133.67), (7.85 184.00), (7.84 131.95), (7.73 124.75), (7.72 133.58), (7.64 131.21), (7.63 133.77), (7.63 124.75), (7.54 133.57), (6.71 109.28), (6.71 30.54), (6.71 144.04), (6.70 121.81), (6.70 147.31), (6.69 117.54), (6.55 182.33), (6.55 123.20), (6.55 164.12), (6.55 143.97), (6.55 128.46), (6.55 147.08), (3.68 147.15), (2.99 21.46), (2.99 44.02), (2.98 22.96), (1.60 43.93), (1.60 21.55), (1.53 43.88), (1.51 22.41).

Piperidin-1-ium 3-((3-bromophenyl)(3-hydroxy-1,4-dioxo-1,4-dihydronaphthalen-2-yl)methyl)-1,4-dioxo-1,4-dihydronaphthalen-2-olate

Orange solid; Mp: 248-250 °C; IR (KBr): υ (cm^{-1}) = 3437, 3147, 3061, 2951, 2860, 1676, 1632, 1598. ^{1}H NMR (400 MHz, DMSO-*d6*) δ 8.26 (s, 1H), 7.96 (dd, J = 7.7, 1.2 Hz, 2H), 7.88 (dd, J = 7.6, 1.3 Hz, 2H), 7.75 (td, J = 7.5, 1.4 Hz, 2H), 7.66 (td, J = 7.5, 1.3 Hz, 2H), 7.32 (d, J = 8.5 Hz, 2H), 7.08 (d, J = 8.0 Hz, 2H), 6.63 (s, 1H), 2.99 (t, J = 5.6 Hz, 4H), 1.61 (p, J = 5.6 Hz, 4H), 1.52 (dp, J = 8.4, 3.0 Hz,

2H). ^{13}C NMR (DEPT-135) (100 MHz, DMSO-*d6*) δ 183.5, 182.4, 164.6, 141.1, 133.8, 133.3, 132.0, 131.0, 130.6, 129.2, 125.8, 125.2, 122.1, 117.8, 43.8, 32.7, 22.2, 21.7. ^{1}H^{1}H COSY-NMR ((400, 400) MHz, DMSO-*d6*) δ (7.97 7.96), (7.97 7.75), (7.95 7.75), (7.95 7.96), (7.89 7.88), (7.88 7.68), (7.87 7.68), (7.86 7.88), (7.77 7.76), (7.77 7.97), (7.77 7.67), (7.75 7.76), (7.73 7.97), (7.73 7.68), (7.73 7.75), (7.68 7.68), (7.68 7.76), (7.68 7.88), (7.66 7.67), (7.64 7.88), (7.64 7.77), (7.33 7.32), (7.33 7.08), (7.31 7.08), (7.31 7.32), (7.09 7.09), (7.09 7.33), (7.07 7.33), (7.06 7.09), (6.63 6.64), (2.99 2.98), (2.97 1.61), (1.62 1.52), (1.61 1.62), (1.60 2.99), (1.53 1.53), (1.52 1.63). ^{1}H^{13}C HSQC-NMR ((400, 100) MHz, DMSO-*d6*) δ (7.96 127.23), (7.96 125.84), (7.88 126.64), (7.87 125.23), (7.75 133.94), (7.66 132.04), (7.64 133.60), (7.33 132.05), (7.32 130.63), (7.08 127.90), (7.08 129.36), (6.64 32.85), (2.99 43.88), (1.61 22.26), (1.53 21.78). ^{1}H^{13}C HMBC-NMR ((400, 100) MHz, DMSO-*d6*) δ (8.15 125.89), (8.07 125.26), (7.96 182.44), (7.96 131.40), (7.87 183.56), (7.87 132.73), (7.77 124.83), (7.75 130.83), (7.75 133.33), (7.75 126.23), (7.67 125.00), (7.67 131.02), (7.56 133.76), (7.52 130.64), (7.47 132.02), (7.44 132.10), (7.33 117.79), (7.33 141.18), (7.32 130.59), (7.08 32.84), (7.08 129.40), (7.08 117.85), (6.64 129.39), (6.64 182.42), (6.64 164.61), (6.64 122.13), (6.64 141.17), (2.99 21.39), (2.99 43.95), (2.99 22.86), (1.61 21.56), (1.61 43.86), (1.54 43.79), (1.52 22.35).

Piperidin-1-ium 3-((3-hydroxy-1,4-dioxo-1,4-dihydronaphthalen-2-yl)(4-isopropylphenyl)methyl)-1,4-dioxo-1,4-dihydronaphthalen-2-olate

Orange solid; Mp: 240-242 °C; IR (KBr): υ (cm^{-1}) =3092, 2956, 2933, 2865, 2722, 1681, 1646, 1598. ^{1}H NMR (400 MHz, DMSO-*d6*) δ 8.25 (s, 1H), 7.96 (dd, *J* = 7.7, 1.3 Hz, 2H), 7.87 (dd, *J* = 7.5, 1.3 Hz, 2H), 7.75 (td, *J* = 7.6, 1.4 Hz, 2H), 7.66 (td, *J* = 7.5, 1.3 Hz, 2H), 7.01 (s, 4H), 6.64 (s, 1H), 2.99 (t, *J* = 5.6 Hz, 4H), 2.78 (hept, *J* = 6.9 Hz, 1H), 1.61 (p, *J* = 5.6 Hz, 4H), 1.53 (q, *J* = 5.5 Hz, 2H), 1.15 (d, *J* = 6.9 Hz, 6H). ^{1}H^{1}H COSY-NMR ((400, 400) MHz, DMSO-*d6*) δ (7.97 7.96), (7.97 7.75), (7.95 7.75), (7.95 7.96), (7.88 7.88), (7.88 7.67), (7.86 7.67), (7.86

7.87), (7.77 7.76), (7.77 7.96), (7.76 7.67), (7.75 7.76), (7.73 7.96), (7.73 7.67), (7.73 7.75), (7.68 7.68), (7.68 7.75), (7.68 7.88), (7.66 7.67), (7.64 7.77), (7.64 7.88), (7.01 7.02), (6.64 6.65), (3.00 1.62), (3.00 2.99), (2.97 1.62), (2.82 1.16), (2.80 1.16), (2.77 1.16), (2.75 1.16), (1.62 1.52), (1.60 2.99), (1.60 1.61), (1.53 1.54), (1.51 1.63), (1.16 1.16), (1.16 2.79), (1.14 2.79), (1.14 1.16). $^{1}H^{13}C$ HSQC-NMR ((400, 100) MHz, DMSO-*d6*) δ (7.96 127.58), (7.96 126.19), (7.87 124.10), (7.87 125.55), (7.87 126.99), (7.75 134.25), (7.74 135.68), (7.66 132.33), (7.02 128.56), (7.01 126.59), (6.64 33.10), (2.99 44.23), (2.78 33.36), (1.61 22.62), (1.53 22.10), (1.15 24.42). $^{1}H^{13}C$ HMBC-NMR ((400, 100) MHz, DMSO-*d6*) δ (8.15 126.23), (8.07 125.57), (7.96 182.96), (7.96 131.72), (7.95 134.26), (7.88 134.08), (7.87 184.14), (7.76 125.16), (7.75 133.78), (7.75 126.62), (7.66 125.28), (7.66 126.76), (7.66 131.45), (7.56 134.26), (7.01 126.38), (7.01 145.04), (7.01 33.29), (7.01 139.22), (6.64 164.98), (6.64 139.21), (6.64 182.87), (6.64 123.07), (6.64 127.12), (2.99 44.25), (2.99 21.71), (2.99 23.31), (2.79 126.05), (2.79 145.20), (2.79 24.49), (1.63 23.39), (1.61 44.36), (1.61 21.93), (1.55 44.16), (1.52 22.74), (1.31 24.47), (1.15 145.05), (1.15 24.51), (1.15 33.42), (0.99 24.49).

Piperidin-1-ium 3-((3,4-difluorophenyl)(3-hydroxy-1,4-dioxo-1,4-dihydronaphthalen-2-yl)methyl)-1,4-dioxo-1,4-dihydronaphthalen-2-olate

Orange solid; Mp: 260-263 °C; IR (KBr): υ (cm^{-1}) = 3433, 3167, 3048, 2945, 2863, 1676, 1635, 1598. ^{1}H NMR (400 MHz, DMSO-*d6*) δ 8.21 (s, 1H), 7.95 (dd, *J* = 7.7, 1.2 Hz, 2H), 7.87 (dd, *J* = 7.6, 1.3 Hz, 2H), 7.75 (td, *J* = 7.5, 1.4 Hz, 2H), 7.66 (td, *J* = 7.5, 1.3 Hz, 2H), 7.17 (dt, *J* = 11.1, 8.8 Hz, 1H), 7.13 – 7.06 (m, 1H), 6.98 – 6.89 (m, 1H), 6.64 (s, 1H), 3.00 (t, *J* = 5.6 Hz, 4H), 1.62 (p, *J* = 5.6 Hz, 4H), 1.53 (tq, *J* = 5.6, 3.7, 3.2 Hz, 2H). ^{13}C NMR (DEPT-135) (100 MHz, DMSO-*d6*) δ 183.5, 182.3, 164.6, 150.5, 150.3, 148.5, 148.4, 148.1, 147.9, 146.1, 145.9, 139.5, 139.5, 139.5, 133.8, 133.3, 131.9, 131.1, 125.7, 125.2, 123.4, 123.4, 123.3, 123.3, 121.8, 116.5, 116.4, 115.8, 115.7, 43.8, 32.6, 22.3, 21.7. $^{1}H^{1}H$ COSY-NMR ((400, ^{400}) MHz, DMSO-*d6*) δ (7.97 7.96), (7.96 7.75), (7.95 7.75), (7.95 7.95), (7.88

7.88), (7.88 7.67), (7.86 7.68), (7.86 7.87), (7.77 7.67), (7.77 7.96), (7.75 7.96), (7.75 7.75), (7.73 7.96), (7.72 7.74), (7.68 7.95), (7.68 7.68), (7.68 7.76), (7.68 7.88), (7.66 7.67), (7.64 7.88), (7.21 7.22), (7.21 6.95), (7.18 7.16), (7.16 6.95), (7.16 7.16), (7.14 6.93), (7.11 7.12), (7.08 7.08), (6.95 6.96), (6.94 7.16), (6.92 6.96), (6.64 6.65), (3.01 3.00), (2.98 1.63), (1.63 1.54), (1.62 1.63), (1.61 3.00), (1.54 1.55), (1.51 1.63). $^{1}H^{13}C$ HSQC-NMR ((400, 100) MHz, DMSO-*d6*) δ (8.05 125.32), (8.02 122.54), (7.95 125.83), (7.88 126.63), (7.87 125.25), (7.75 133.87), (7.66 132.01), (7.17 116.56), (7.11 115.91), (6.94 123.45), (6.64 32.72), (2.99 43.93), (1.62 22.35), (1.53 21.80). $^{1}H^{13}C$ HMBC-NMR ((400, 100) MHz, DMSO-*d6*) δ (8.15 125.79), (8.06 125.34), (7.96 182.46), (7.96 131.07), (7.95 133.88), (7.88 183.56), (7.88 133.79), (7.75 124.85), (7.75 133.44), (7.75 126.21), (7.66 125.12), (7.52 133.94), (7.47 131.91), (7.20 146.29), (7.17 139.63), (7.17 150.48), (7.11 123.44), (7.10 32.88), (6.94 32.73), (6.94 148.39), (6.94 115.78), (6.64 115.60), (6.64 182.41), (6.64 164.74), (6.64 121.97), (6.64 139.63), (3.00 43.97), (2.99 22.96), (2.99 21.40), (1.63 44.22), (1.63 21.60), (1.62 22.99), (1.53 44.00), (1.53 22.25). ^{19}F NMR (376 MHz, DMSO-*d6*) δ -140.38, -140.44, -144.39, -144.45. ^{19}F NMR (spin-spin coupling) (376 MHz, DMSO-*d6*) δ -140.36, -140.38, -140.39, -140.41, -140.44, -140.45, -140.47, -144.36, -144.37, -144.38, -144.38, -144.39, -144.39, -144.40, -144.42, -144.42, -144.43, -144.44, -144.45, -144.45, -144.46, -144.47, -144.48, -144.48.

Piperidin-1-ium 3-((2-bromophenyl)(3-hydroxy-1,4-dioxo-1,4-dihydronaphthalen-2-yl)methyl)-1,4-dioxo-1,4-dihydronaphthalen-2-olate

Orange solid; Mp: 216-218 °C; IR (KBr): υ (cm^{-1}) = 3526, 3239, 3164, 2955, 2857, 1672, 1645, 1595. ^{1}H NMR (400 MHz, DMSO-*d6*) δ 8.26 (s, 1H), 7.94 (dd, *J* = 7.7, 1.2 Hz, 2H), 7.87 (dd, *J* = 7.6, 1.3 Hz, 2H), 7.70 (dtd, *J* = 33.3, 7.5, 1.4 Hz, 4H), 7.44 (dd, *J* = 7.9, 1.3 Hz, 1H), 7.35 (dd, *J* = 7.9, 1.6 Hz, 1H), 7.21 (td, *J* = 7.6, 1.3 Hz, 1H), 7.05 (td, *J* = 7.6, 1.6 Hz, 1H), 6.40 (s, 1H), 3.02 – 2.95 (m, 4H), 1.61 (p, *J* = 5.7 Hz, 4H), 1.52 (tt, *J* = 8.6, 3.0 Hz, 2H). ^{13}C NMR (DEPT-135) (100

MHz, DMSO-*d6*) δ 183.5, 182.1, 164.3, 141.0, 133.8, 133.2, 132.8, 132.0, 131.1, 130.8, 127.4, 126.7, 125.7, 125.2, 123.1, 121.9, 43.8, 35.8, 22.2, 21.6. $^{1}H^{1}H$ COSY-NMR ((400, 400) MHz, DMSO-*d6*) δ (7.95 7.94), (7.95 7.74), (7.93 7.74), (7.93 7.94), (7.88 7.88), (7.88 7.66), (7.86 7.67), (7.86 7.87), (7.76 7.66), (7.76 7.95), (7.74 7.75), (7.72 7.95), (7.72 7.67), (7.68 7.68), (7.68 7.75), (7.68 7.88), (7.66 7.67), (7.64 7.88), (7.45 7.44), (7.45 7.06), (7.43 7.06), (7.43 7.44), (7.36 7.35), (7.36 7.21), (7.34 7.21), (7.34 7.06), (7.34 7.35), (7.23 7.21), (7.23 7.05), (7.23 7.36), (7.21 7.22), (7.19 7.36), (7.19 7.07), (7.19 7.22), (7.07 7.07), (7.07 7.21), (7.07 7.44), (7.05 7.06), (7.05 7.44), (7.03 7.23), (7.03 7.44), (7.03 7.06), (6.40 6.41), (2.99 2.98), (2.97 1.62), (1.62 1.53), (1.61 1.62), (1.60 2.99), (1.53 1.52), (1.51 1.62). $^{1}H^{13}C$ HSQC-NMR ((400, 100) MHz, DMSO-*d6*) δ (7.94 125.78), (7.93 127.21), (7.87 126.63), (7.87 125.22), (7.74 133.93), (7.68 134.03), (7.66 132.06), (7.65 133.51), (7.43 132.92), (7.35 130.91), (7.21 126.78), (7.05 127.50), (6.40 35.92), (2.98 43.87), (1.61 22.27), (1.53 21.81). $^{1}H^{13}C$ HMBC-NMR ((400, 100) MHz, DMSO-*d6*) δ (8.14 125.71), (8.06 125.18), (7.94 131.32), (7.94 182.19), (7.94 133.83), (7.87 183.51), (7.87 134.21), (7.87 132.57), (7.75 125.02), (7.74 133.15), (7.66 125.16), (7.66 131.05), (7.44 123.09), (7.44 126.69), (7.44 140.98), (7.36 36.00), (7.35 127.37), (7.35 123.08), (7.21 132.80), (7.21 140.96), (7.05 130.81), (7.05 123.08), (6.40 140.95), (6.40 122.11), (6.40 164.30), (6.40 182.08), (6.40 130.85), (2.98 22.80), (2.98 43.86), (2.90 23.05), (1.61 21.47), (1.59 43.86), (1.53 22.10), (1.51 43.76).

Piperidin-1-ium 3-((3-hydroxy-1,4-dioxo-1,4-dihydronaphthalen-2-yl)(naphthalen-2-yl)methyl)-1,4-dioxo-1,4-dihydronaphthalen-2-olate

Orange solid; Mp: 259-260 °C; IR (KBr): υ (cm^{-1}) = 3132, 3061, 2959, 2929, 2860, 1679, 1643, 1598. ^{1}H NMR (400 MHz, DMSO-*d6*) δ 8.23 (s, 1H), 7.99 (dd, *J* = 7.6, 1.2 Hz, 2H), 7.90 (dd, *J* = 7.6, 1.3 Hz, 2H), 7.82 – 7.63 (m, 7H), 7.59 (s, 1H), 7.37 (dt, *J* = 6.3, 3.6 Hz, 2H), 7.29 (dd, *J* = 8.6, 1.8 Hz, 1H), 6.83 (d, *J* = 1.4 Hz, 1H), 3.01 – 2.93 (m, 4H), 1.59 (p, *J* = 5.9 Hz, 4H), 1.50 (dt, *J* = 10.9, 5.6 Hz, 2H).

^{13}C NMR (100 MHz, DMSO-*d6*) δ 183.7, 182.5, 164.7, 139.2, 133.8, 133.4, 133.1, 131.9, 131.4, 131.1, 127.5, 127.2, 127.1, 126.3, 125.8, 125.5, 125.2, 124.8, 124.4, 122.4, 43.8, 33.4, 22.2, 21.6. ^{1}H^{1}H COSY-NMR ((400, 400) MHz, DMSO-*d6*) δ (8.00 7.98), (8.00 7.77), (7.98 7.76), (7.98 7.98), (7.91 7.69), (7.91 7.90), (7.89 7.69), (7.89 7.89), (7.79 7.37), (7.79 7.78), (7.79 7.99), (7.79 7.69), (7.77 7.38), (7.77 7.77), (7.75 7.37), (7.75 7.99), (7.75 7.76), (7.73 7.38), (7.70 7.90), (7.70 7.69), (7.70 7.30), (7.68 7.68), (7.68 7.29), (7.66 7.90), (7.59 7.59), (7.38 7.38), (7.38 7.75), (7.36 7.77), (7.36 7.37), (7.31 7.31), (7.31 7.69), (7.29 7.69), (7.28 7.32), (6.83 6.83), (2.97 2.96), (2.96 1.59), (1.61 1.50), (1.59 1.59), (1.58 2.98), (1.51 1.51), (1.50 1.60). ^{1}H^{13}C HSQC-NMR ((400, 100) MHz, DMSO-*d6*) δ (7.99 125.81), (7.91 126.53), (7.90 125.18), (7.78 127.21), (7.76 133.86), (7.74 127.49), (7.68 127.23), (7.68 131.95), (7.59 124.43), (7.37 125.17), (7.30 126.40), (6.83 33.19), (2.97 43.79), (1.59 22.19), (1.50 21.65). ^{1}H^{13}C HMBC-NMR ((400, 100) MHz, DMSO-*d6*) δ (8.18 125.89), (8.09 125.31), (7.99 182.72), (7.99 131.20), (7.98 133.86), (7.91 133.71), (7.90 183.73), (7.78 125.05), (7.77 133.59), (7.74 125.03), (7.69 139.38), (7.69 133.35), (7.68 131.27), (7.59 33.61), (7.59 131.54), (7.59 126.81), (7.57 133.83), (7.49 132.10), (7.39 127.15), (7.30 131.47), (7.29 33.79), (7.29 124.52), (6.83 182.64), (6.83 164.92), (6.83 122.53), (6.83 139.38), (2.98 44.03), (2.98 21.40), (1.59 21.66), (1.59 44.10), (1.53 43.83), (1.50 43.79), (1.49 22.38).

Piperidin-1-ium 3-((3-hydroxy-1,4-dioxo-1,4-dihydronaphthalen-2-yl)(thiophen-2-yl)methyl)-1,4-dioxo-1,4-dihydronaphthalen-2-olate

Orange solid; Mp: 240-241 °C; IR (KBr): υ (cm^{-1}) = ^{1}H NMR (400 MHz, DMSO-*d6*) δ 8.09 (d, *J* = 61.2 Hz, 1H), 7.94 (dd, *J* = 7.7, 1.2 Hz, 2H), 7.87 (dd, *J* = 7.5, 1.3 Hz, 2H), 7.74 (td, *J* = 7.5, 1.4 Hz, 2H), 7.66 (td, *J* = 7.5, 1.3 Hz, 2H), 7.38 (d, *J* = 2.2 Hz, 1H), 7.35 (d, *J* = 8.5 Hz, 1H), 7.24 (dd, *J* = 8.5, 2.3 Hz, 1H), 6.50 (s, 1H), 3.02 – 2.95 (m, 4H), 1.61 (p, *J* = 5.8 Hz, 4H), 1.53 (qd, *J* = 7.5, 4.0 Hz, 2H). ^{13}C NMR (DEPT-135) (100 MHz, DMSO-*d6*) δ 183.4, 182.0, 164.4, 138.8, 133.8,

133.2, 133.1, 132.0, 132.0, 131.0, 130.7, 128.7, 126.2, 125.7, 125.2, 121.3, 43.9, 33.1, 22.3, 21.7. $^1H^1H$ COSY-NMR ((400, 400) MHz, DMSO-*d6*) δ (7.95 7.94), (7.95 7.74), (7.93 7.74), (7.93 7.93), (7.88 7.87), (7.88 7.67), (7.86 7.68), (7.86 7.87), (7.76 7.67), (7.76 7.95), (7.75 7.75), (7.73 7.94), (7.72 7.75), (7.68 7.68), (7.68 7.75), (7.68 7.87), (7.66 7.67), (7.64 7.87), (7.38 7.27), (7.38 7.38), (7.36 7.34), (7.36 7.25), (7.34 7.25), (7.34 7.34), (7.26 7.26), (7.24 7.35), (7.23 7.27), (6.50 6.50), (2.99 2.98), (2.98 1.62), (1.63 2.99), (1.63 1.53), (1.62 1.62), (1.53 1.64), (1.53 1.54). $^1H^{13}C$ HSQC-NMR ((400, 100) MHz, DMSO-*d6*) δ (7.96 124.35), (7.94 125.81), (7.87 125.27), (7.74 133.98), (7.66 132.14), (7.65 130.71), (7.38 128.79), (7.34 132.08), (7.29 127.81), (7.25 126.28), (6.50 33.07), (2.99 43.83), (1.62 22.24), (1.53 21.71). $^1H^{13}C$ HMBC-NMR ((400, 100) MHz, DMSO-*d6*) δ (8.14 125.74), (8.07 125.32), (7.94 131.72), (7.94 182.15), (7.94 134.11), (7.88 183.44), (7.87 132.89), (7.75 125.35), (7.74 133.21), (7.67 126.09), (7.66 131.23), (7.38 138.93), (7.35 133.44), (7.35 130.79), (7.35 133.41), (7.25 138.92), (7.25 128.91), (7.24 130.94), (7.17 128.84), (6.50 133.05), (6.50 164.57), (6.50 121.47), (6.50 138.97), (6.50 182.14), (3.00 21.49), (3.00 44.08), (2.99 23.00), (1.61 21.62), (1.61 44.23), (1.52 22.58).

Piperazine-1,4-diium 3-((2-chlorophenyl)(3-hydroxy-1,4-dioxo-1,4-dihydronaphthalen-2-yl)methyl)-1,4-dioxo-1,4-dihydronaphthalen-2-olate

Orange solid; Mp: 255-257 °C; IR (KBr): υ (cm^{-1}) = 3589, 3490, 3086, 2966, 2929, 1677, 1642, 1602, 1577. ^1H NMR (600 MHz, DMSO-*d6*) δ 9.35 (s, 2H), 7.96 (dd, *J* = 7.7, 1.2 Hz, 2H), 7.90 (dd, *J* = 7.6, 1.3 Hz, 2H), 7.77 (td, *J* = 7.6, 1.3 Hz, 2H), 7.69 (td, *J* = 7.5, 1.3 Hz, 2H), 7.36 (dd, *J* = 7.7, 1.8 Hz, 1H), 7.28 (dd, *J* = 7.6, 1.6 Hz, 1H), 7.17 (td, *J* = 7.8, 1.8 Hz, 2H), 6.51 (s, 1H), 3.21 (s, 4H). ^{13}C NMR (150 MHz, DMSO-*d6*) δ 183.7, 182.6, 139.7, 134.4, 133.4, 133.0, 132.6, 131.3, 131.0, 129.8, 127.6, 126.6, 126.2, 125.7, 122.2, 41.2, 34.1. $^1H^{13}C$ HSQC NMR ((600, 150) MHz, DMSO-*d6*) δ (7.96 126.22), (7.90 125.71), (7.77 134.43), (7.69

132.60), (7.37 131.06), (7.28 129.79), (7.17 127.09), (6.51 34.01), (3.22 41.20). HR-Mass: M= 1027.203, M+H= 1028.206 and base peak ($C_{27}H_{15}ClO_6$= 471.063).

Piperazine-1,4-diium 3-((3,4-difluorophenyl)(3-hydroxy-1,4-dioxo-1,4-dihydronaphthalen-2-yl)methyl)-1,4-dioxo-1,4-dihydronaphthalen-2-olate

Orange solid; Mp: 235-237 °C; IR (KBr): υ (cm^{-1}) = 3618, 3521, 3437, 3122, 2921, 2854, 1673, 1634, 1598. ^{1}H NMR (600 MHz, DMSO-*d6*) δ 8.84 (s, 2H), 7.97 (d, *J* = 7.7 Hz, 2H), 7.89 (d, *J* = 7.5 Hz, 2H), 7.77 (t, *J* = 7.6 Hz, 2H), 7.69 (t, *J* = 7.6 Hz, 2H), 7.18 (dd, *J* = 19.5, 10.0 Hz, 2H), 6.97 (s, 1H), 6.63 (s, 1H), 3.27 (s, 4H). ^{13}C NMR (150 MHz, DMSO-*d6*) δ 183.86, 182.83, 164.64, 150.49, 150.40, 148.87, 148.79, 148.52, 148.43, 146.9, 146.8, 139.8, 139.8, 139.8, 134.3, 133.6, 132.4, 131.4, 126.2, 125.6, 123.9, 123.9, 123.8, 123.8, 122.3, 117.0, 116.9, 116.3, 116.2, 40.9, 33.2. ^{1}H^{13}C HSQC-NMR ((600, 151) MHz, DMSO-*d6*) δ (7.98 126.24), (7.90 125.68), (7.77 134.30), (7.69 132.46), (7.19 116.86), (7.17 116.44), (6.97 123.89), (6.63 33.18), (3.28 40.92). HR-Mass: M=1031.243, M+1= 1032.247 and base peak ($C_{27}H_{14}F_2O_6$= 473.084).

Piperazine-1,4-diium 3-((3-hydroxy-1,4-dioxo-1,4-dihydronaphthalen-2-yl)(3-hydroxyphenyl)methyl)-1,4-dioxo-1,4-dihydronaphthalen-2-olate

Orange solid; Mp: 216-218 °C; IR (KBr): υ (cm^{-1}) = 3380, 3216, 2917, 1662, 1651, 1587. ^{1}H NMR (600 MHz, DMSO-*d6*) δ 8.93 (s, 1H), 8.71 (s, 1H), 7.98 (d, *J* = 7.6 Hz, 2H), 7.90 (d, *J* = 7.6 Hz, 2H), 7.77 (t, *J* = 7.6 Hz, 2H), 7.69 (t, *J* = 7.5 Hz, 2H), 6.95 (t, *J* = 7.8 Hz, 1H), 6.61 (s, 1H), 6.58 (s, 1H), 6.56 (d, *J* = 7.8 Hz, 1H), 6.47 (d, *J* = 8.0 Hz, 1H), 3.26 (s, 4H). ^{13}C NMR (150 MHz, DMSO-*d6*) δ 184.0, 182.9, 164.5, 157.4, 143.3, 134.3, 133.7, 132.4, 131.3, 129.0, 126.2, 125.6, 123.0, 118.1, 114.3, 112.3, 40.9, 33.5. ^{1}H^{13}C HSQC-NMR ((600, 151) MHz, DMSO-*d6*) δ (7.98 126.18), (7.90 125.55), (7.78 134.31), (7.69 132.39), (6.94 128.98), (6.61 33.50), (6.58 114.25), (6.56 118.05), (6.47 112.30), (3.26 40.92). HR-Mass: M= 991.271, M+1= 992.274 and base peak ($C_{27}H_{16}O_7$= 253.096).

Piperazine-1,4-diium 3-((3-hydroxy-1,4-dioxo-1,4-dihydronaphthalen-2-yl)(4-hydroxyphenyl)methyl)-1,4-dioxo-1,4-dihydronaphthalen-2-olate

Orange solid; Mp: 215-217 °C; IR (KBr): υ (cm^{-1}) = 3391, 3017, 2929, 1671, 1595, 1573. ^{1}H NMR (600 MHz, DMSO-*d6*) δ 8.96 (s, 1H), 7.97 (d, *J* = 7.5 Hz, 2H), 7.88 (d, *J* = 7.5 Hz, 2H), 7.76 (t, *J* = 7.7 Hz, 2H), 7.67 (t, *J* = 7.5 Hz, 2H), 6.91 (d, *J* = 8.0 Hz, 2H), 6.57 (d, *J* = 9.1 Hz, 3H), 3.23 (s, 4H). ^{13}C NMR (150 MHz, DMSO-*d6*) δ 184.17, 182.98, 164.66, 155.12, 134., 134.3, 133.7, 133.5, 132.3, 131.6, 131.3, 128.2, 126.34, 126.20, 125.8, 125.5, 123.4, 115.0, 111.1, 41.2, 32.7. ^{1}H^{13}C HSQC-NMR ((600, 151) MHz, DMSO-*d6*) δ (7.97 126.20), (7.88 125.55), (7.76 134.27), (7.67 132.34), (6.91 128.18), (6.58 32.68), (6.57 115.04), (3.23 41.19). HR-Mass: M= 991.271, M+1= 992.274 and base peak ($C_{27}H_{16}O_{7}$= 253.096).

Piperazine-1,4-diium 3-((4-chlorophenyl)(3-hydroxy-1,4-dioxo-1,4-dihydronaphthalen-2-yl)methyl)-1,4-dioxo-1,4-dihydronaphthalen-2-olate

Orange solid; Mp: 230-232 °C; IR (KBr): υ (cm^{-1}) = 3626, 3525, 3114, 2921, 1677, 1598. ^{1}H NMR (600 MHz, DMSO*d6*) δ 8.98 (s, 2H), 7.98 (dd, *J* = 7.6, 1.2 Hz, 2H), 7.89 (dd, *J* = 7.6, 1.3 Hz, 2H), 7.77 (td, *J* = 7.6, 1.4 Hz, 2H), 7.69 (td, *J* = 7.5, 1.3 Hz, 2H), 7.21 (d, *J* = 8.6 Hz, 2H), 7.16 (d, *J* = 8.3 Hz, 2H), 6.65 (s, 1H), 3.28 (s, 4H). ^{13}C NMR (151 MHz, DMSO-*d6*) δ 183.9, 182.9, 164.6, 140.9, 134.3, 133.6, 132.5, 131.4, 129.9, 129.3, 128.1, 126.2, 125.6, 122.6, 40.9, 33.3. ^{1}H^{13}C HSQC-NMR ((600, 151) MHz, DMSO-*d6*) δ (7.98 126.23), (7.90 125.65), (7.77 134.35), (7.68 132.48), (7.22 128.12), (7.16 129.26), (6.65 33.22), (3.28 40.91). HR-Mass: M= 1027.203, M+H= 1028.206 and base peak ($C_{27}H_{15}ClO_{6}$= 471.063).

Piperazine-1,4-diium 3-((3-hydroxy-1,4-dioxo-1,4-dihydronaphthalen-2-yl)(3-nitrophenyl)methyl)-1,4-dioxo-1,4-dihydronaphthalen-2-olate

Orange solid; Mp: 240-242 °C; IR (KBr): υ (cm^{-1}) = 3247, 3173, 2936, 2863, 1672, 1598, 1569, 1354. ^{1}H NMR (600 MHz, DMSO-*d6*) δ 8.86 (s, 1H), 8.00 (dd, *J* = 7.8, 1.3 Hz, 3H), 7.93 (d, *J* = 1.7 Hz, 1H), 7.91 (dd, *J* = 7.7, 1.3 Hz, 2H), 7.79 (td, *J* = 7.5, 1.3 Hz, 2H), 7.71 (dd, *J* = 7.5, 1.3 Hz, 2H), 7.66 (dq, *J* = 7.8, 1.3 Hz, 1H), 7.50 (t, *J* = 8.0 Hz, 1H), 6.78 (s, 1H). ^{13}C NMR (150 MHz, DMSO-*d6*) δ 183.8,

182.8, 164.7, 148.3, 144.6, 134.6, 134.4, 133.6, 132.6, 131.4, 129.8, 126.3, 125.8, 122.0, 121.8, 120.8, 40.9, 33.7. $^{1}H^{13}C$ HSQC-NMR ((600, 151) MHz, DMSO-*d6*) δ (8.00 120.77), (8.00 126.28), (7.93 121.74), (7.91 125.76), (7.79 134.44), (7.70 132.59), (7.66 134.57), (7.50 129.79), (6.78 33.72), (3.27 40.90). HR-Mass: M= 1049.251, M+H= 1050.254 and base peak ($C_{27}H_{15}NO_8$= 482.087)

Piperazine-1,4-diium 3-((3-hydroxy-1,4-dioxo-1,4-dihydronaphthalen-2-yl)(4-(trifluoromethyl)phenyl)methyl)-1,4-dioxo-1,4-dihydronaphthalen-2-olate

Orange solid; Mp: 242-244 °C; IR (KBr): υ (cm^{-1}) = 3064, 2894, 2859, 2745, 2439, 1681, 1640, 1602. ^{1}H NMR (600 MHz, DMSO-*d6*) δ 8.86 (s, 1H), 7.99 (d, *J* = 7.6 Hz, 2H), 7.91 (d, *J* = 7.6 Hz, 2H), 7.78 (t, *J* = 7.5 Hz, 2H), 7.70 (t, *J* = 7.5 Hz, 2H), 7.53 (d, *J* = 8.1 Hz, 2H), 7.38 (d, *J* = 8.1 Hz, 2H), 6.75 (s, 1H), 3.28 (s, 4H). ^{13}C NMR (150 MHz, DMSO-*d6*) δ 183.88, 182.88, 164.72, 147.12, 134.37, 133.61, 132.53, 131.38, 128.10, 126.27, 126.08, 125.99, 125.87, 125.69, 125.15, 125.13, 125.10, 125.08, 124.19, 122.29, 40.89, 33.84. $^{1}H^{13}C$ HSQC-NMR ((600, 151) MHz, DMSO-*d6*) δ (7.99 126.25), (7.91 125.70), (7.78 134.39), (7.70 132.53), (7.53 125.13), (7.38 128.10), (6.75 33.84), (3.28 40.87). HR-Mass: M= 1095.256, M+H= 1060.238 and base peak ($C_{28}H_{15}F_3O_6$= 505.089).

Piperazine-1,4-diium 3-((3-hydroxy-1,4-dioxo-1,4-dihydronaphthalen-2-yl)(thiophen-2-yl)methyl)-1,4-dioxo-1,4-dihydronaphthalen-2-olate

Orange solid; Mp: 215-217 °C; IR (KBr): υ (cm^{-1}) = 3559, 3501, 2981, 2853, 2853, 2762, 2639, 1671, 1597. ^{1}H NMR (600 MHz, DMSO-*d6*) δ 8.90 (d, *J* = 19.7 Hz, 1H), 7.99 (dd, *J* = 7.8, 1.2 Hz, 2H), 7.89 (dd, *J* = 7.6, 1.2 Hz, 2H), 7.78 (td, *J* = 7.5, 1.4 Hz, 2H), 7.69 (td, *J* = 7.5, 1.3 Hz, 2H), 7.17 (d, *J* = 5.0 Hz, 1H), 6.85 (s, 1H), 6.81 (dd, *J* = 5.1, 3.5 Hz, 1H), 6.69 (dt, *J* = 3.3, 1.4 Hz, 1H), 3.30 (s, 4H). ^{13}C NMR (150 MHz, DMSO-*d6*) δ 184.00, 182.35, 164.85, 146.95, 134.44, 133.56, 132.51, 131.27, 126.59, 126.31, 125.68, 124.04, 123.28, 122.91, 40.86, 30.11. $^{1}H^{13}C$ HSQC-NMR ((600, 151) MHz, DMSO-*d6*) δ (7.99 126.28), (7.90 125.68), (7.78 134.45), (7.68 132.51), (7.17 123.28), (6.85 30.12), (6.81 126.57), (6.69

124.05), (3.30 40.87). HR-Mass: M= 971.194, M+H= 972.197 and base peak ($C_{25}H_{14}O_6S$= 443.058).

Piperazine-1,4-diium 3-((3-hydroxy-1,4-dioxo-1,4-dihydronaphthalen-2-yl)(pyridin-3-yl)methyl)-1,4-dioxo-1,4-dihydronaphthalen-2-olate

Orange solid; Mp: 248-250 °C; IR (KBr): υ (cm^{-1}) = 2893, 2497, 1674, 1655, 1604, 1574. ^{1}H NMR (600 MHz, DMSO-*d6*) δ 8.70 (s, 1H), 8.39 – 8.32 (m, 2H), 7.98 (dd, *J* = 7.7, 1.2 Hz, 2H), 7.90 (dd, *J* = 7.7, 1.3 Hz, 2H), 7.77 (td, *J* = 7.6, 1.4 Hz, 2H), 7.69 (td, *J* = 7.5, 1.3 Hz, 2H), 7.61 (d, *J* = 8.2 Hz, 1H), 7.28 (dd, *J* = 8.0, 4.8 Hz, 1H), 6.72 (s, 1H), 3.18 (s, 4H). ^{13}C NMR (150 MHz, DMSO-*d6*) δ 183.8, 148.0, 145.70, 137.80, 134.3, 133.6, 132.4, 131.5, 126.2, 125.7, 123.8, 121.8, 41.5, 31.7. $^{1}H^{13}C$ HSQC-NMR ((600, 151) MHz, DMSO-*d6*) δ (8.34 145.76), (7.98 126.22), (7.90 125.68), (7.78 134.31), (7.69 132.48), (7.61 136.20), (7.28 123.80), (6.72 31.42), (3.18 41.45). HR-Mass: M= 961.272, M+H= 962.275 and base peak ($C_{26}H_{15}NO_6$= 438.098).

Piperazine-1,4-diium 3-((3-hydroxy-1,4-dioxo-1,4-dihydronaphthalen-2-yl)(p-tolyl)methyl)-1,4-dioxo-1,4-dihydronaphthalen-2-olate

Orange solid; Mp: 225-227 °C; IR (KBr): υ (cm^{-1}) = 3477, 3107, 2923, 2498, 1675, 163, 1598. ^{1}H NMR (600 MHz, DMSO-*d6*) δ 8.94 (s, 1H), 7.97 (dd, *J* = 7.7, 1.3 Hz, 2H), 7.89 (dd, *J* = 7.6, 1.3 Hz, 2H), 7.77 (td, *J* = 7.6, 1.4 Hz, 2H), 7.68 (td, *J* = 7.5, 1.3 Hz, 2H), 7.02 (d, *J* = 8.0 Hz, 2H), 6.97 (d, *J* = 8.0 Hz, 2H), 6.63 (s, 1H), 3.26 (s, 4H), 2.23 (s, 3H). ^{13}C NMR (150 MHz, DMSO-*d6*) δ 184.1, 183.0, 164.5, 138.6, 134.3, 134.0, 133.7, 132.4, 131.3, 128.8, 127.2, 126.2, 125.6, 123.1, 40.9, 33.3, 21.0. $^{1}H^{13}C$ HSQC-NMR ((600, 151) MHz, DMSO-*d6*) δ (7.98 126.21), (7.88 125.58), (7.77 134.32), (7.68 132.40), (7.01 127.21), (6.97 128.82), (6.63 33.26), (3.26 40.91), (2.23 20.97). HR-Mass: 987.312, M+H= 988.316 and base peak ($C_{28}H_{18}O_6$= 451.117).

Piperazine-1,4-diium 3-((3-hydroxy-1,4-dioxo-1,4-dihydronaphthalen-2-yl)(phenyl)methyl)-1,4-dioxo-1,4-dihydronaphthalen-2-olate

Orange solid; Mp: 220-222 °C; IR (KBr): υ (cm^{-1}) = 3447, 3021, 1672, 1637, 1598. ^{1}H NMR (600 MHz, DMSO-*d6*) δ 8.89 (s, 1H), 7.98 (dd, *J* = 7.7, 1.3 Hz, 2H), 7.89 (dd, *J* = 7.6, 1.3 Hz, 2H), 7.77 (td, *J* = 7.5, 1.4 Hz, 2H), 7.68 (td, *J* = 7.5, 1.4 Hz, 2H), 7.20 – 7.12 (m, 4H), 7.08 (t, *J* = 7.0 Hz, 1H), 6.70 (s, 1H), 3.24 (s, 4H). ^{13}C NMR (150 MHz, DMSO-*d6*) δ 184.1, 183.0, 141.7, 134.3, 133.7, 132.4, 131.4, 128.2, 127.3, 126.2, 125.6, 125.3, 122.9, 41.0, 33.6. $^{1}H^{13}C$ HSQC-NMR ((600, 151) MHz, DMSO-*d6*) δ (7.98 126.24), (7.89 125.63), (7.77 134.36), (7.68 132.46), (7.16 127.97), (7.15 127.39), (7.08 125.32), (6.70 33.53), (3.24 40.96). HR-Mass: M=959.281, M+H= 960.264 and base peak ($C_{27}H_{16}O_6$= 437.101).

Piperazine-1,4-diium 3-((3,4-dimethoxyphenyl)(3-hydroxy-1,4-dioxo-1,4-dihydronaphthalen-2-yl)methyl)-1,4-dioxo-1,4-dihydronaphthalen-2-olate

Orange solid; Mp: 245-247 °C; IR (KBr): υ (cm^{-1}) = 3436, 3199, 3064, 2926, 1618, 1587. ^{1}H NMR (600 MHz, DMSO-*d6*) δ 9.17 (s, 1H), 8.00 – 7.96 (m, 2H), 7.88 (dd, *J* = 7.6, 1.3 Hz, 2H), 7.76 (td, *J* = 7.5, 1.3 Hz, 2H), 7.67 (td, *J* = 7.5, 1.3 Hz, 2H), 6.76 (d, *J* = 8.4 Hz, 1H), 6.71 (s, 1H), 6.67 (d, *J* = 8.3 Hz, 1H), 6.61 (s, 1H), 3.69 (s, 3H), 3.57 (s, 3H), 3.28 (s, 4H). ^{13}C NMR (151 MHz, DMSO-*d6*) δ .184.1, 183.0, 164.6, 148.8, 147.1, 134.3, 134.1, 133.7, 132.4, 131.3, 126.2, 125.6, 123.2, 119.5, 112.0, 111.9, 56.0, 40.9, 33.3. $^{1}H^{13}C$ HSQC-NMR ((600, 151) MHz, DMSO-*d6*) δ (7.98 126.22), (7.88 125.60), (7.76 134.33), (7.68 134.42), (7.67 132.40), (6.75 111.98), (6.72 111.93), (6.67 119.52), (6.61 33.26), (3.69 56.00), (3.57 56.00), (3.28 40.93).

Piperazine-1,4-diium 3-((3-hydroxy-1,4-dioxo-1,4-dihydronaphthalen-2-yl)(4-nitrophenyl)methyl)-1,4-dioxo-1,4-dihydronaphthalen-2-olate

Orange solid; Mp: 260-262 °C; IR (KBr): υ (cm^{-1}) = 3074, 2438, 1682, 1598, 1571, 1342. ^{1}H NMR (600 MHz, DMSO-*d6*) δ 8.89 (s, 1H), 8.08 – 8.03 (m, 2H), 7.99 (dd, *J* = 7.7, 1.2 Hz, 2H), 7.91 (dd, *J* = 7.6, 1.2 Hz, 2H), 7.78 (td, *J* = 7.5, 1.3 Hz, 2H), 7.70 (td, *J* = 7.5, 1.3 Hz, 2H), 7.45 (d, *J* = 8.5 Hz, 2H), 6.78 (s, 1H), 3.27 (s, 4H). ^{13}C NMR (151 MHz, DMSO-*d6*) δ 183.8, 182.8, 164.8, 151.1, 145.7, 134.4,

133.6, 132.6, 131.4, 128.6, 126.3, 125.7, 123.5, 122.0, 40.9, 34.2. ^{1}H-^{13}C NMR ((600, 151) MHz, DMSO-*d6*) δ (8.06 123.55), (7.99 126.28), (7.91 125.76), (7.78 134.44), (7.70 132.60), (7.45 128.63), (6.78 34.19), (3.28 40.86). HR-Mass: M= 1049.251, M+H= 1050.254 and base peak ($C_{27}H_{15}NO_8$= 482.087).

Piperazine-1,4-diium 3-((3-hydroxy-1,4-dioxo-1,4-dihydronaphthalen-2-yl)(4-isopropylphenyl)methyl)-1,4-dioxo-1,4-dihydronaphthalen-2-olate

Orange solid; Mp: 213-214 °C; IR (KBr): υ (cm^{-1}) = 3466, 3018, 2954, 2866, 1675, 1598. ^{1}H NMR (600 MHz, DMSO-*d6*) δ 8.88 (s, 1H), 8.00 – 7.96 (m, 2H), 7.89 (dd, *J* = 7.6, 1.3 Hz, 2H), 7.77 (td, *J* = 7.5, 1.4 Hz, 2H), 7.68 (td, *J* = 7.5, 1.3 Hz, 2H), 7.04 (s, 4H), 6.65 (s, 1H), 3.26 (s, 4H), 2.80 (hept, *J* = 6.8 Hz, 1H), 1.16 (d, *J* = 6.9 Hz, 6H). ^{13}C NMR (150 MHz, DMSO-*d6*) δ 184.1, 183.0, 164.6, 145.1, 139.0, 134.3, 133.7, 132.4, 131.3, 127.2, 126.2, 126.1, 125.6, 123.0, 41.0, 33.4, 33.2, 24.5. ^{1}H^{13}C HSQC-NMR ((600, 151) MHz, DMSO-*d6*) δ (7.98 126.22), (7.89 125.60), (7.77 134.34), (7.68 132.42), (7.04 126.78), (6.65 33.22), (3.25 40.96), (2.80 33.38), (1.16 24.46). HR-Mass : M= 1043.375, M+H= 1044.387 and base peak ($C_{30}H_{22}O_6$= 479.149).

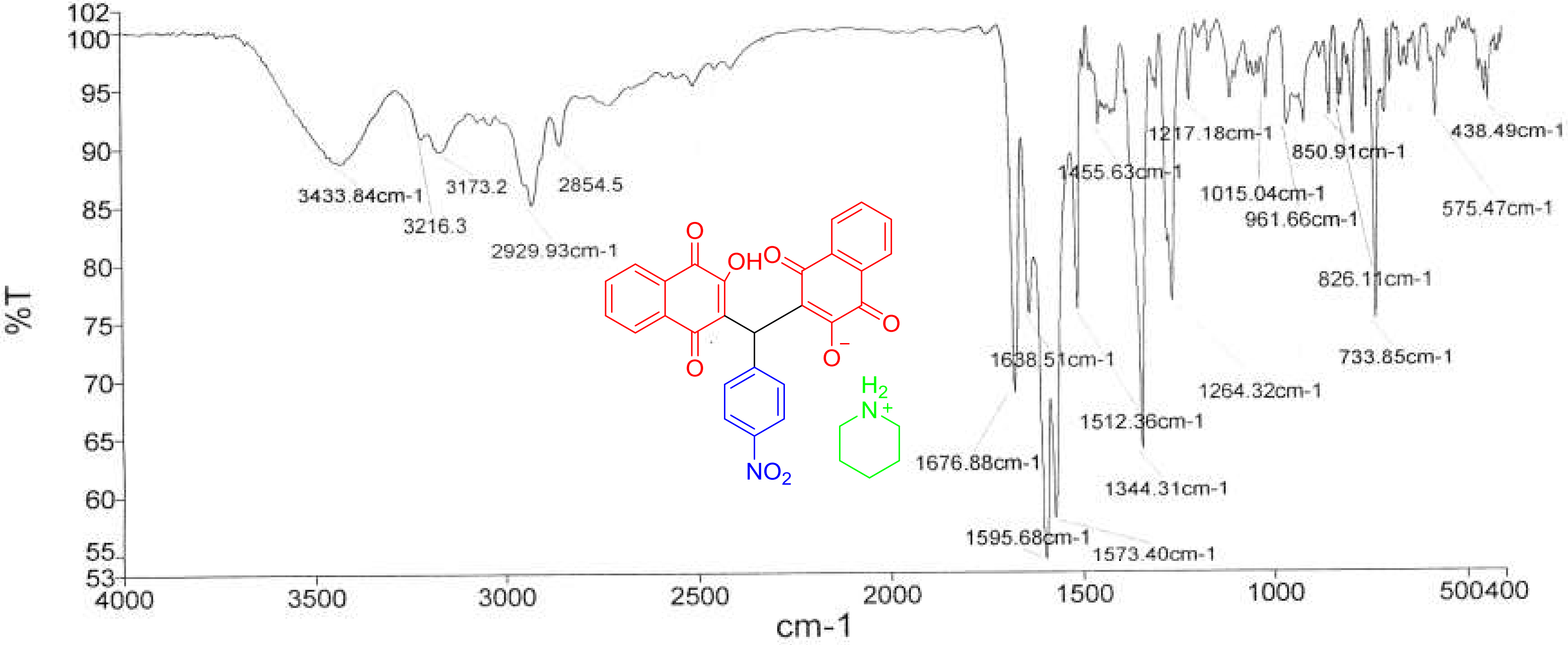

FT-IR spectrum of piperidin-1-ium 3-((3-hydroxy-1,4-dioxo-1,4-dihydronaphthalen-2-yl)(phenyl)methyl)-1,4-dioxo-1,4-dihydronaphthalen-2-olate

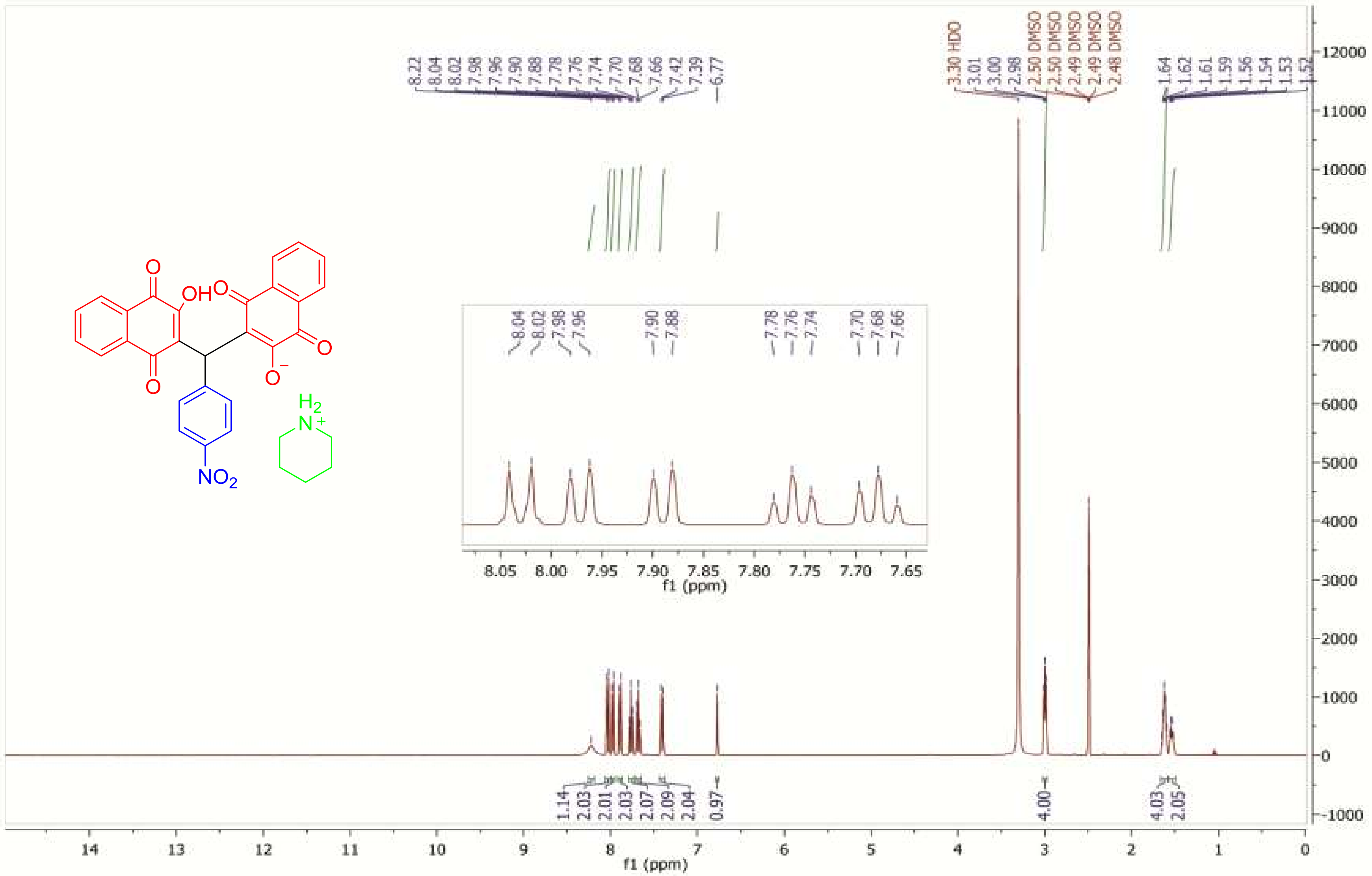

^{1}H NMR spectrum of piperidin-1-ium 3-((3-hydroxy-1,4-dioxo-1,4-dihydronaphthalen-2-yl)(phenyl)methyl)-1,4-dioxo-1,4-dihydronaphthalen-2-olate

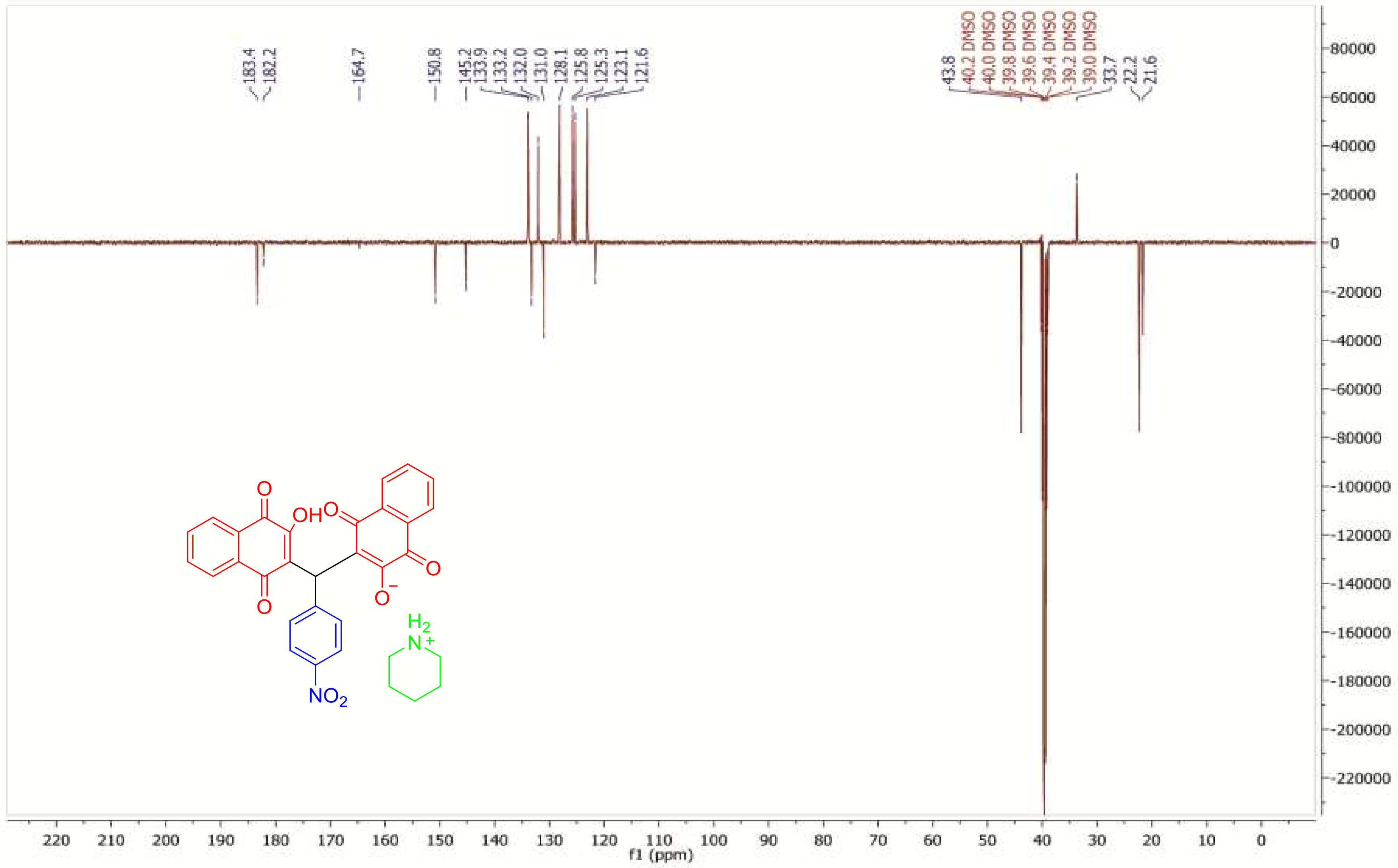

^{13}C NMR (DEPT-135) spectrum of piperidin-1-ium 3-((3-hydroxy-1,4-dioxo-1,4-dihydronaphthalen-2-yl)(phenyl)methyl)-1,4-dioxo-1,4-dihydronaphthalen-2-olate

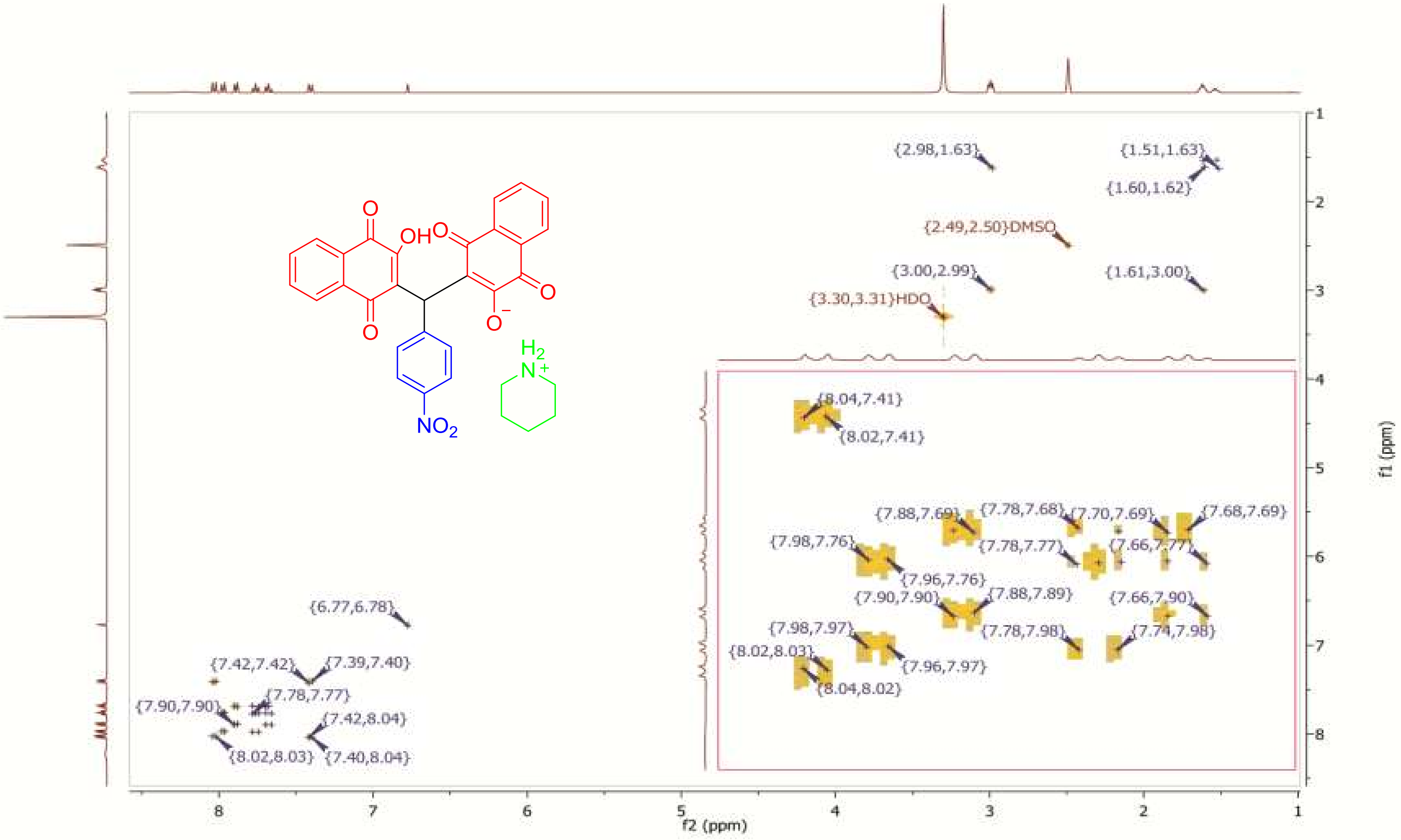

^{1}H^{1}H, COSY-NMR spectrum of piperidin-1-ium 3-((3-hydroxy-1,4-dioxo-1,4-dihydronaphthalen-2-yl)(phenyl)methyl)-1,4-dioxo-1,4-dihydronaphthalen-2-olate

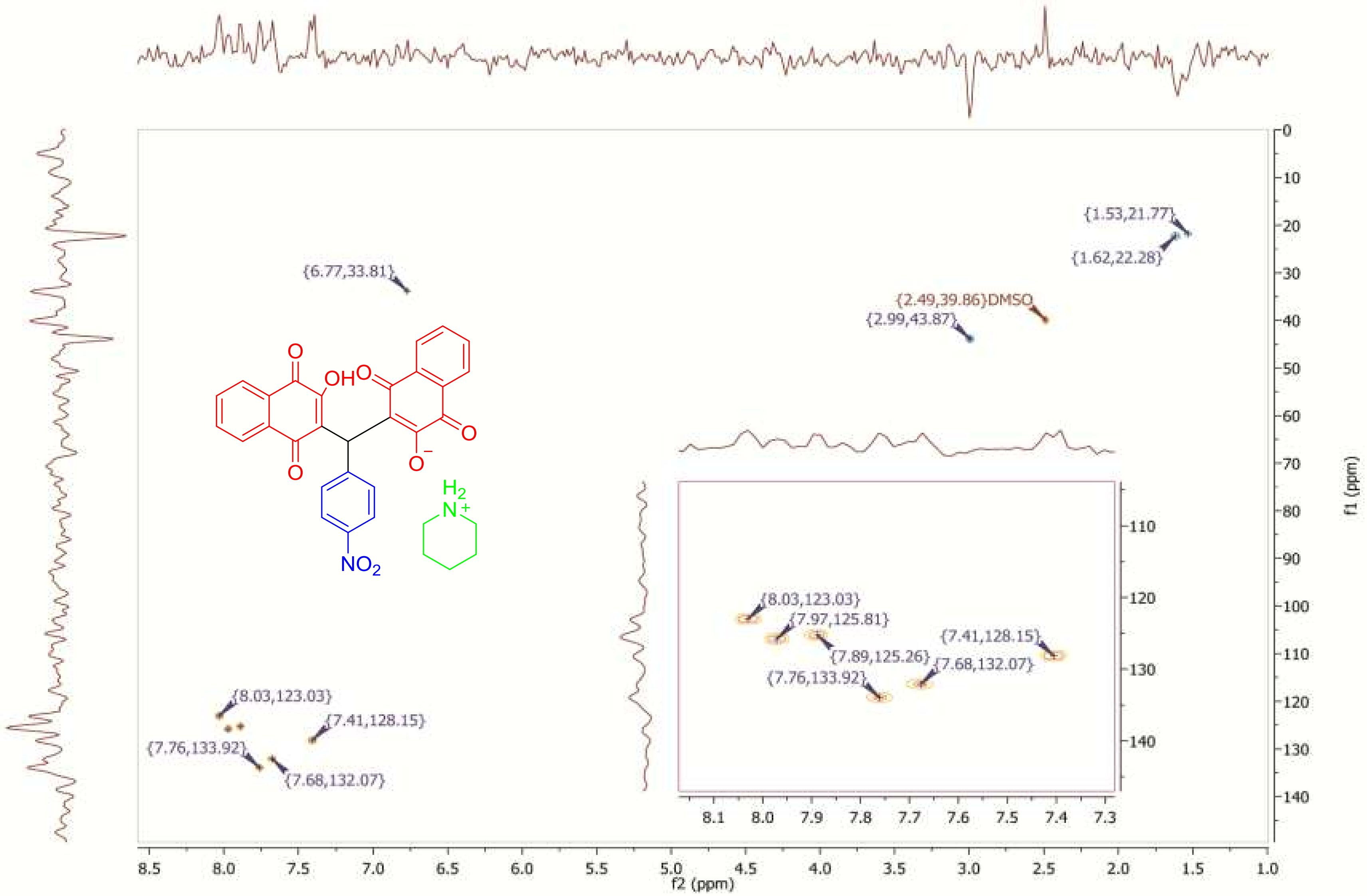

$^1H^{13}C$, HSQC-NMR spectrum of piperidin-1-ium 3-((3-hydroxy-1,4-dioxo-1,4-dihydronaphthalen-2-yl)(phenyl)methyl)-1,4-dioxo-1,4-dihydronaphthalen-2-olate

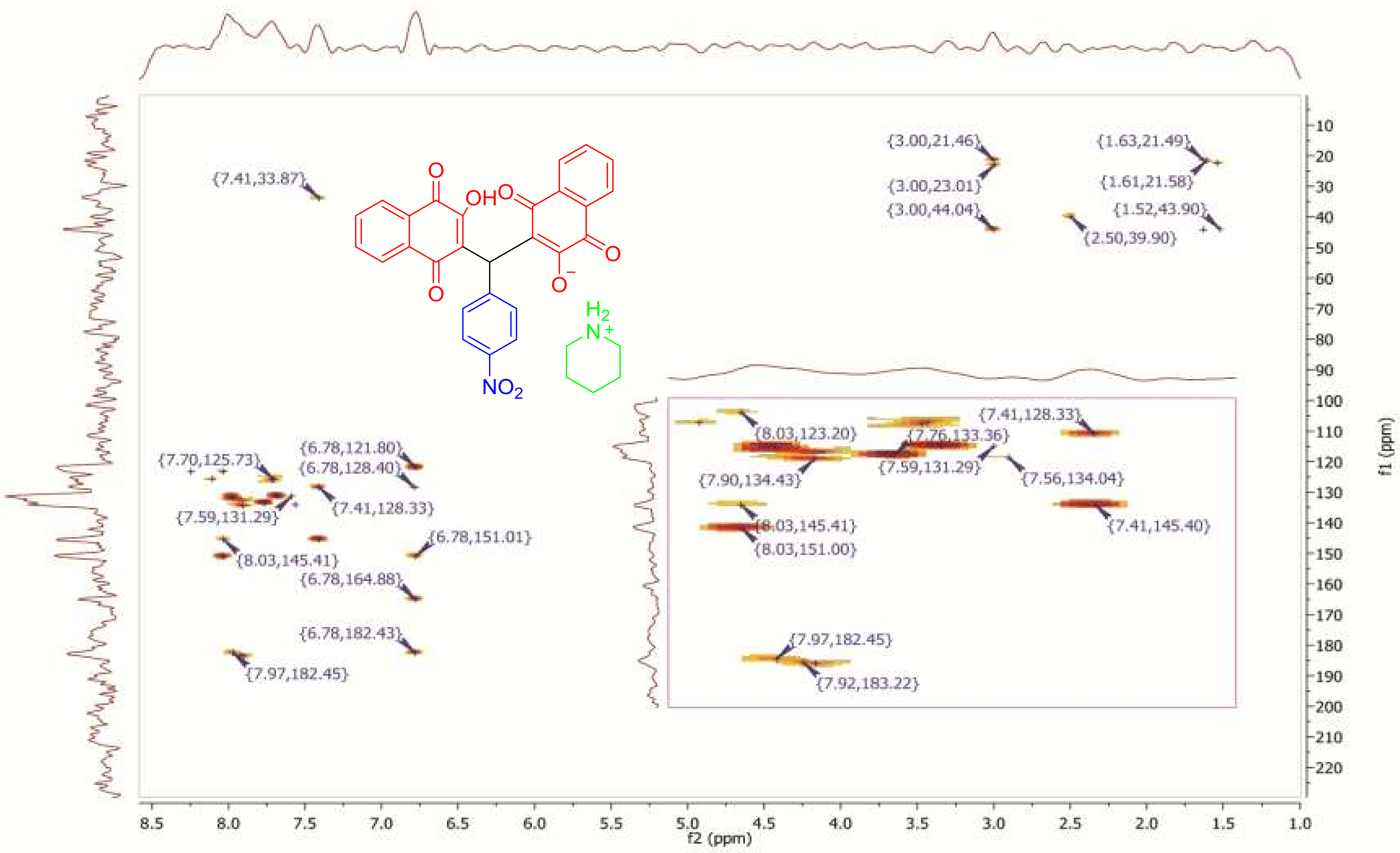

$^{1}H^{13}C$, HMBC-NMR spectrum of piperidin-1-ium 3-((3-hydroxy-1,4-dioxo-1,4-dihydronaphthalen-2-yl)(phenyl)methyl)-1,4-dioxo-1,4-dihydronaphthalen-2-olate

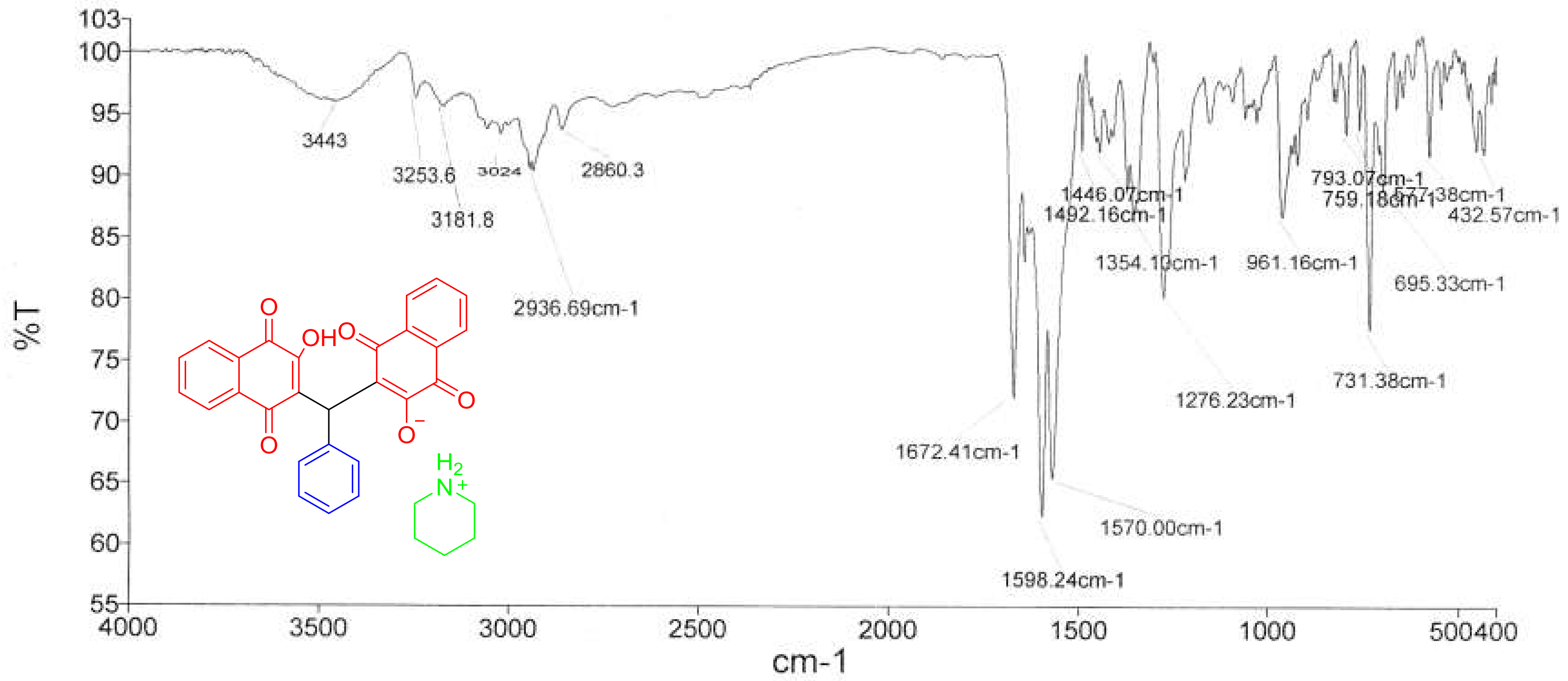

FT-IR spectrum of piperidin-1-ium 3-((3-hydroxy-1,4-dioxo-1,4-dihydronaphthalen-2-yl)(phenyl)methyl)-1,4-dioxo-1,4-dihydronaphthalen-2-olate

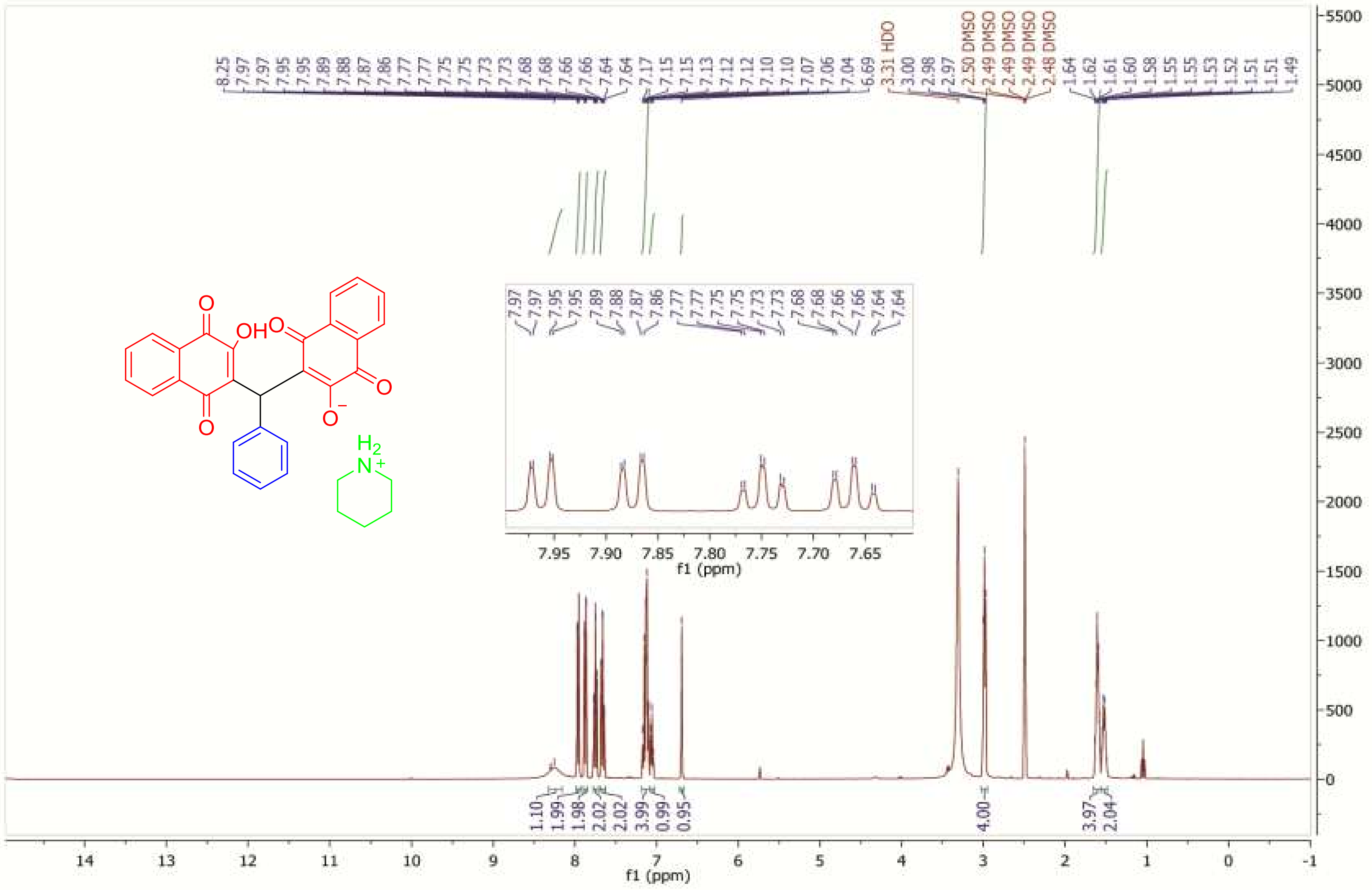

^{1}H NMR spectrum of piperidin-1-ium 3-((3-hydroxy-1,4-dioxo-1,4-dihydronaphthalen-2-yl)(phenyl)methyl)-1,4-dioxo-1,4-dihydronaphthalen**-2-olate**

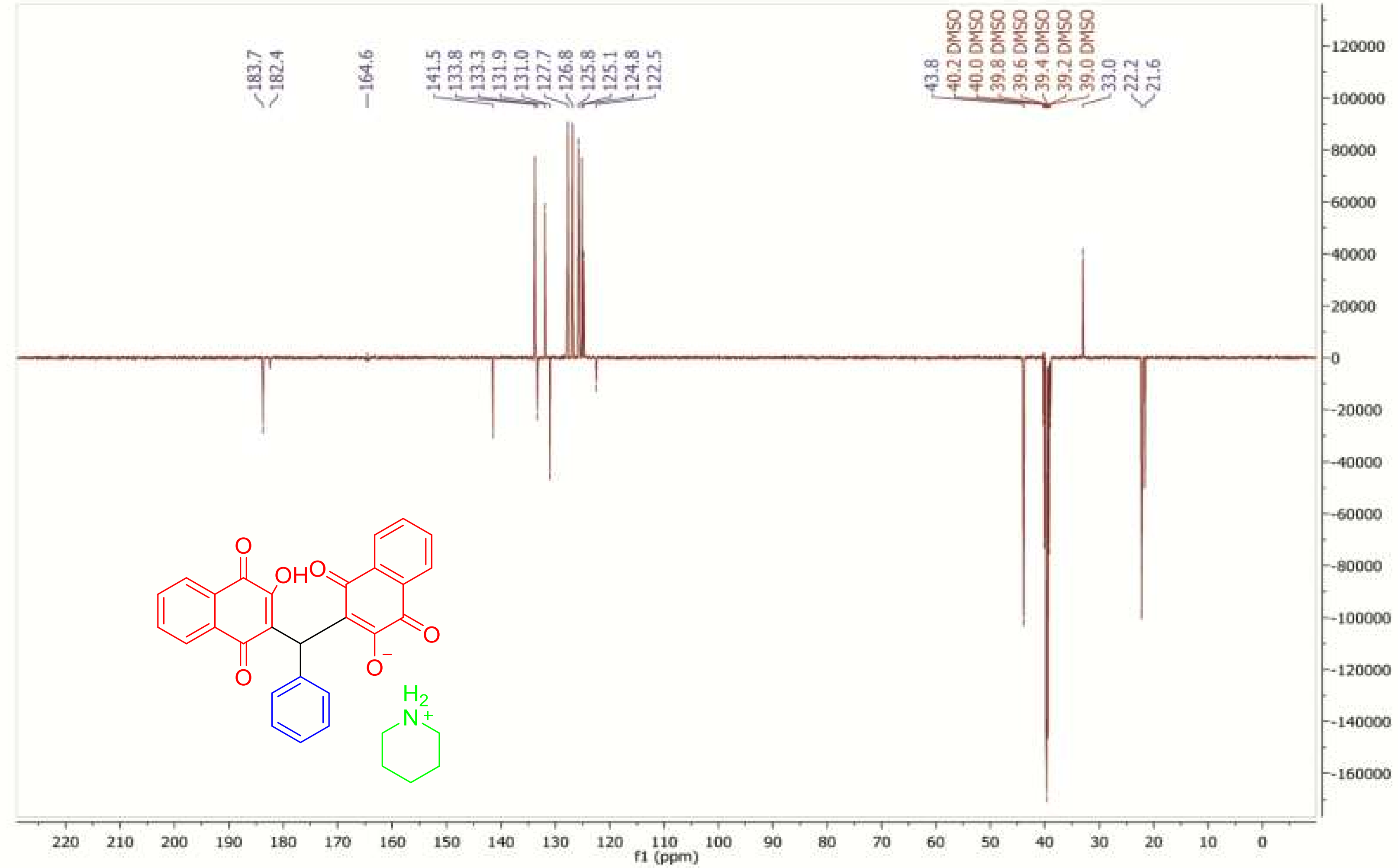

^{13}C NMR (DEPT-135) spectrum of piperidin-1-ium 3-((3-hydroxy-1,4-dioxo-1,4-dihydronaphthalen-2-yl)(phenyl)methyl)-1,4-dioxo-1,4-dihydronaphthalen-2-olate

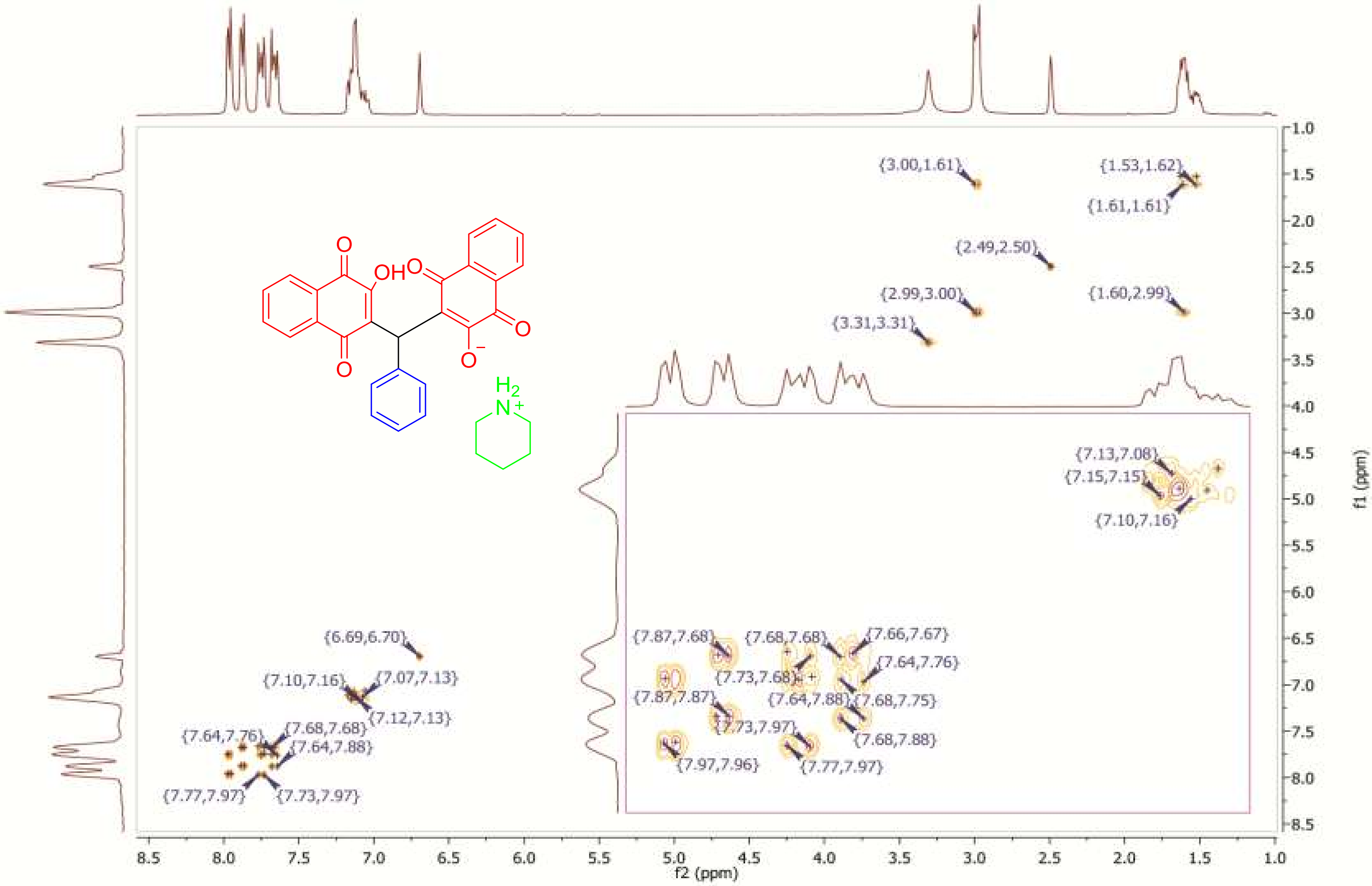

^{1}H^{1}H, COSY-NMR spectrum of piperidin-1-ium 3-((3-hydroxy-1,4-dioxo-1,4-dihydronaphthalen-2-yl)(phenyl)methyl)-1,4-dioxo-1,4-dihydronaphthalen-2-olate

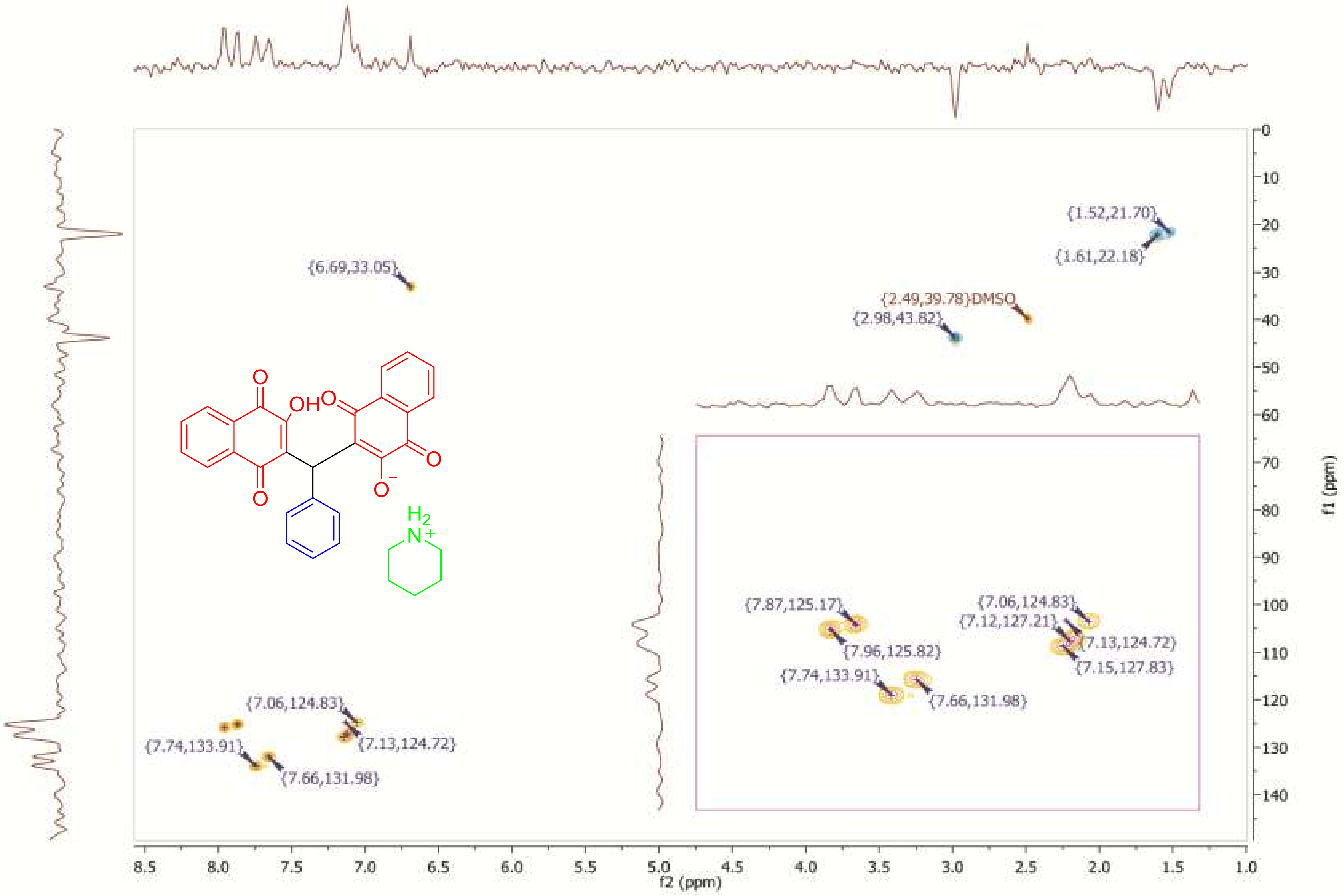

$^{1}H^{13}C$, HSQC-NMR spectrum piperidin-1-ium 3-((3-hydroxy-1,4-dioxo-1,4-dihydronaphthalen-2-yl)(phenyl)methyl)-1,4-dioxo-1,4-dihydronaphthalen-2-olate

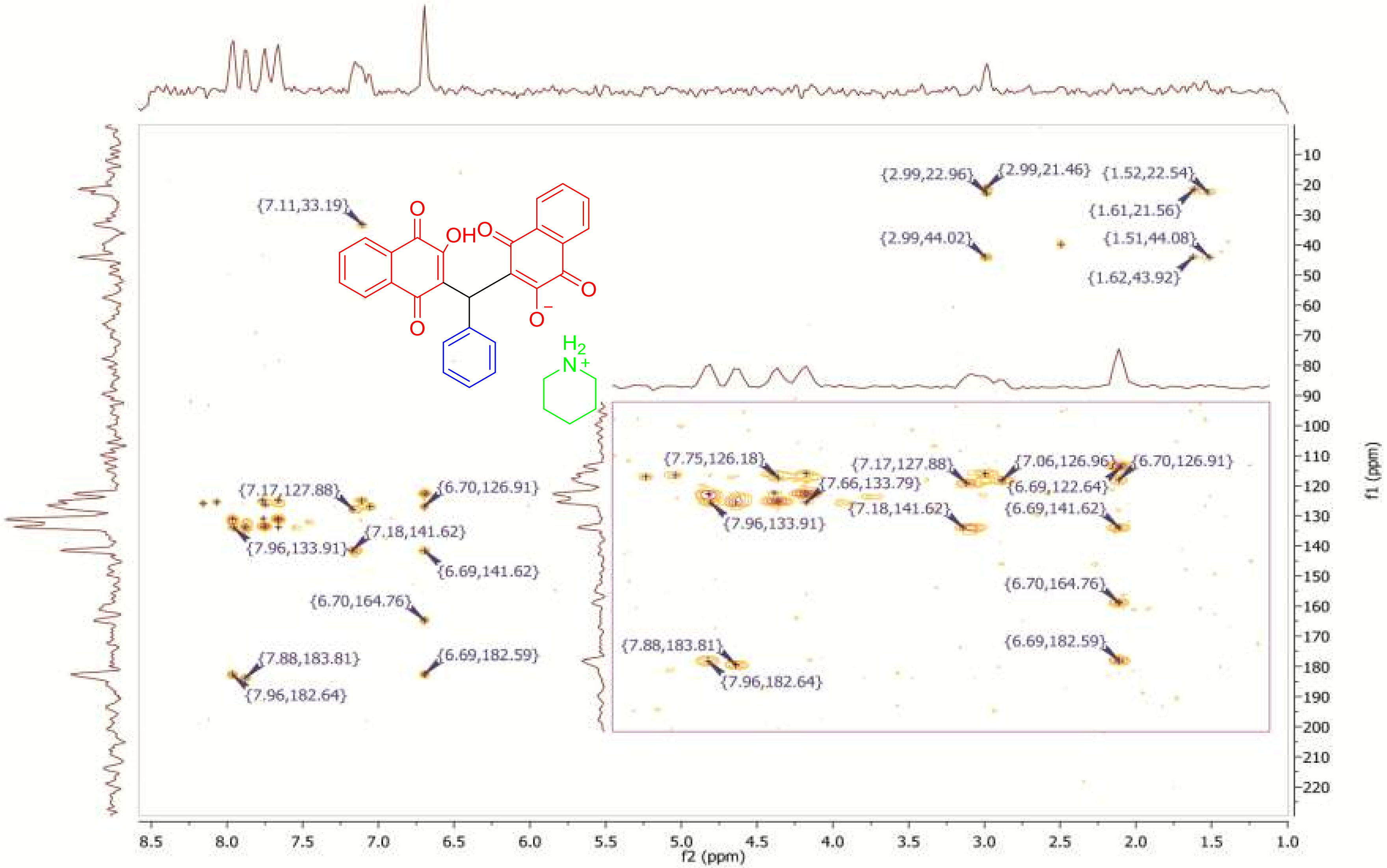

^{1}H^{13}C, HMBC-NMR spectrum of piperidin-1-ium 3-((3-hydroxy-1,4-dioxo-1,4-dihydronaphthalen-2-yl)(phenyl)methyl)-1,4-dioxo-1,4-dihydronaphthalen-2-olate**

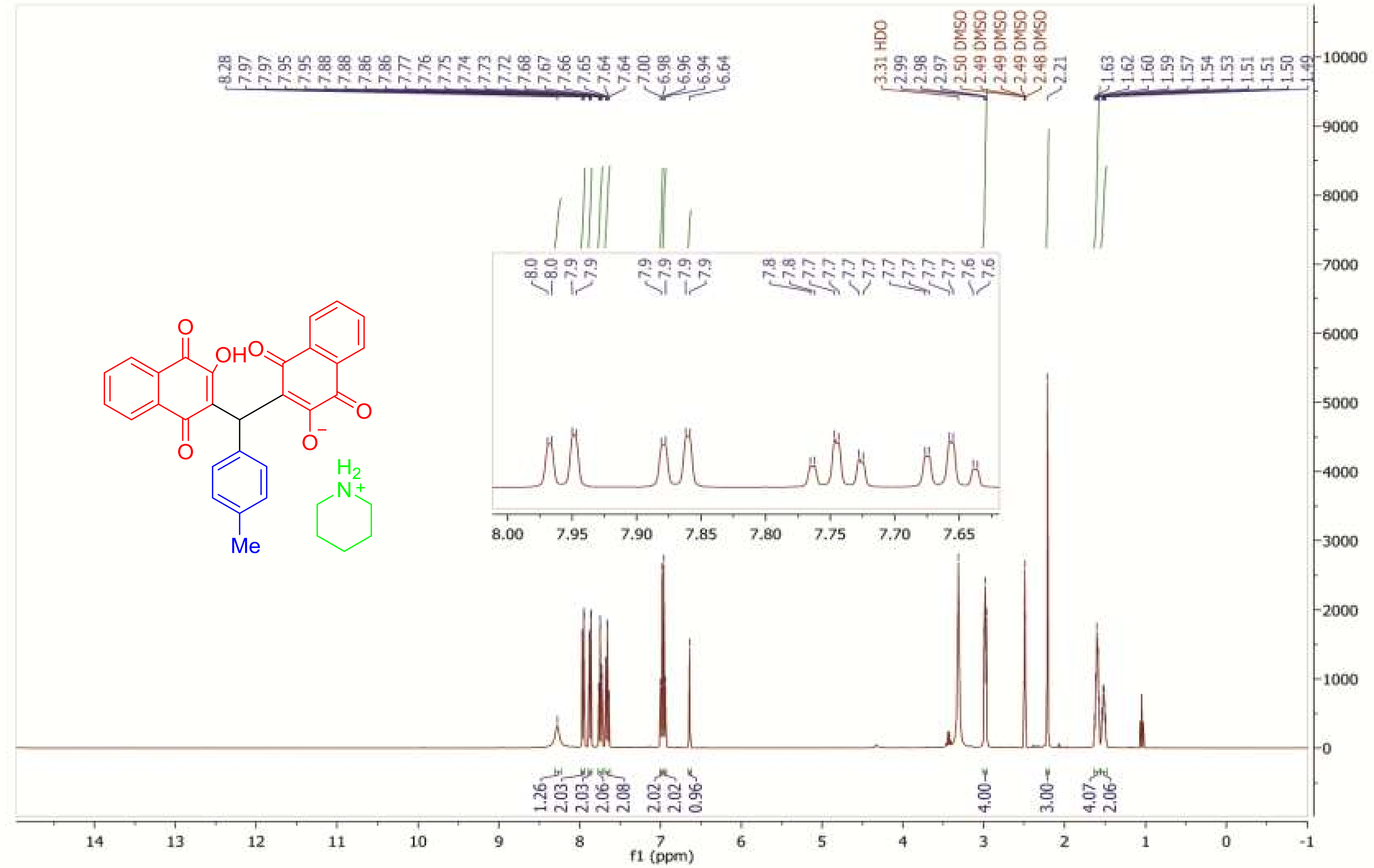

^{1}H NMR spectrum of piperidin-1-ium 3-((3-hydroxy-1,4-dioxo-1,4-dihydronaphthalen-2-yl)(p-tolyl)methyl)-1,4-dioxo-1,4-dihydronaphthalen-2-olate

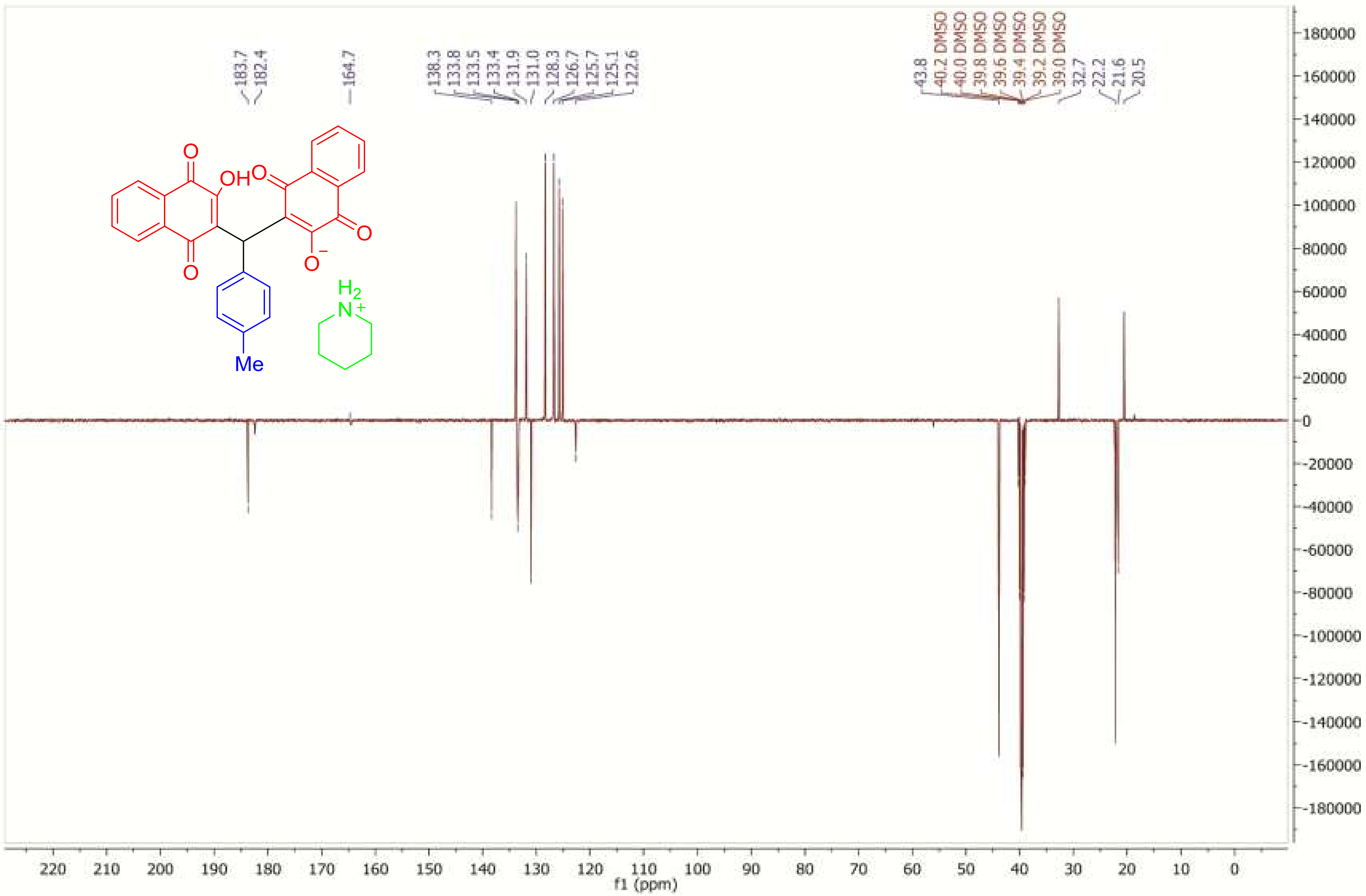

^{13}C NMR (DEPT-135) spectrum of piperidin-1-ium 3-((3-hydroxy-1,4-dioxo-1,4-dihydronaphthalen-2-yl)(p-tolyl)methyl)-1,4-dioxo-1,4-dihydronaphthalen-2-olate

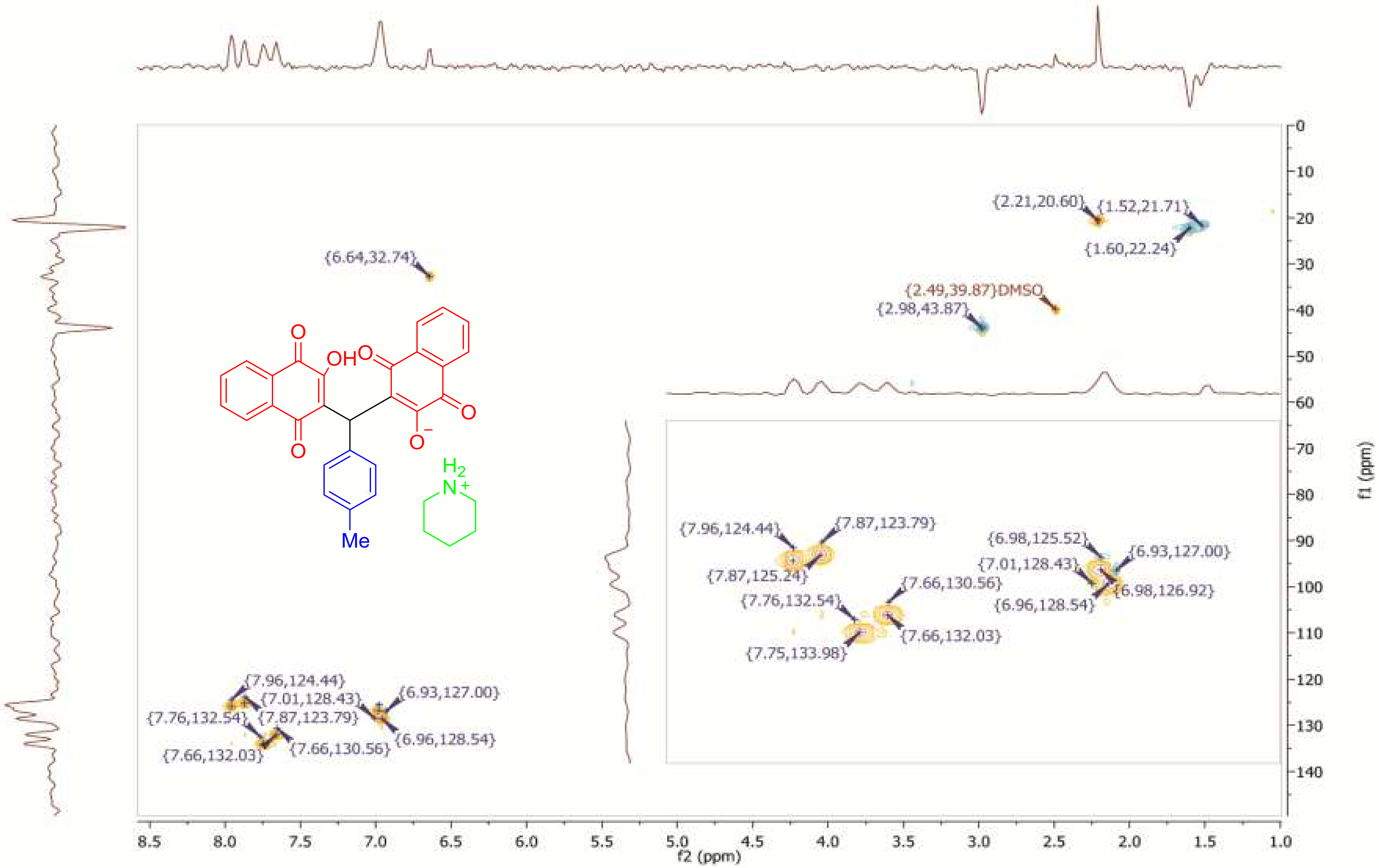

$^{1}H^{13}C$, HSQC-NMR spectrum piperidin-1-ium 3-((3-hydroxy-1,4-dioxo-1,4-dihydronaphthalen-2-yl)(p-tolyl)methyl)-1,4-dioxo-1,4-dihydronaphthalen-2-olate

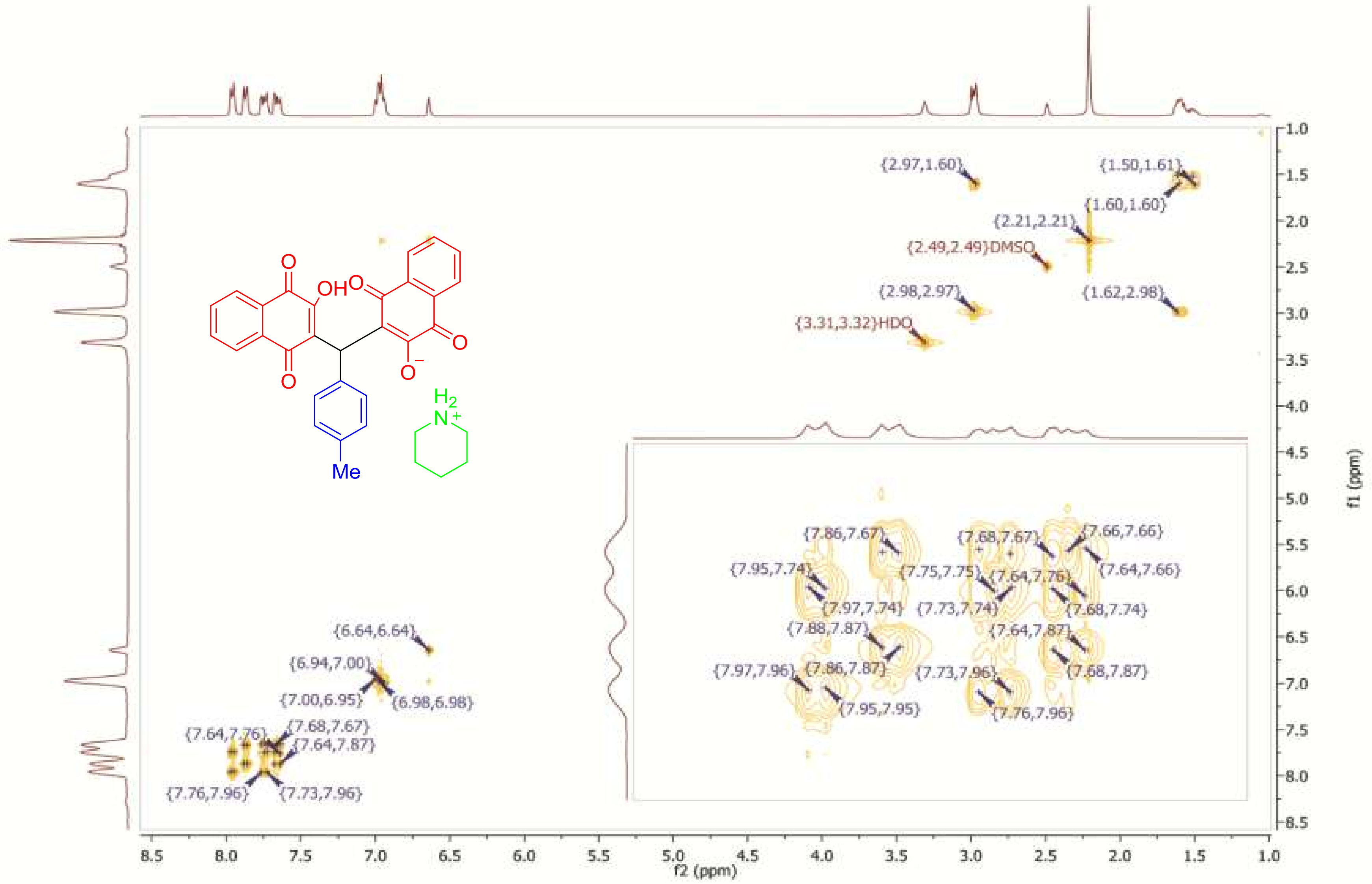

^{1}H^{1}H, COSY-NMR spectrum of piperidin-1-ium 3-((3-hydroxy-1,4-dioxo-1,4-dihydronaphthalen-2-yl)(p-tolyl)methyl)-1,4-dioxo-1,4-dihydronaphthalen-2-olate

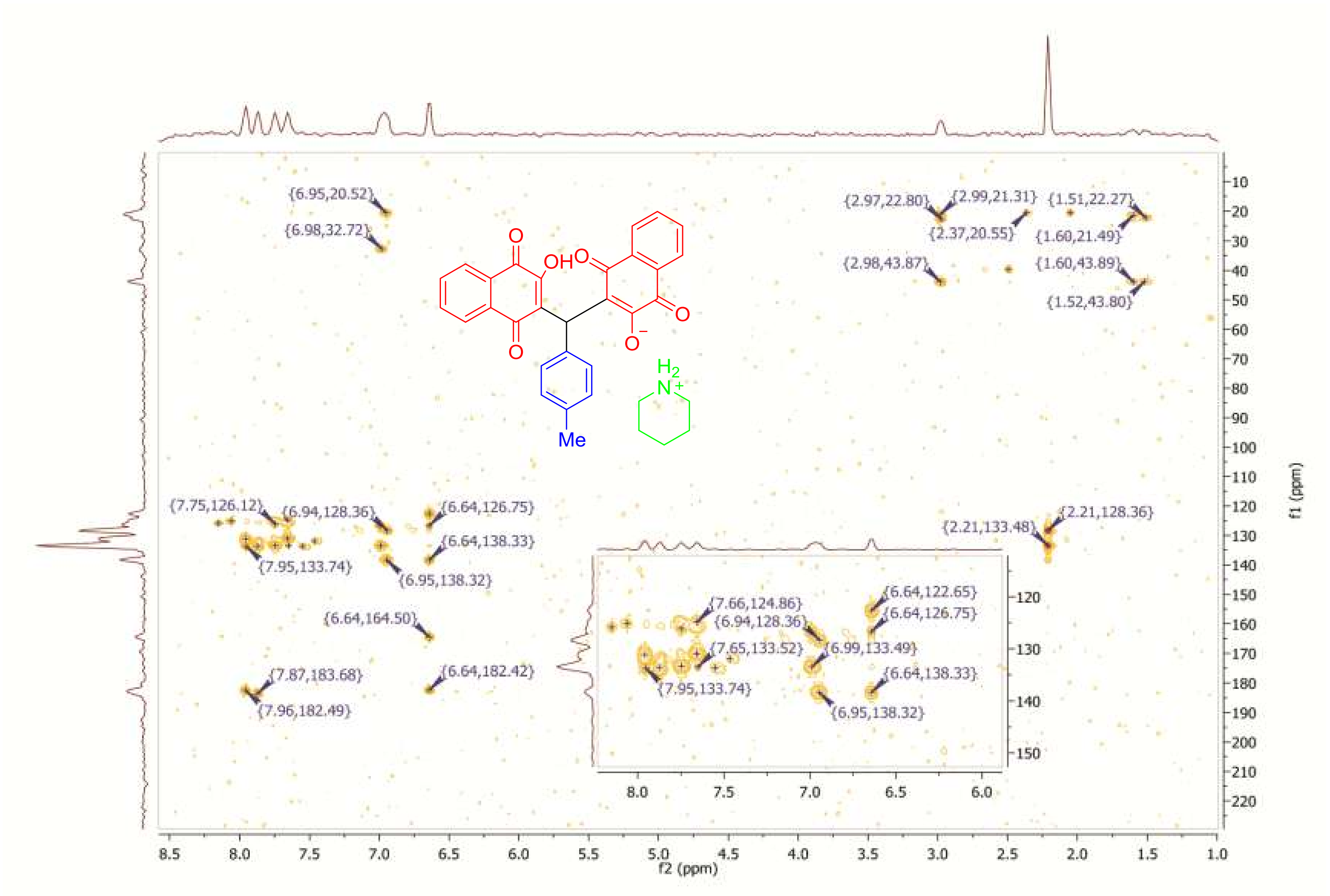

^{1}H^{13}C, HMBC-NMR spectrum of piperidin-1-ium 3-((3-hydroxy-1,4-dioxo-1,4-dihydronaphthalen-2-yl)(p-tolyl)methyl)-1,4-dioxo-1,4-dihydronaphthalen-2-olate

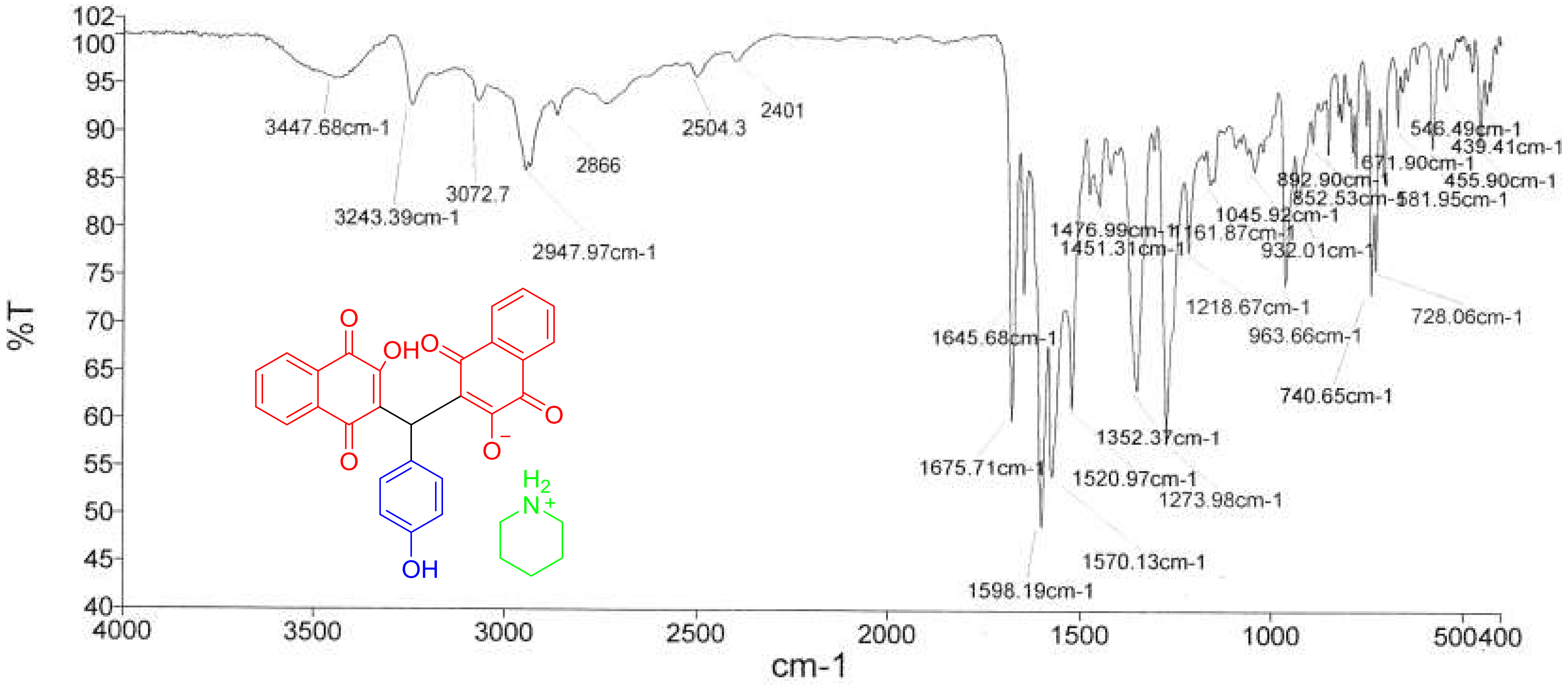

FT-IR spectrum of piperidin-1-ium 3-((3-hydroxy-1,4-dioxo-1,4-dihydronaphthalen-2-yl)(4-hydroxyphenyl)methyl)-1,4-dioxo-1,4-dihydronaphthalen-2-olate

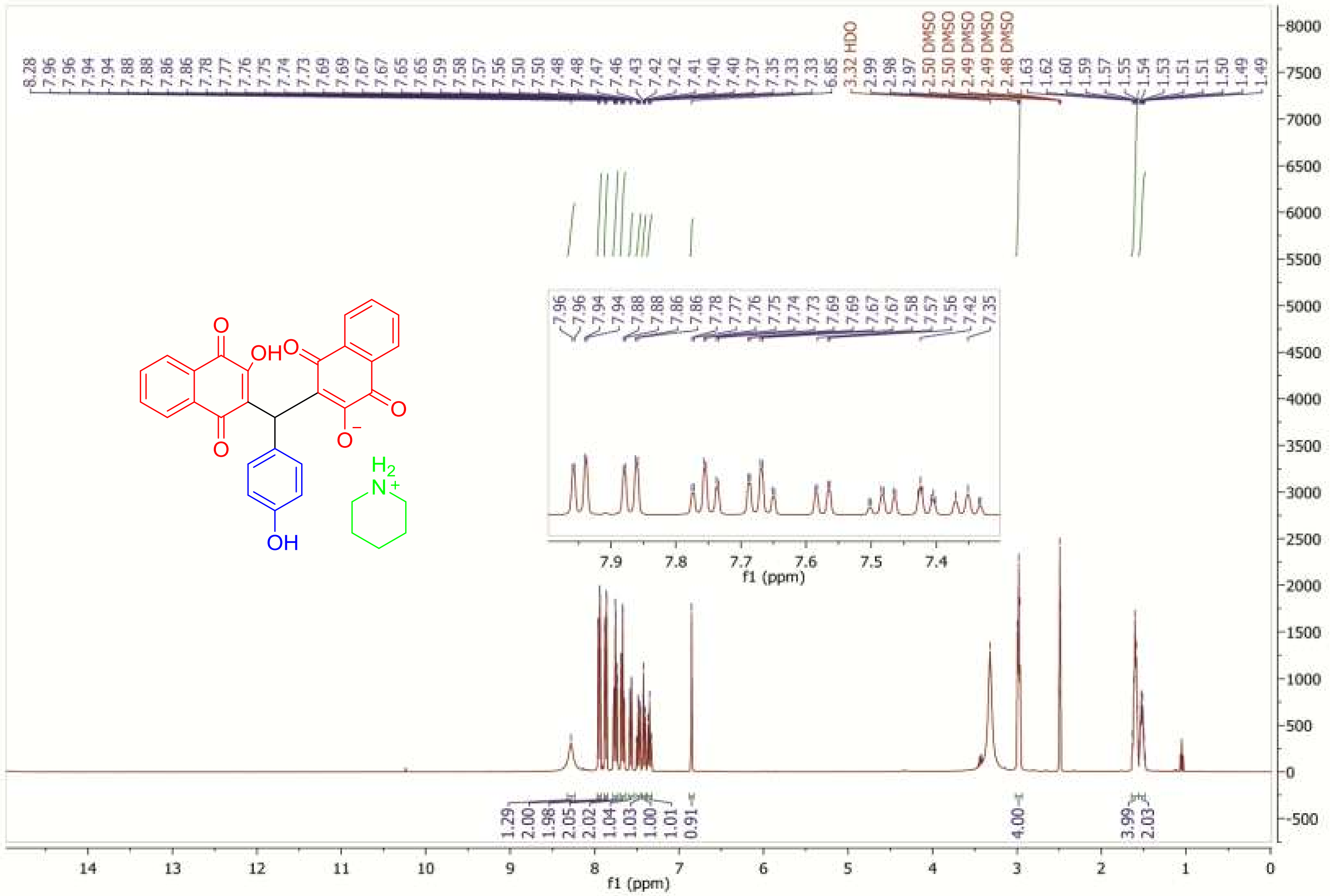

^{1}H NMR spectrum of piperidin-1-ium 3-((3-hydroxy-1,4-dioxo-1,4-dihydronaphthalen-2-yl)(4-hydroxyphenyl)methyl)-1,4-dioxo-1,4-dihydronaphthalen-2-olate

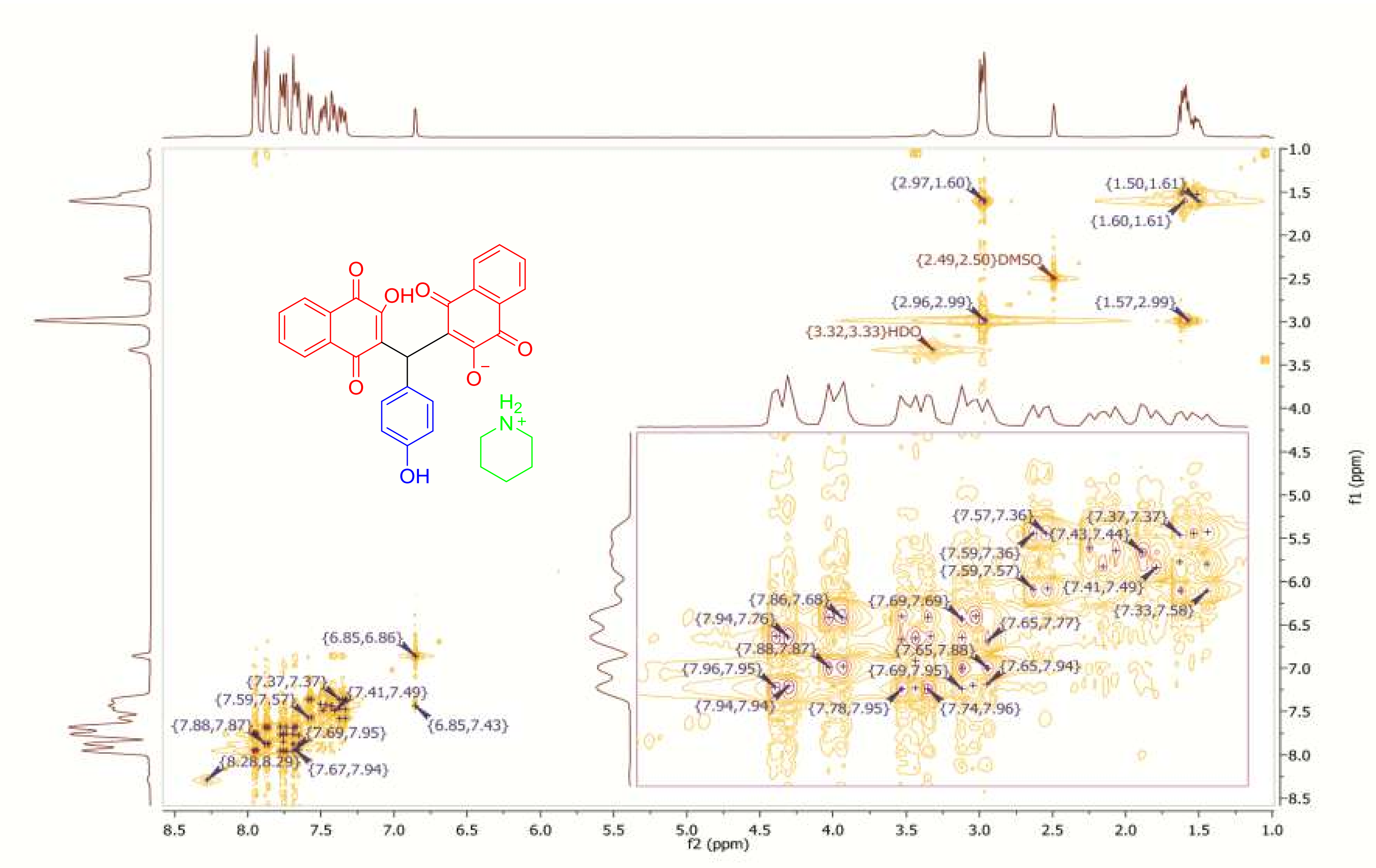

^{1}H^{1}H, COSY-NMR spectrum of piperidin-1-ium 3-((3-hydroxy-1,4-dioxo-1,4-dihydronaphthalen-2-yl)(4-hydroxyphenyl)methyl)-1,4-dioxo-1,4-dihydronaphthalen-2-olate

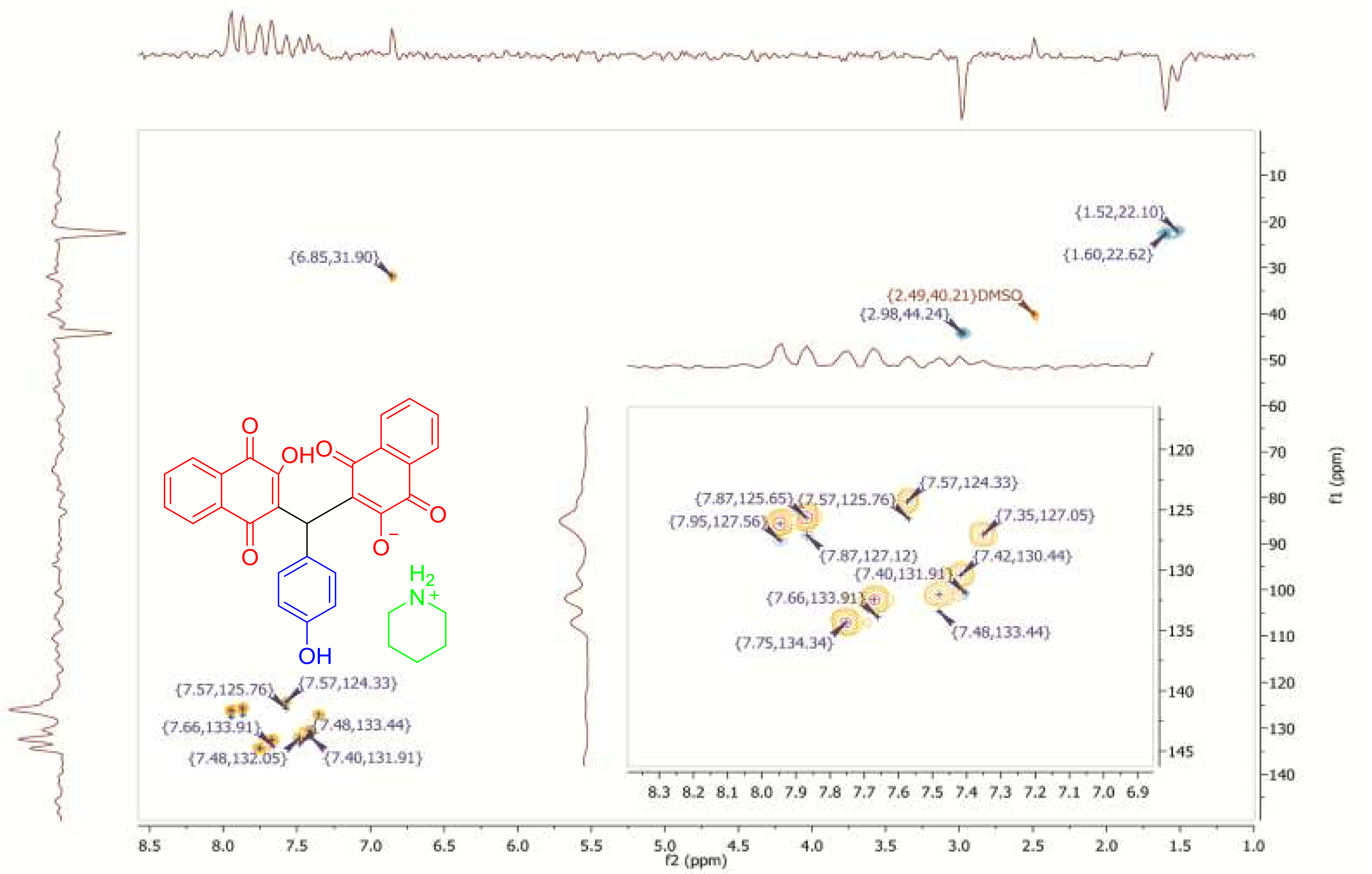

^{1}H^{13}C, HSQC-NMR spectrum piperidin-1-ium 3-((3-hydroxy-1,4-dioxo-1,4-dihydronaphthalen-2-yl)(4-hydroxyphenyl)methyl)-1,4-dioxo-1,4-dihydronaphthalen-2-olate

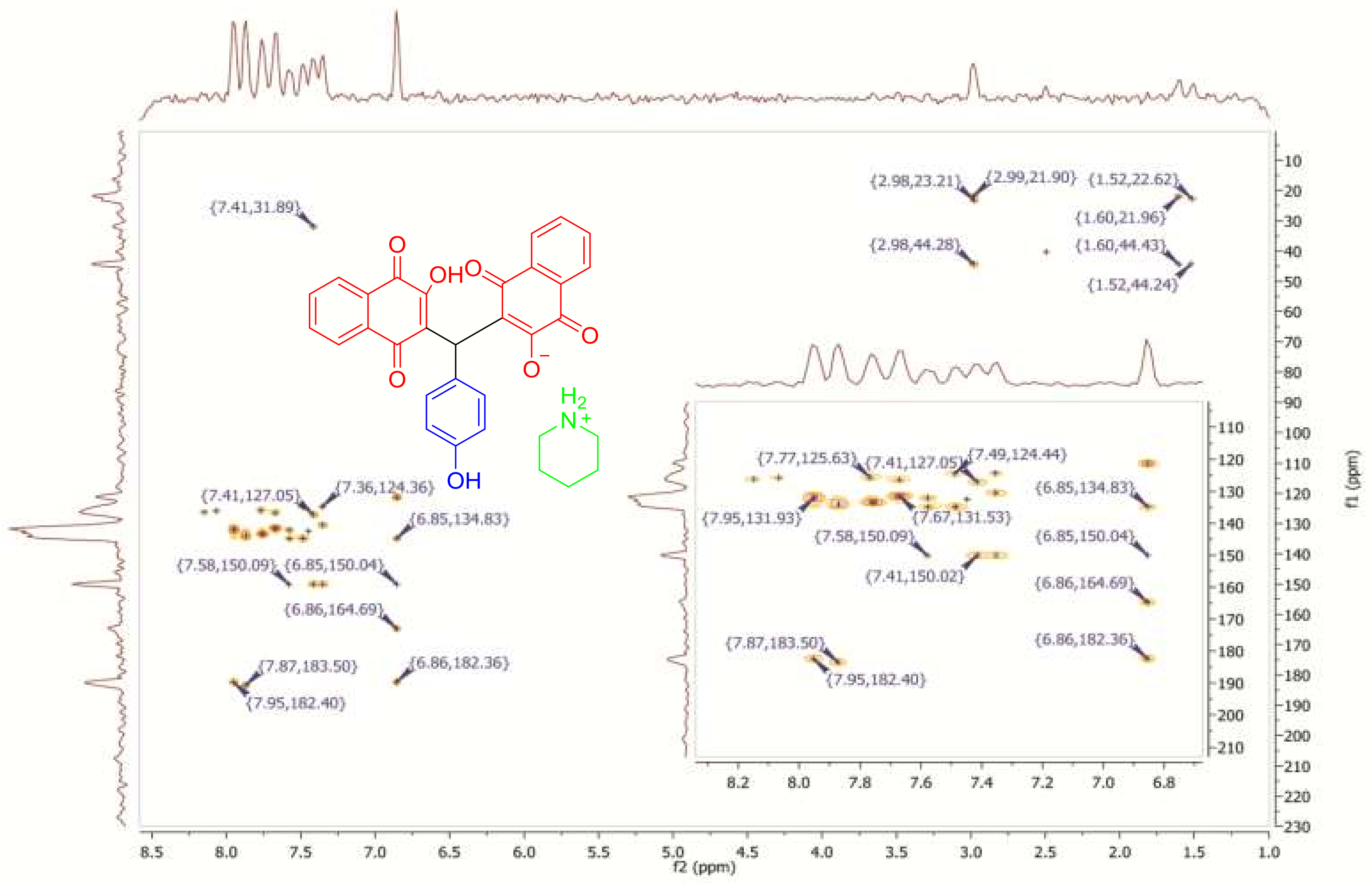

^{1}H^{13}C, HMBC-NMR spectrum of piperidin-1-ium 3-((3-hydroxy-1,4-dioxo-1,4-dihydronaphthalen-2-yl)(4-hydroxyphenyl)methyl)-1,4-dioxo-1,4-dihydronaphthalen-2-olate

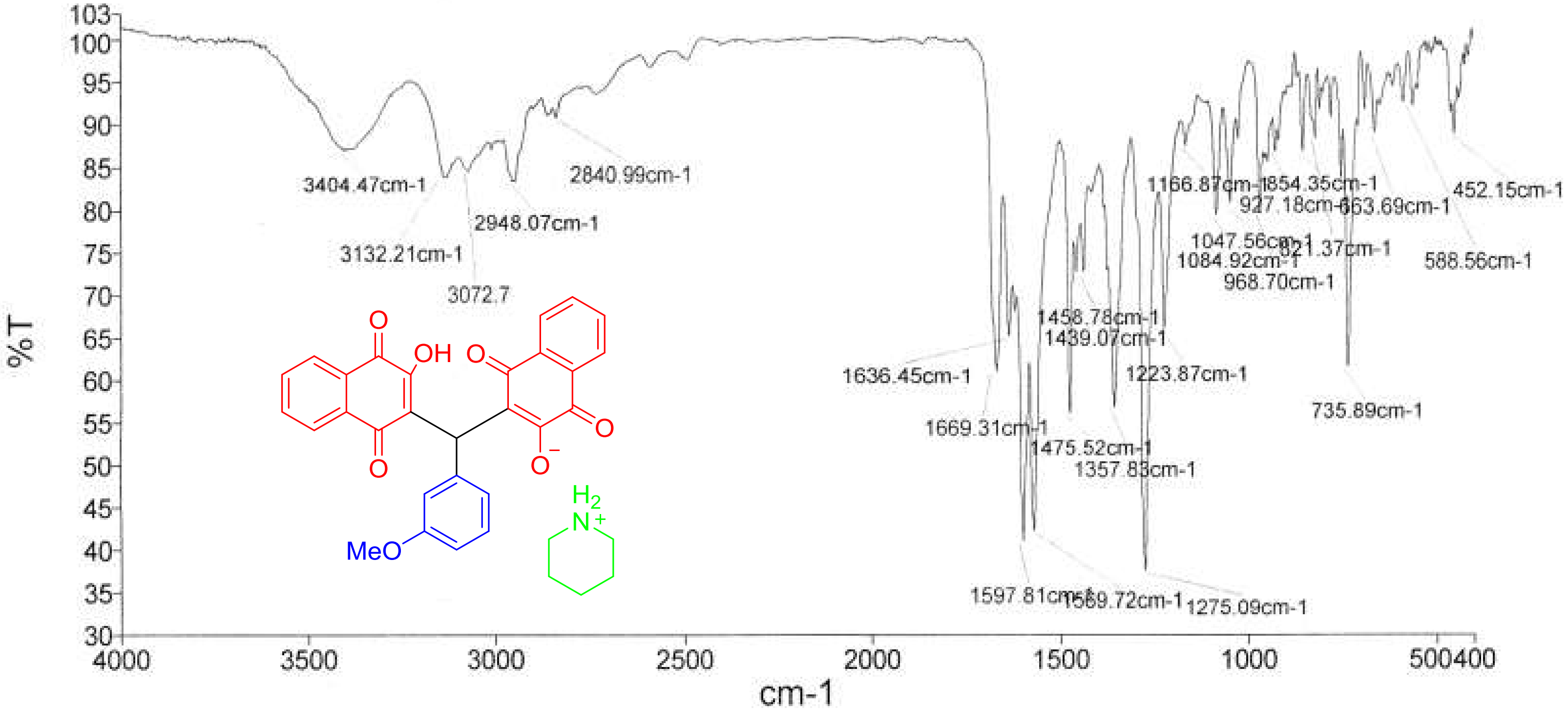

FT-IR spectrum of piperidin-1-ium 3-((3-hydroxy-1,4-dioxo-1,4-dihydronaphthalen-2-yl)(3-methoxyphenyl)methyl)-1,4-dioxo-1,4-dihydronaphthalen-2-olate

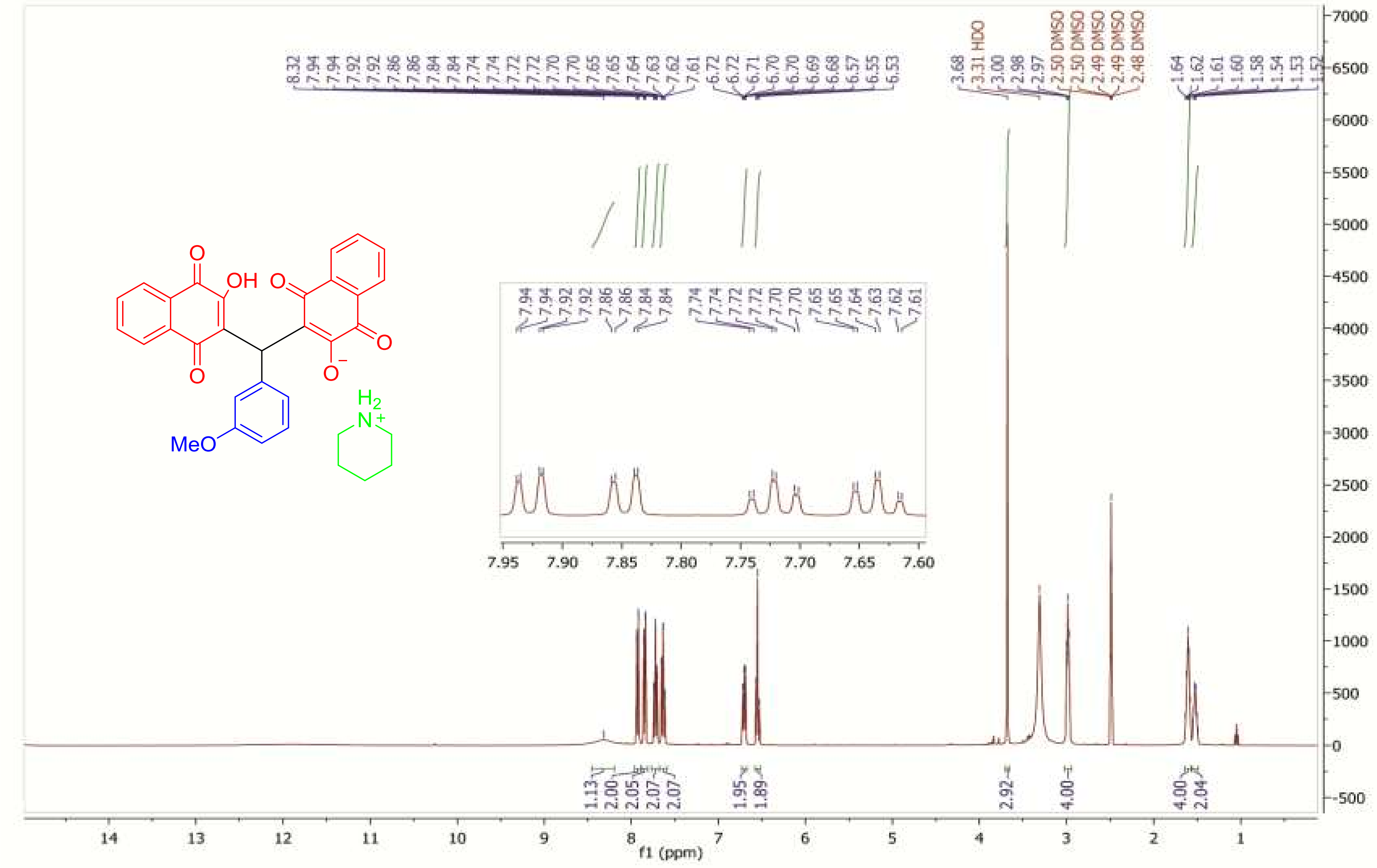

^{1}H NMR spectrum of piperidin-1-ium 3-((3-hydroxy-1,4-dioxo-1,4-dihydronaphthalen-2-yl)(3-methoxyphenyl)methyl)-1,4-dioxo-1,4-dihydronaphthalen-2-olate

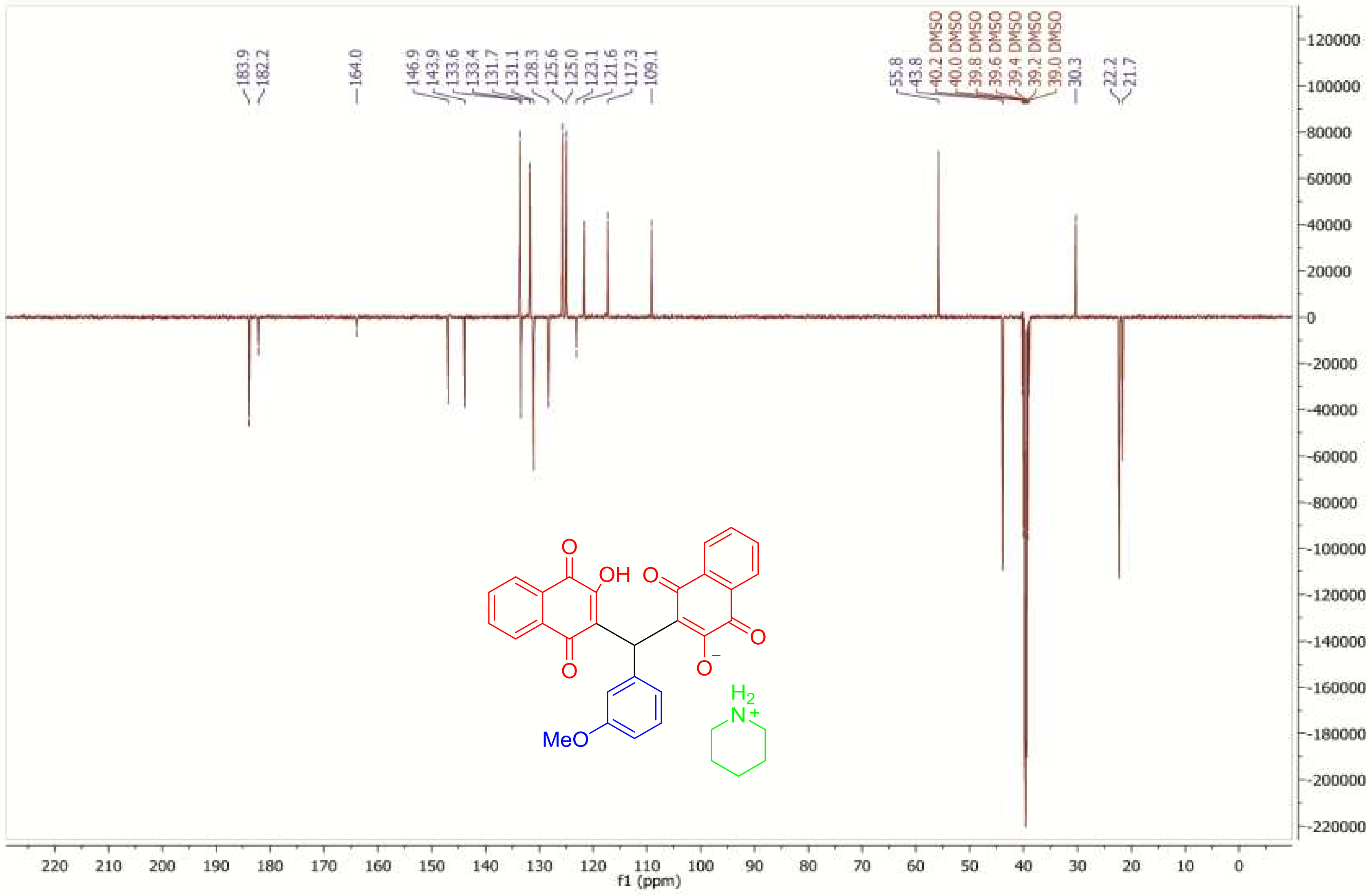

^{13}C NMR (DEPT-135) spectrum of piperidin-1-ium 3-((3-hydroxy-1,4-dioxo-1,4-dihydronaphthalen-2-yl)(3-methoxyphenyl)methyl)-1,4-dioxo-1,4-dihydronaphthalen-2-olate

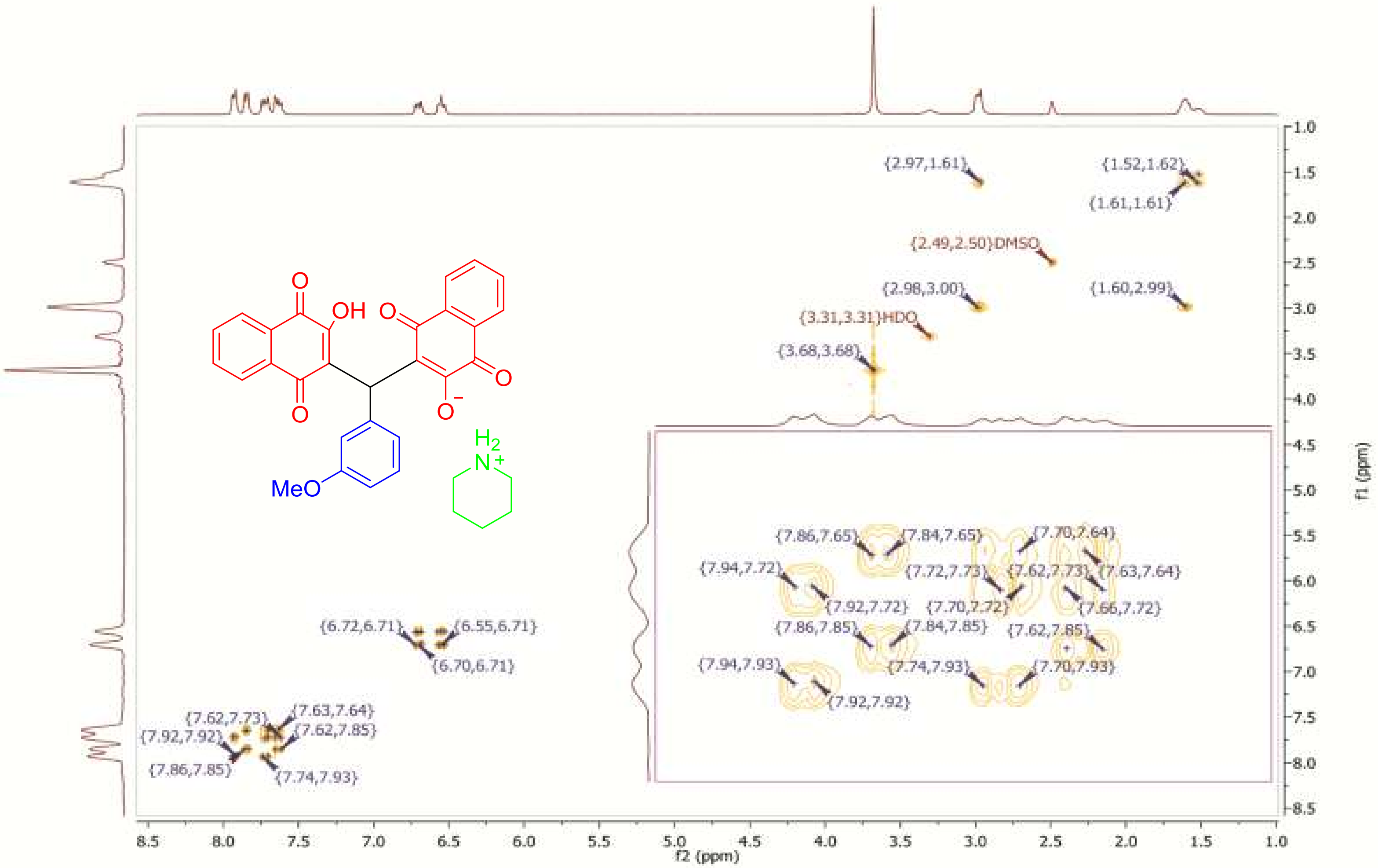

$^{1}H^{1}H$, COSY-NMR spectrum of piperidin-1-ium 3-((3-hydroxy-1,4-dioxo-1,4-dihydronaphthalen-2-yl)(3-methoxyphenyl)methyl)-1,4-dioxo-1,4-dihydronaphthalen-2-olate

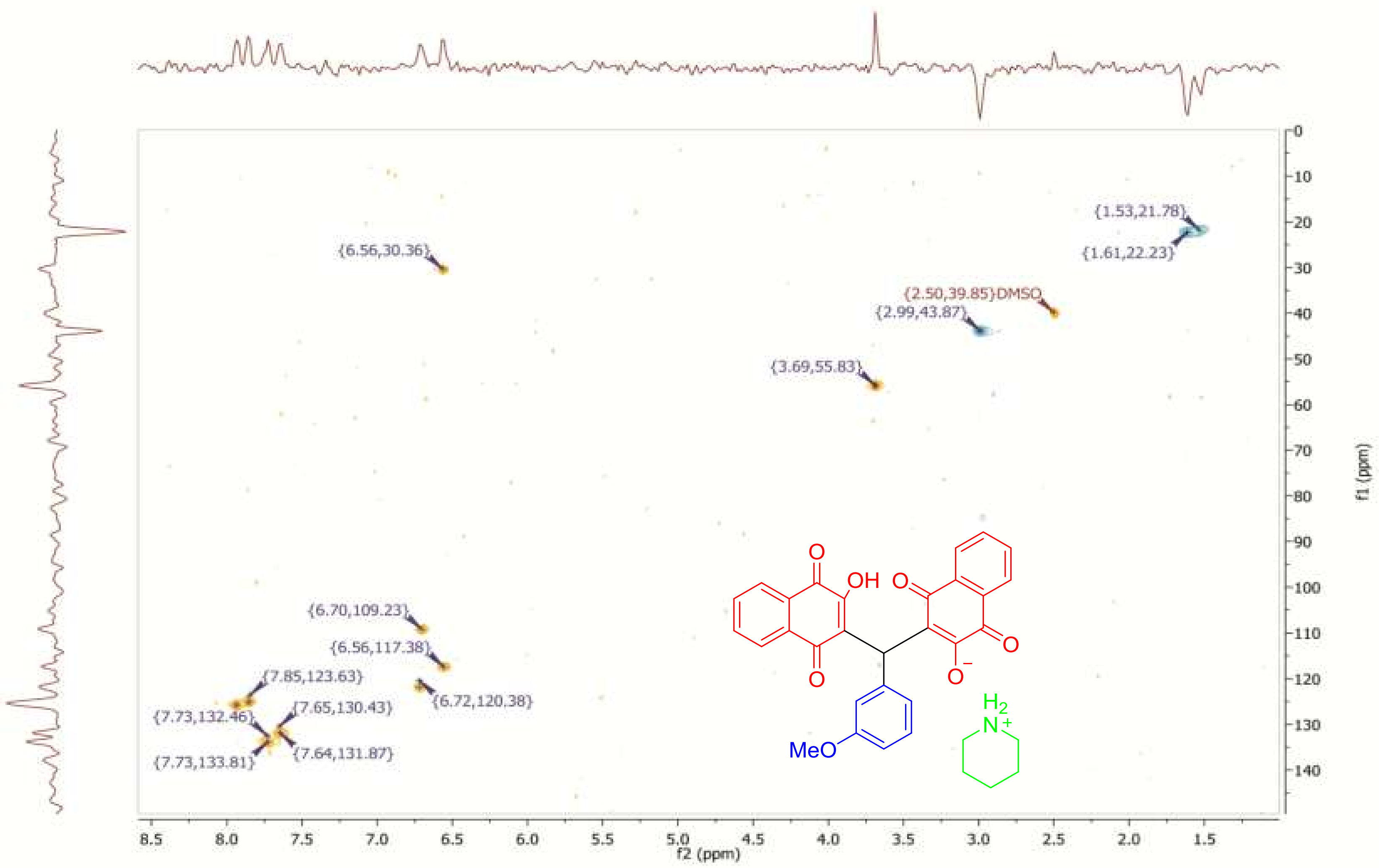

$^{1}H^{13}C$, HSQC-NMR spectrum piperidin-1-ium 3-((3-hydroxy-1,4-dioxo-1,4-dihydronaphthalen-2-yl)(3-methoxyphenyl)methyl)-1,4-dioxo-1,4-dihydronaphthalen-2-olate

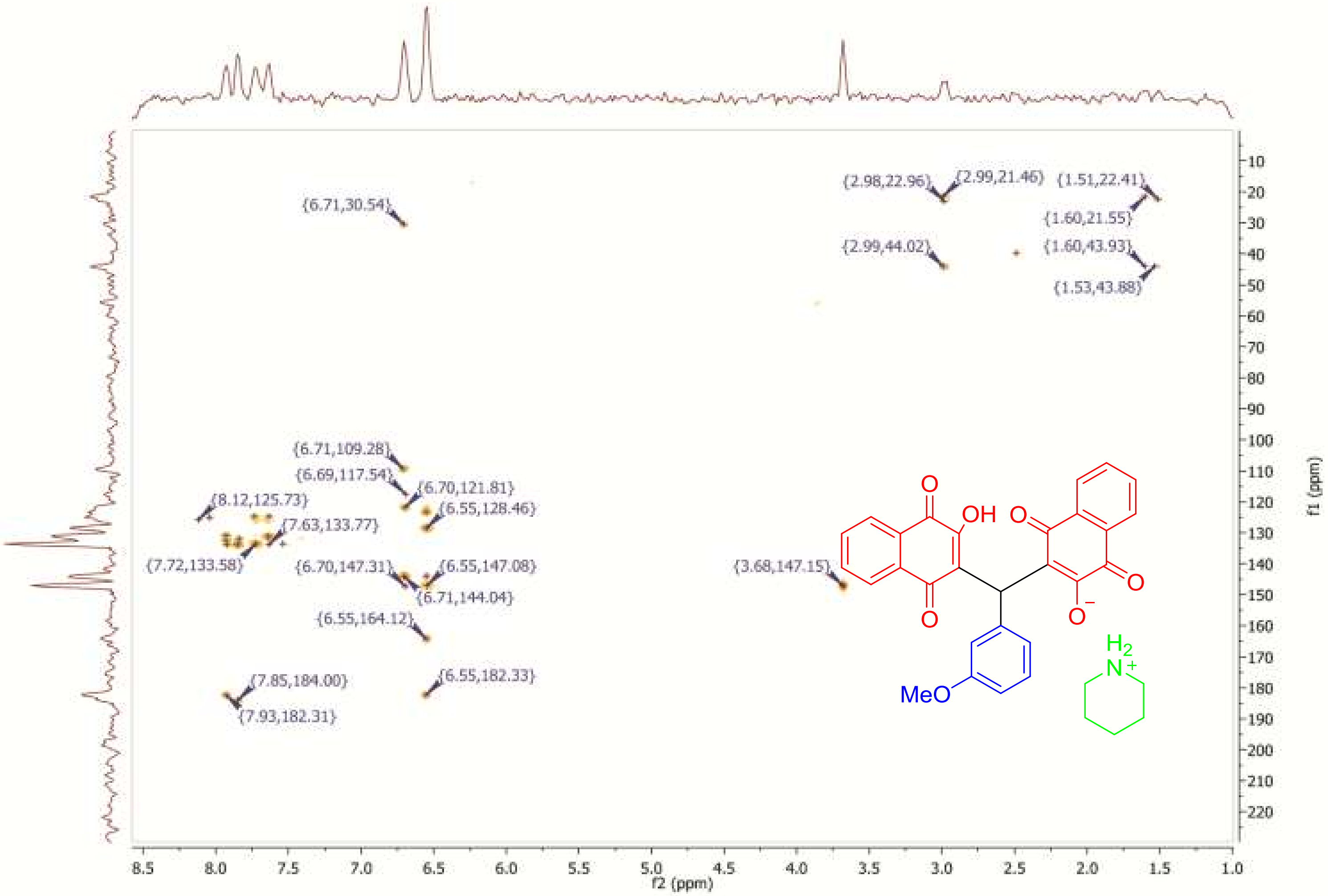

^{1}H^{13}C, HMBC-NMR spectrum of piperidin-1-ium 3-((3-hydroxy-1,4-dioxo-1,4-dihydronaphthalen-2-yl)(3-methoxyphenyl)methyl)-1,4-dioxo-1,4-dihydronaphthalen-2-olate

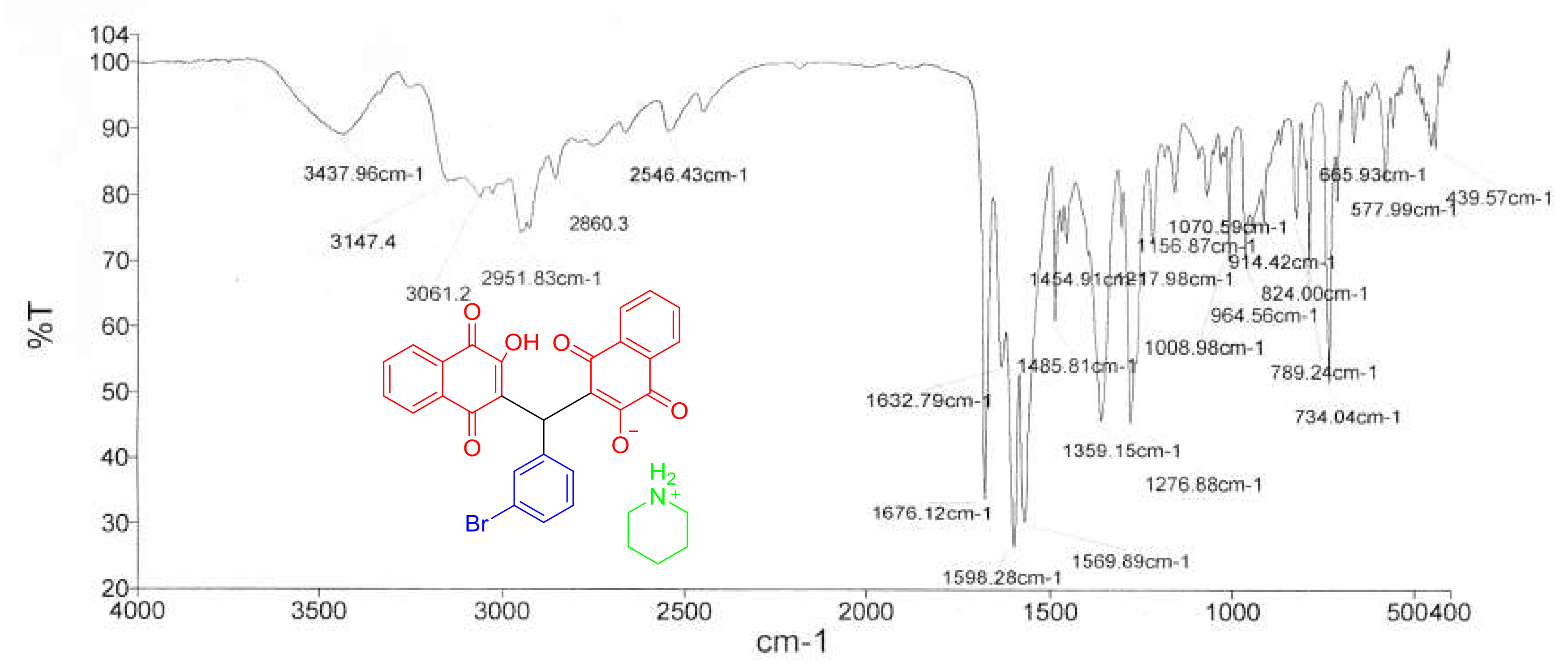

FT-IR spectrum of piperidin-1-ium 3-((3-bromophenyl)(3-hydroxy-1,4-dioxo-1,4-dihydronaphthalen-2-yl)methyl)-1,4-dioxo-1,4-dihydronaphthalen-2-olate

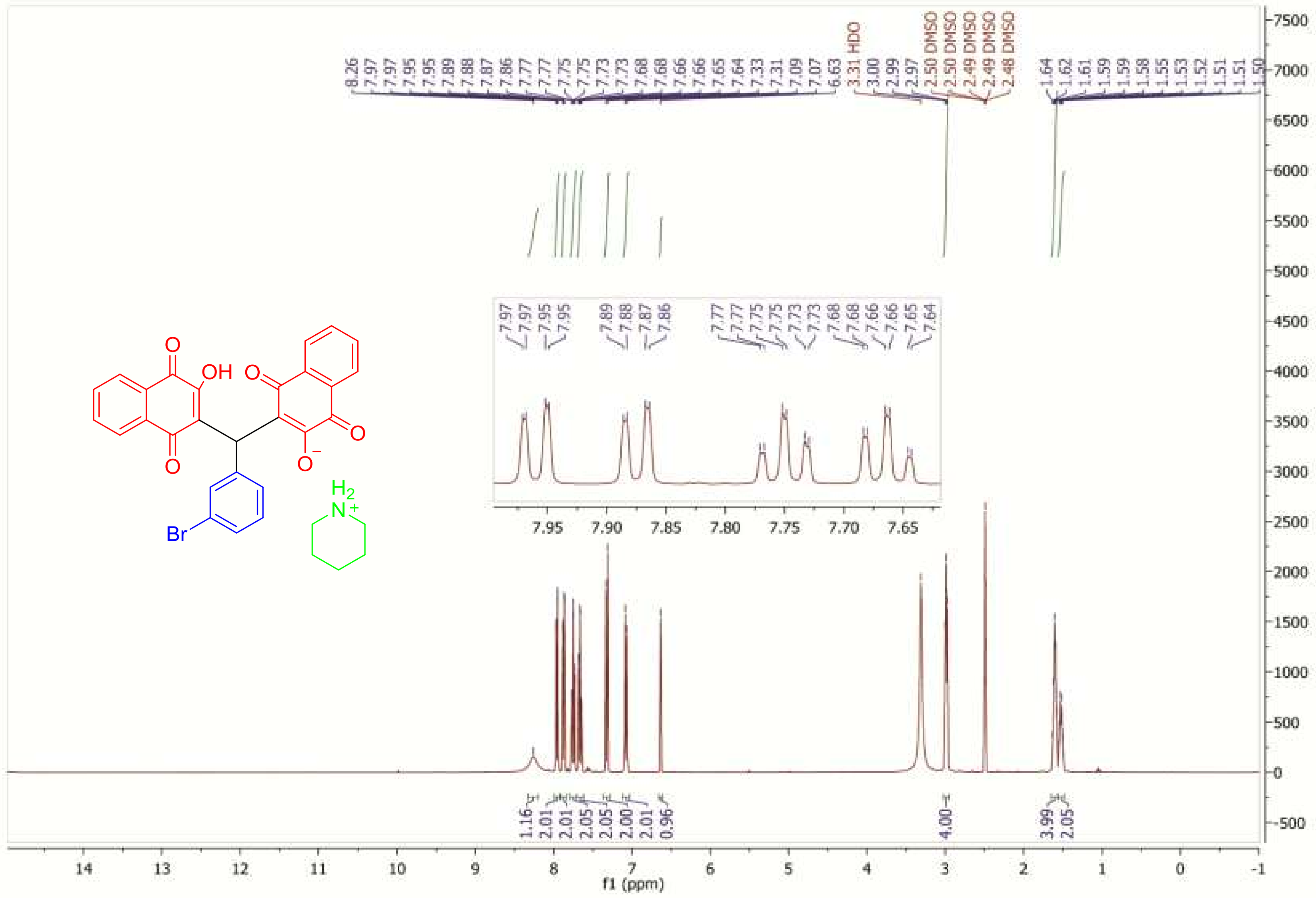

^{1}H NMR spectrum of piperidin-1-ium 3-((3-bromophenyl)(3-hydroxy-1,4-dioxo-1,4-dihydronaphthalen-2-yl)methyl)-1,4-dioxo-1,4-dihydronaphthalen-2-olate

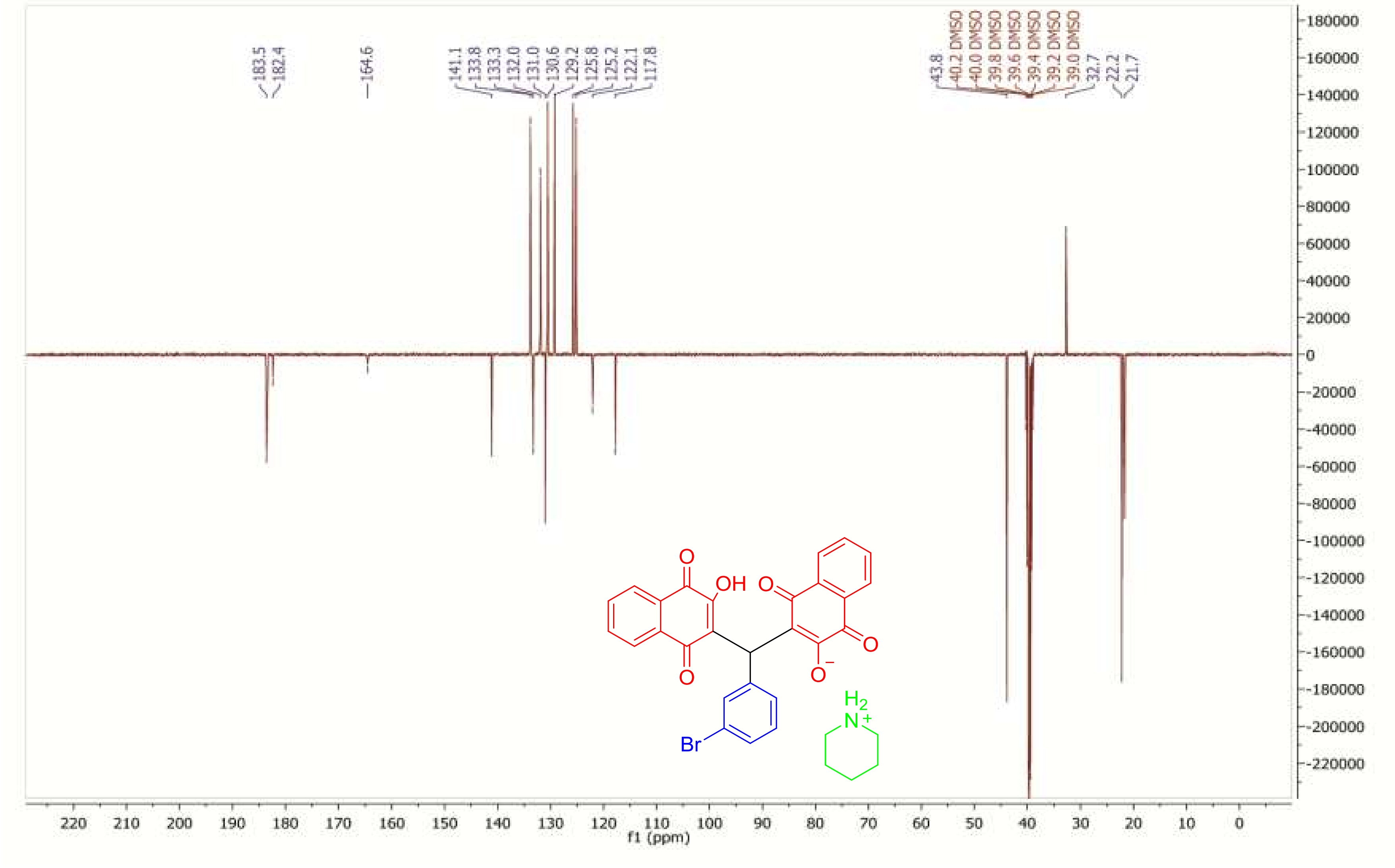

^{13}C NMR (DEPT-135) spectrum of piperidin-1-ium 3-((3-bromophenyl)(3-hydroxy-1,4-dioxo-1,4-dihydronaphthalen-2-yl)methyl)-1,4-dioxo-1,4-dihydronaphthalen-2-olate

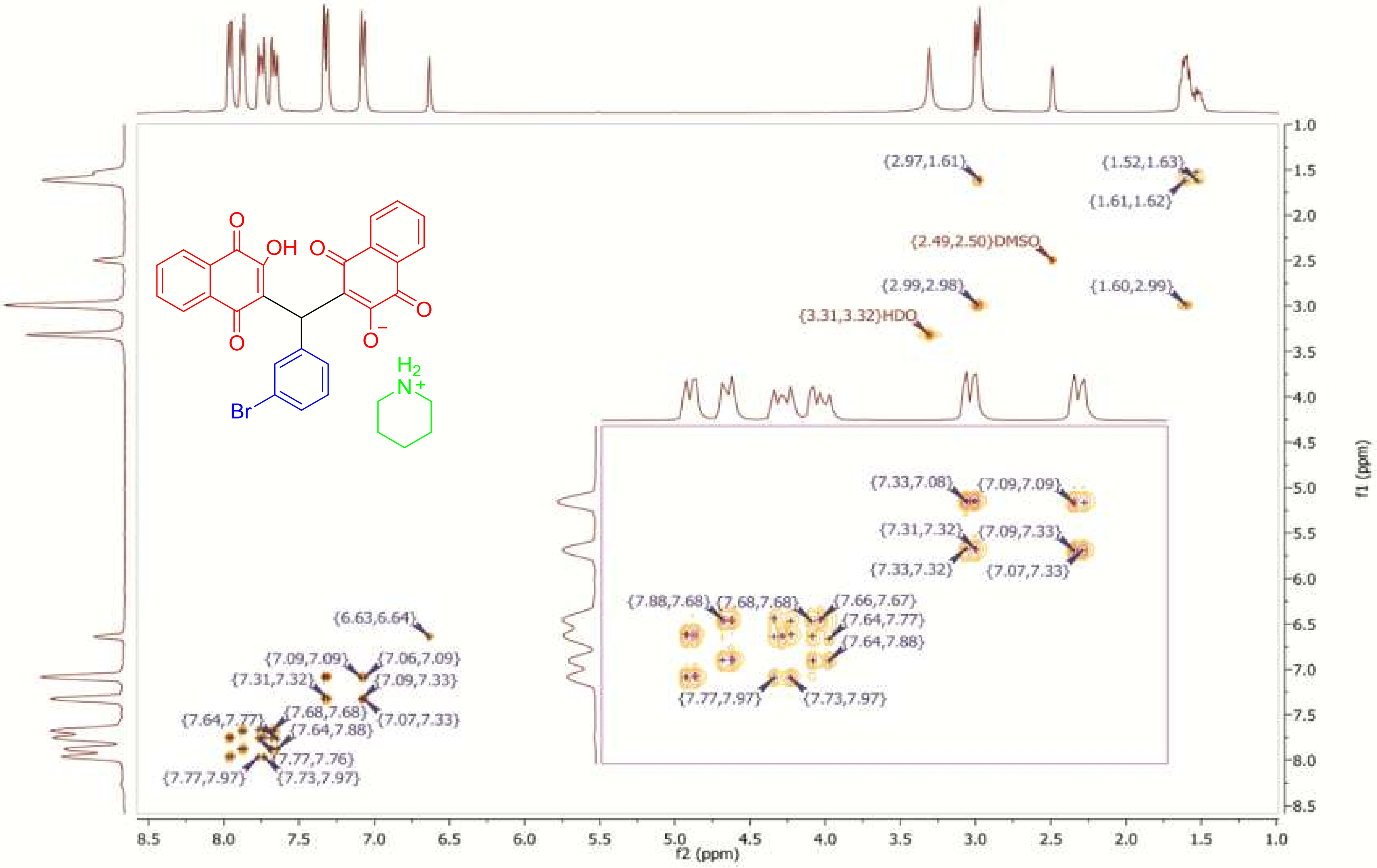

^{1}H^{1}H, COSY-NMR spectrum of piperidin-1-ium 3-((3-bromophenyl)(3-hydroxy-1,4-dioxo-1,4-dihydronaphthalen-2-yl)methyl)-1,4-dioxo-1,4-dihydronaphthalen-2-olate

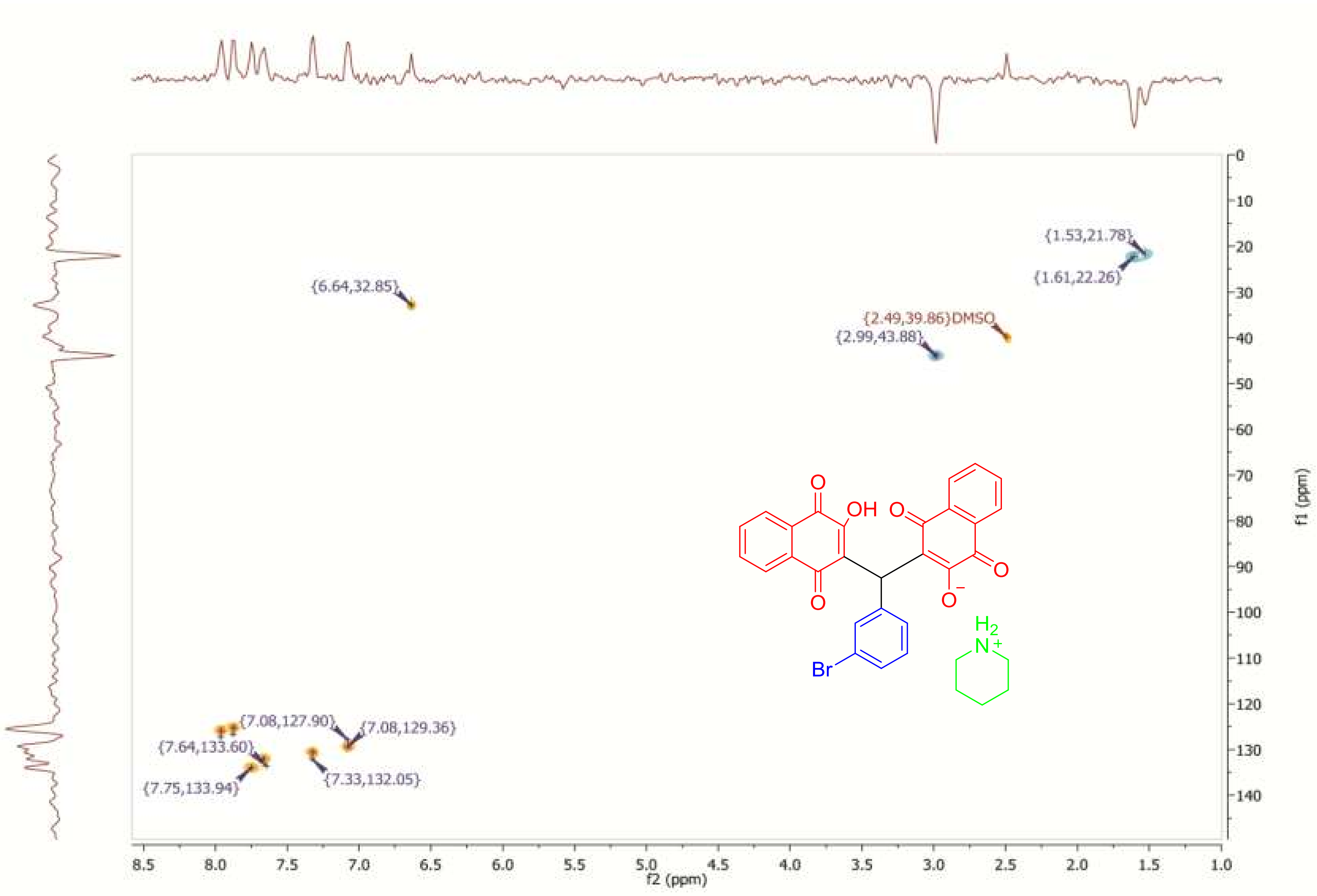

^{1}H^{13}C, HSQC-NMR spectrum piperidin-1-ium 3-((3-bromophenyl)(3-hydroxy-1,4-dioxo-1,4-dihydronaphthalen-2-yl)methyl)-1,4-dioxo-1,4-dihydronaphthalen-2-olate

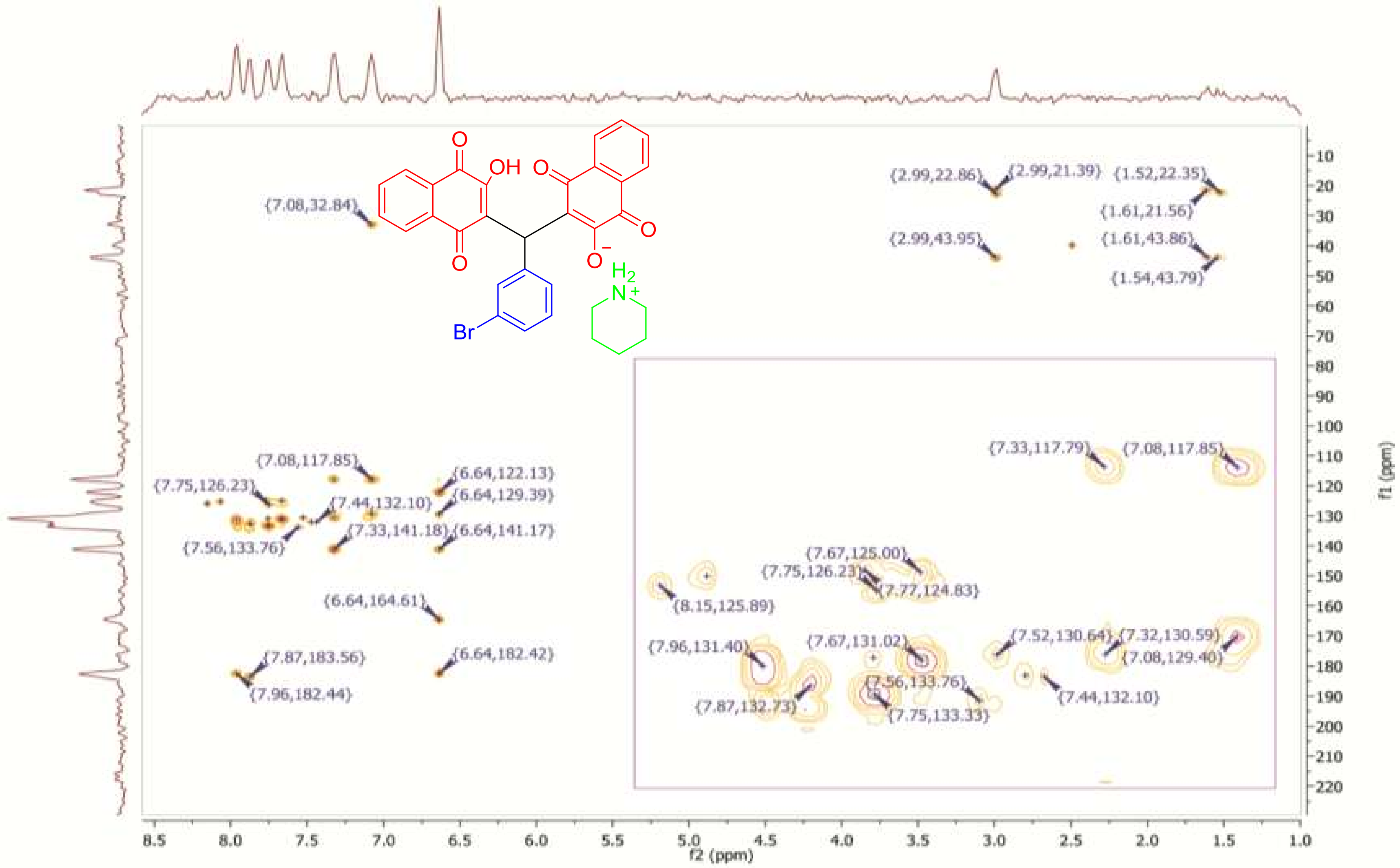

$^1H^{13}C$, HMBC-NMR spectrum of piperidin-1-ium 3-((3-bromophenyl)(3-hydroxy-1,4-dioxo-1,4-dihydronaphthalen-2-yl)methyl)-1,4-dioxo-1,4-dihydronaphthalen-2-olate

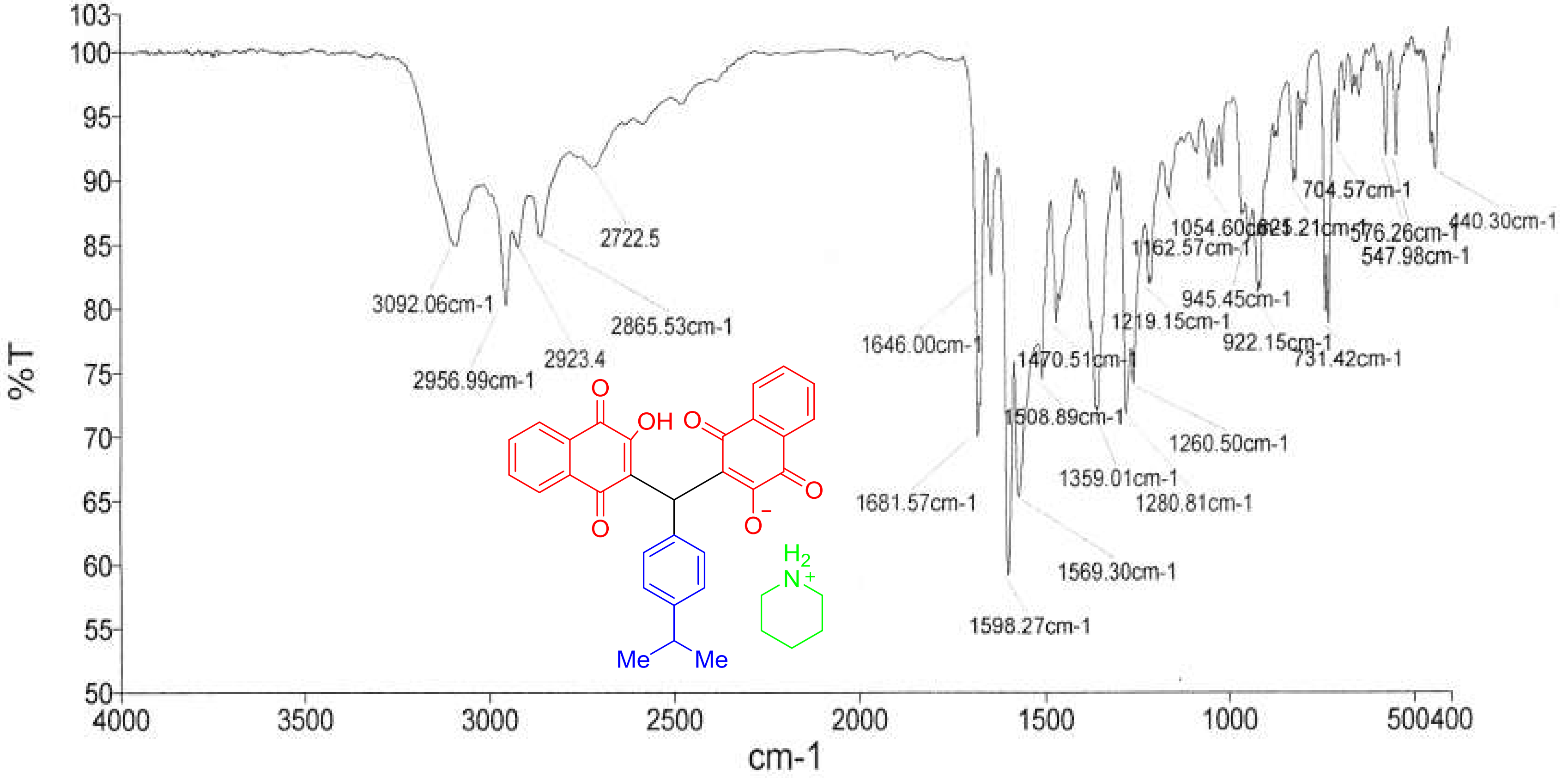

FT-IR spectrum of piperidin-1-ium 3-((3-hydroxy-1,4-dioxo-1,4-dihydronaphthalen-2-yl)(4-isopropylphenyl)methyl)-1,4-dioxo-1,4-dihydronaphthalen-2-olate

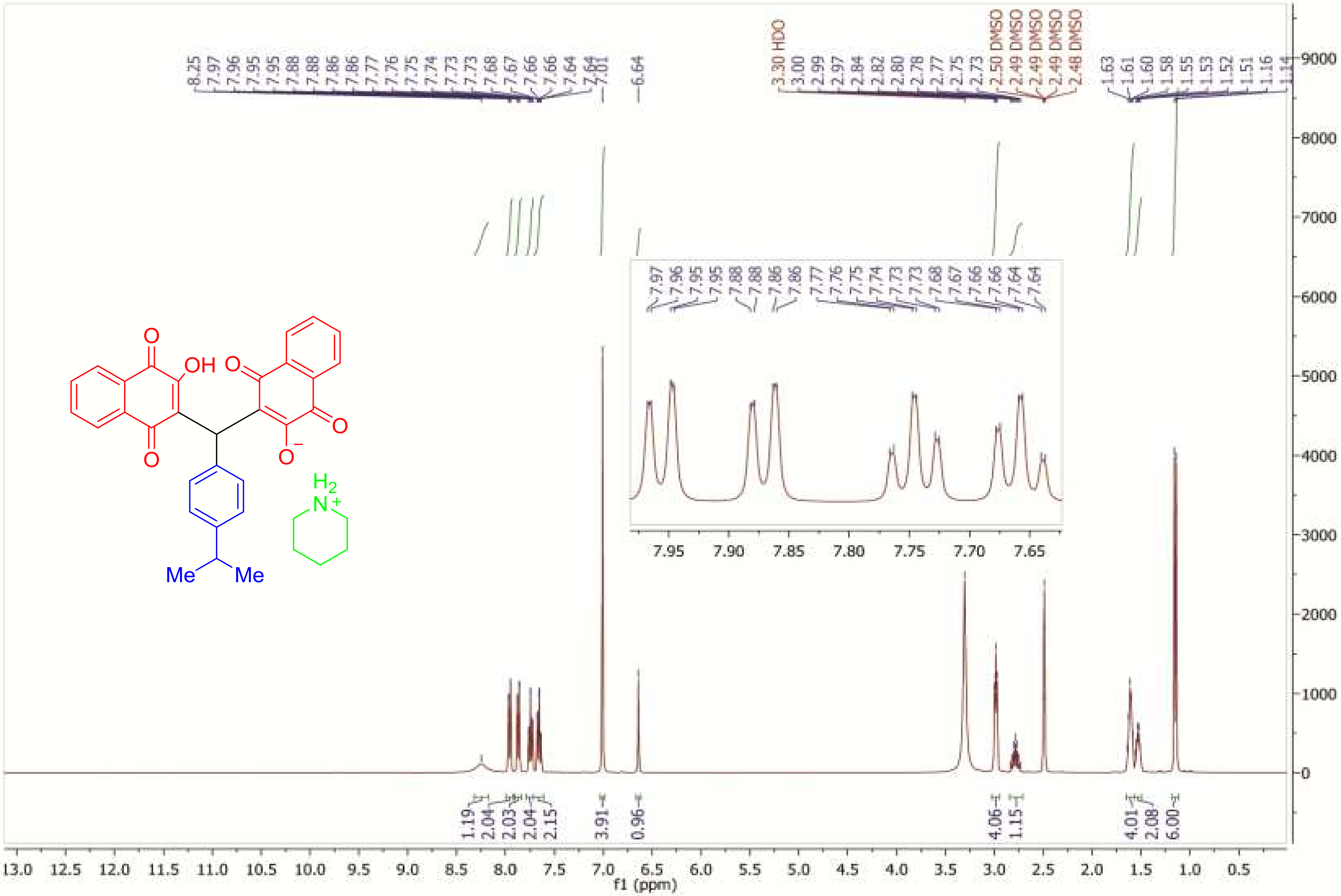

^{1}H NMR spectrum of piperidin-1-ium 3-((3-hydroxy-1,4-dioxo-1,4-dihydronaphthalen-2-yl)(4-isopropylphenyl)methyl)-1,4-dioxo-1,4-dihydronaphthalen-2-olate

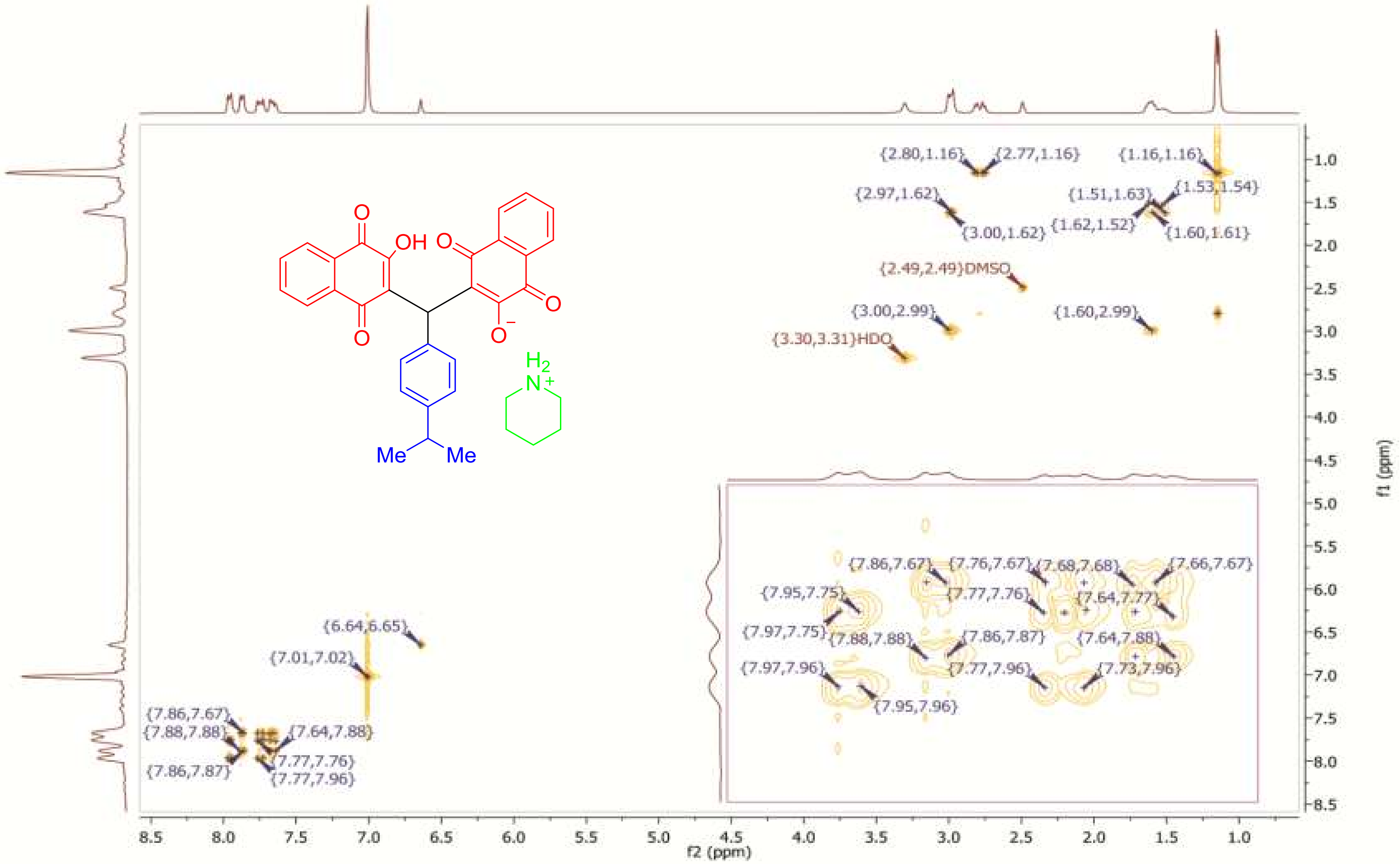

^{1}H^{1}H, COSY-NMR spectrum of piperidin-1-ium 3-((3-hydroxy-1,4-dioxo-1,4-dihydronaphthalen-2-yl)(4-isopropylphenyl)methyl)-1,4-dioxo-1,4-dihydronaphthalen-2-olate

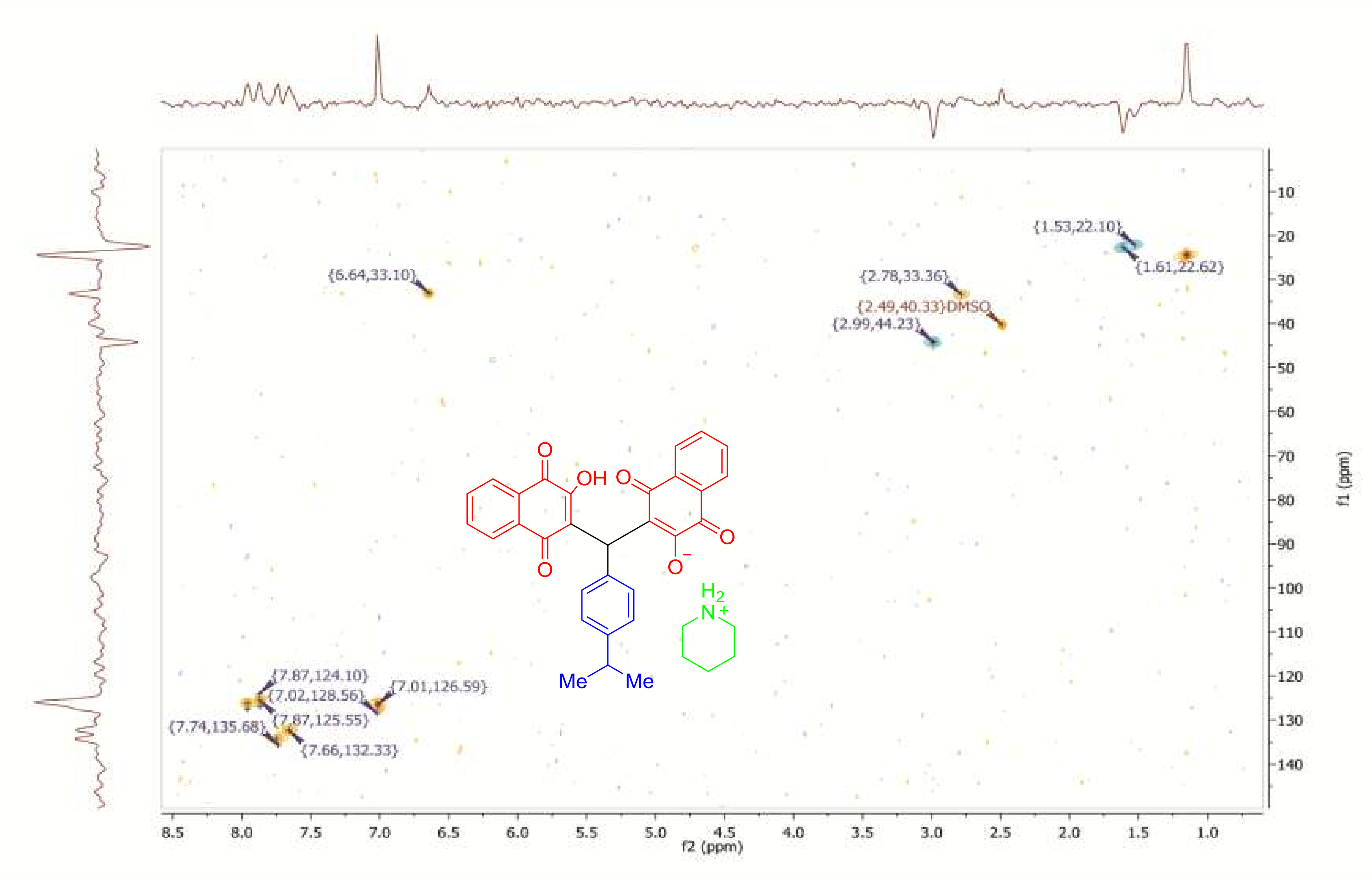

^{1}H^{13}C, HSQC-NMR spectrum piperidin-1-ium 3-((3-hydroxy-1,4-dioxo-1,4-dihydronaphthalen-2-yl)(4-isopropylphenyl)methyl)-1,4-dioxo-1,4-dihydronaphthalen-2-olate

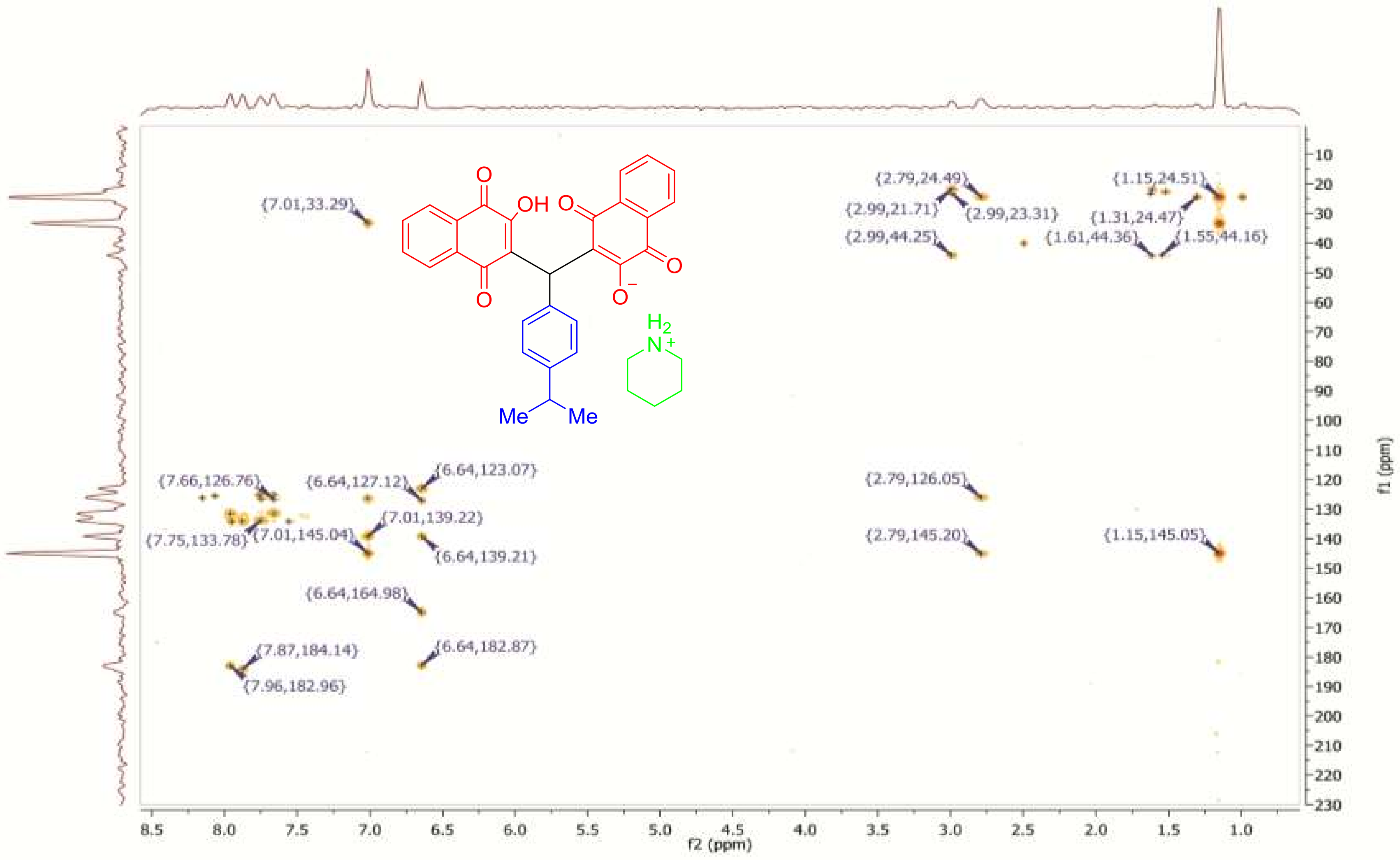

^{1}H^{13}C, HMBC-NMR spectrum of piperidin-1-ium 3-((3-hydroxy-1,4-dioxo-1,4-dihydronaphthalen-2-yl)(4-isopropylphenyl)methyl)-1,4-dioxo-1,4-dihydronaphthalen-2-olate

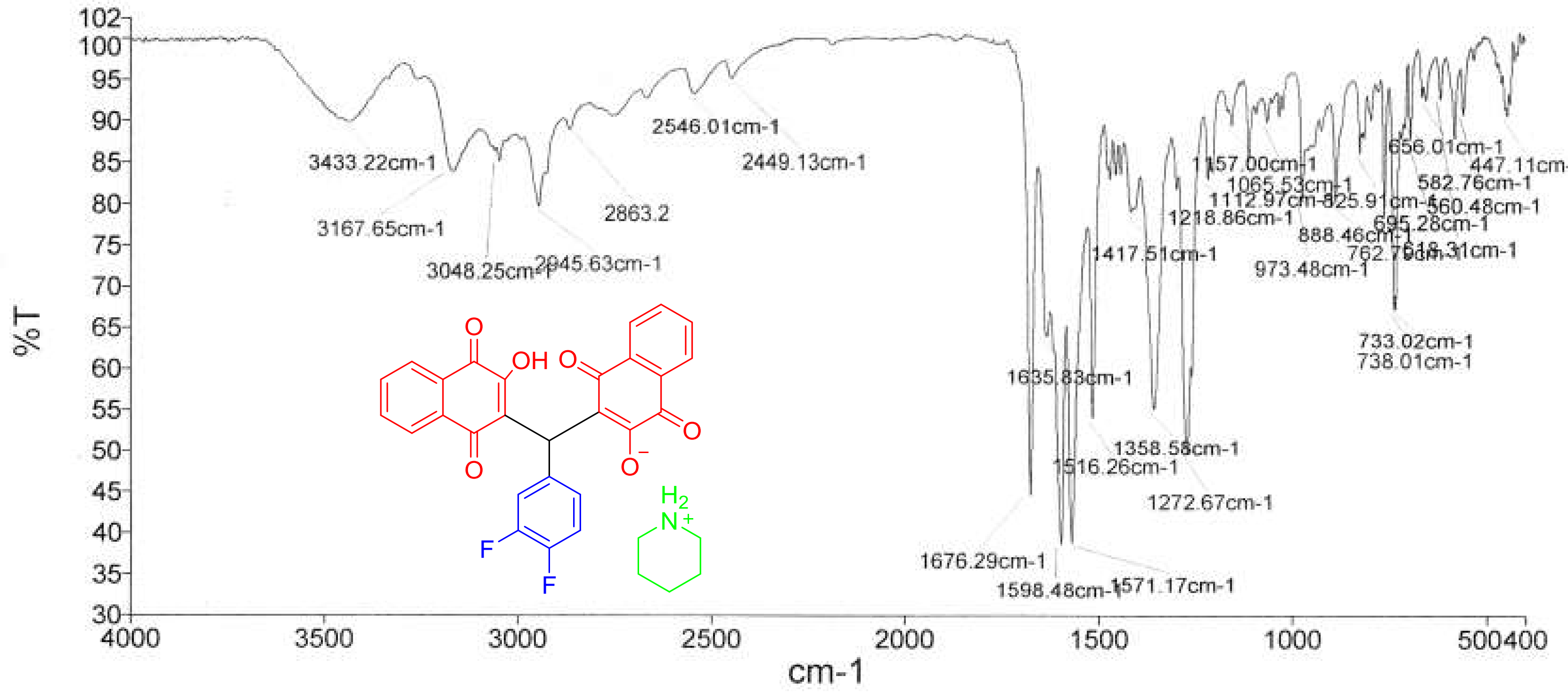

FT-IR spectrum of piperidin-1-ium 3-((3,4-difluorophenyl)(3-hydroxy-1,4-dioxo-1,4-dihydronaphthalen-2-yl)methyl)-1,4-dioxo-1,4-dihydronaphthalen-2-olate

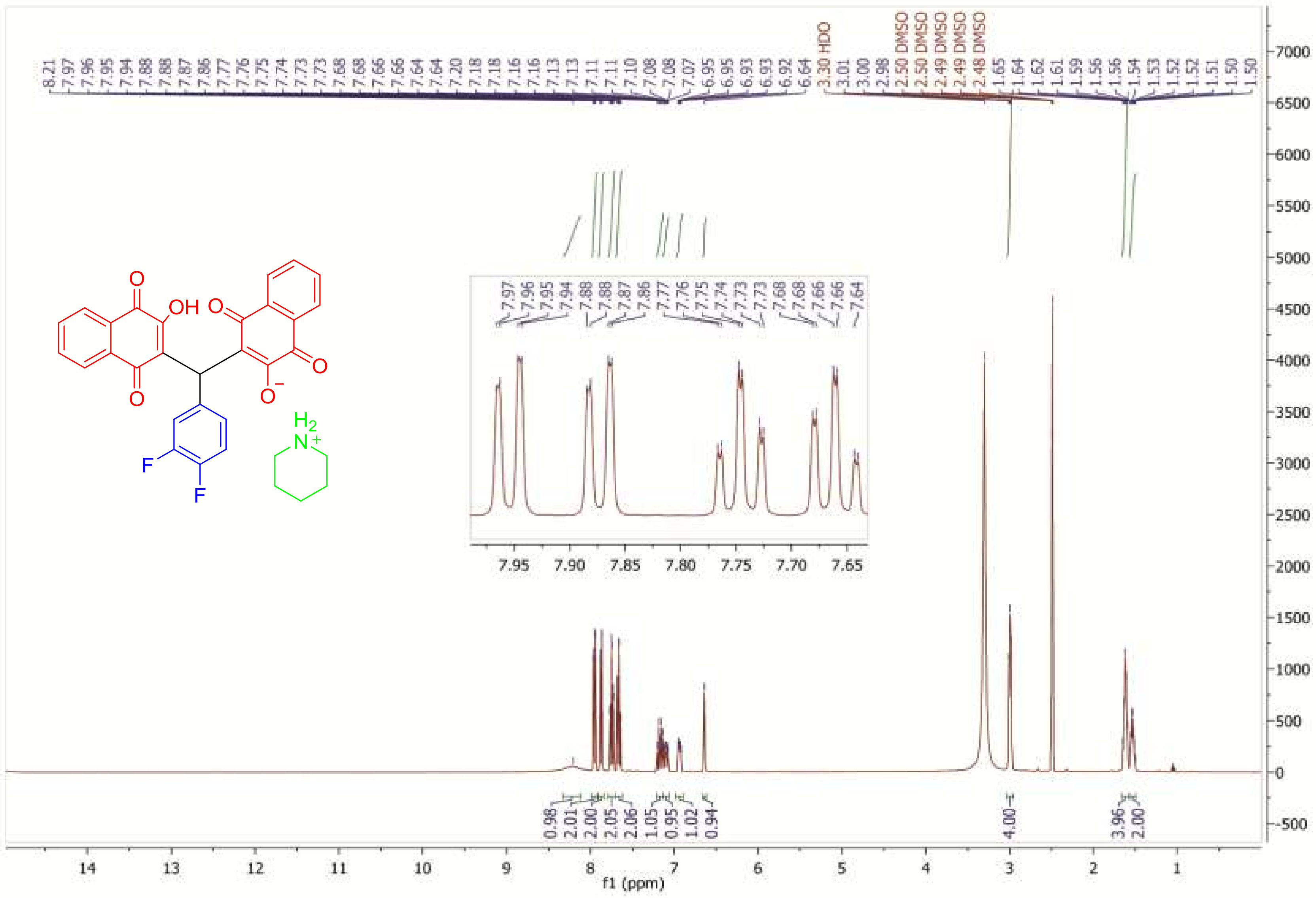

^{1}H NMR spectrum of piperidin-1-ium 3-((3,4-difluorophenyl)(3-hydroxy-1,4-dioxo-1,4-dihydronaphthalen-2-yl)methyl)-1,4-dioxo-1,4-dihydronaphthalen-2-olate

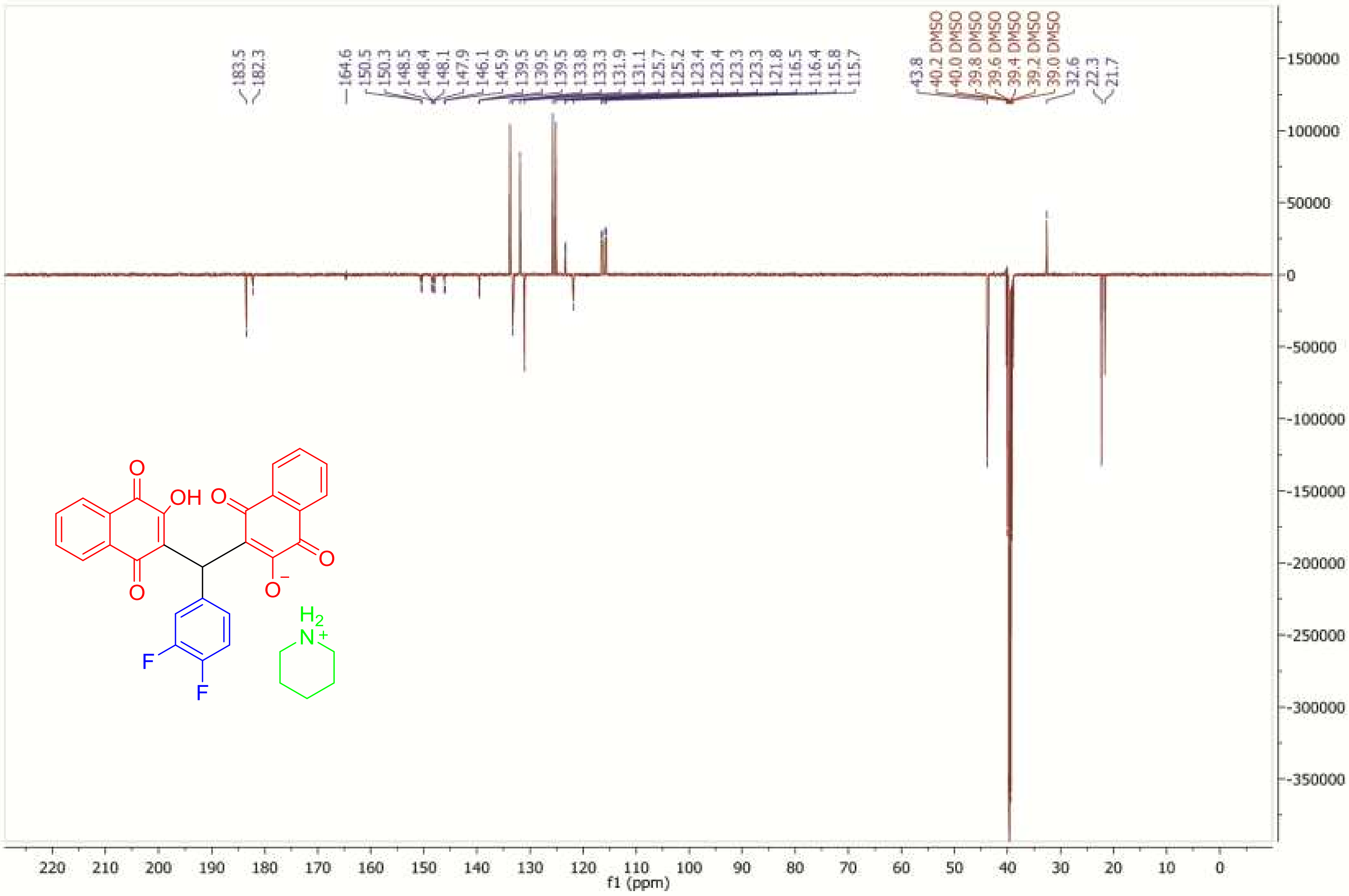

^{13}C NMR (DEPT-135) spectrum of piperidin-1-ium 3-((3,4-difluorophenyl)(3-hydroxy-1,4-dioxo-1,4-dihydronaphthalen-2-yl)methyl)-1,4-dioxo-1,4-dihydronaphthalen-2-olate

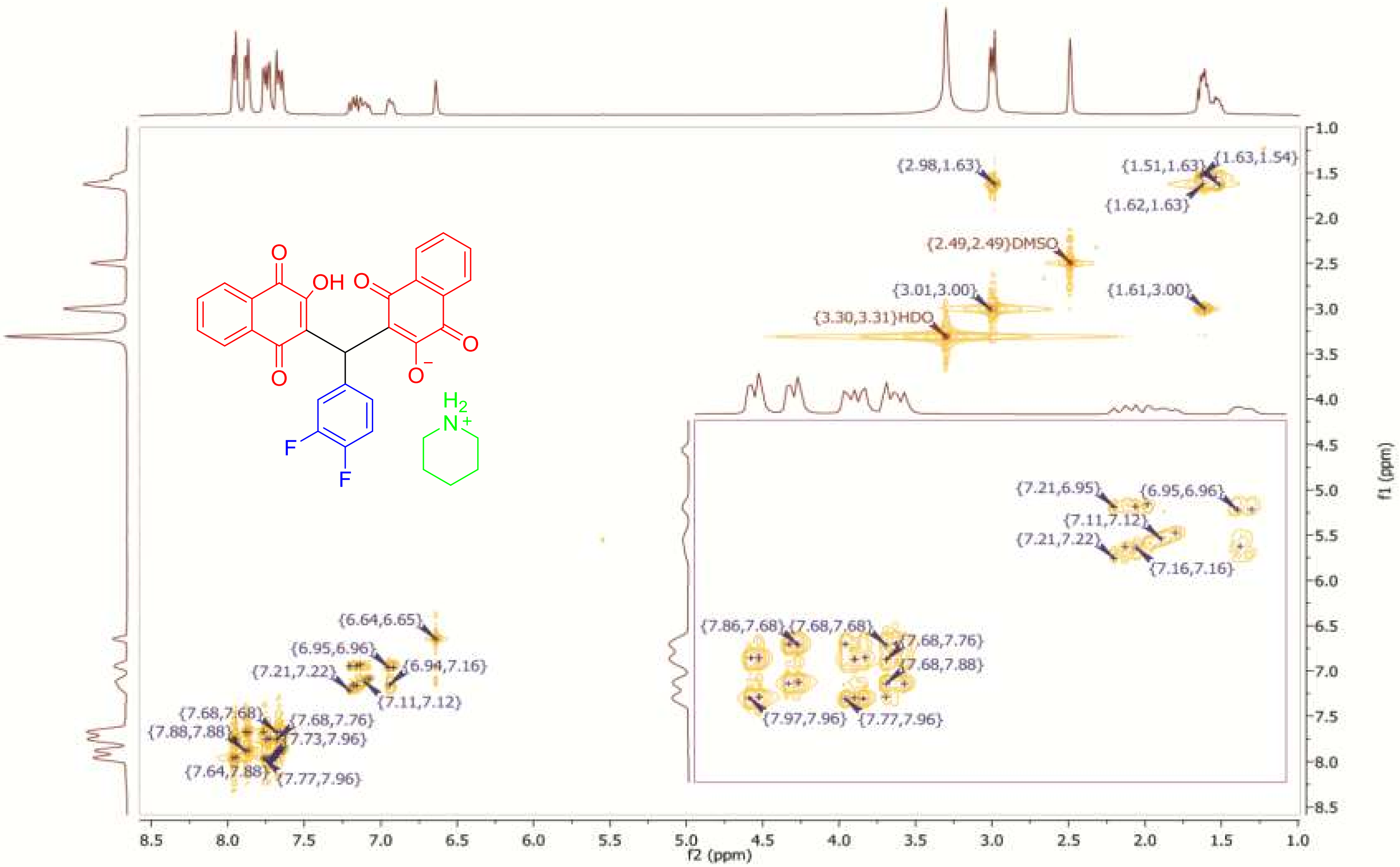

^{1}H^{1}H, COSY-NMR spectrum of piperidin-1-ium 3-((3,4-difluorophenyl)(3-hydroxy-1,4-dioxo-1,4-dihydronaphthalen-2-yl)methyl)-1,4-dioxo-1,4-dihydronaphthalen-2-olate

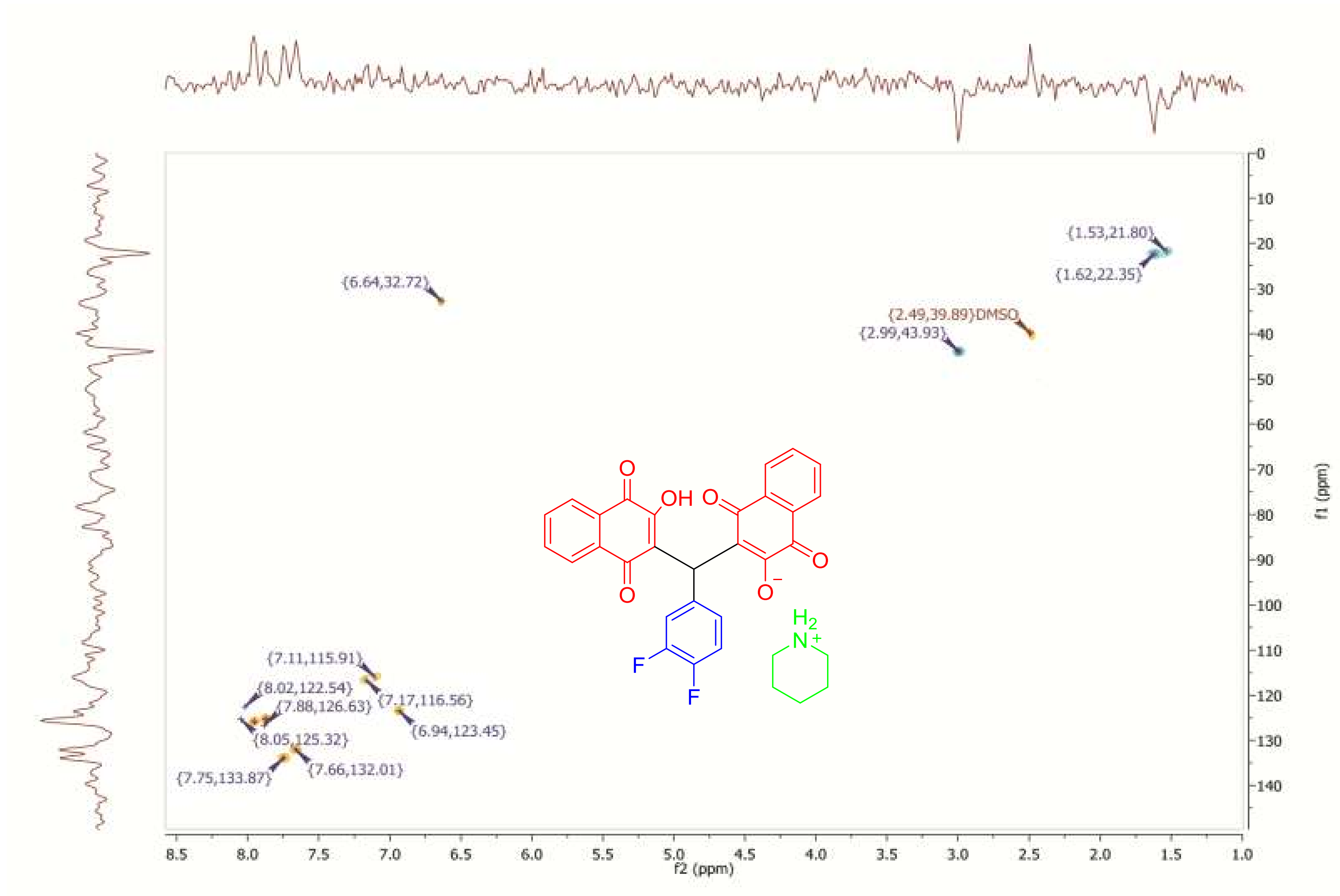

$^{1}H^{13}C$, HSQC-NMR spectrum piperidin-1-ium 3-((3,4-difluorophenyl)(3-hydroxy-1,4-dioxo-1,4-dihydronaphthalen-2-yl)methyl)-1,4-dioxo-1,4-dihydronaphthalen-2-olate

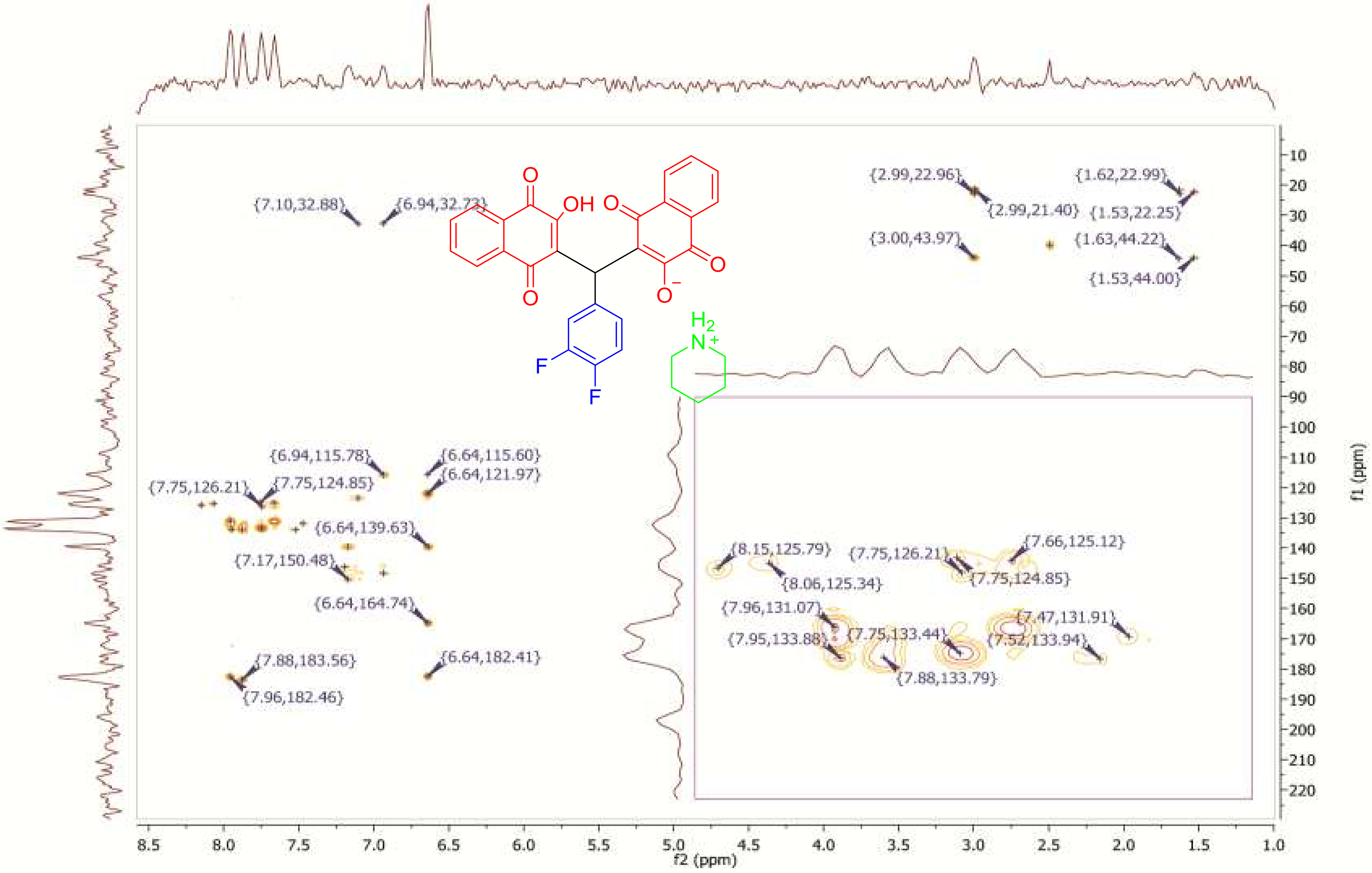

$^{1}H^{13}C$, HMBC-NMR spectrum of piperidin-1-ium 3-((3,4-difluorophenyl)(3-hydroxy-1,4-dioxo-1,4-dihydronaphthalen-2-yl)methyl)-1,4-dioxo-1,4-dihydronaphthalen-2-olate

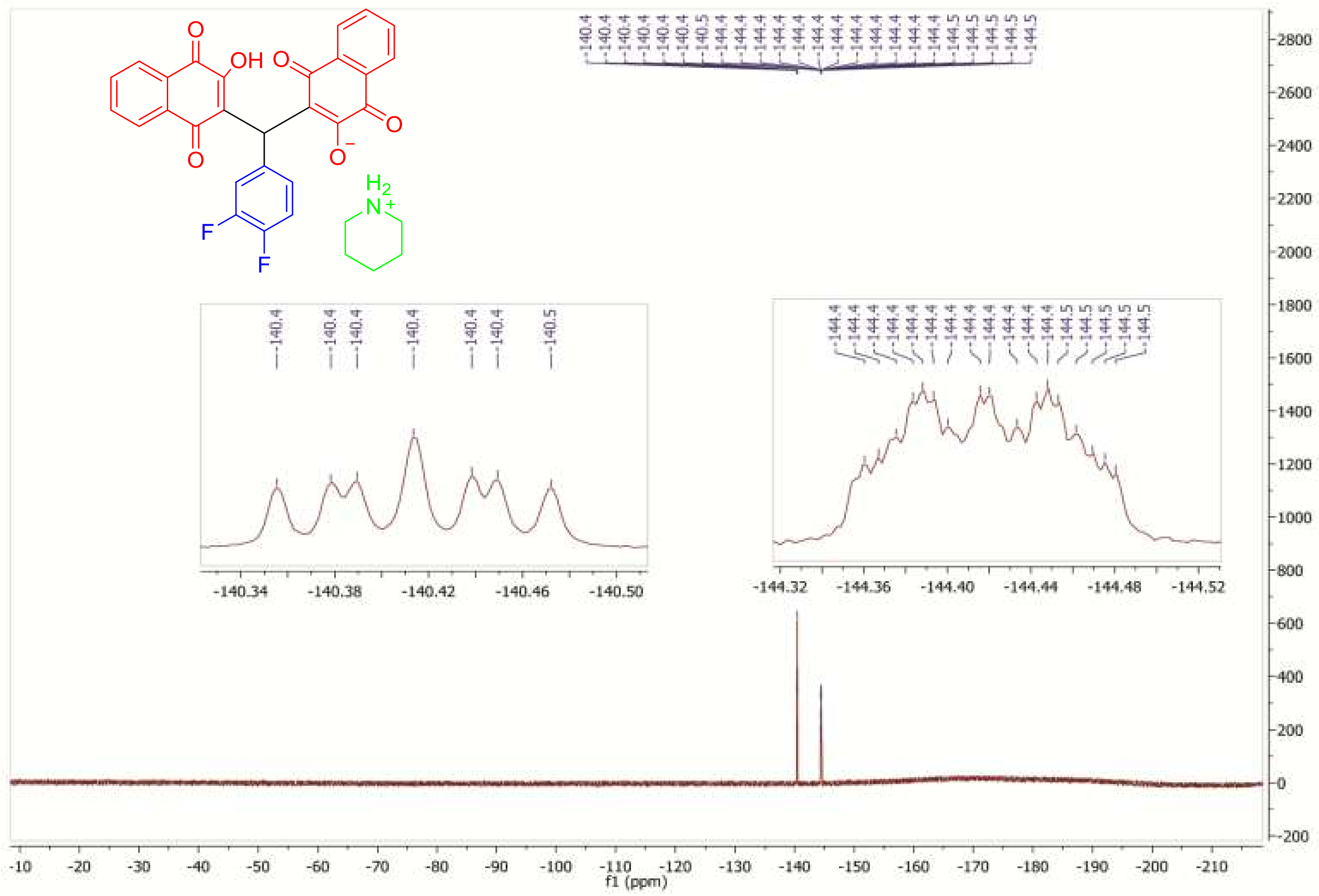

^{19}F NMR (spin-spin coupling) piperidin-1-ium 3-((3,4-difluorophenyl)(3-hydroxy-1,4-dioxo-1,4-dihydronaphthalen-2-yl)methyl)-1,4-dioxo-1,4-dihydronaphthalen-2-olate

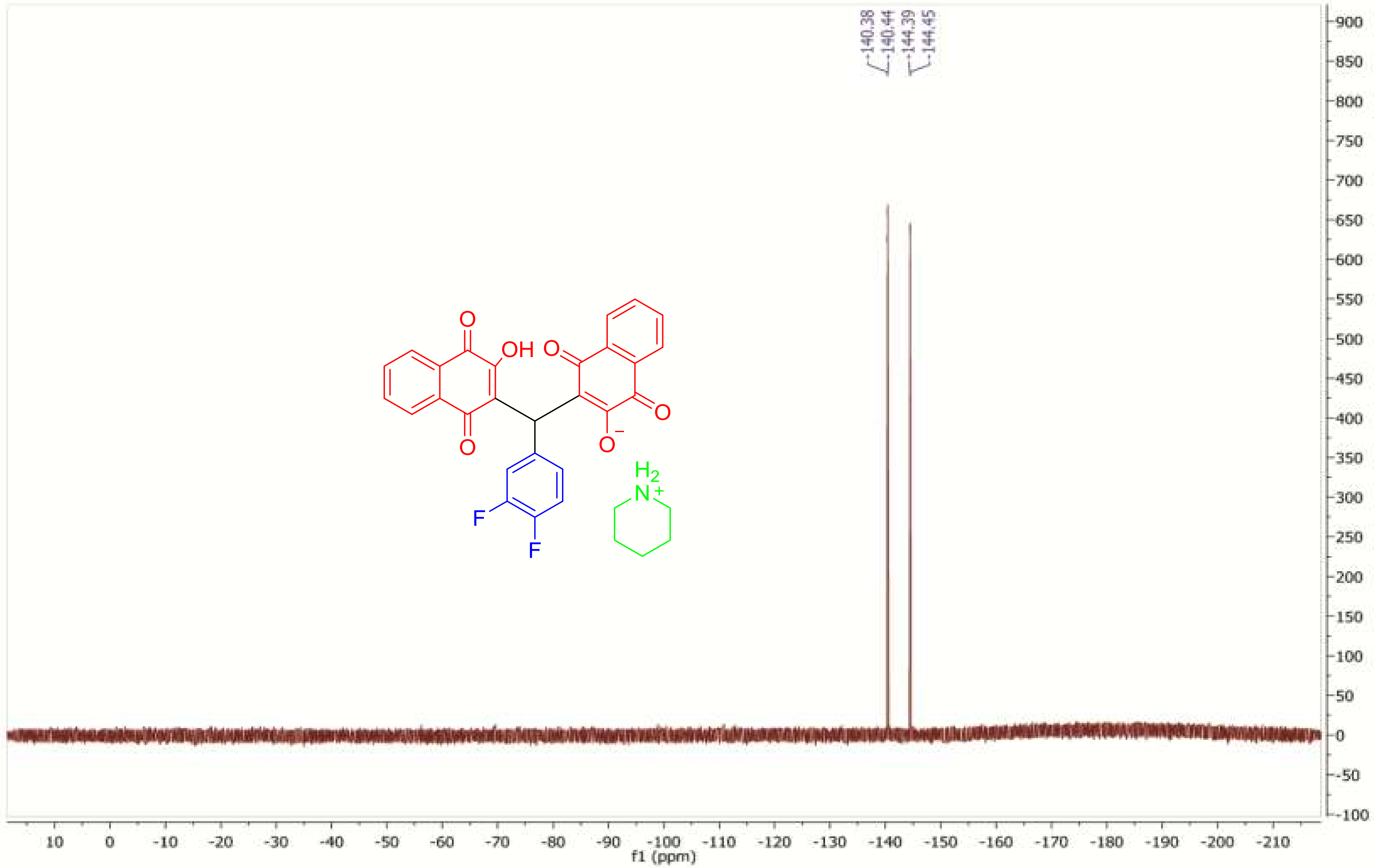

^{19}F NMR piperidin-1-ium 3-((3,4-difluorophenyl)(3-hydroxy-1,4-dioxo-1,4-dihydronaphthalen-2-yl)methyl)-1,4-dioxo-1,4-dihydronaphthalen-2-olate

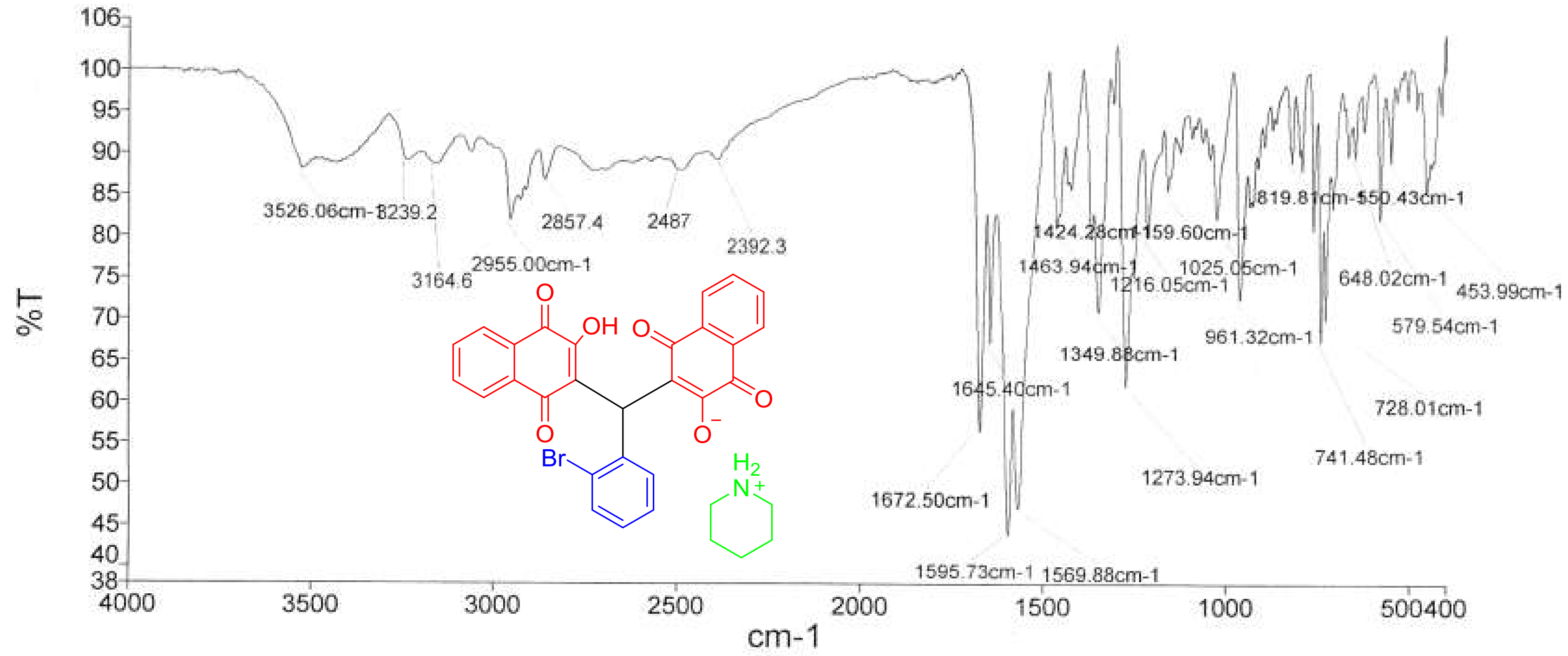

FT-IR spectrum of piperidin-1-ium 3-((2-bromophenyl)(3-hydroxy-1,4-dioxo-1,4-dihydronaphthalen-2-yl)methyl)-1,4-dioxo-1,4-dihydronaphthalen-2-olate

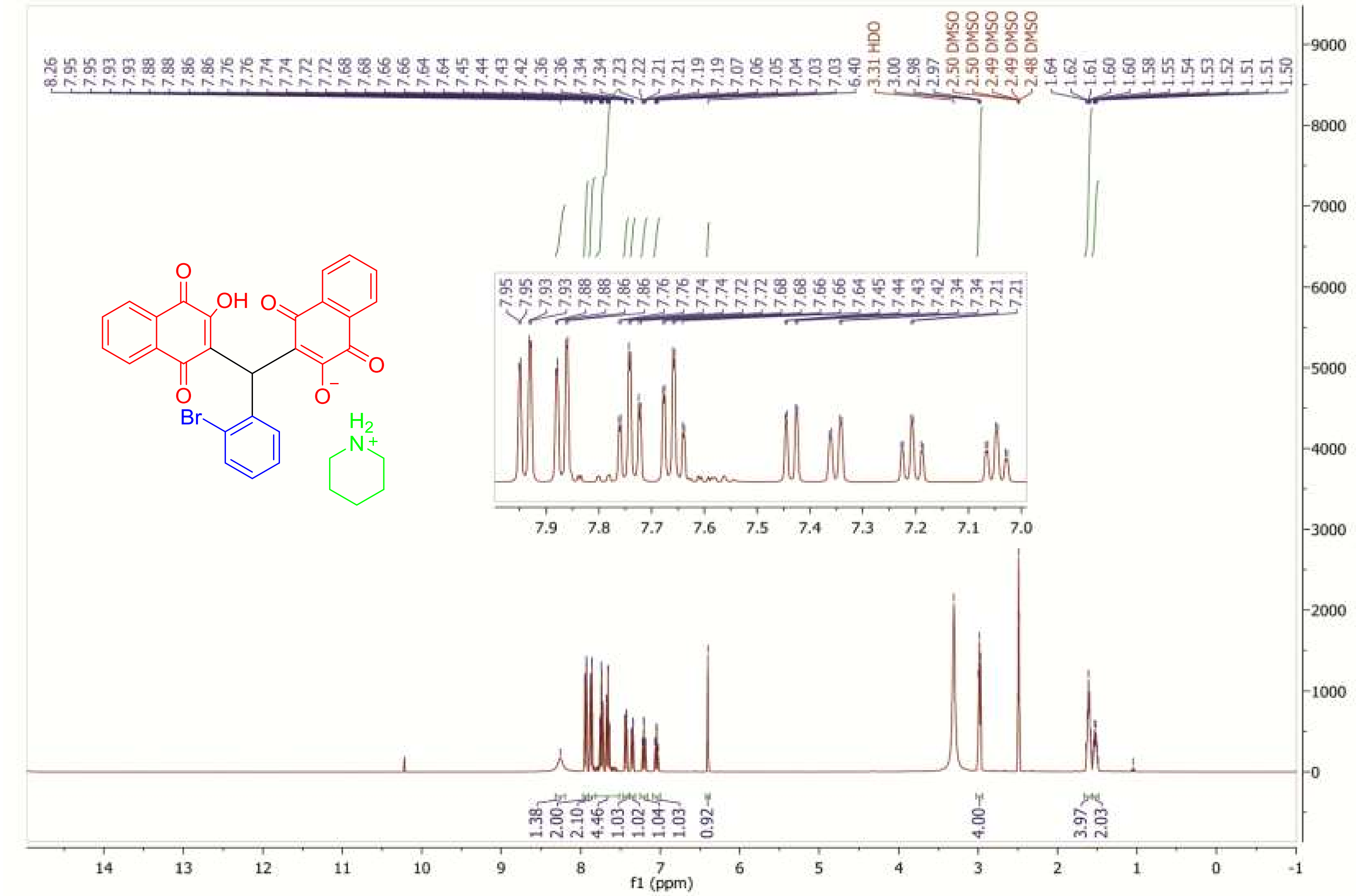

^{1}H NMR spectrum of piperidin-1-ium 3-((2-bromophenyl)(3-hydroxy-1,4-dioxo-1,4-dihydronaphthalen-2-yl)methyl)-1,4-dioxo-1,4-dihydronaphthalen-2-olate

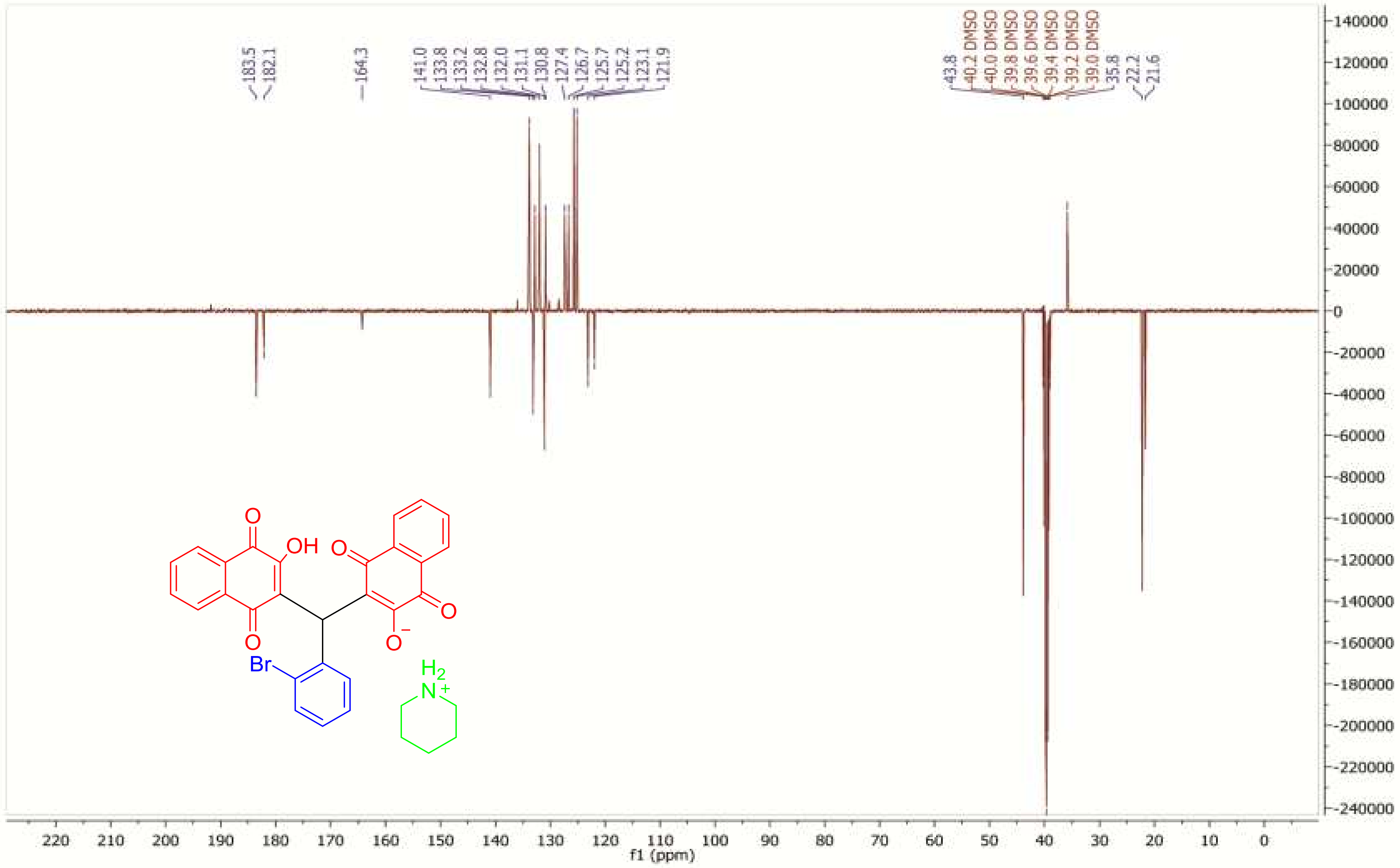

^{13}C NMR (DEPT-135) spectrum of piperidin-1-ium 3-((2-bromophenyl)(3-hydroxy-1,4-dioxo-1,4-dihydronaphthalen-2-yl)methyl)-1,4-dioxo-1,4-dihydronaphthalen-2-olate

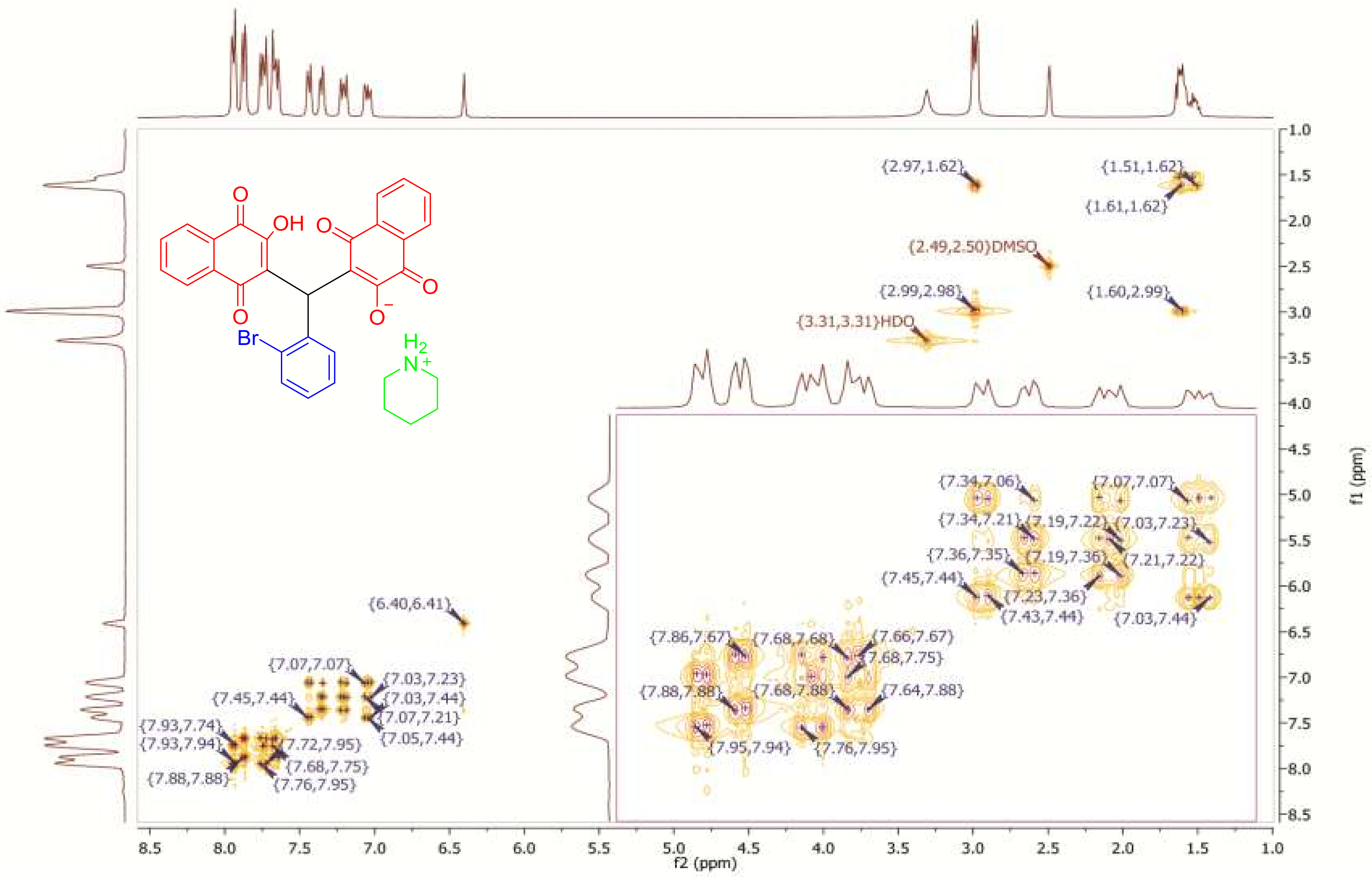

^{1}H^{1}H, COSY-NMR spectrum of piperidin-1-ium 3-((2-bromophenyl)(3-hydroxy-1,4-dioxo-1,4-dihydronaphthalen-2-yl)methyl)-1,4-dioxo-1,4-dihydronaphthalen-2-olate

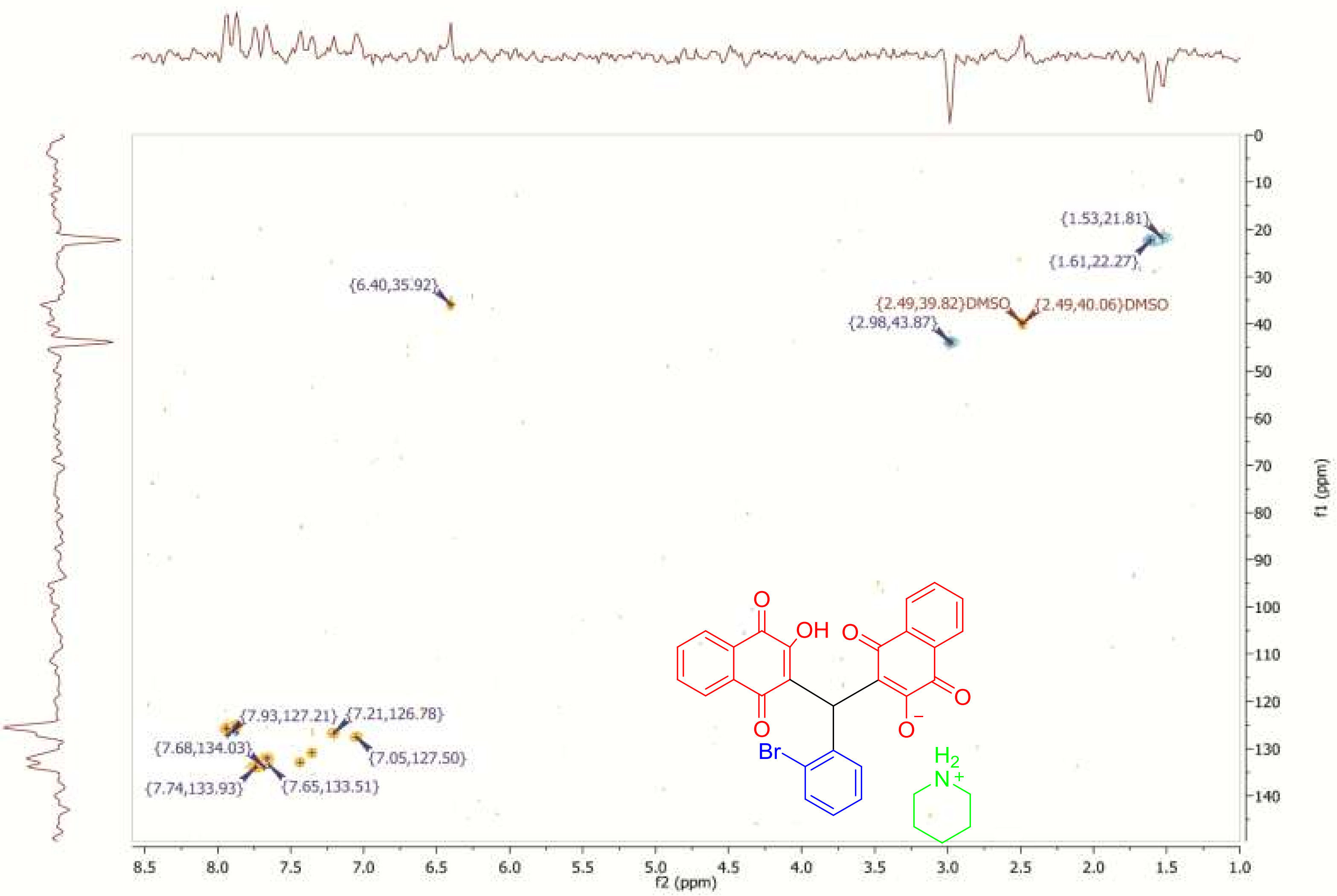

^{1}H^{13}C, HSQC-NMR spectrum of -1-ium 3-((2-bromophenyl)(3-hydroxy-1,4-dioxo-1,4-dihydronaphthalen-2-yl)methyl)-1,4-dioxo-1,4-dihydronaphthalen-2-olate

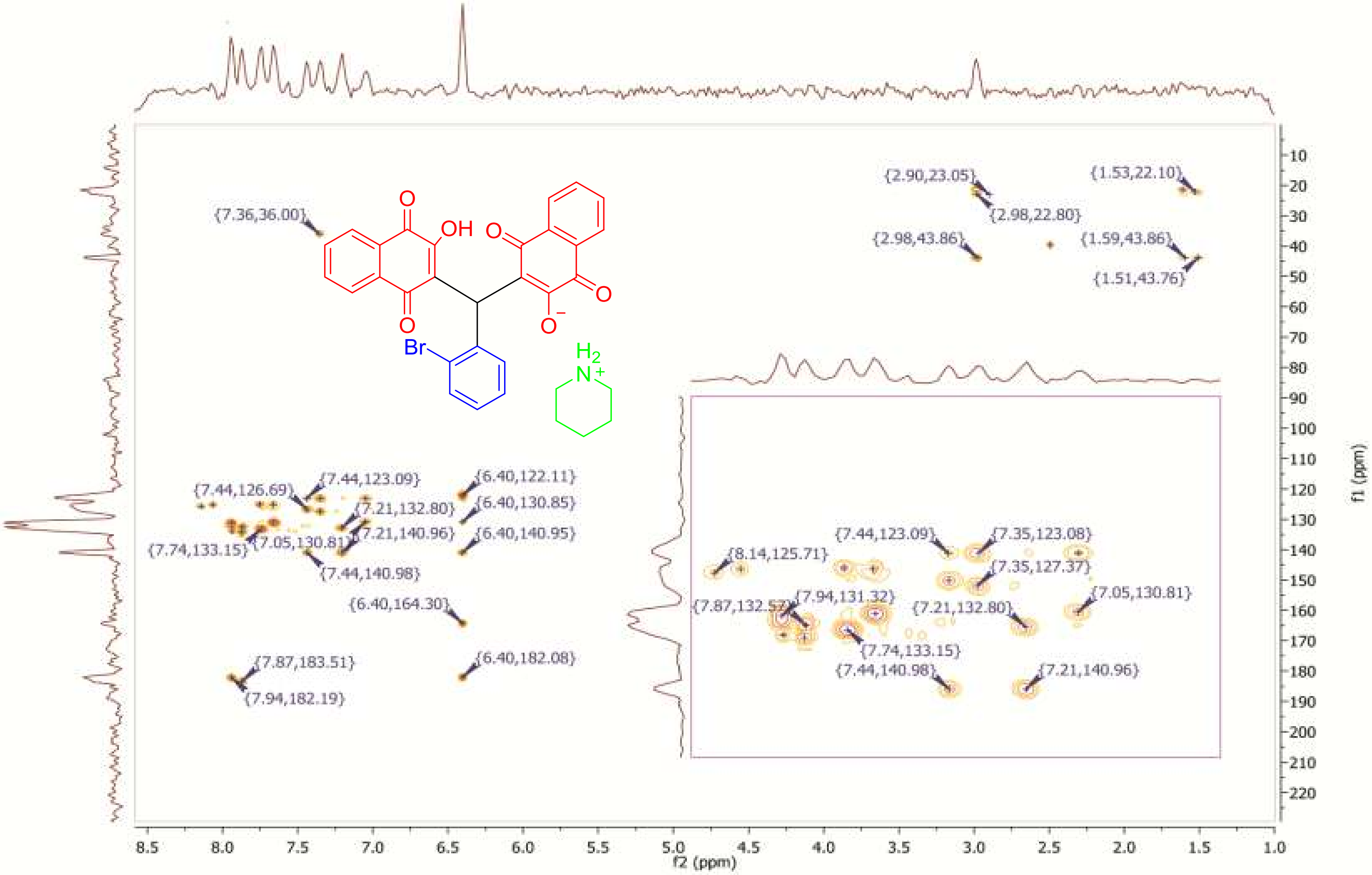

^{1}H^{13}C, HMBC-NMR spectrum of piperidin-1-ium 3-((2-bromophenyl)(3-hydroxy-1,4-dioxo-1,4-dihydronaphthalen-2-yl)methyl)-1,4-dioxo-1,4-dihydronaphthalen-2-olate

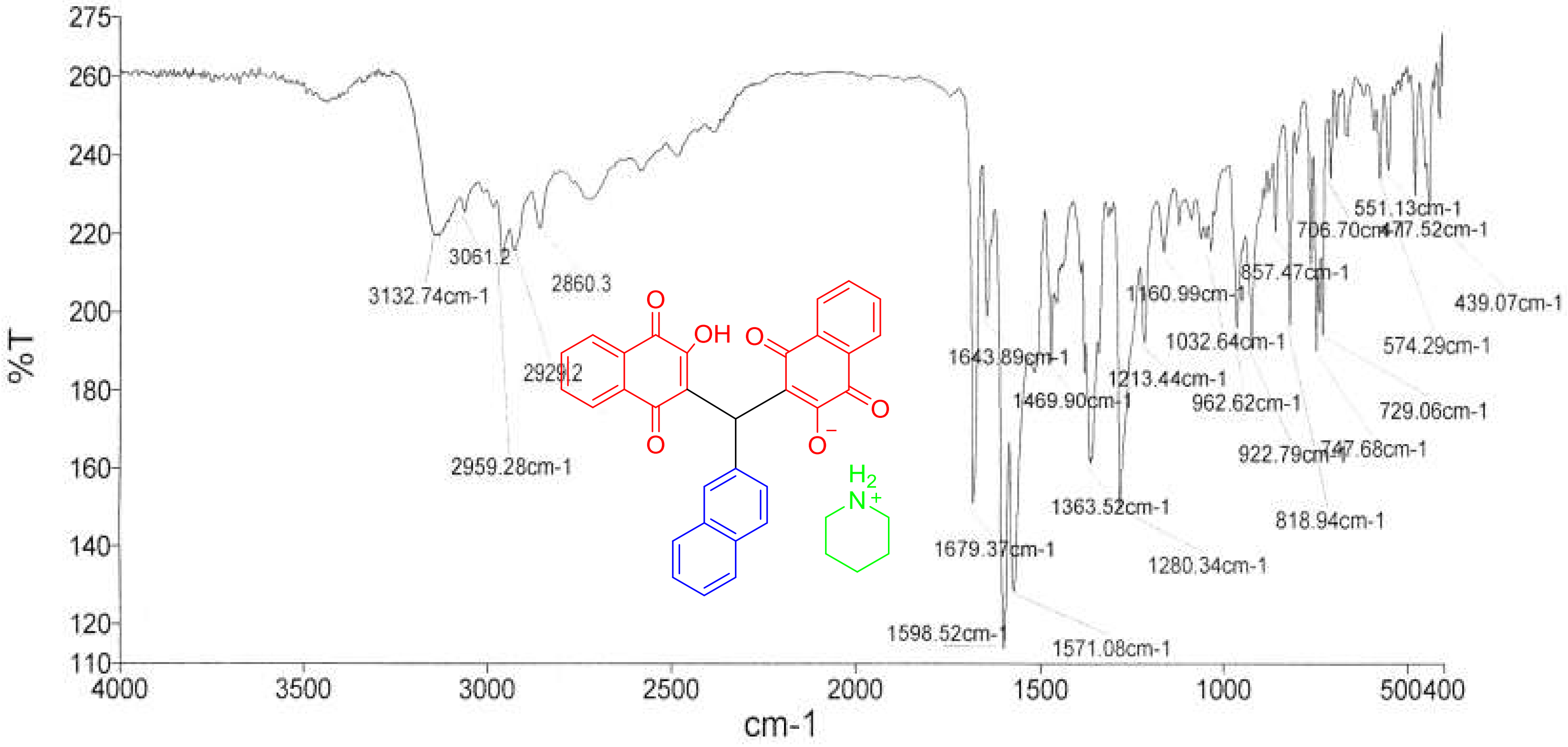

FT-IR spectrum of piperidin-1-ium 3-((3-hydroxy-1,4-dioxo-1,4-dihydronaphthalen-2-yl)(naphthalen-2-yl)methyl)-1,4-dioxo-1,4-dihydronaphthalen-2-olate

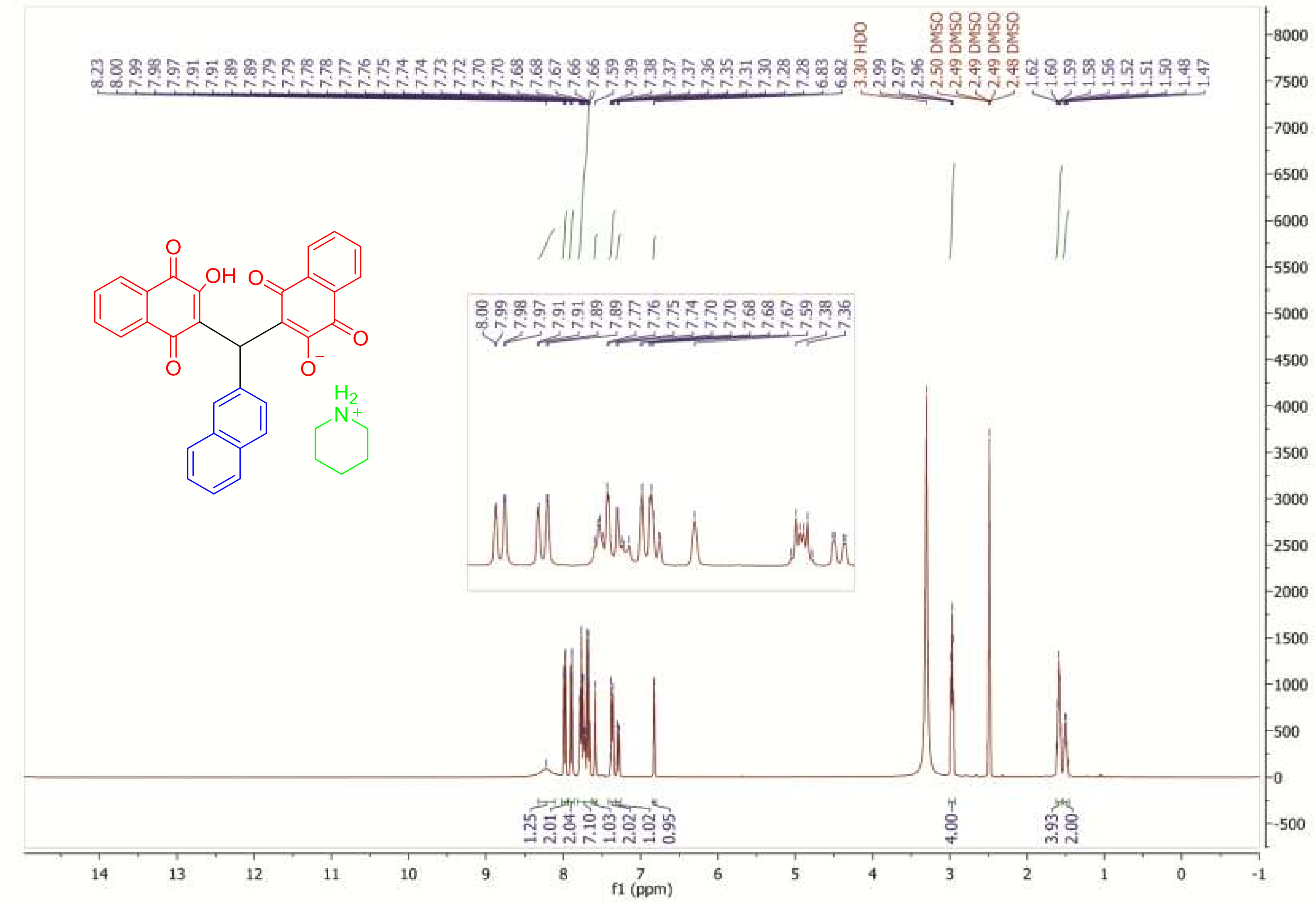

^{1}H NMR spectrum of piperidin-1-ium 3-((3-hydroxy-1,4-dioxo-1,4-dihydronaphthalen-2-yl)(naphthalen-2-yl)methyl)-1,4-dioxo-1,4-dihydronaphthalen-2-olate

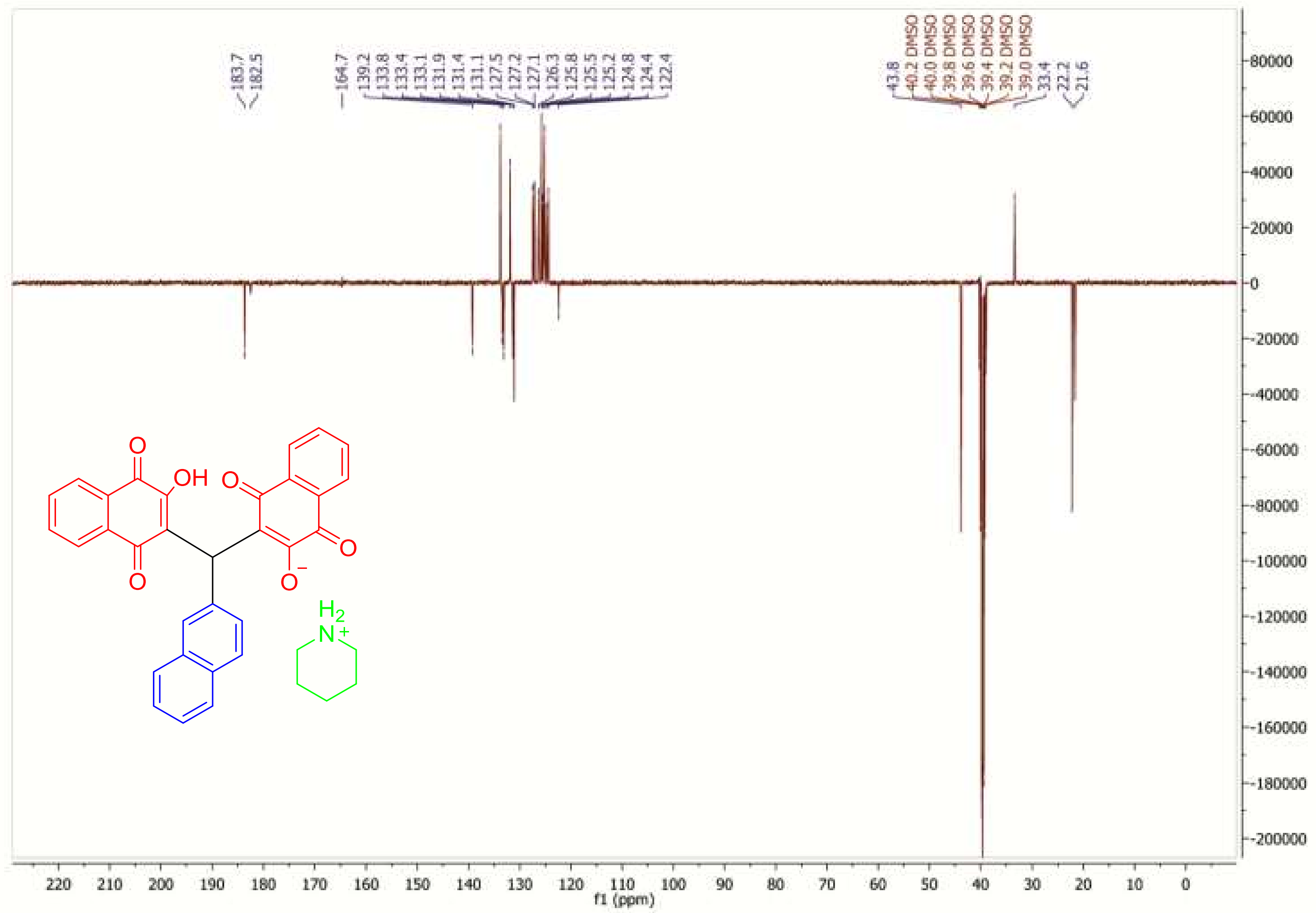

^{13}C NMR (DEPT-135) spectrum of piperidin-1-ium 3-((3-hydroxy-1,4-dioxo-1,4-dihydronaphthalen-2-yl)(naphthalen-2-yl)methyl)-1,4-dioxo-1,4-dihydronaphthalen-2-olate

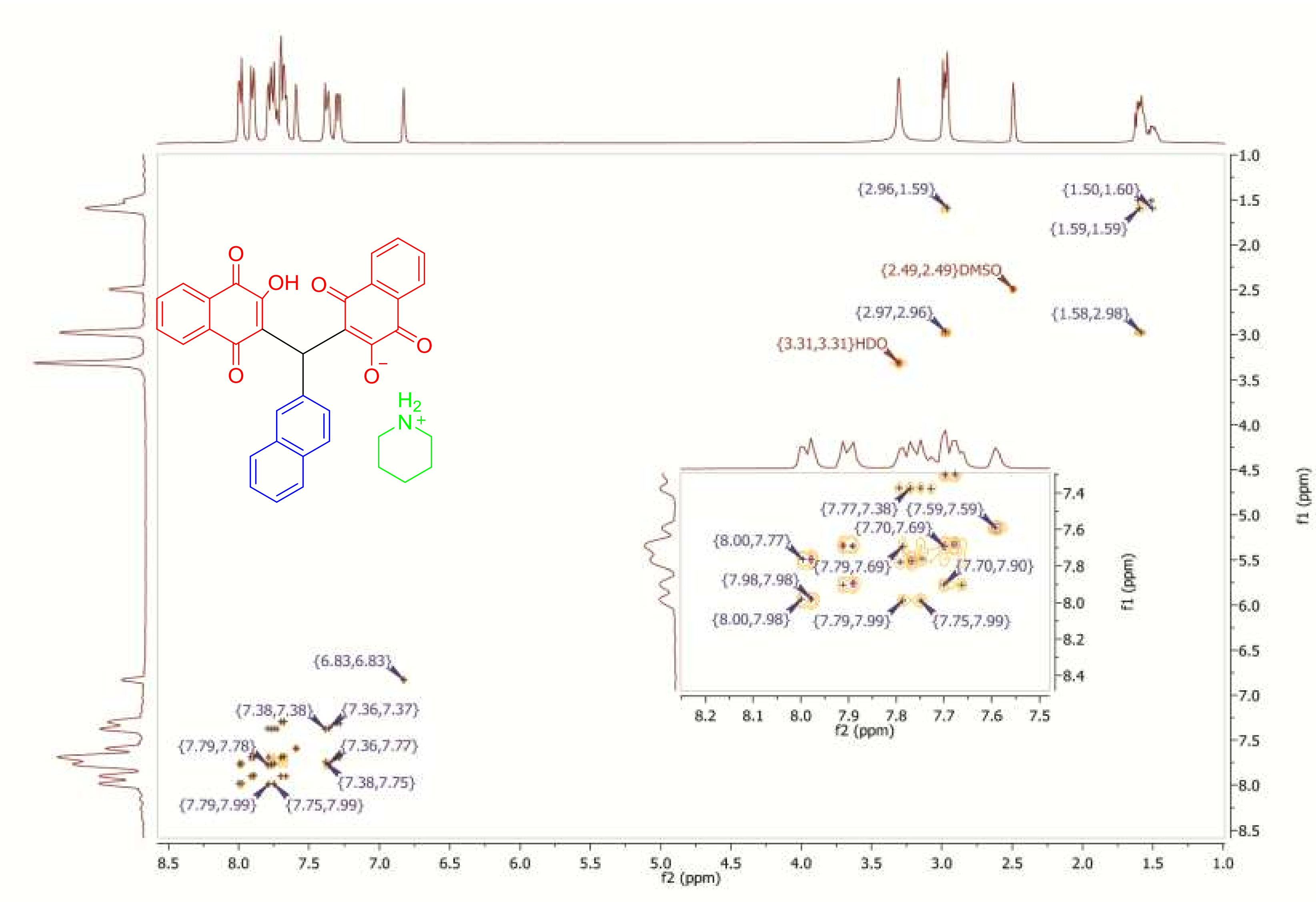

^{1}H^{1}H, COSY-NMR spectrum of piperidin-1-ium 3-((3-hydroxy-1,4-dioxo-1,4-dihydronaphthalen-2-yl)(naphthalen-2-yl)methyl)-1,4-dioxo-1,4-dihydronaphthalen-2-olate

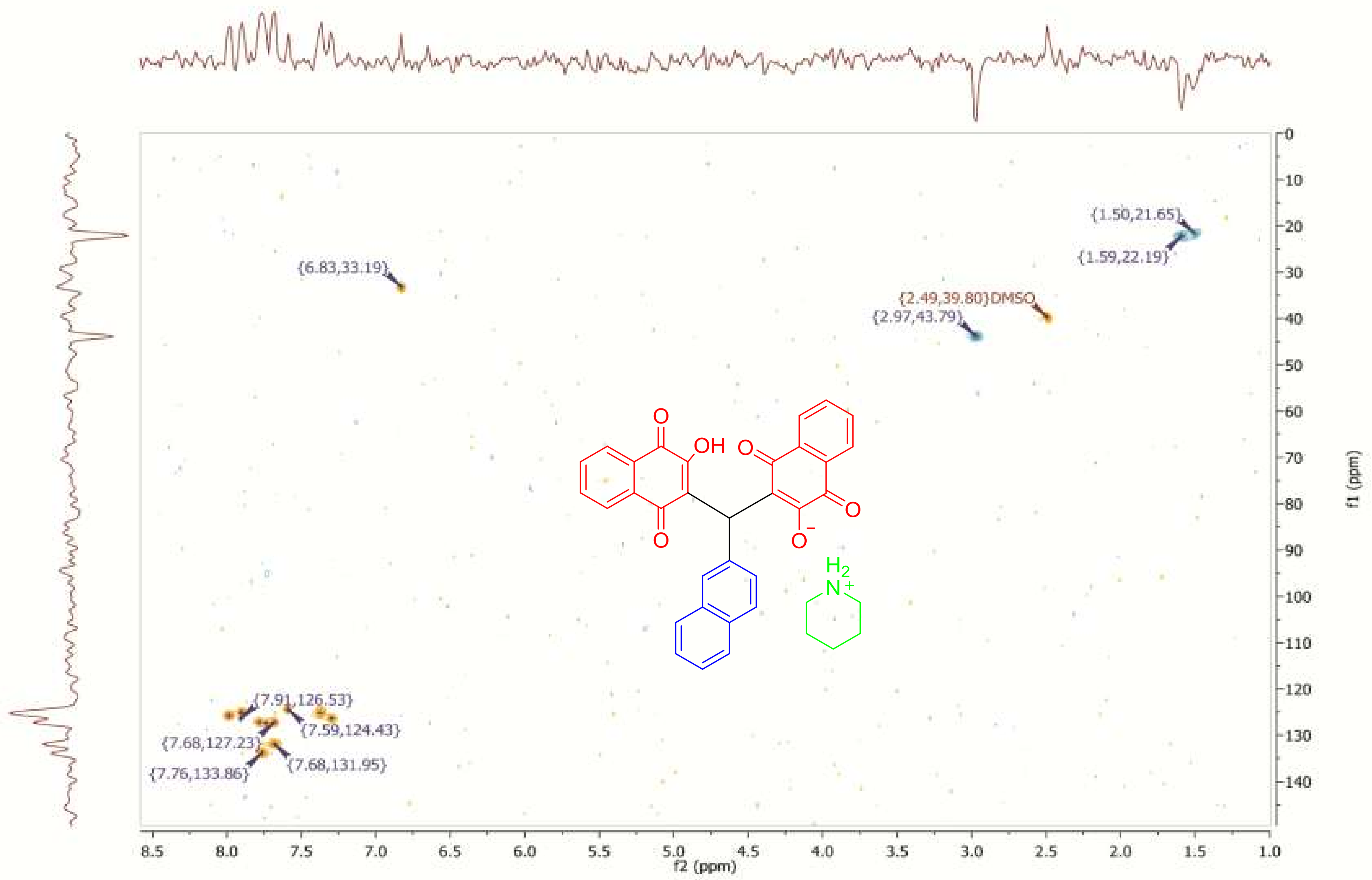

$^{1}H^{13}C$, HSQC-NMR spectrum of piperidin-1-ium 3-((3-hydroxy-1,4-dioxo-1,4-dihydronaphthalen-2-yl)(naphthalen-2-yl)methyl)-1,4-dioxo-1,4-dihydronaphthalen-2-olate

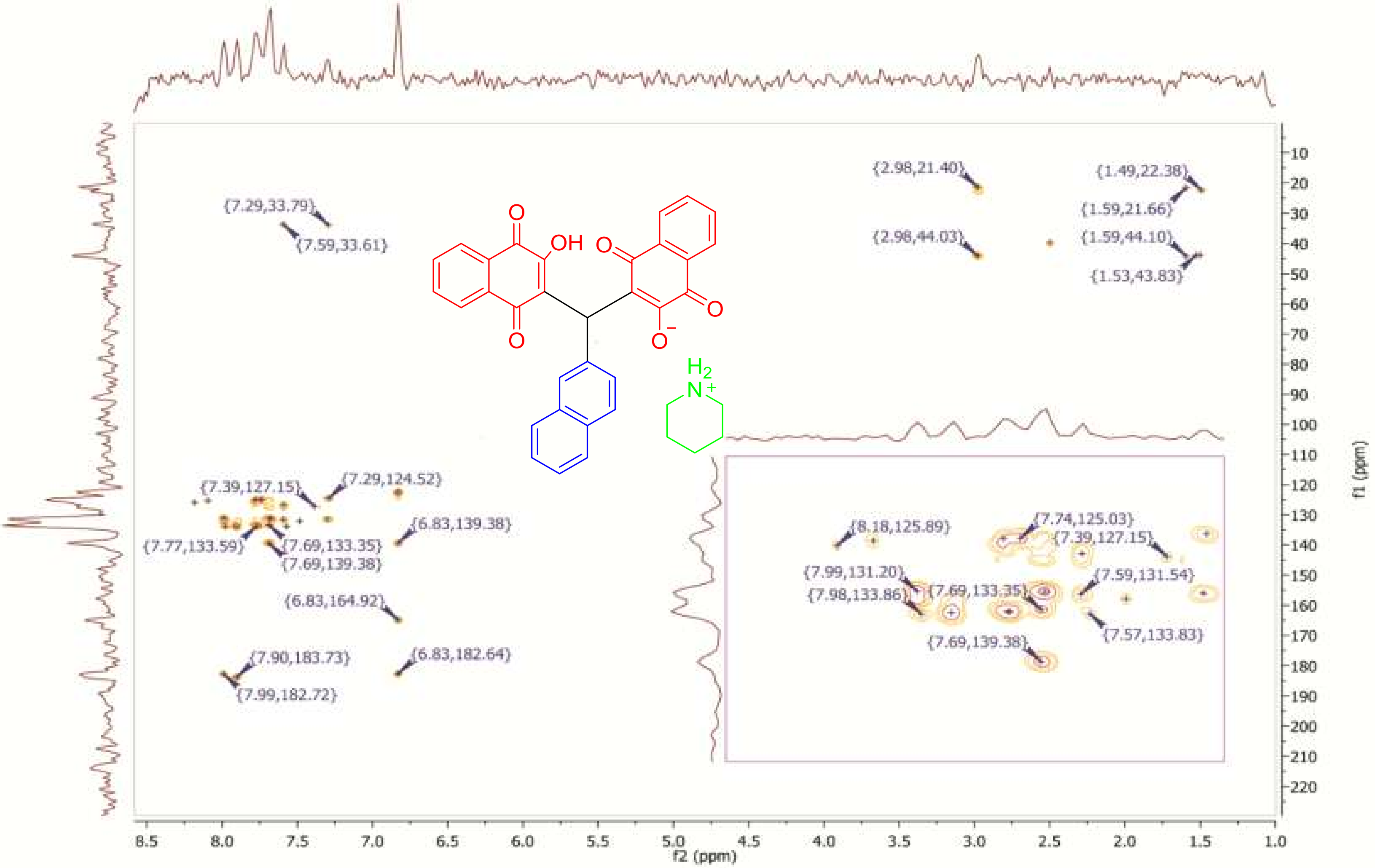

$^{1}H^{13}C$, HMBC-NMR spectrum of piperidin-1-ium 3-((3-hydroxy-1,4-dioxo-1,4-dihydronaphthalen-2-yl)(naphthalen-2-yl)methyl)-1,4-dioxo-1,4-dihydronaphthalen-2-olate

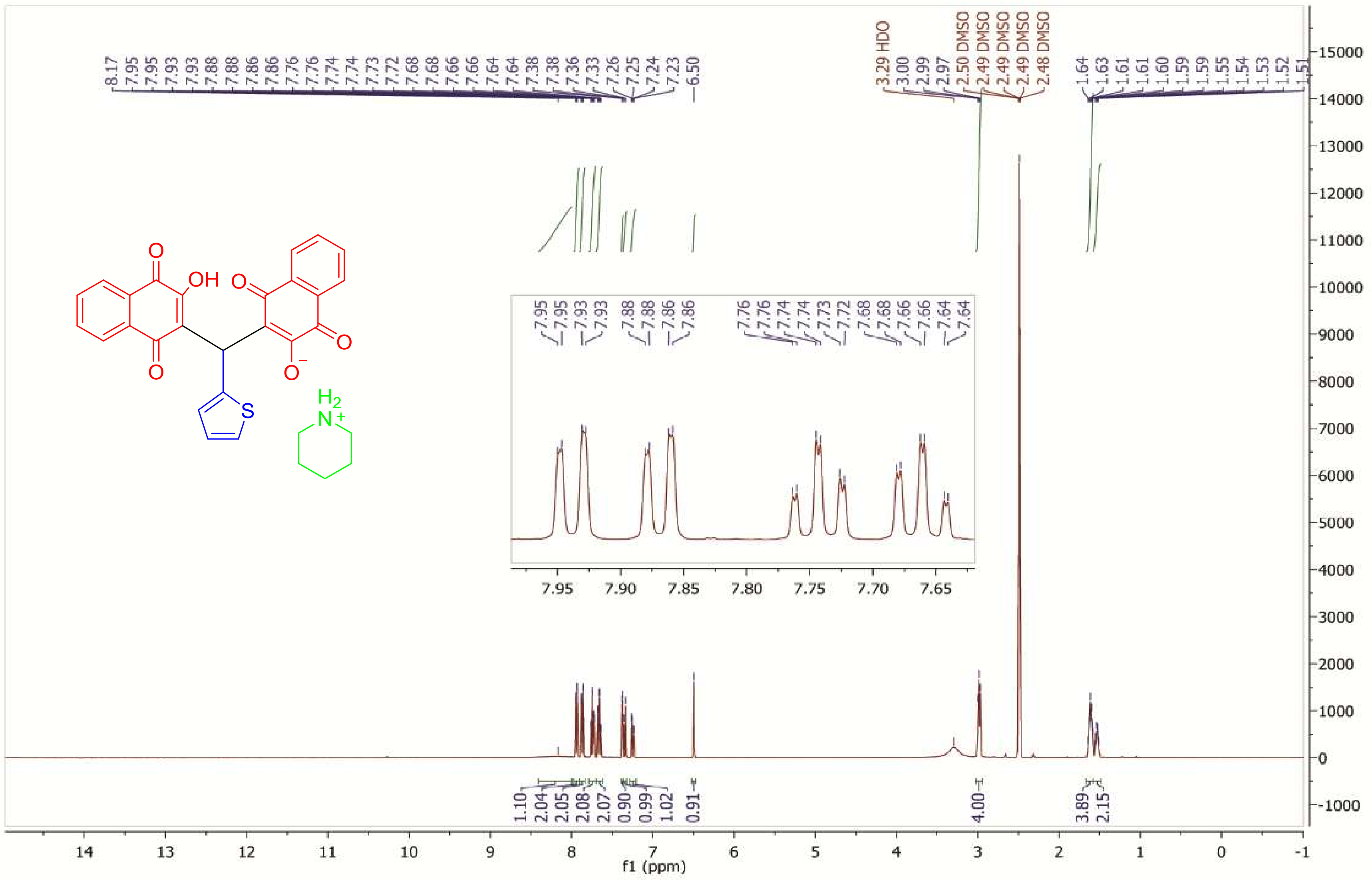

^{1}H NMR spectrum of piperidin-1-ium 3-((3-hydroxy-1,4-dioxo-1,4-dihydronaphthalen-2-yl)(thiophen-2-yl)methyl)-1,4-dioxo-1,4-dihydronaphthalen-2-olate

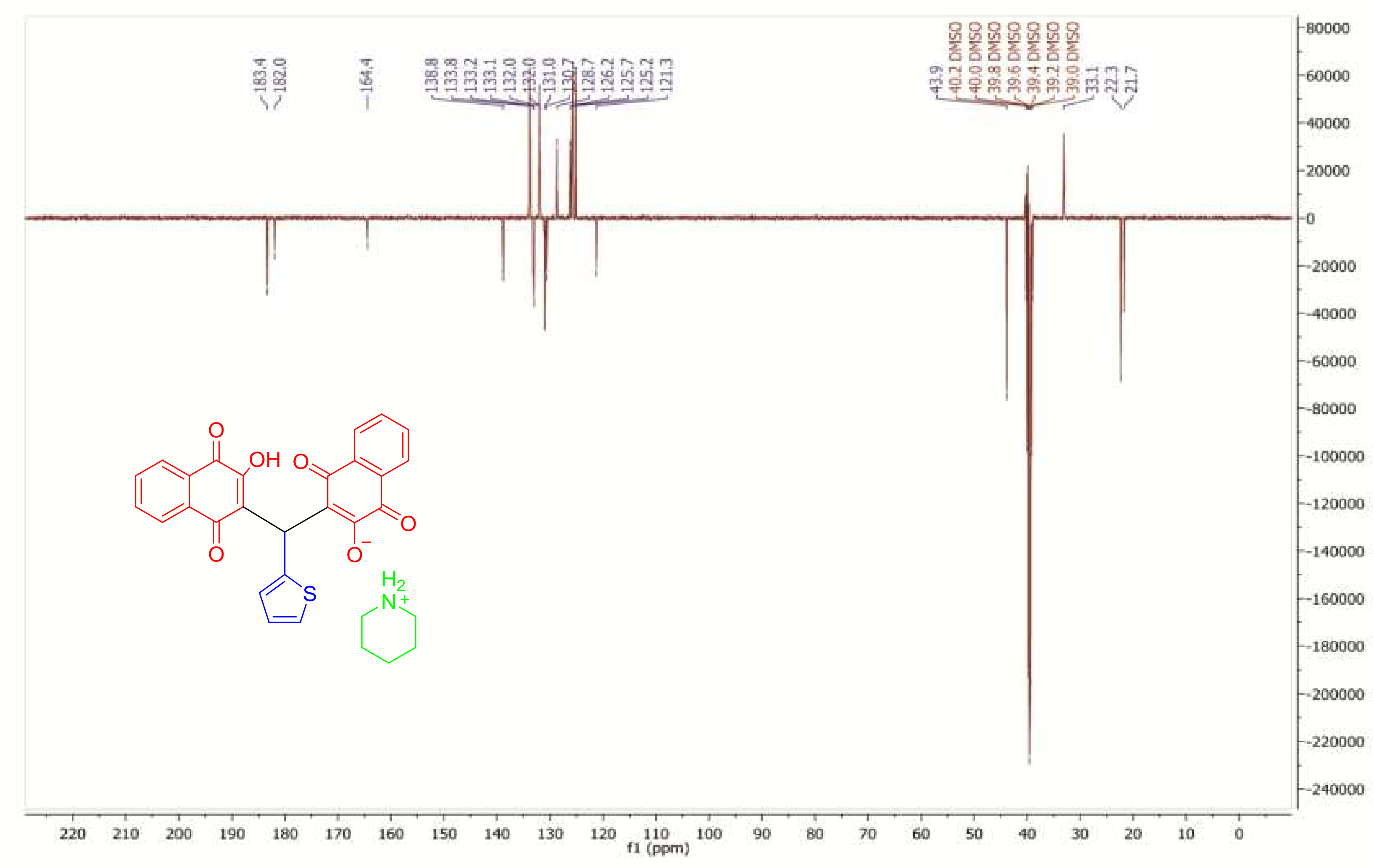

^{13}C NMR (DEPT-135) spectrum of piperidin-1-ium 3-((3-hydroxy-1,4-dioxo-1,4-dihydronaphthalen-2-yl)(thiophen-2-yl)methyl)-1,4-dioxo-1,4-dihydronaphthalen-2-olate

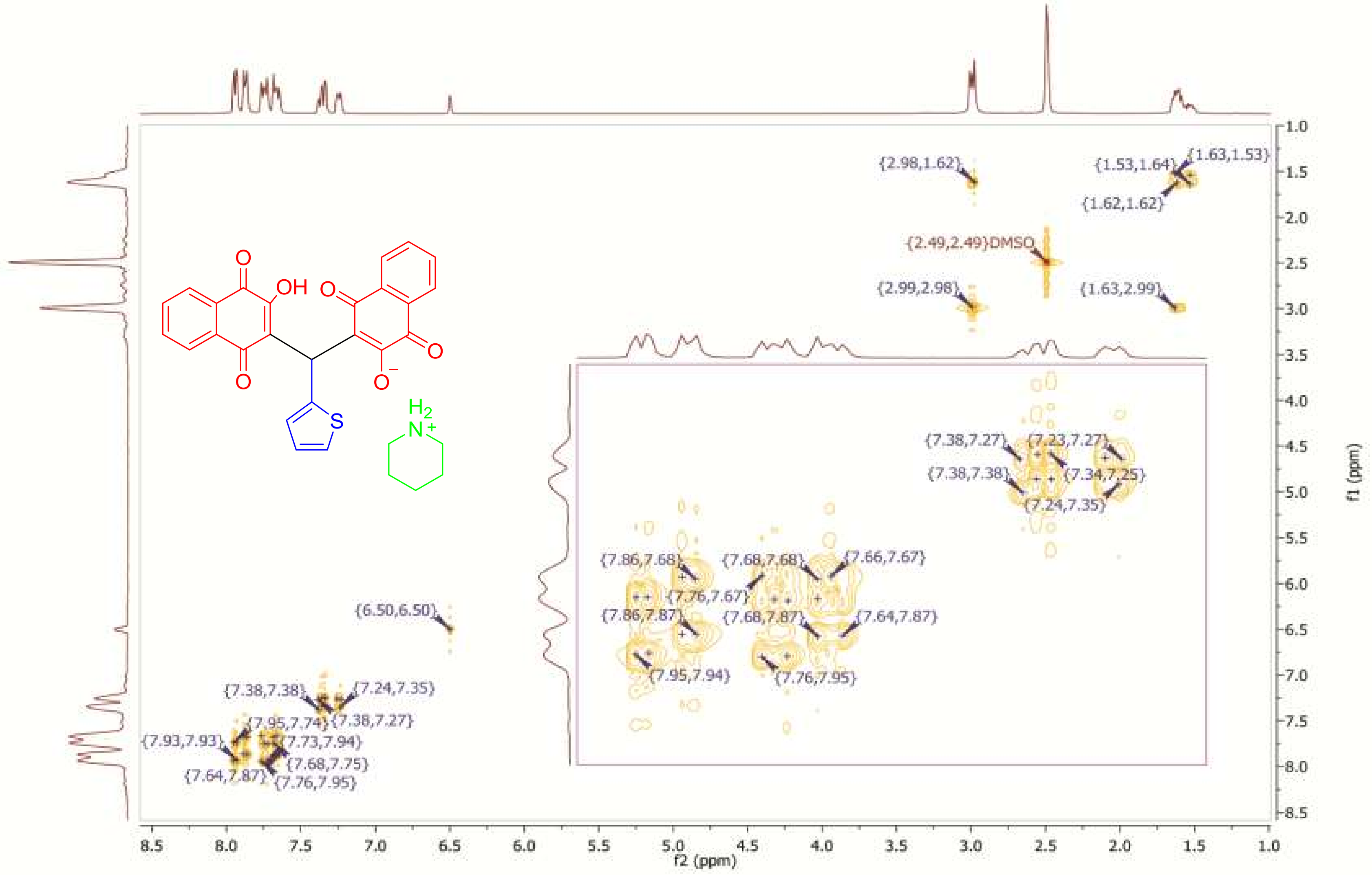

^{1}H^{1}H, COSY-NMR spectrum of piperidin-1-ium 3-((3-hydroxy-1,4-dioxo-1,4-dihydronaphthalen-2-yl)(thiophen-2-yl)methyl)-1,4-dioxo-1,4-dihydronaphthalen-2-olate

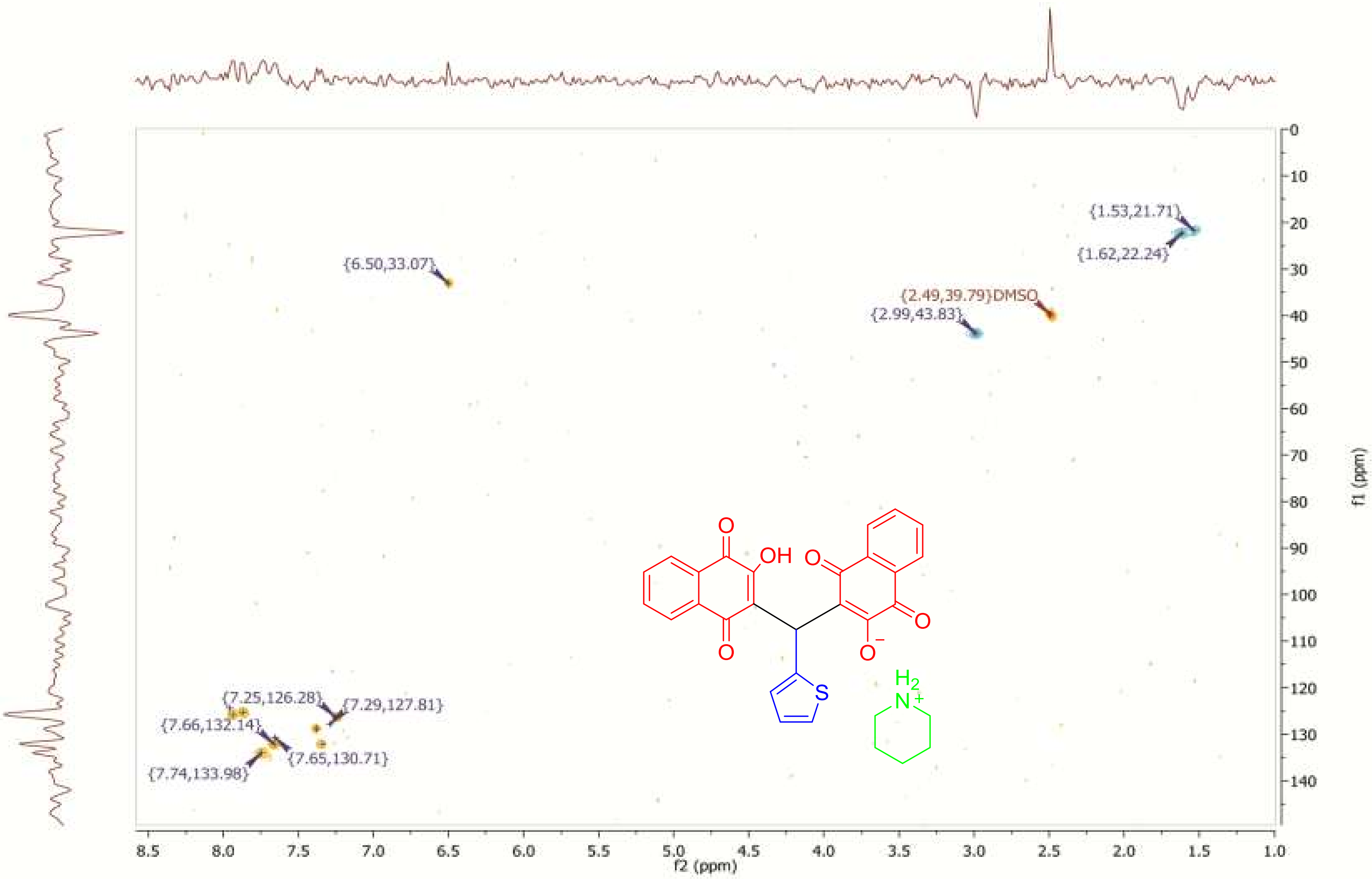

^{1}H^{13}C, HSQC-NMR spectrum of piperidin-1-ium 3-((3-hydroxy-1,4-dioxo-1,4-dihydronaphthalen-2-yl)(thiophen-2-yl)methyl)-1,4-dioxo-1,4-dihydronaphthalen-2-olate

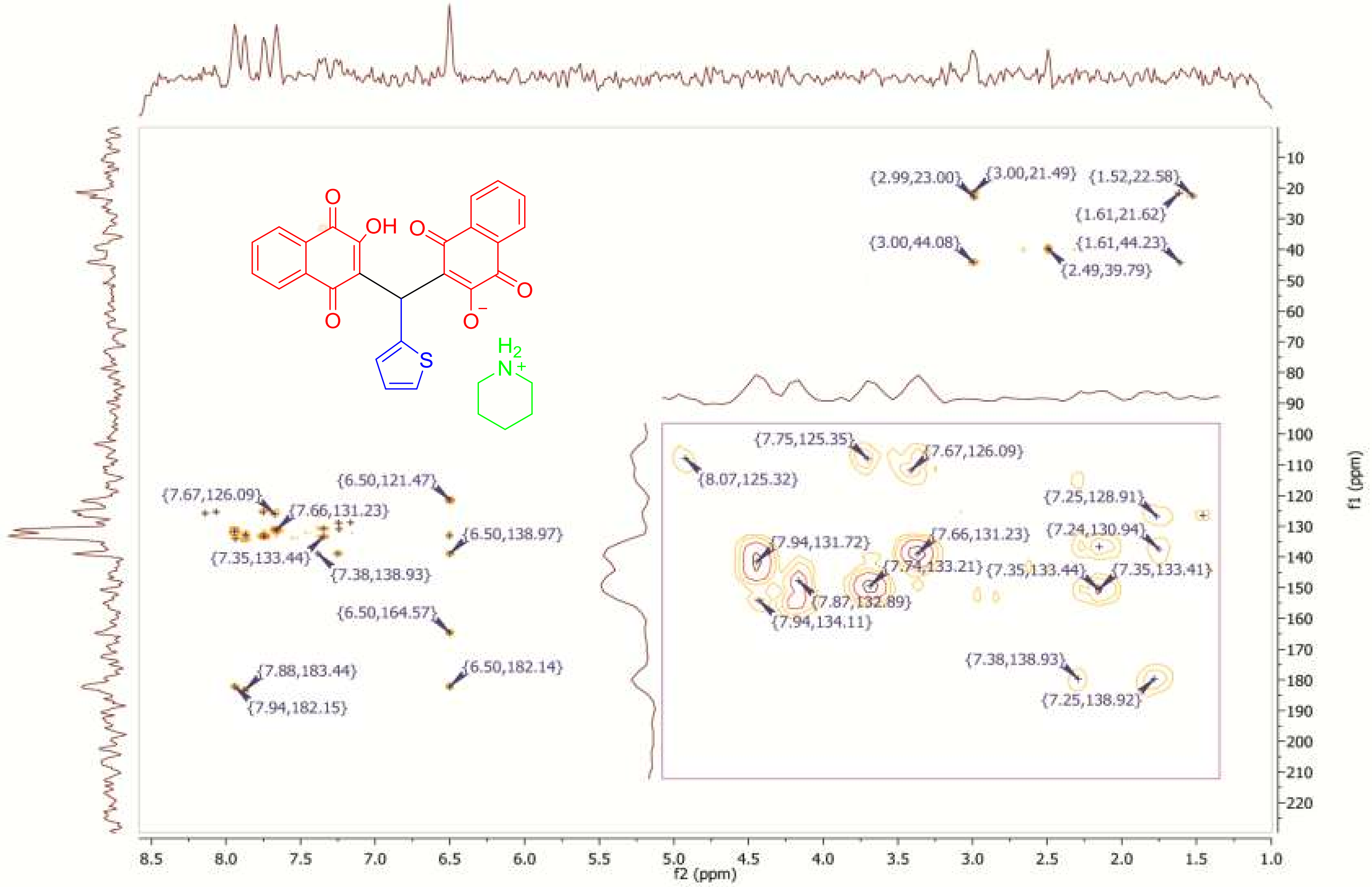

$^{1}H^{13}C$, HMBC-NMR spectrum of piperidin-1-ium 3-((3-hydroxy-1,4-dioxo-1,4-dihydronaphthalen-2-yl)(thiophen-2-yl)methyl)-1,4-dioxo-1,4-dihydronaphthalen-2-olate

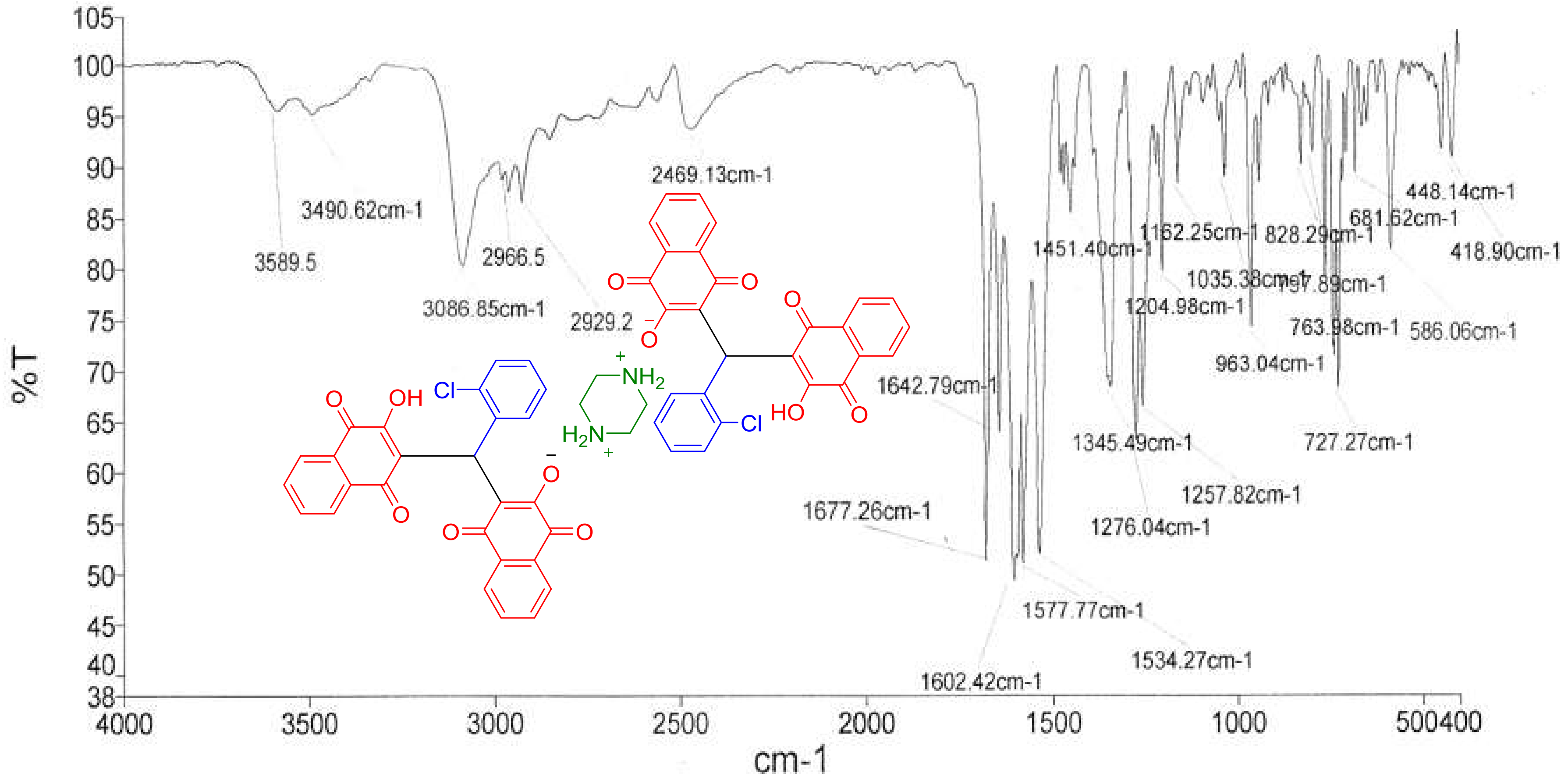

FT-IR spectrum of piperazine-1,4-diium 3-((2-chlorophenyl)(3-hydroxy-1,4-dioxo-1,4-dihydronaphthalen-2-yl)methyl)-1,4-dioxo-1,4-dihydronaphthalen-2-olate

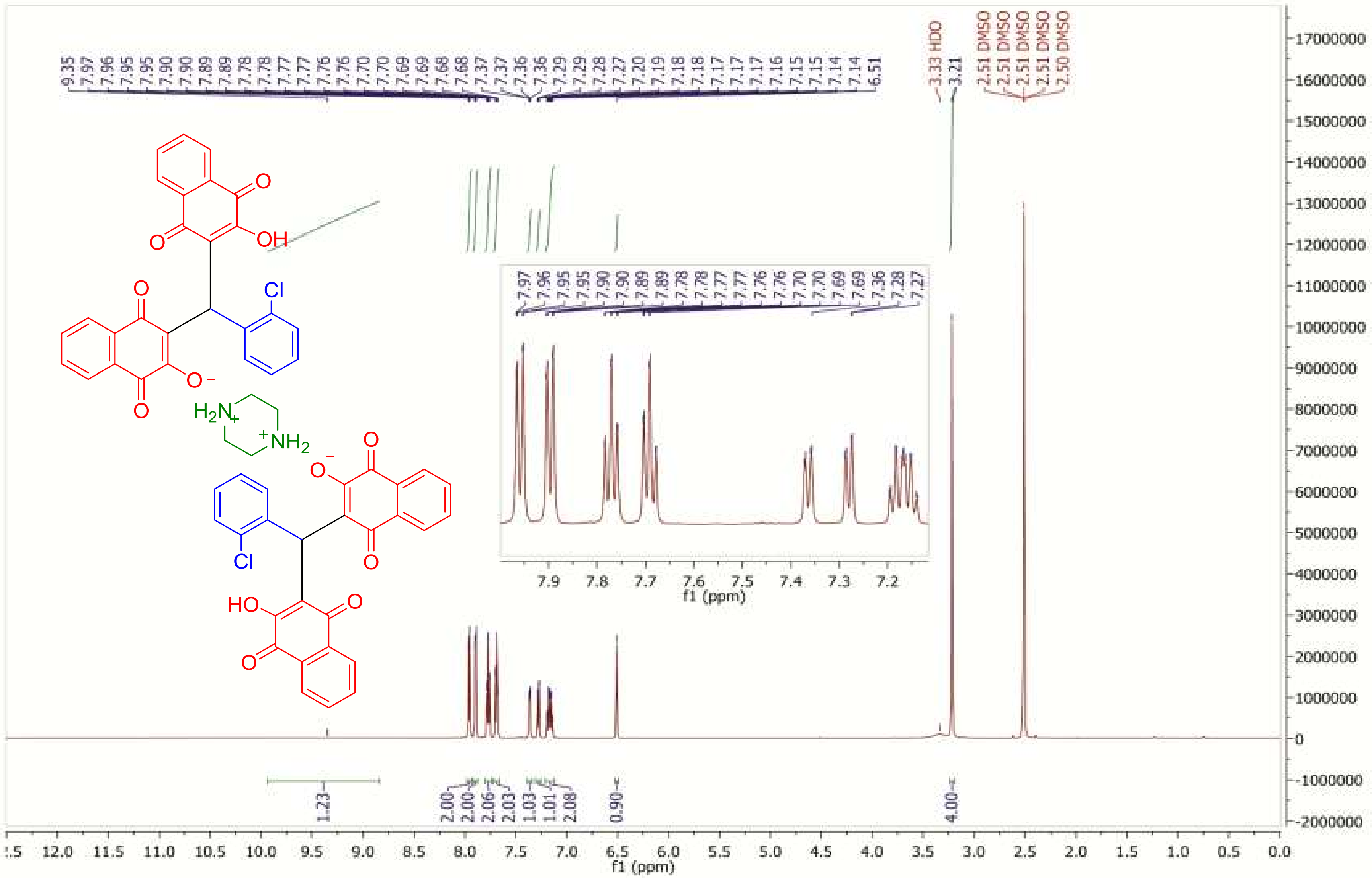

^{1}H NMR spectrum of piperazine-1,4-diium 3-((2-chlorophenyl)(3-hydroxy-1,4-dioxo-1,4-dihydronaphthalen-2-yl)methyl)-1,4-dioxo-1,4-dihydronaphthalen-2-olate

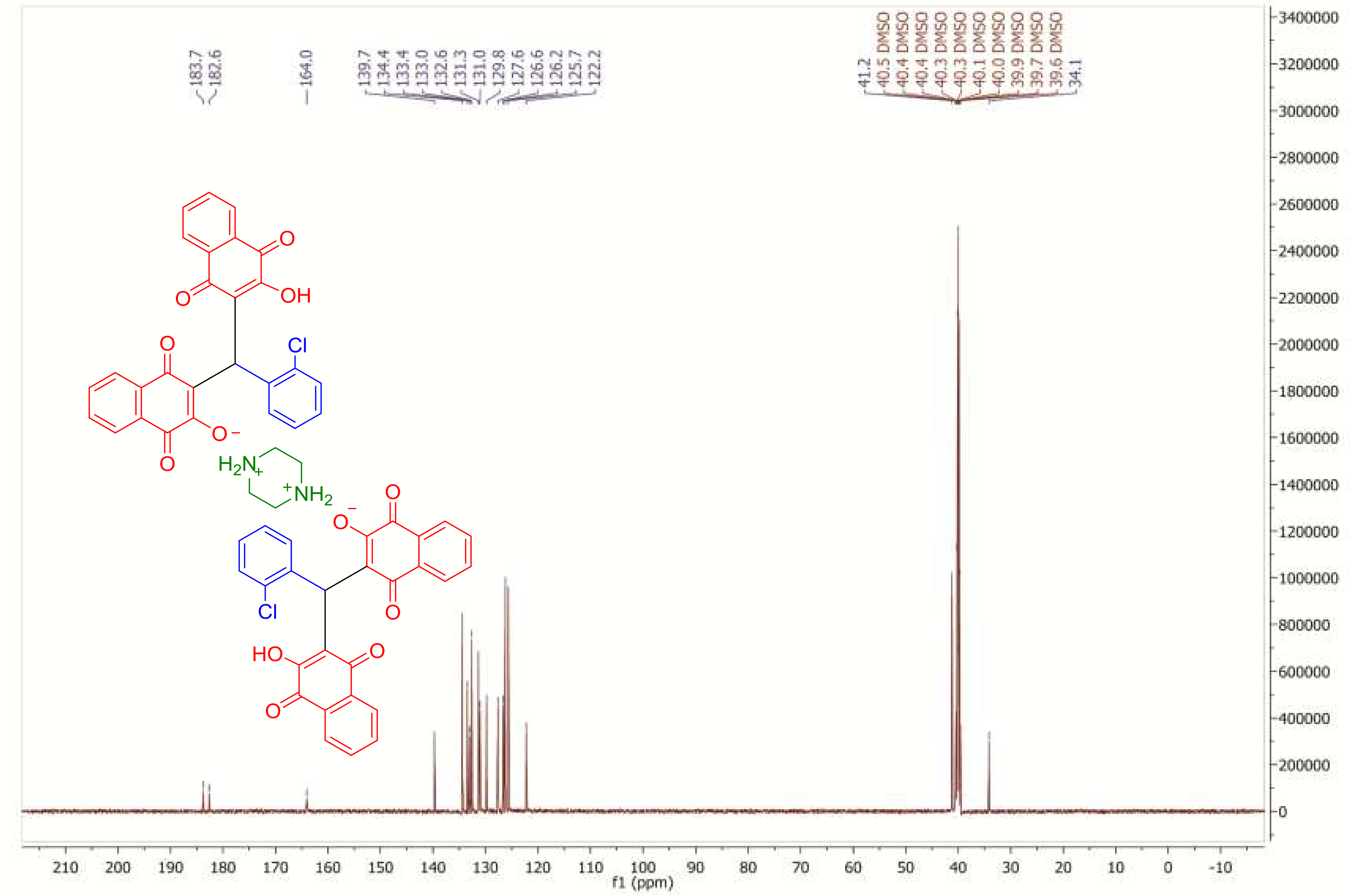

^{13}C NMR spectrum of piperazine-1,4-diium 3-((2-chlorophenyl)(3-hydroxy-1,4-dioxo-1,4-dihydronaphthalen-2-yl)methyl)-1,4-dioxo-1,4-dihydronaphthalen-2-olate

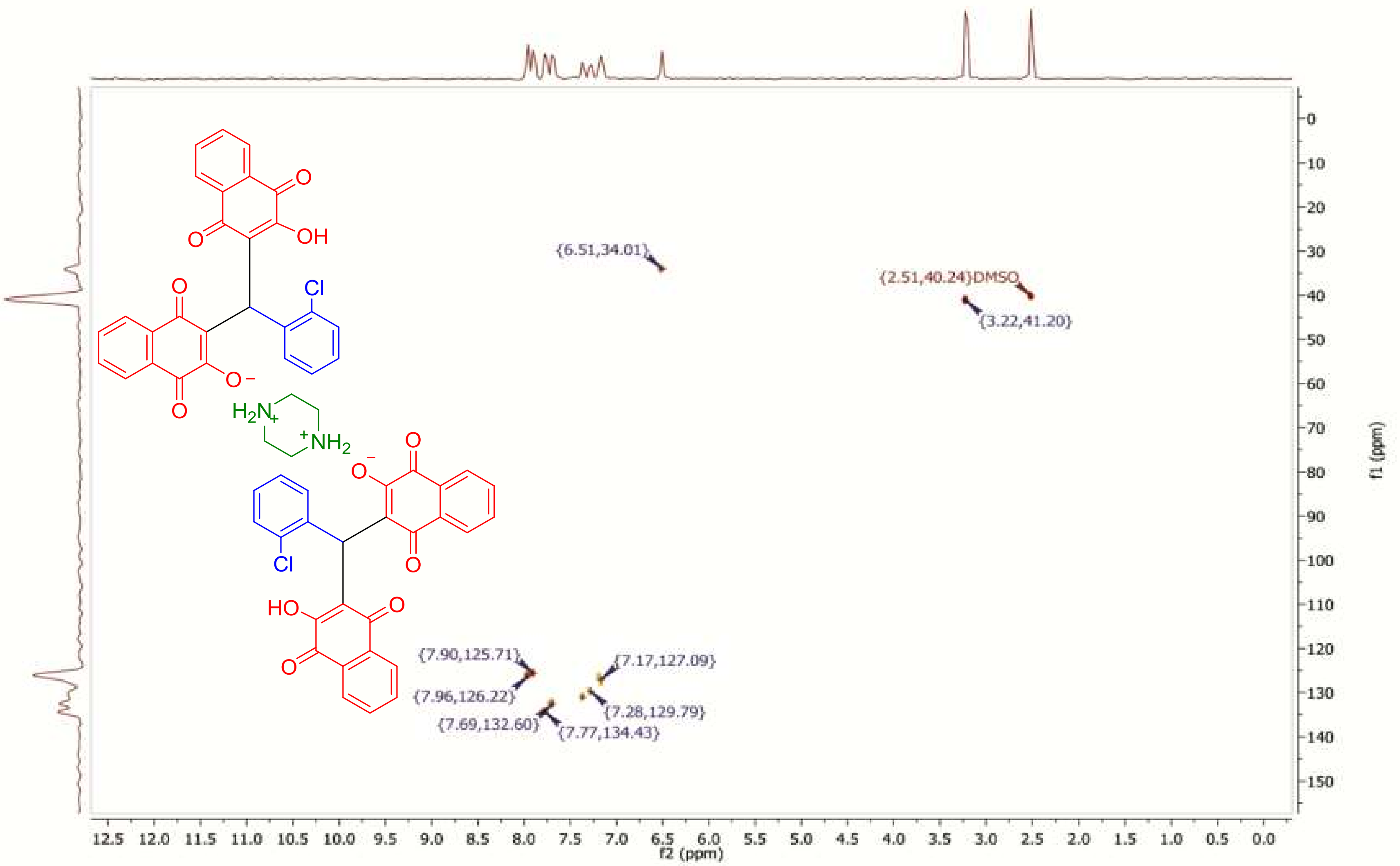

$^{1}H^{13}C$, HSQC-NMR spectrum of piperazine-1,4-diium 3-((2-chlorophenyl)(3-hydroxy-1,4-dioxo-1,4-dihydronaphthalen-2-yl)methyl)-1,4-dioxo-1,4-dihydronaphthalen-2-olate

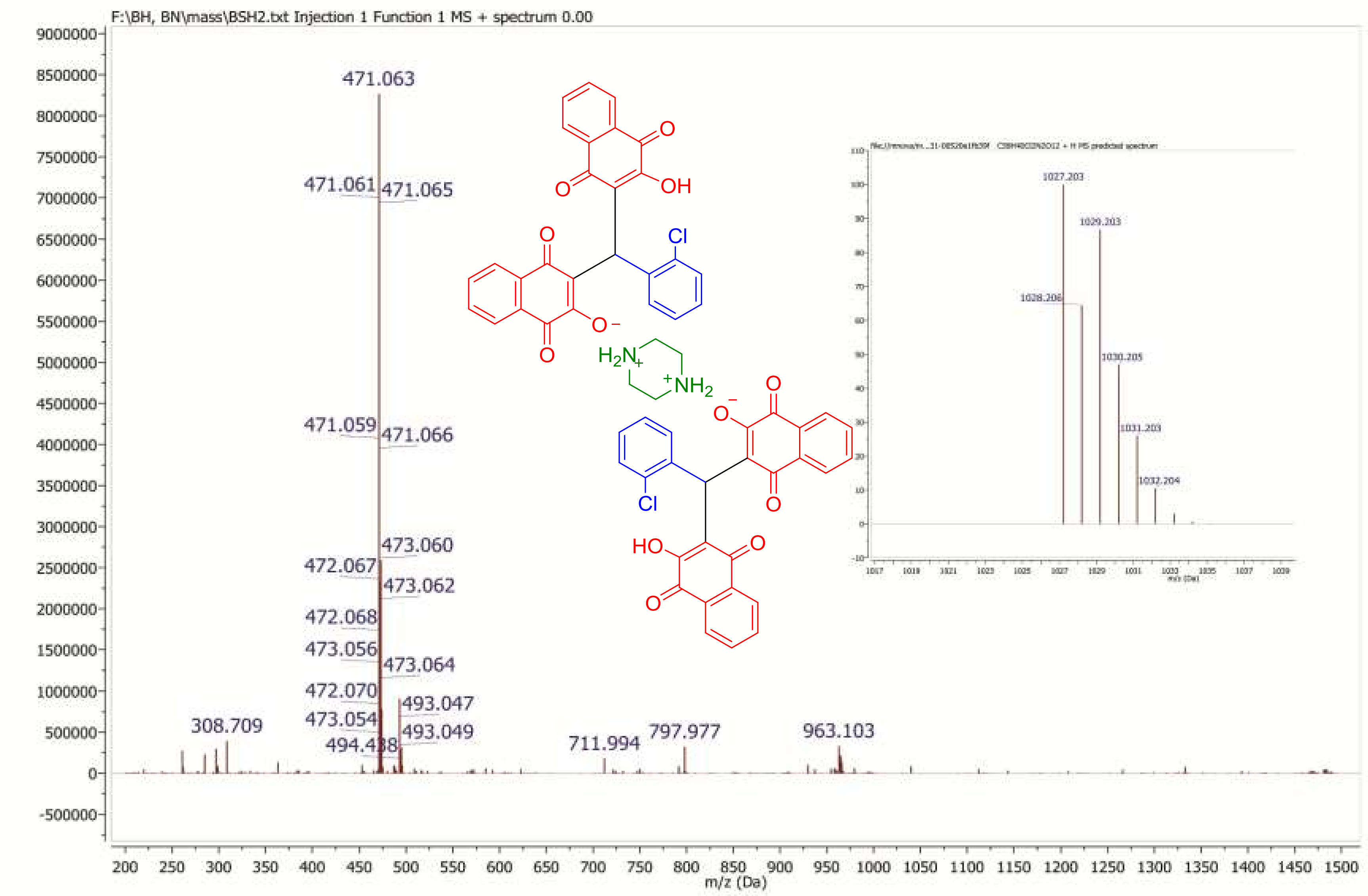

HR-Mass spectrum of piperazine-1,4-diium 3-((2-chlorophenyl)(3-hydroxy-1,4-dioxo-1,4-dihydronaphthalen-2-yl)methyl)-1,4-dioxo-1,4-dihydronaphthalen-2-olate

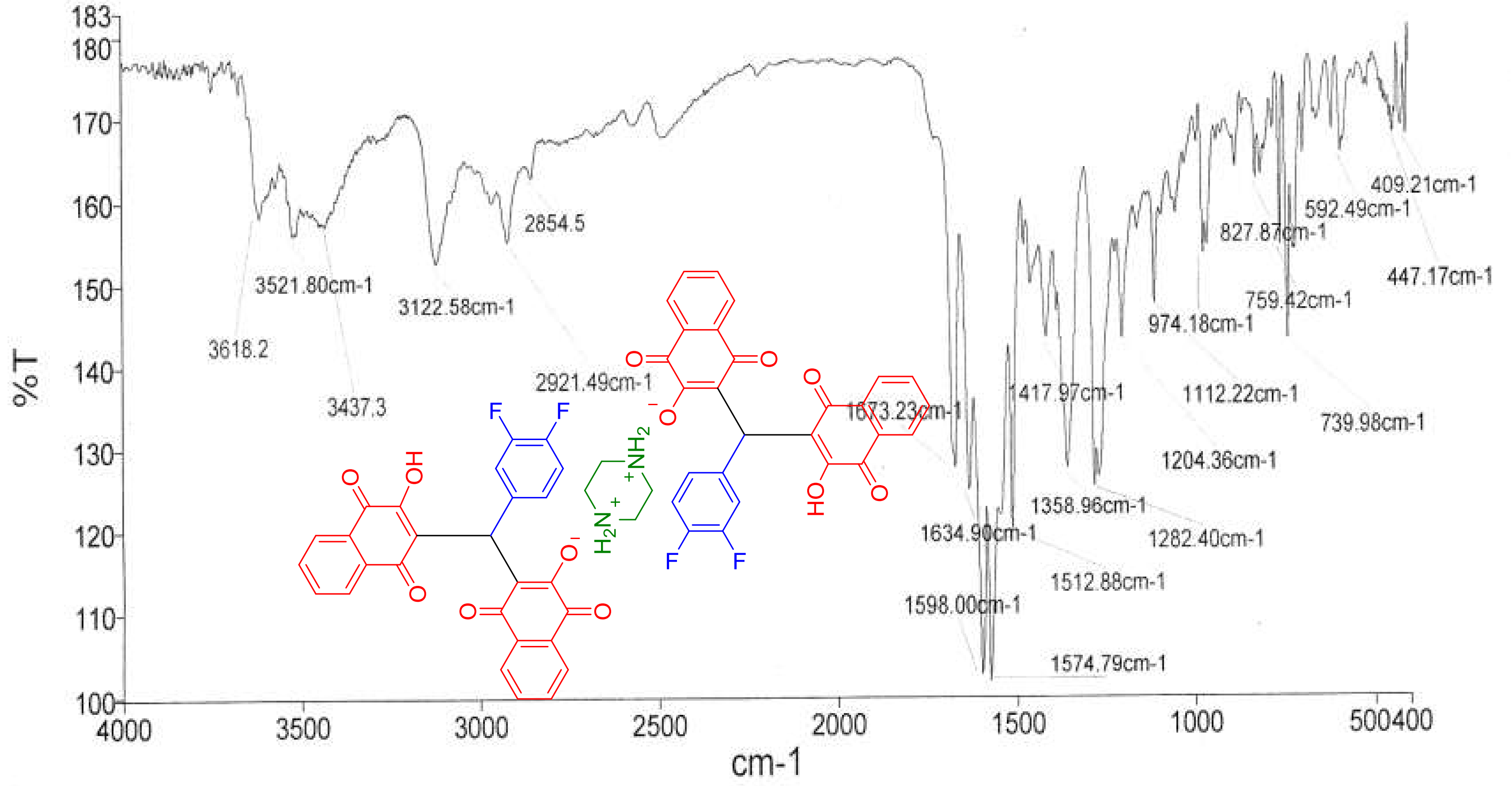

FT-IR spectrum of piperazine-1,4-diium 3-((3,4-difluorophenyl)(1,4-dioxo-1,4-dihydronaphthalen-2-yl)methyl)-1,4-dioxo-1,4-dihydronaphthalen-2-olate

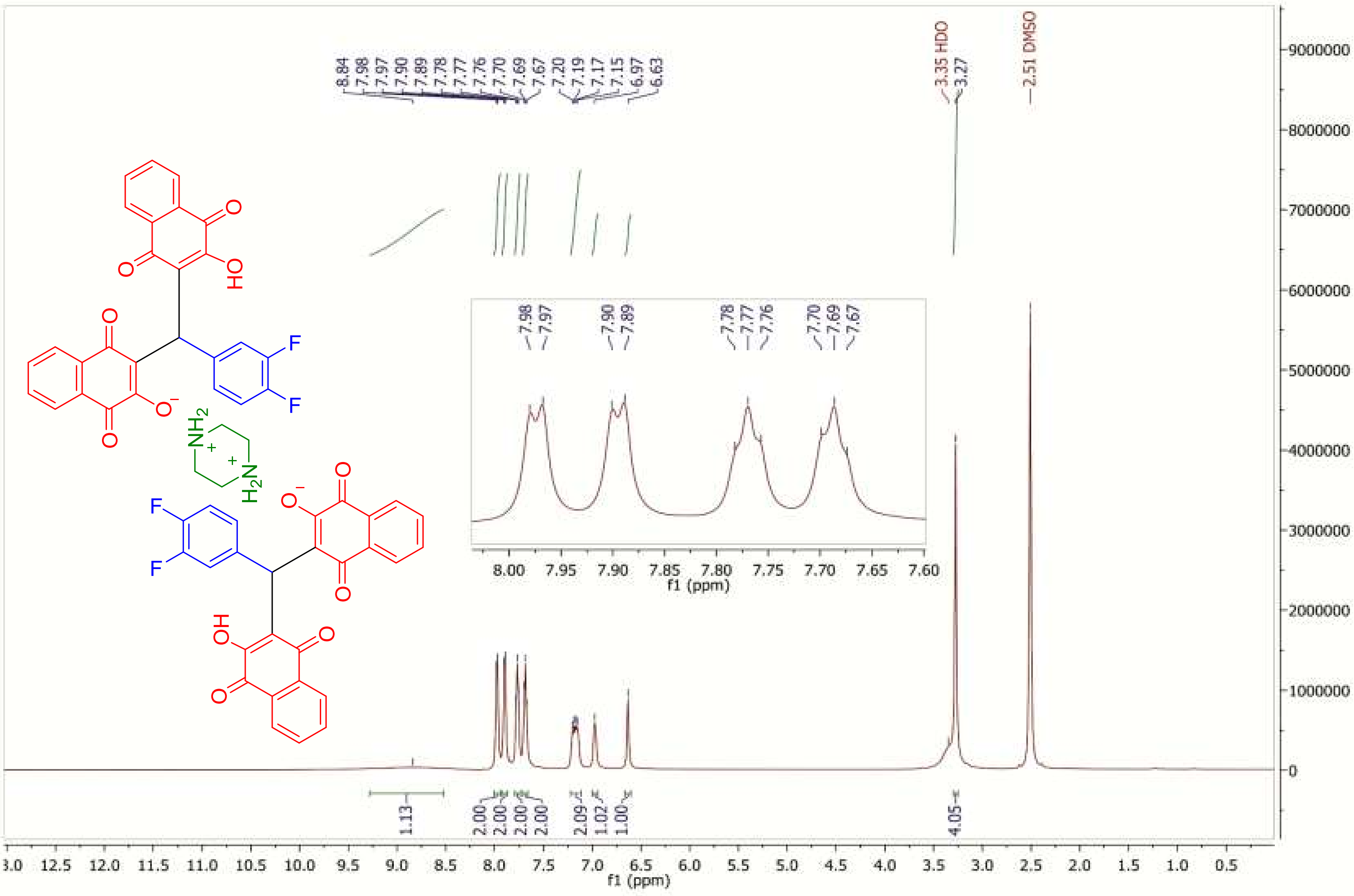

^{1}H NMR spectrum of piperazine-1,4-diium 3-((3,4-difluorophenyl)(1,4-dioxo-1,4-dihydronaphthalen-2-yl)methyl)-1,4-dioxo-1,4-dihydronaphthalen-2-olate

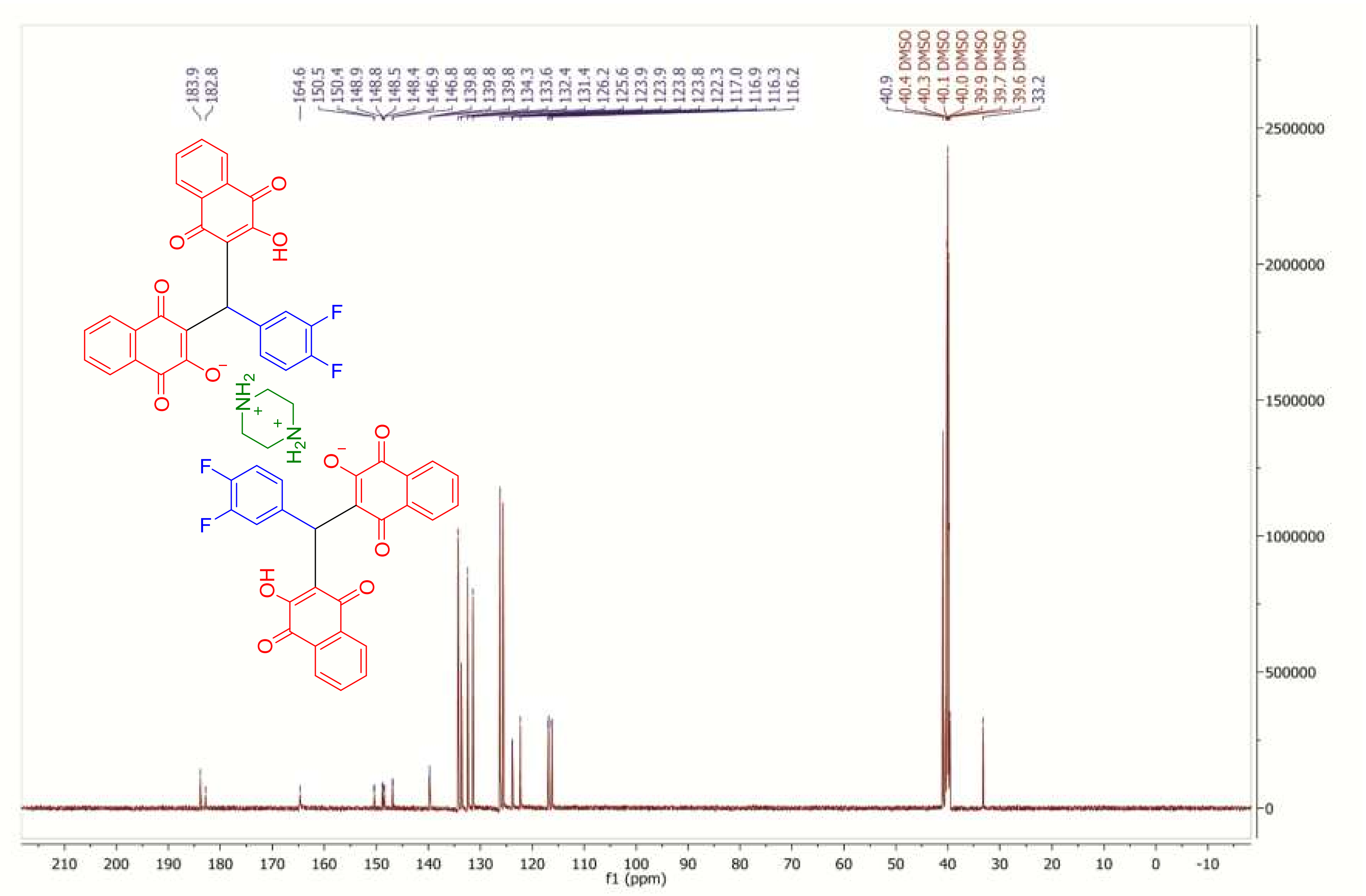

^{13}C NMR spectrum of piperazine-1,4-diium 3-((3,4-difluorophenyl)(1,4-dioxo-1,4-dihydronaphthalen-2-yl)methyl)-1,4-dioxo-1,4-dihydronaphthalen-2-olate

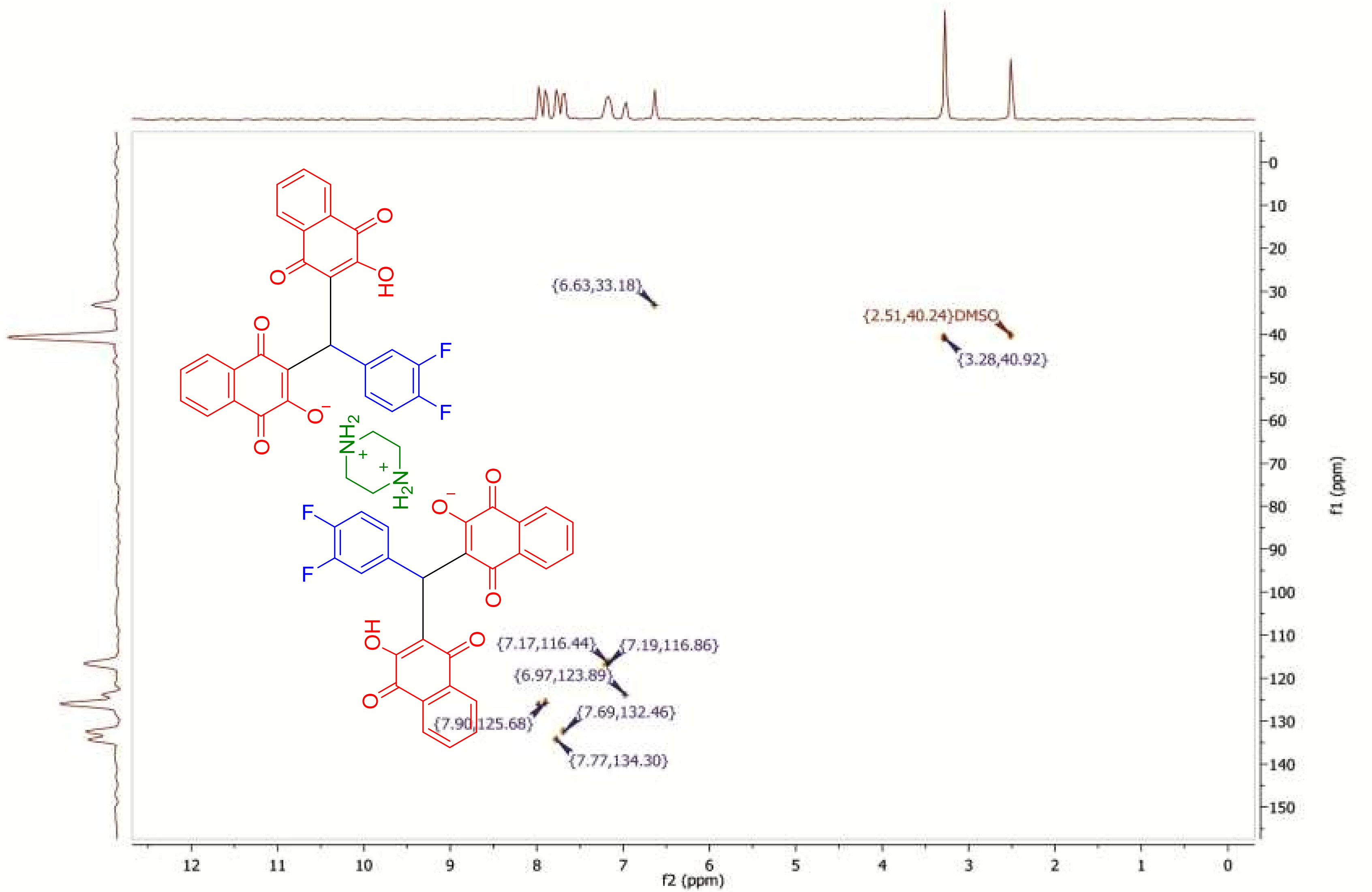

^{1}H^{13}C, HSQC-NMR spectrum of piperazine-1,4-diium 3-((3,4-difluorophenyl)(1,4-dioxo-1,4-dihydronaphthalen-2-yl)methyl)-1,4-dioxo-1,4-dihydronaphthalen-2-olate

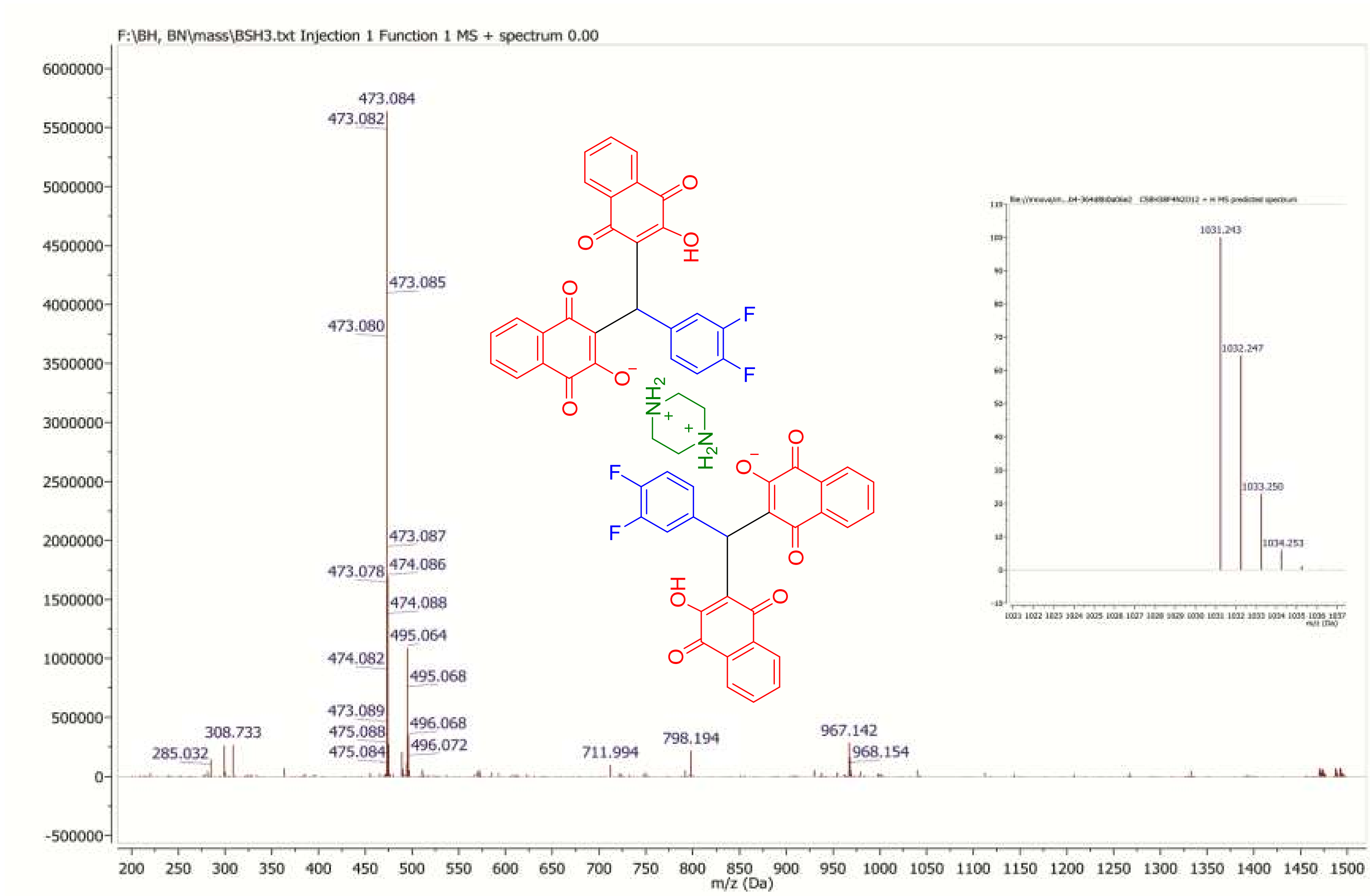

HR-Mass spectrum of piperazine-1,4-diium 3-((3,4-difluorophenyl)(1,4-dioxo-1,4-dihydronaphthalen-2-yl)methyl)-1,4-dioxo-1,4-dihydronaphthalen-2-olate

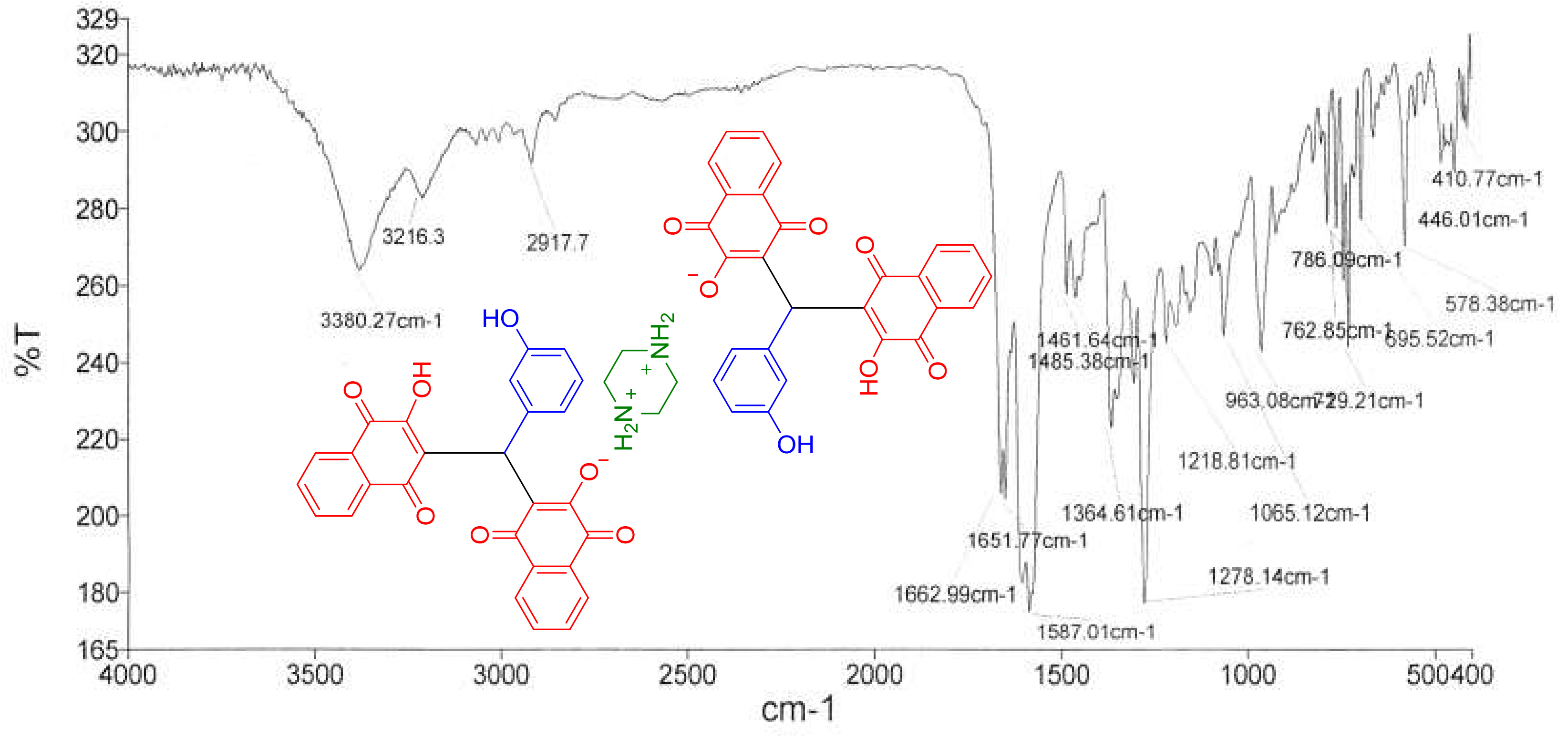

FT-IR spectrum of piperazine-1,4-diium 3-((1,4-dioxo-1,4-dihydronaphthalen-2-yl)(3-hydroxyphenyl)methyl)-1,4-dioxo-1,4-dihydronaphthalen-2-olate

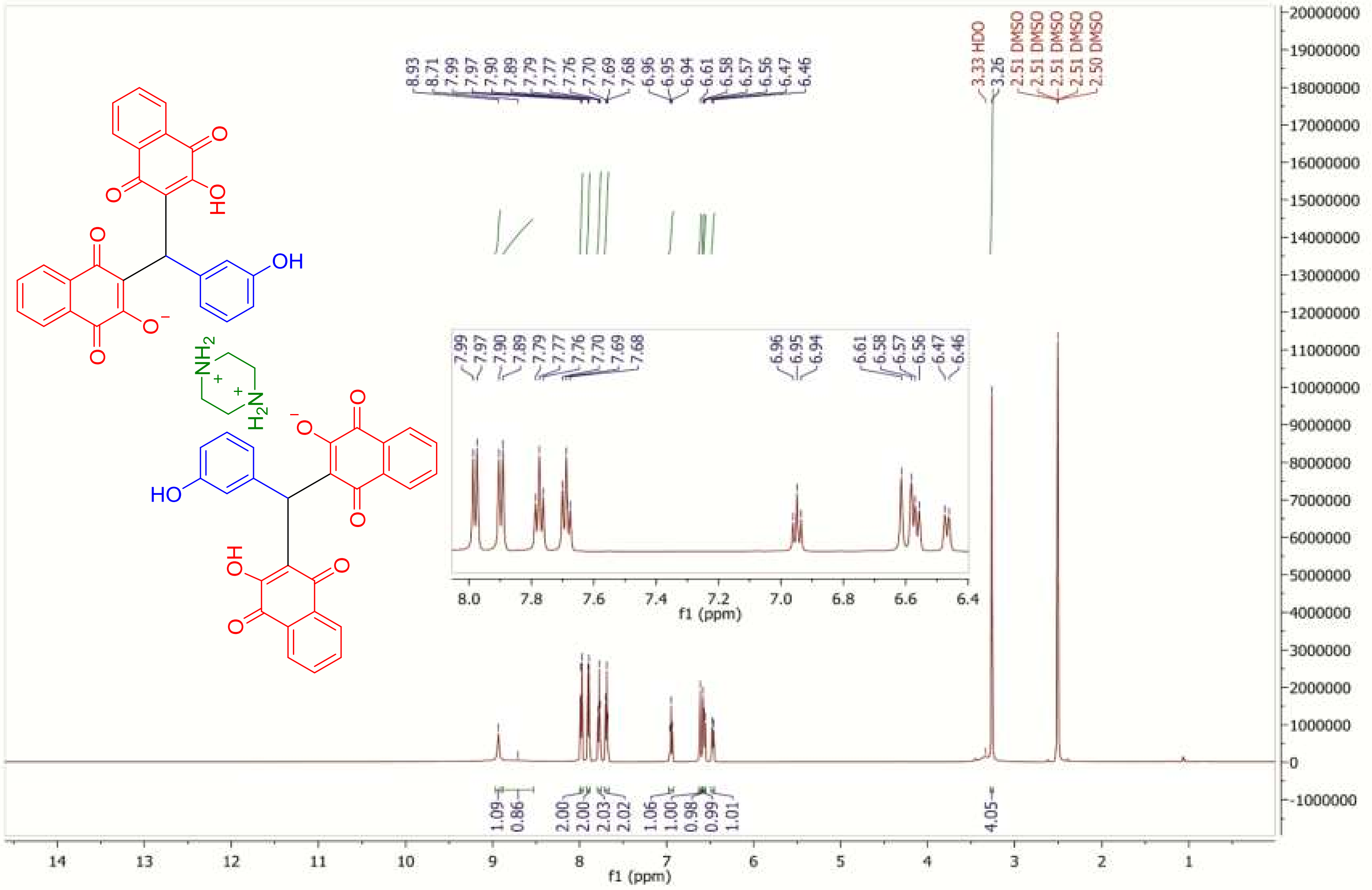

^{1}H NMR spectrum of piperazine-1,4-diium 3-((1,4-dioxo-1,4-dihydronaphthalen-2-yl)(3-hydroxyphenyl)methyl)-1,4-dioxo-1,4-dihydronaphthalen-2-olate

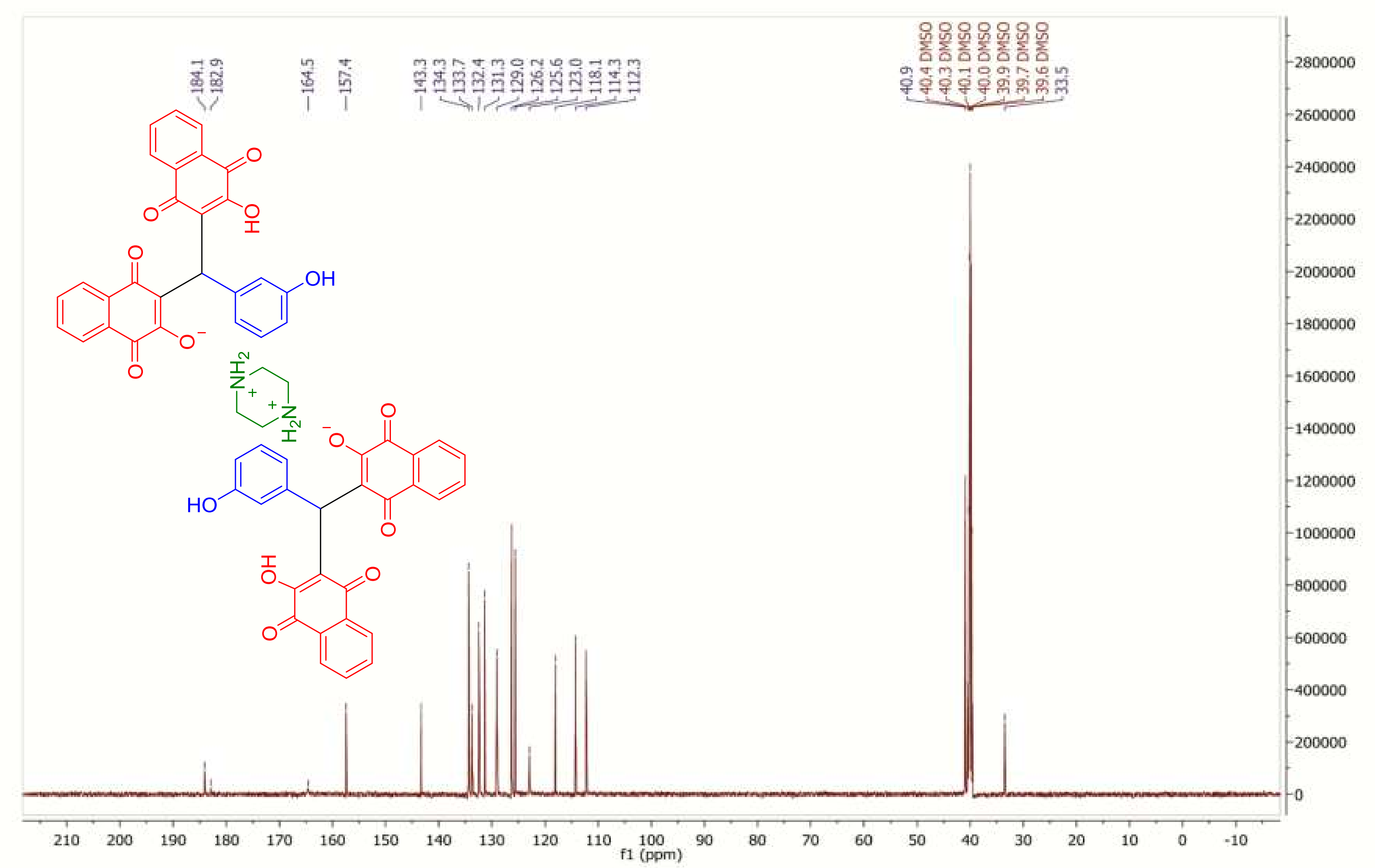

^{13}C NMR spectrum of piperazine-1,4-diium 3-((1,4-dioxo-1,4-dihydronaphthalen-2-yl)(3-hydroxyphenyl)methyl)-1,4-dioxo-1,4-dihydronaphthalen-2-olate

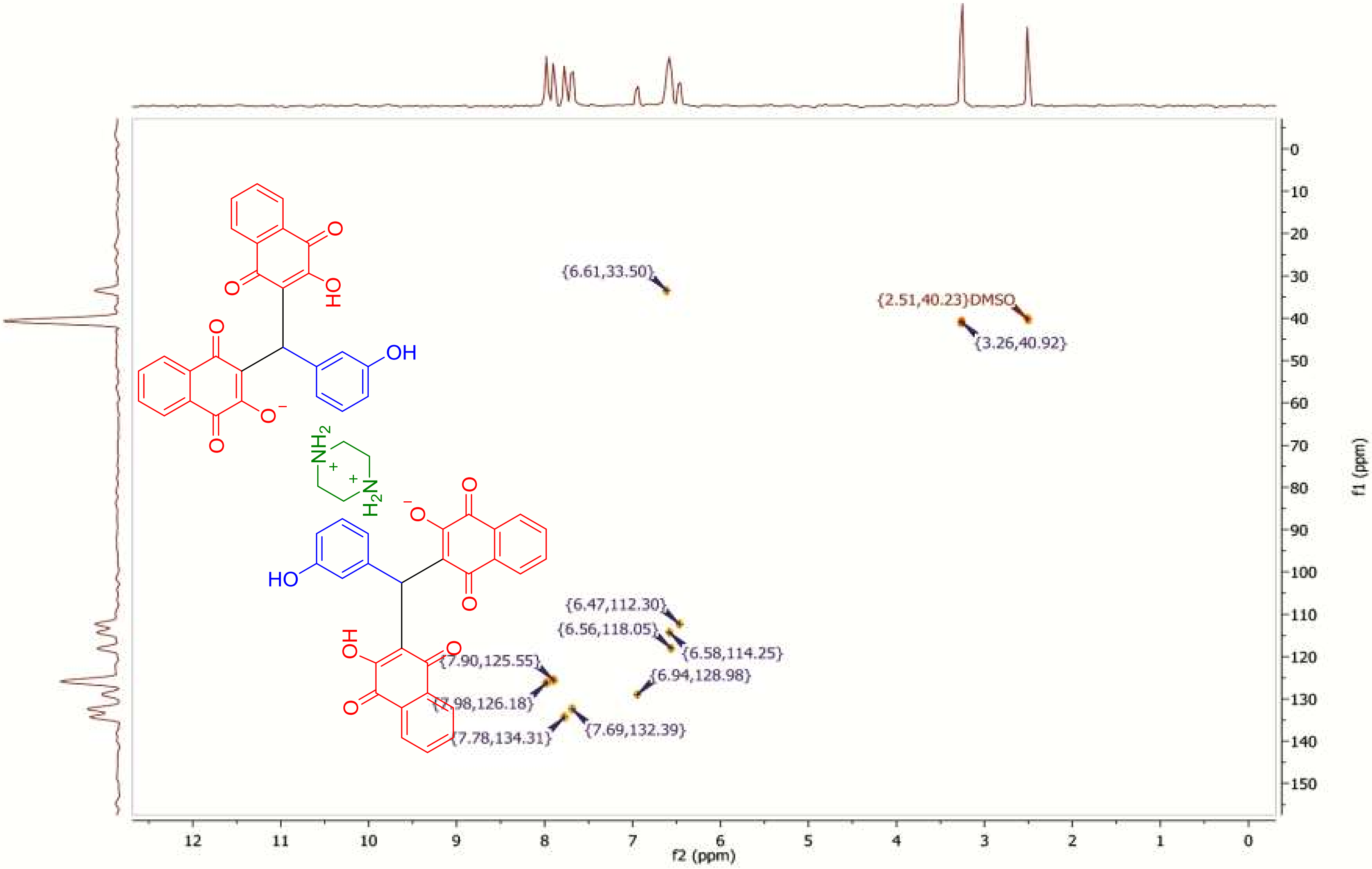

^{1}H^{13}C, HSQC-NMR spectrum of piperazine-1,4-diium 3-((1,4-dioxo-1,4-dihydronaphthalen-2-yl)(3-hydroxyphenyl)methyl)-1,4-dioxo-1,4-dihydronaphthalen-2-olate

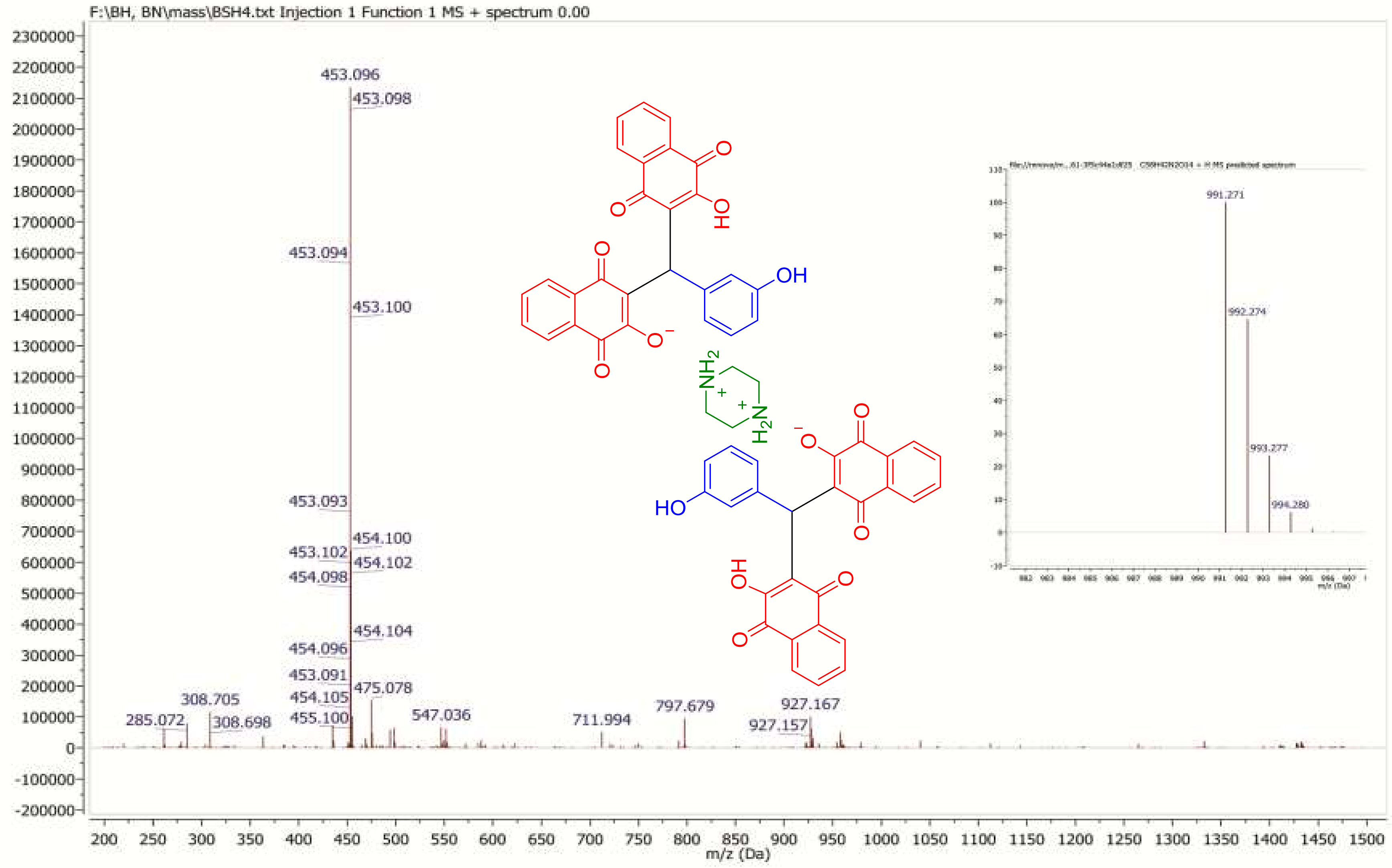

HR-Mass spectrum of piperazine-1,4-diium 3-((1,4-dioxo-1,4-dihydronaphthalen-2-yl)(3-hydroxyphenyl)methyl)-1,4-dioxo-1,4-dihydronaphthalen-2-olate

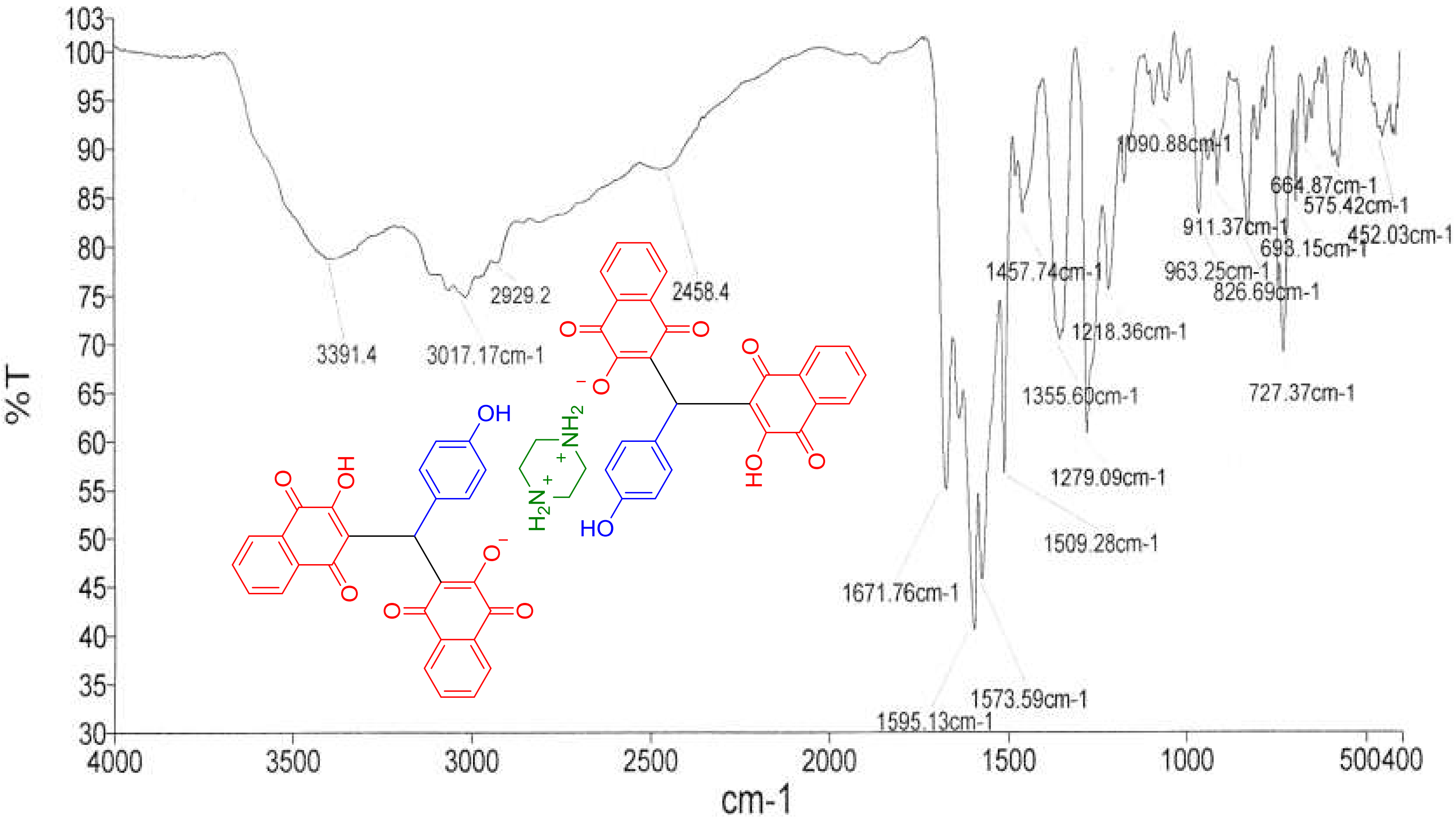

FT-IR spectrum ofpiperazine-1,4-diium 3-((1,4-dioxo-1,4-dihydronaphthalen-2-yl)(4-hydroxyphenyl)methyl)-1,4-dioxo-1,4-dihydronaphthalen-2-olate

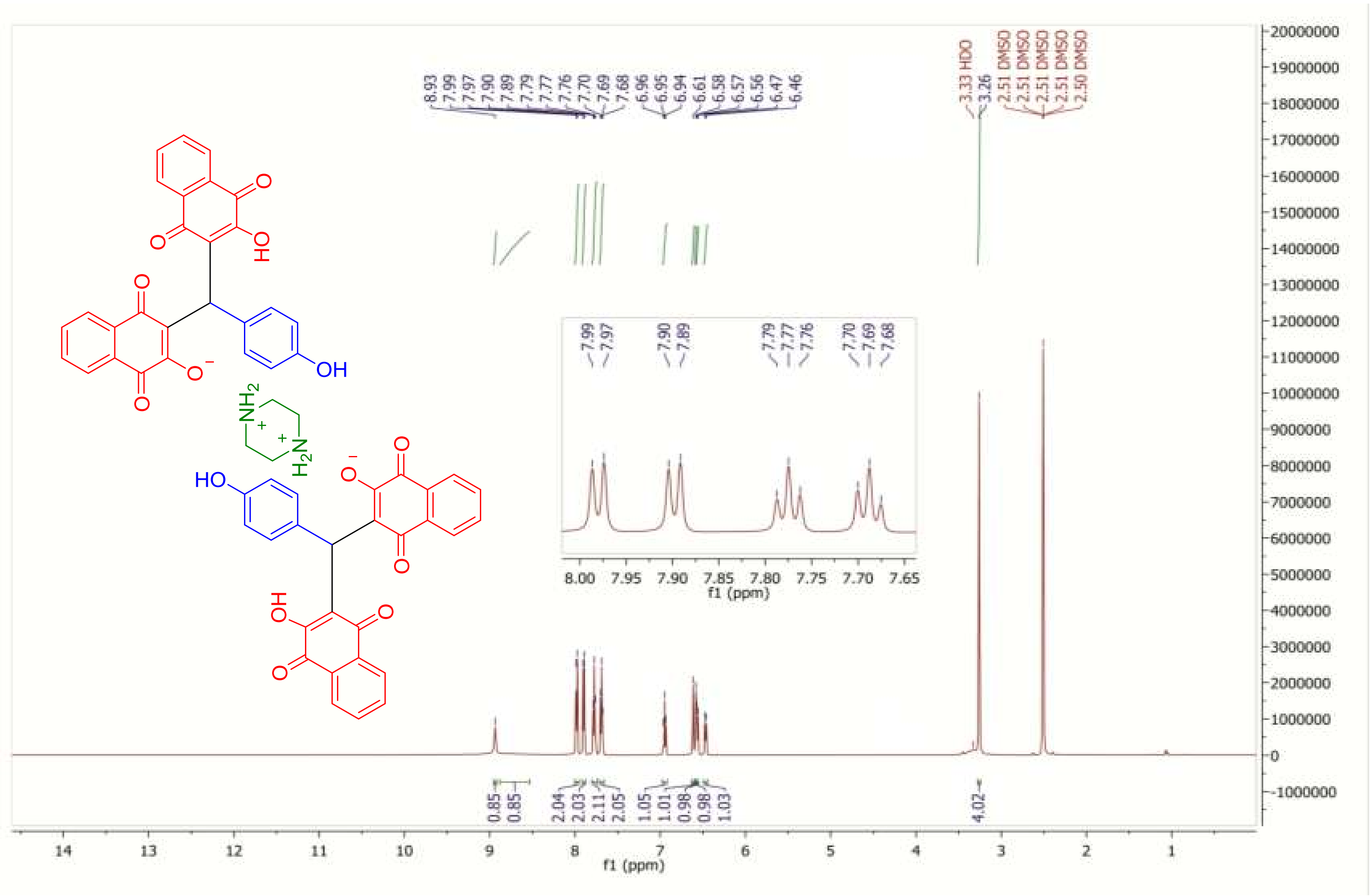

^{1}HNMR spectrum of piperazine-1,4-diium 3-((1,4-dioxo-1,4-dihydronaphthalen-2-yl)(4-hydroxyphenyl)methyl)-1,4-dioxo-1,4-dihydronaphthalen-2-olate

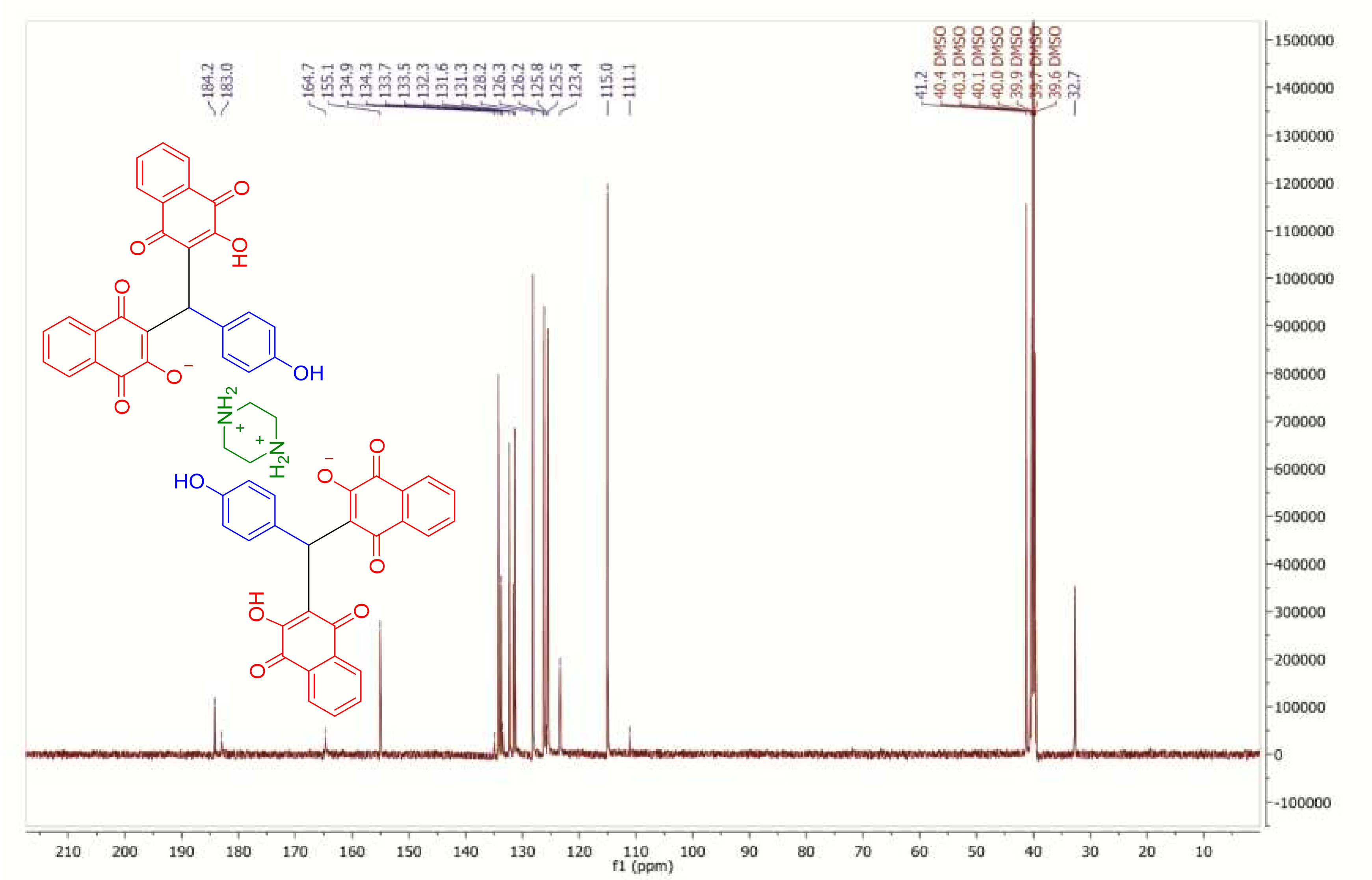

^{13}C NMR spectrum of piperazine-1,4-diium 3-((1,4-dioxo-1,4-dihydronaphthalen-2-yl)(4-hydroxyphenyl)methyl)-1,4-dioxo-1,4-dihydronaphthalen-2-olate

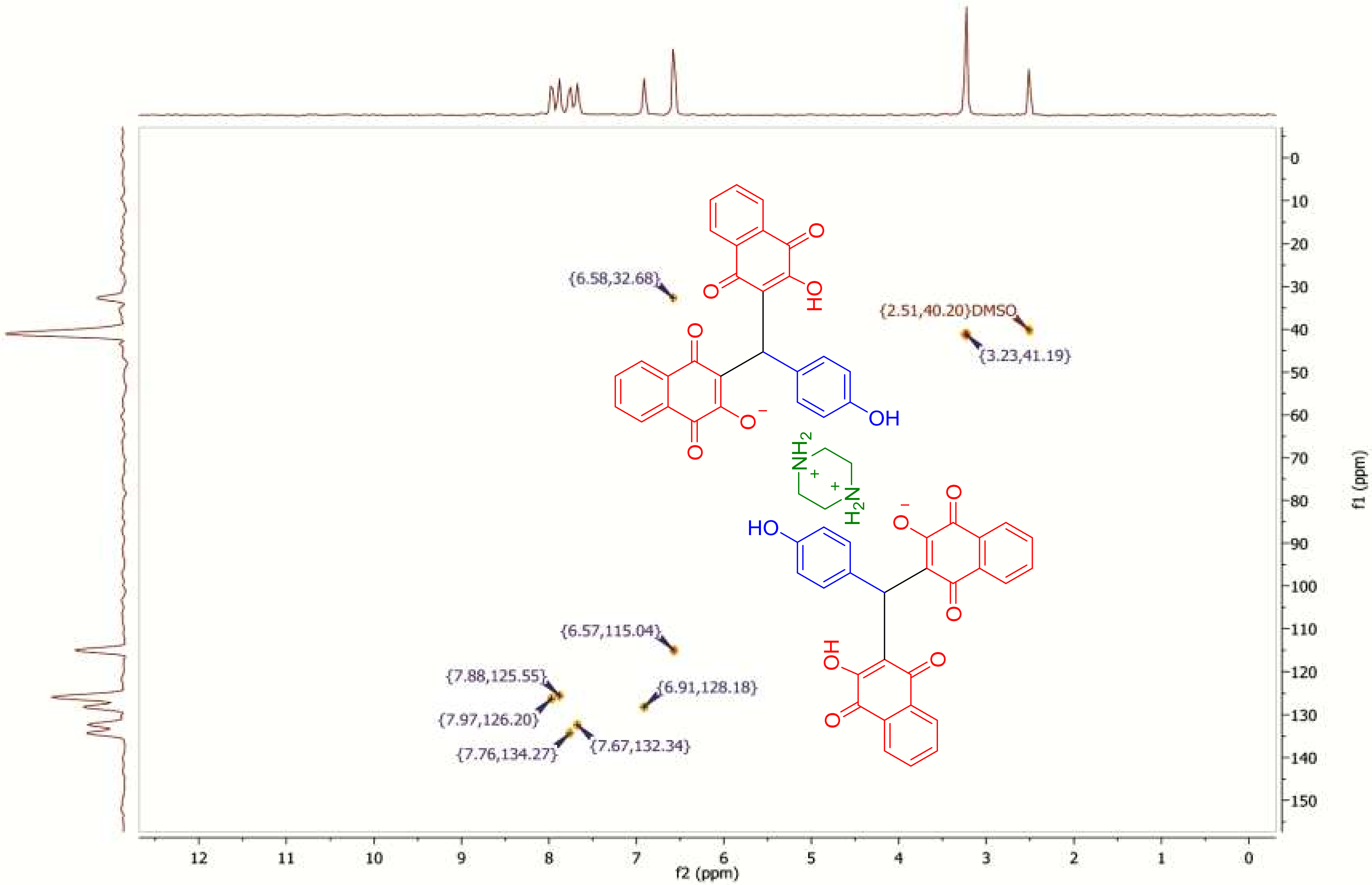

$^{1}H^{13}C$, HSQC-NMR spectrum of piperazine-1,4-diium 3-((1,4-dioxo-1,4-dihydronaphthalen-2-yl)(4-hydroxyphenyl)methyl)-1,4-dioxo-1,4-dihydronaphthalen-2-olate

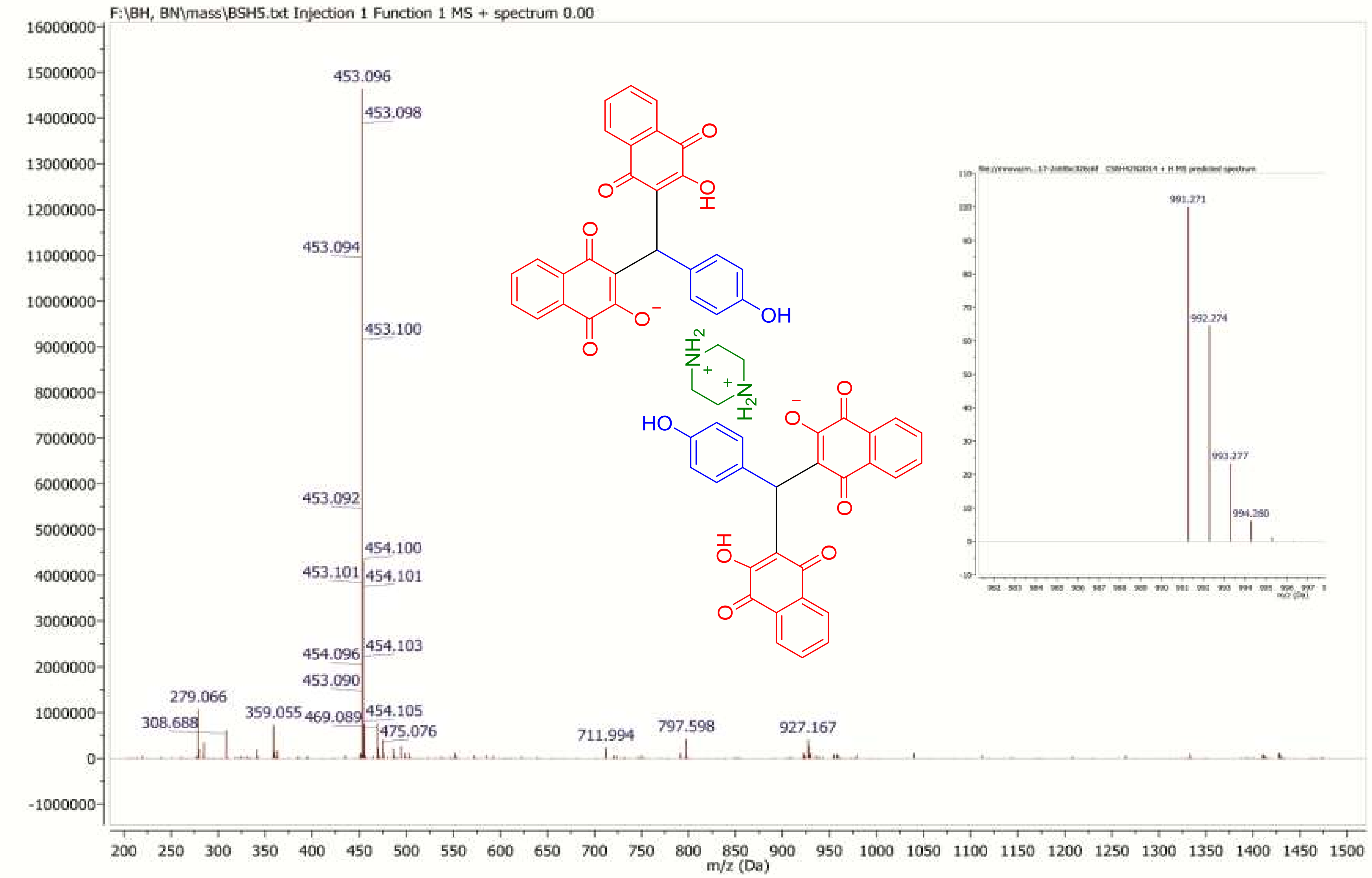

HR-Mass spectrum of piperazine-1,4-diium 3-((1,4-dioxo-1,4-dihydronaphthalen-2-yl)(4-hydroxyphenyl)methyl)-1,4-dioxo-1,4-dihydronaphthalen-2-olate

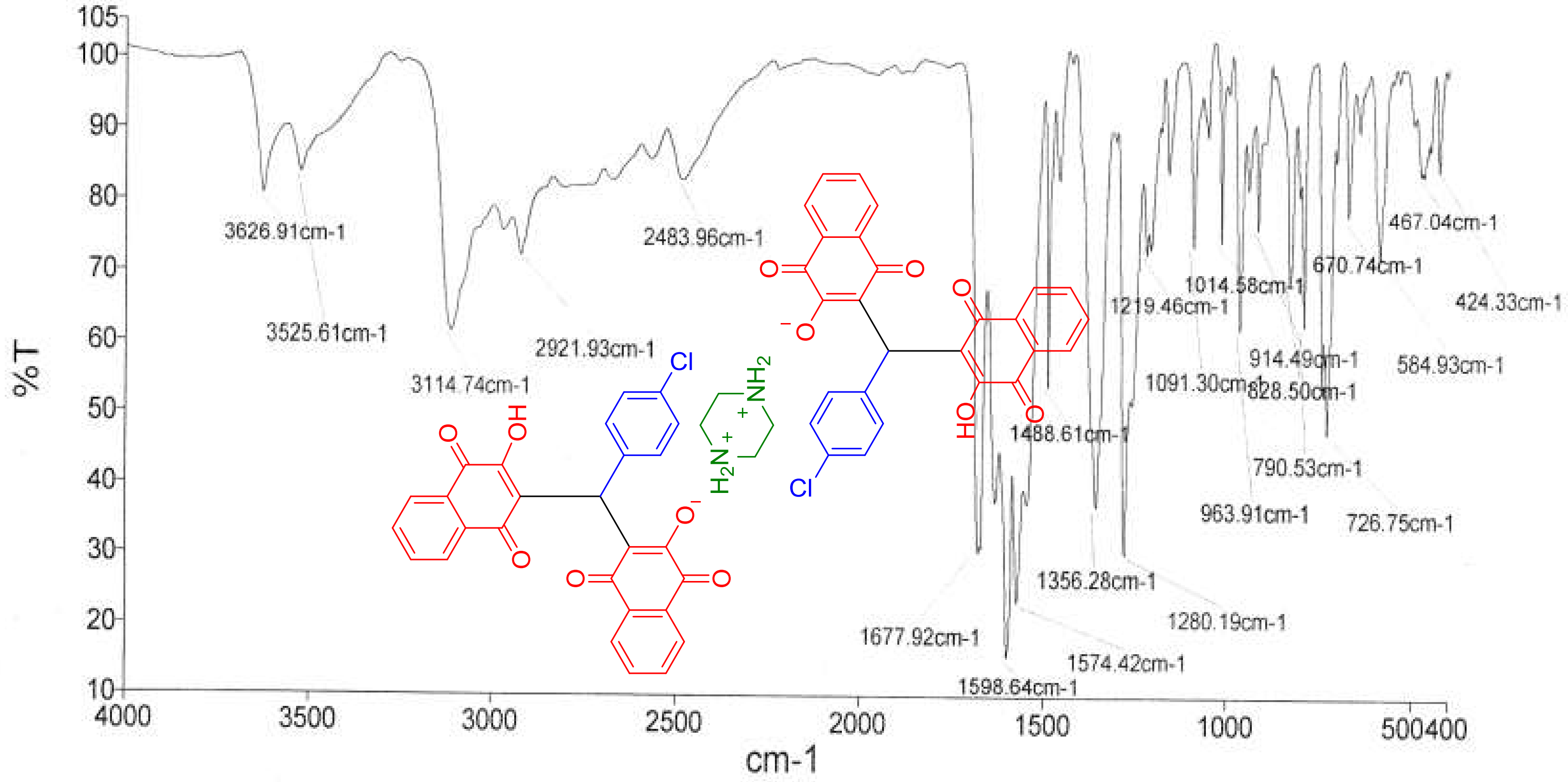

FT-IR spectrum of piperazine-1,4-diium 3-((4-chlorophenyl)(1,4-dioxo-1,4-dihydronaphthalen-2-yl)methyl)-1,4-dioxo-1,4-dihydronaphthalen-2-olate

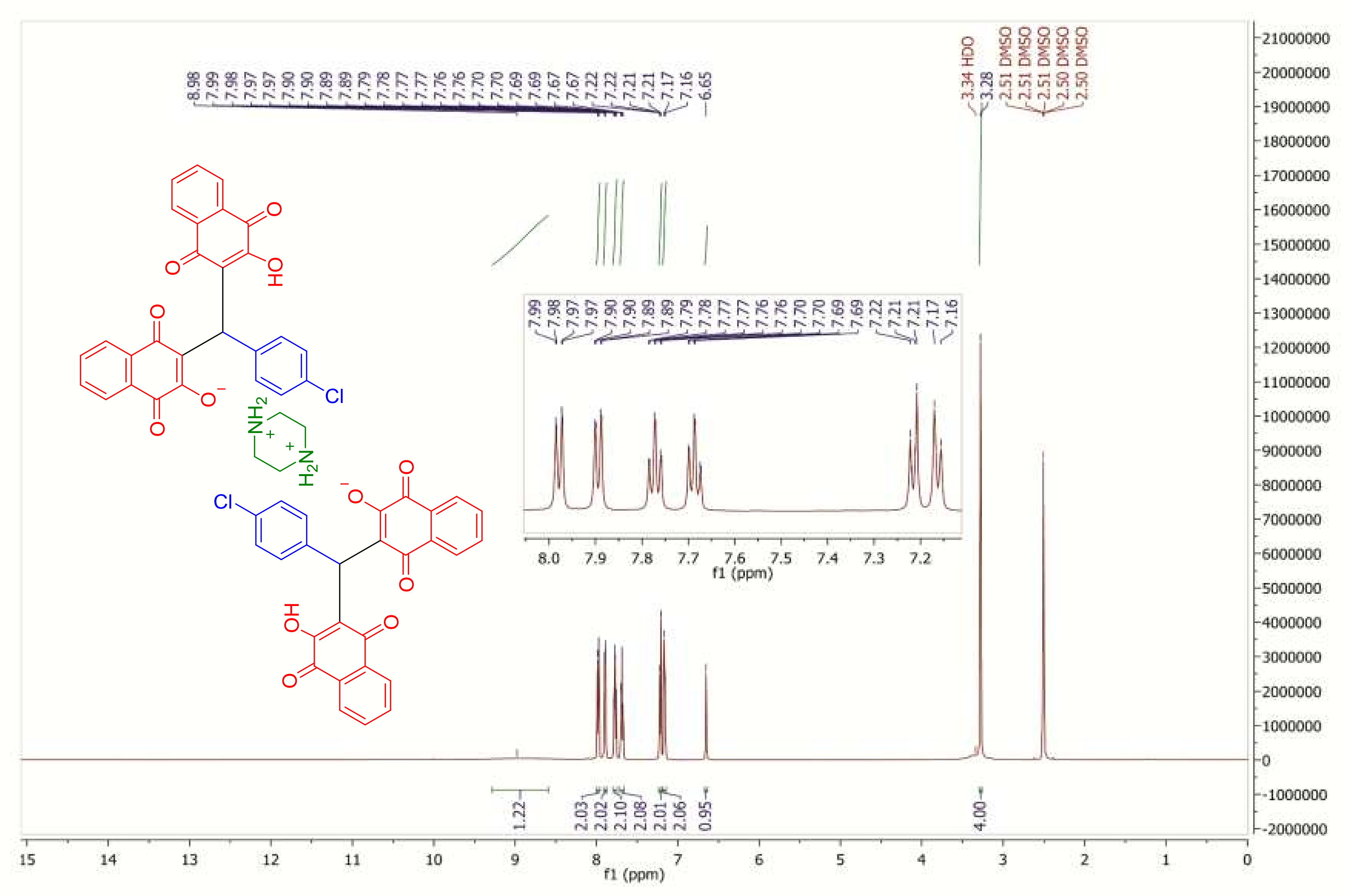

^{1}H NMR spectrum of piperazine-1,4-diium 3-((4-chlorophenyl)(1,4-dioxo-1,4-dihydronaphthalen-2-yl)methyl)-1,4-dioxo-1,4-dihydronaphthalen-2-olate

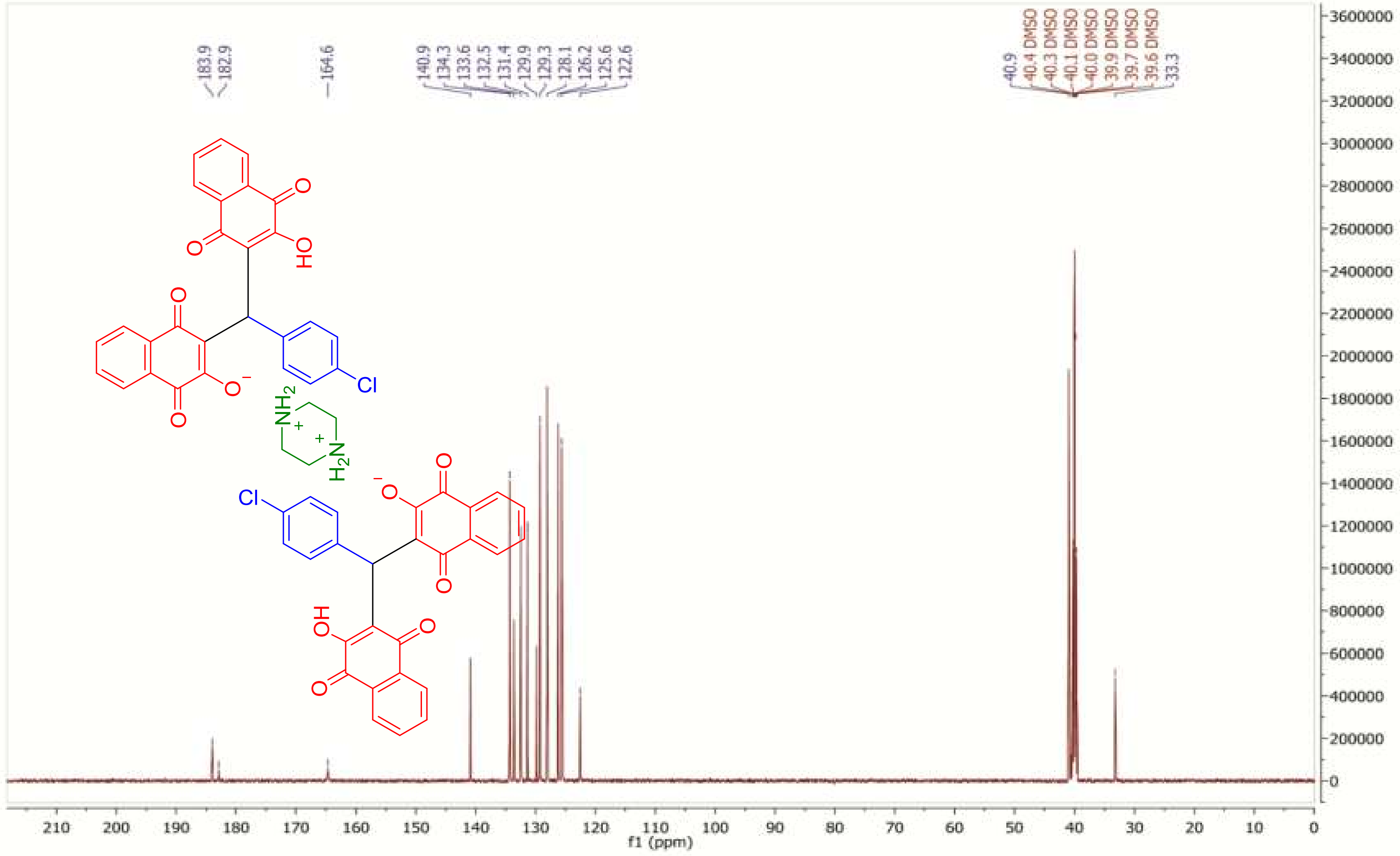

^{13}C NMR spectrum of piperazine-1,4-diium 3-((4-chlorophenyl)(1,4-dioxo-1,4-dihydronaphthalen-2-yl)methyl)-1,4-dioxo-1,4-dihydronaphthalen-2-olate

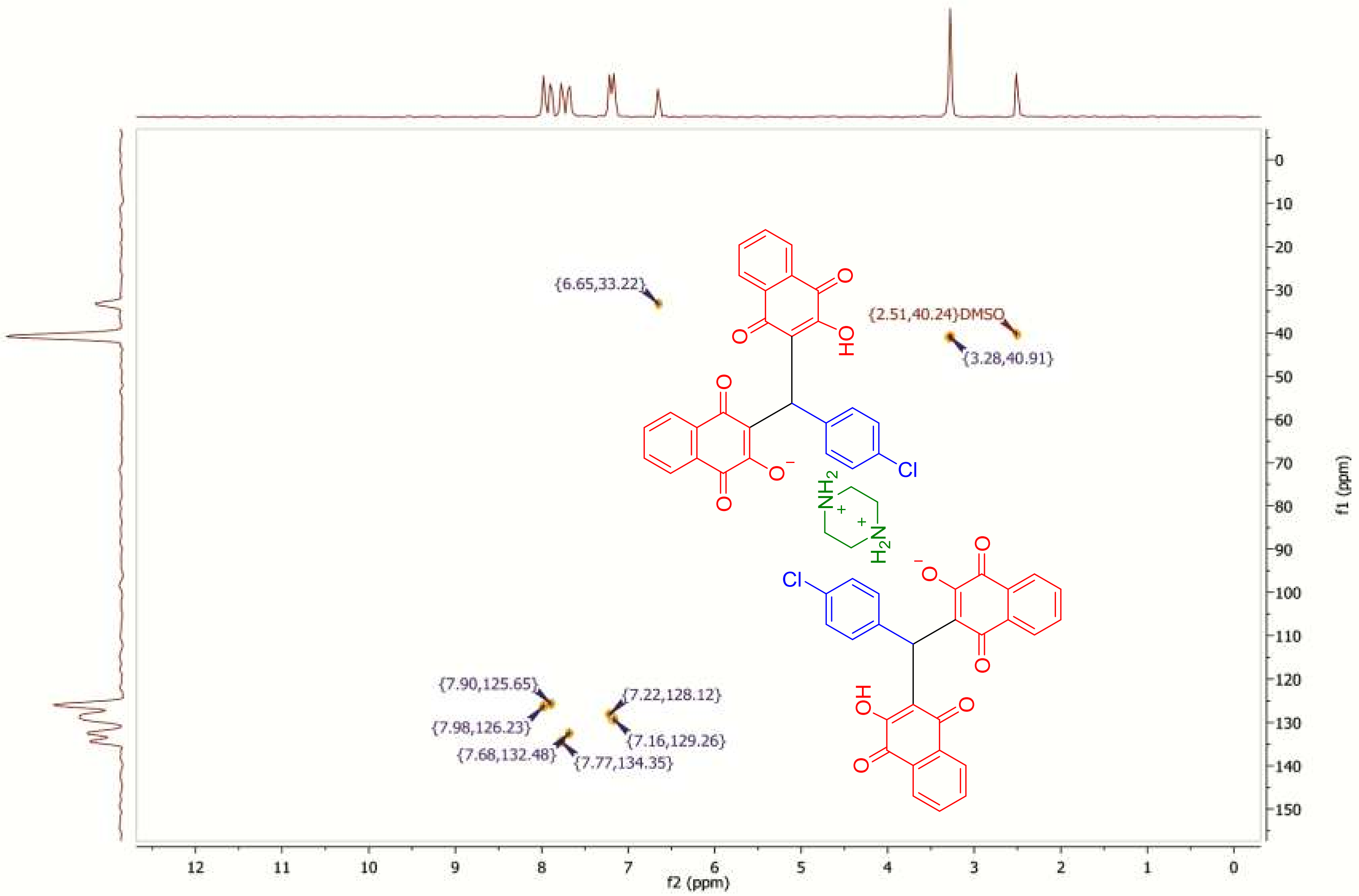

$^{1}H^{13}C$, HSQC-NMR spectrum of piperazine-1,4-diium 3-((4-chlorophenyl)(1,4-dioxo-1,4-dihydronaphthalen-2-yl)methyl)-1,4-dioxo-1,4-dihydronaphthalen-2-olate

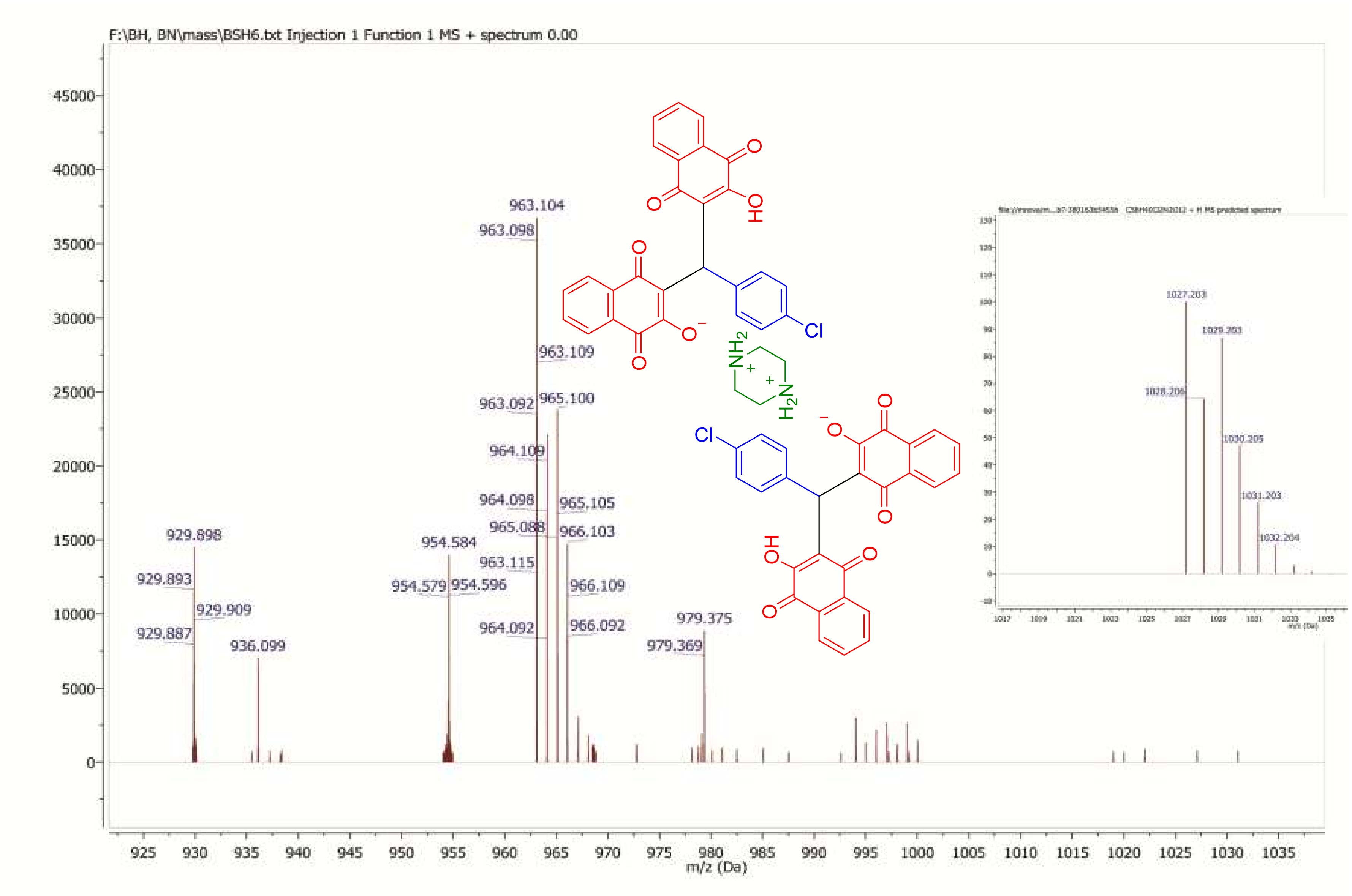

HR-Mass spectrum of piperazine-1,4-diium 3-((4-chlorophenyl)(1,4-dioxo-1,4-dihydronaphthalen-2-yl)methyl)-1,4-dioxo-1,4-dihydronaphthalen-2-olate

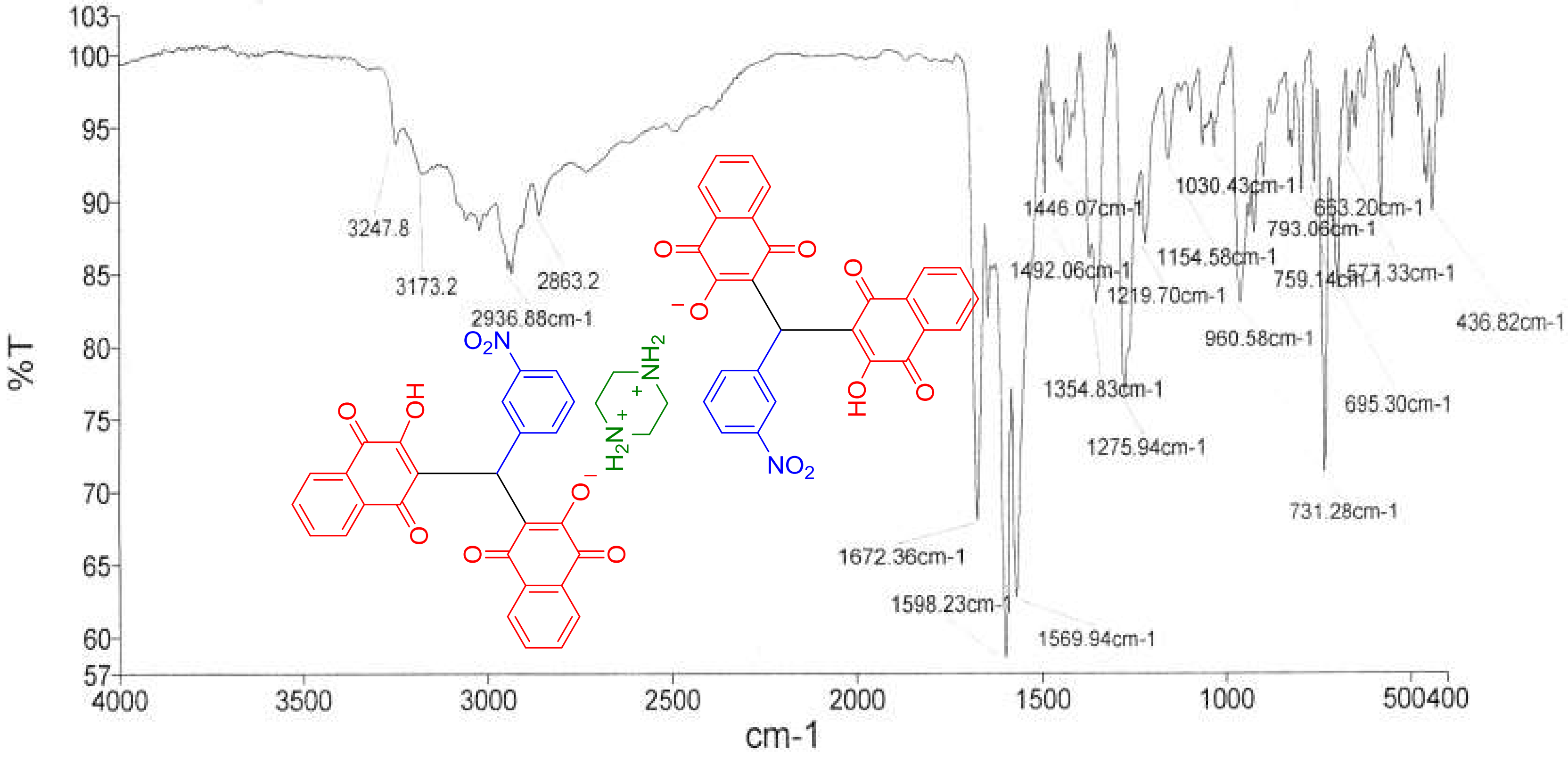

FT-IR spectrum of piperazine-1,4-diium 3-((1,4-dioxo-1,4-dihydronaphthalen-2-yl)(3-nitrophenyl)methyl)-1,4-dioxo-1,4-dihydronaphthalen-2-olate

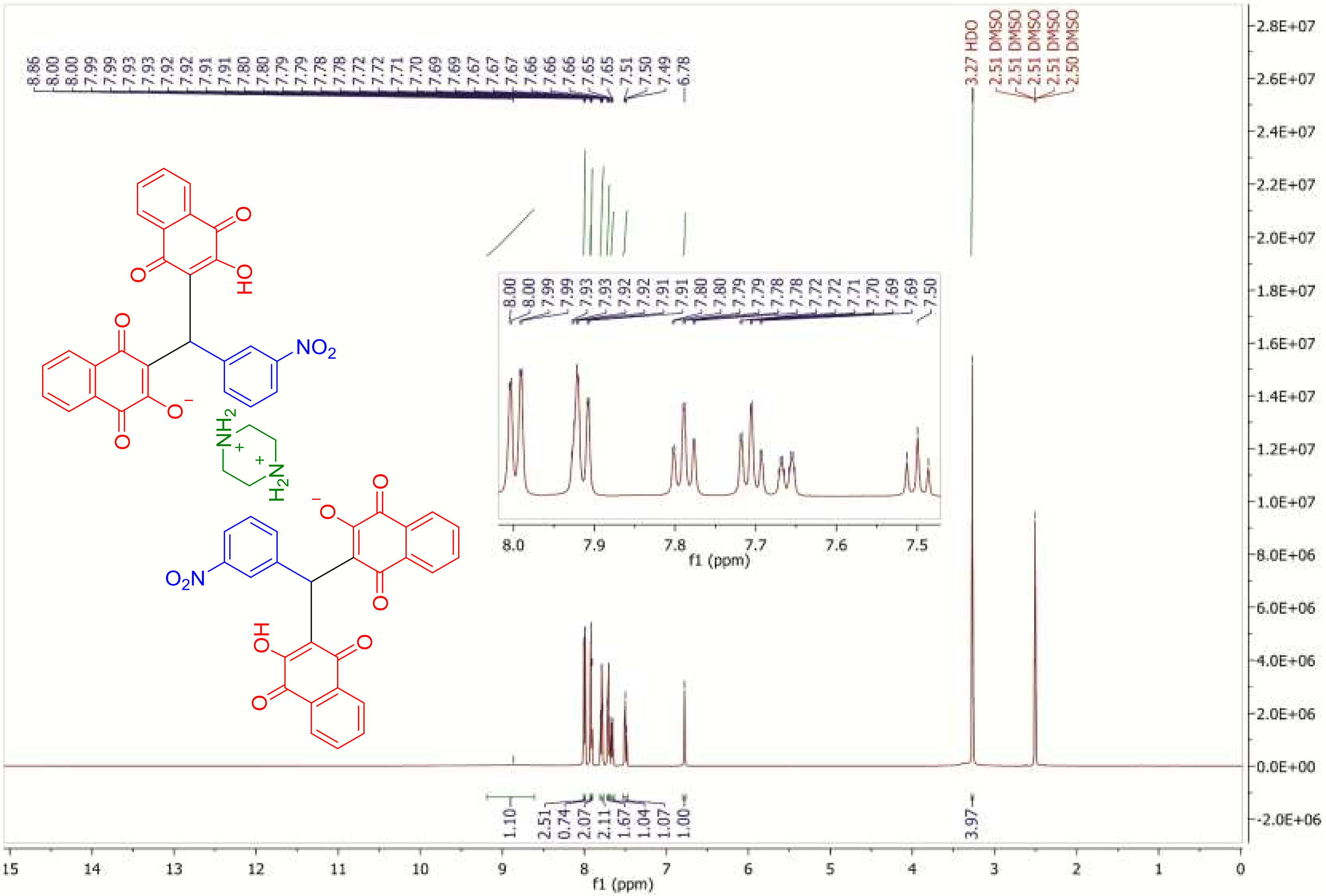

^{1}H NMR spectrum of piperazine-1,4-diium 3-((1,4-dioxo-1,4-dihydronaphthalen-2-yl)(3-nitrophenyl)methyl)-1,4-dioxo-1,4-dihydronaphthalen-2-olate

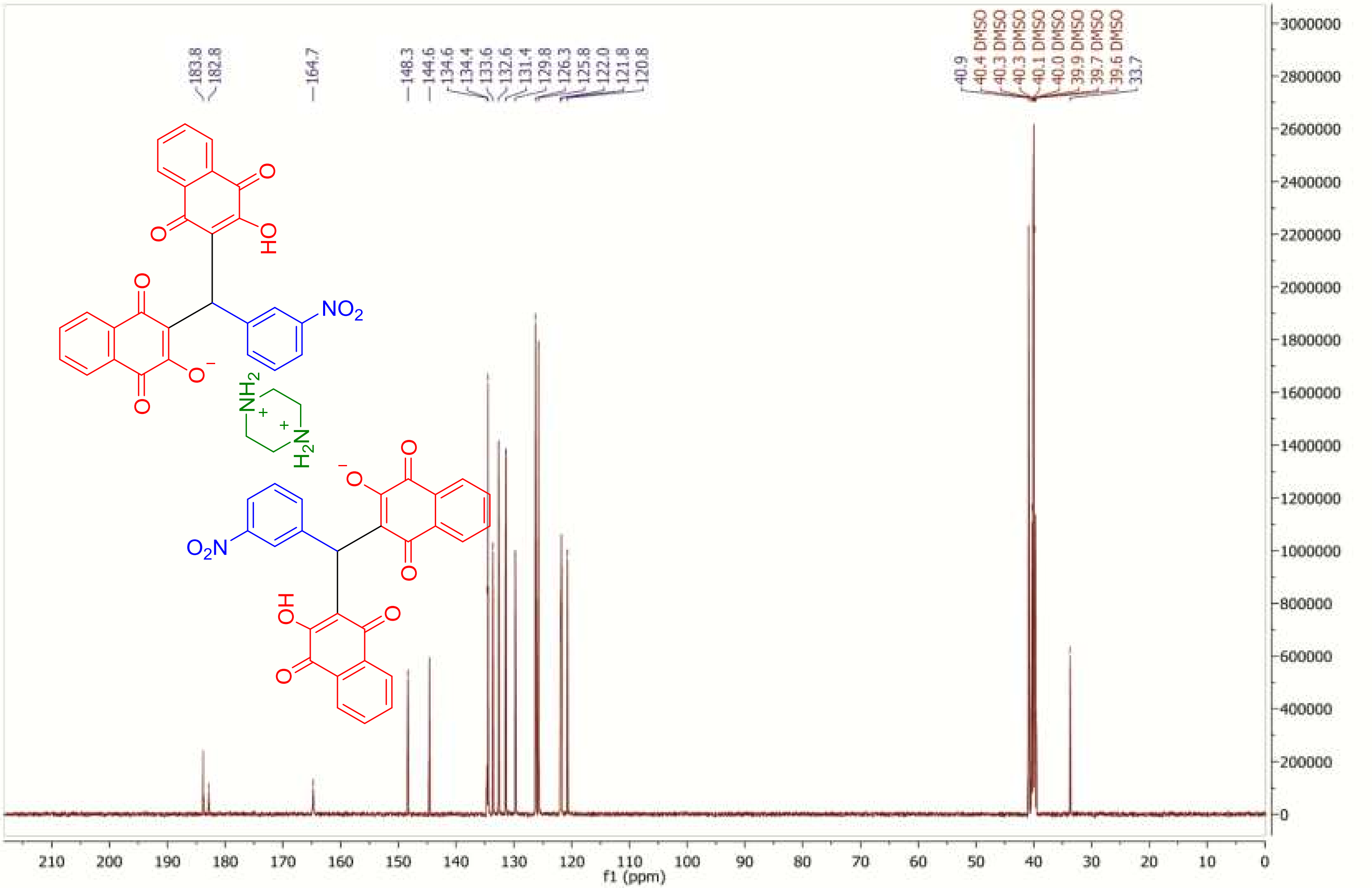

^{13}C NMR spectrum of piperazine-1,4-diium 3-((1,4-dioxo-1,4-dihydronaphthalen-2-yl)(3-nitrophenyl)methyl)-1,4-dioxo-1,4-dihydronaphthalen-2-olate

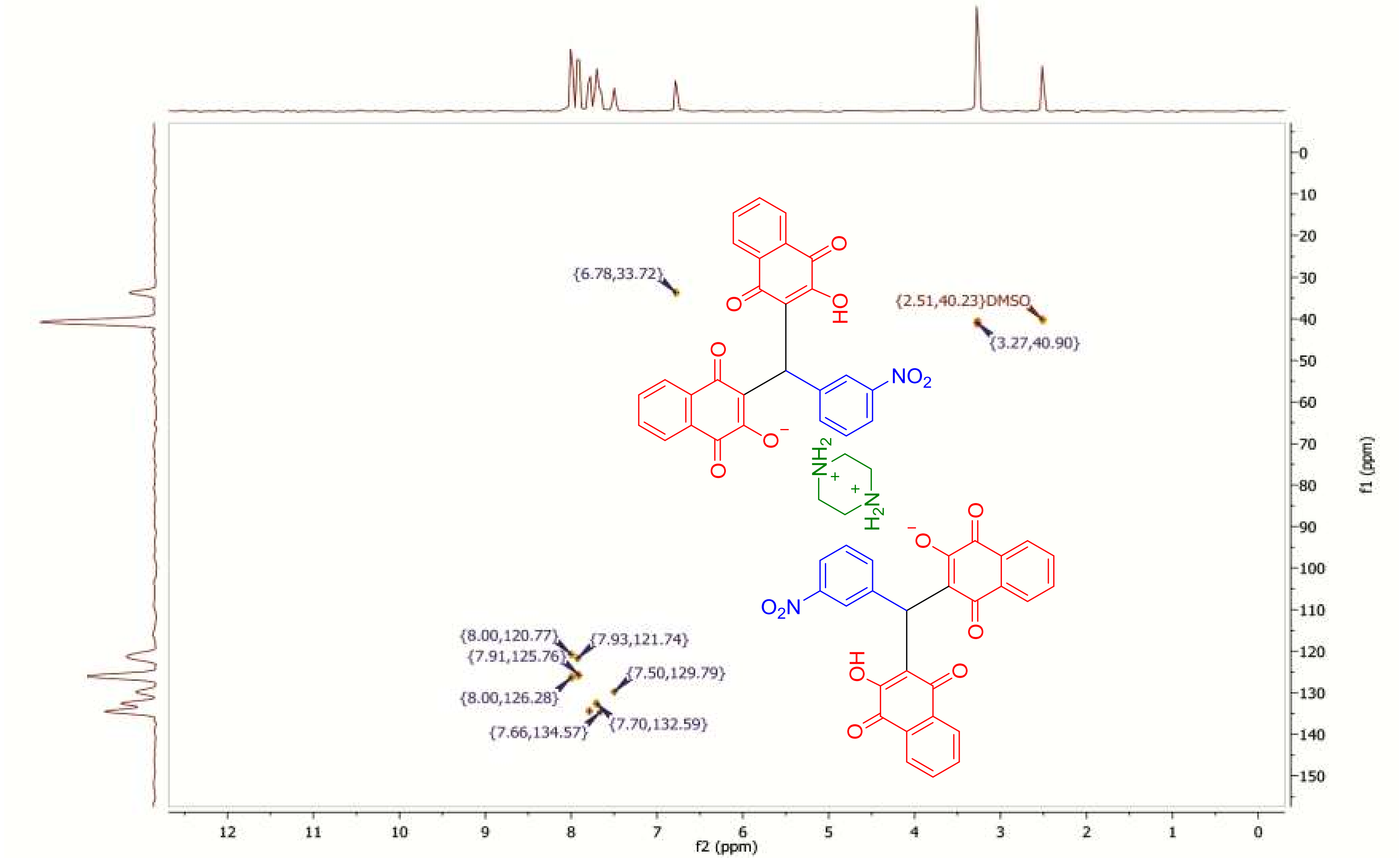

$^{1}H^{13}C$, HSQC-NMR spectrum of piperazine-1,4-diium 3-((1,4-dioxo-1,4-dihydronaphthalen-2-yl)(3-nitrophenyl)methyl)-1,4-dioxo-1,4-dihydronaphthalen-2-olate

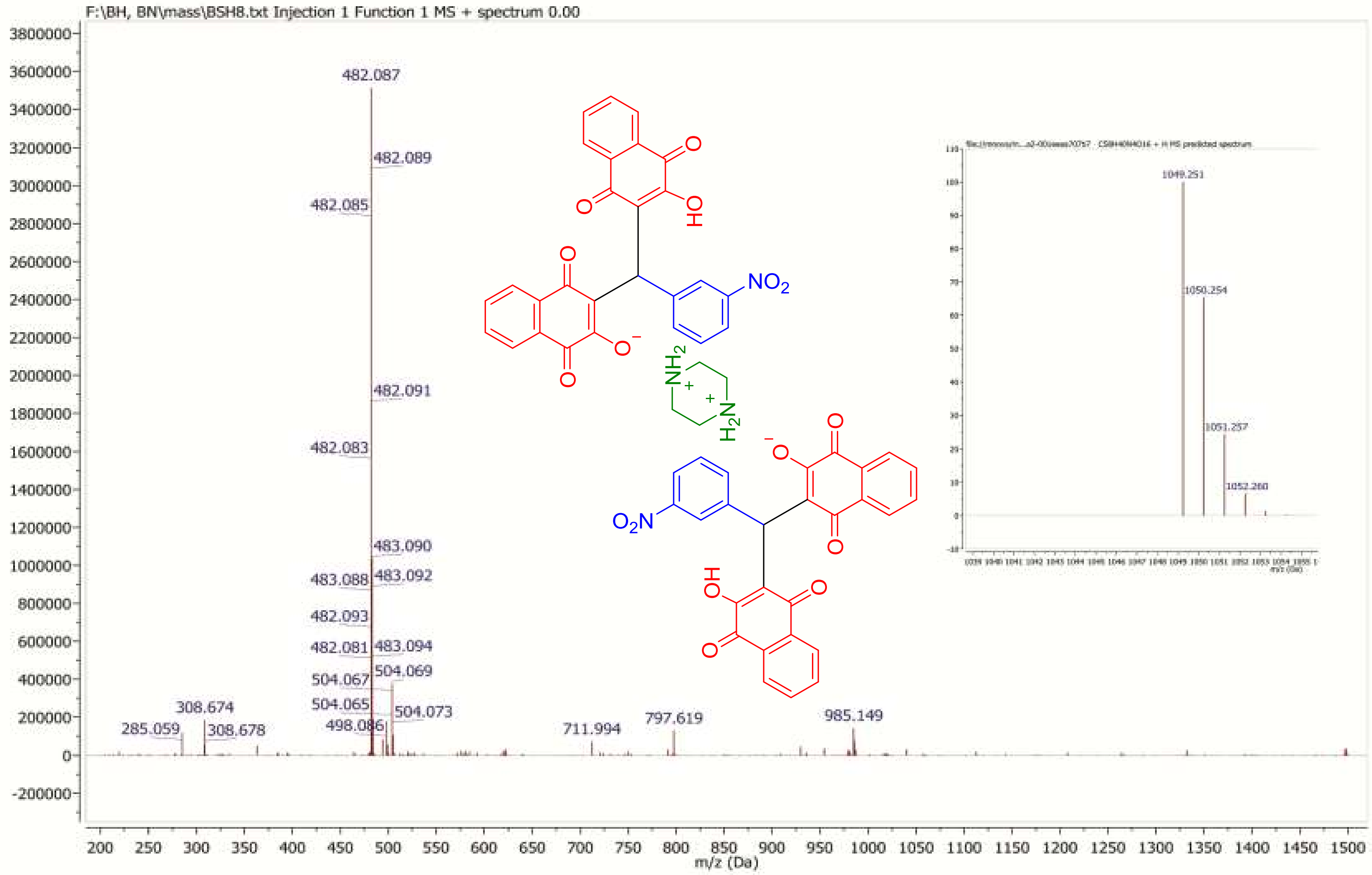

HR-Mass spectrum of piperazine-1,4-diium 3-((1,4-dioxo-1,4-dihydronaphthalen-2-yl)(3-nitrophenyl)methyl)-1,4-dioxo-1,4-dihydronaphthalen-2-olate

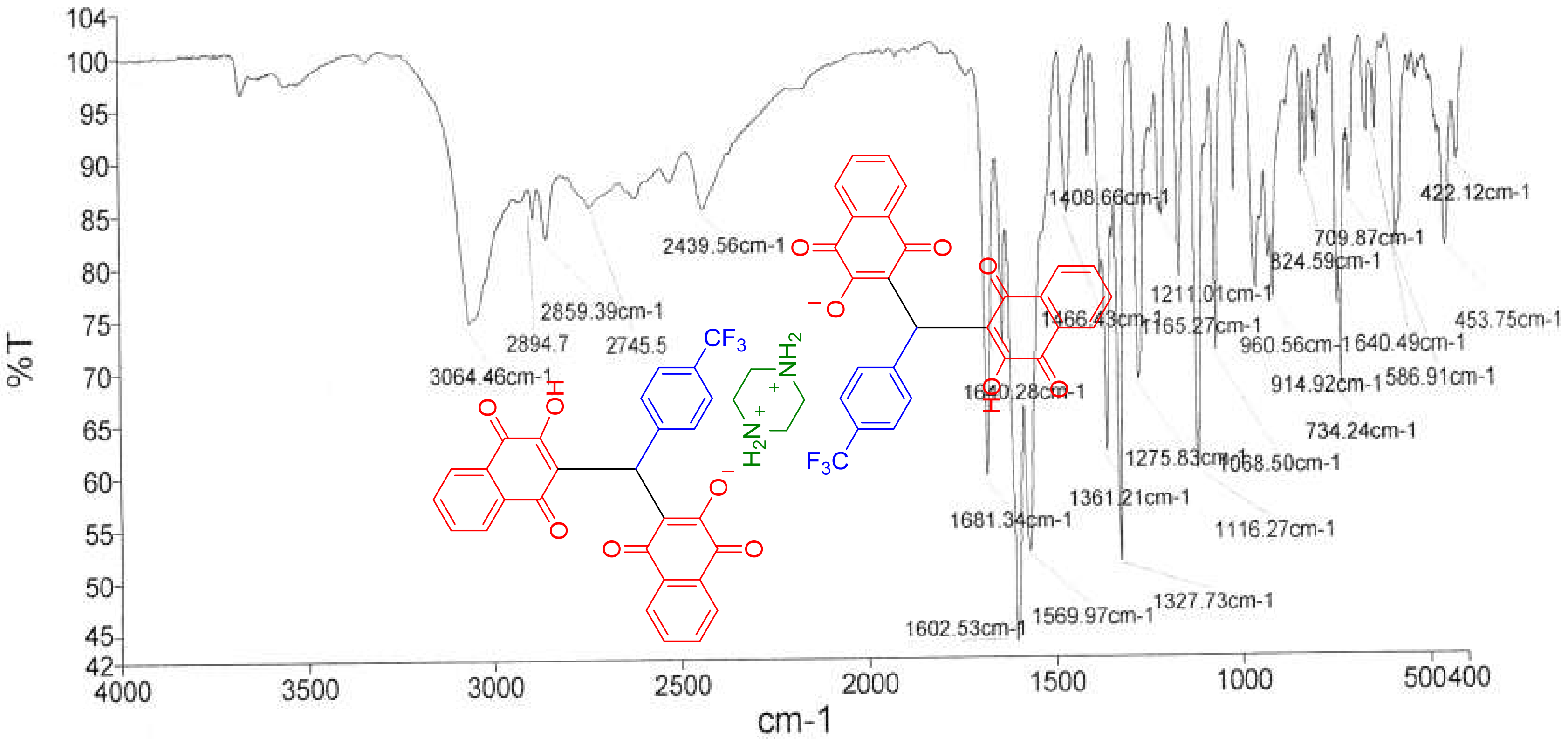

FT-IR spectrum of piperazine-1,4-diium 3-((1,4-dioxo-1,4-dihydronaphthalen-2-yl)(4-(trifluoromethyl)phenyl)methyl)-1,4-dioxo-1,4-dihydronaphthalen-2-olate

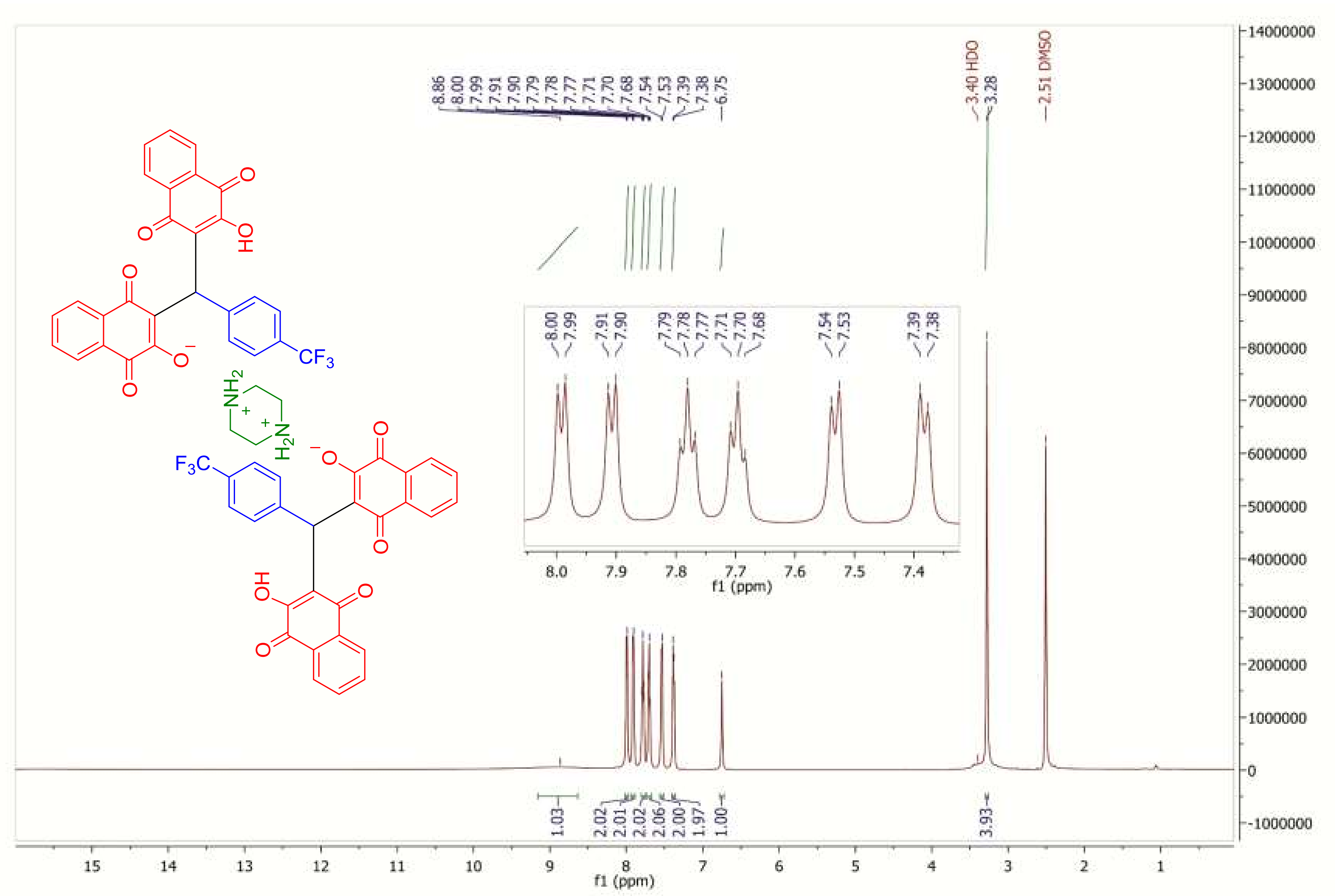

^{1}H NMR spectrum of piperazine-1,4-diium 3-((1,4-dioxo-1,4-dihydronaphthalen-2-yl)(4-(trifluoromethyl)phenyl)methyl)-1,4-dioxo-1,4-dihydronaphthalen-2-olate

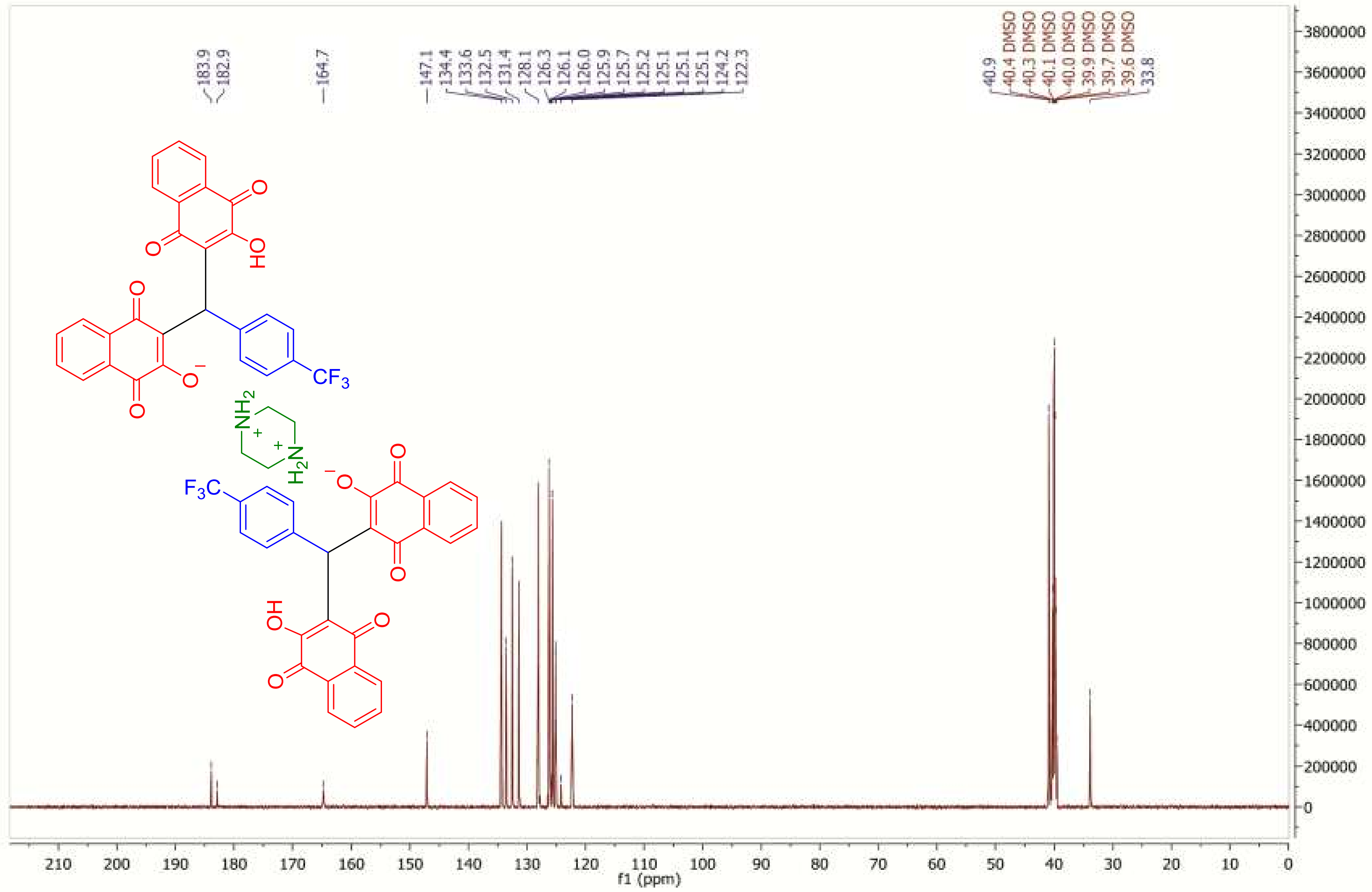

^{13}C NMR spectrum of piperazine-1,4-diium 3-((1,4-dioxo-1,4-dihydronaphthalen-2-yl)(4-(trifluoromethyl)phenyl)methyl)-1,4-dioxo-1,4-dihydronaphthalen-2-olate

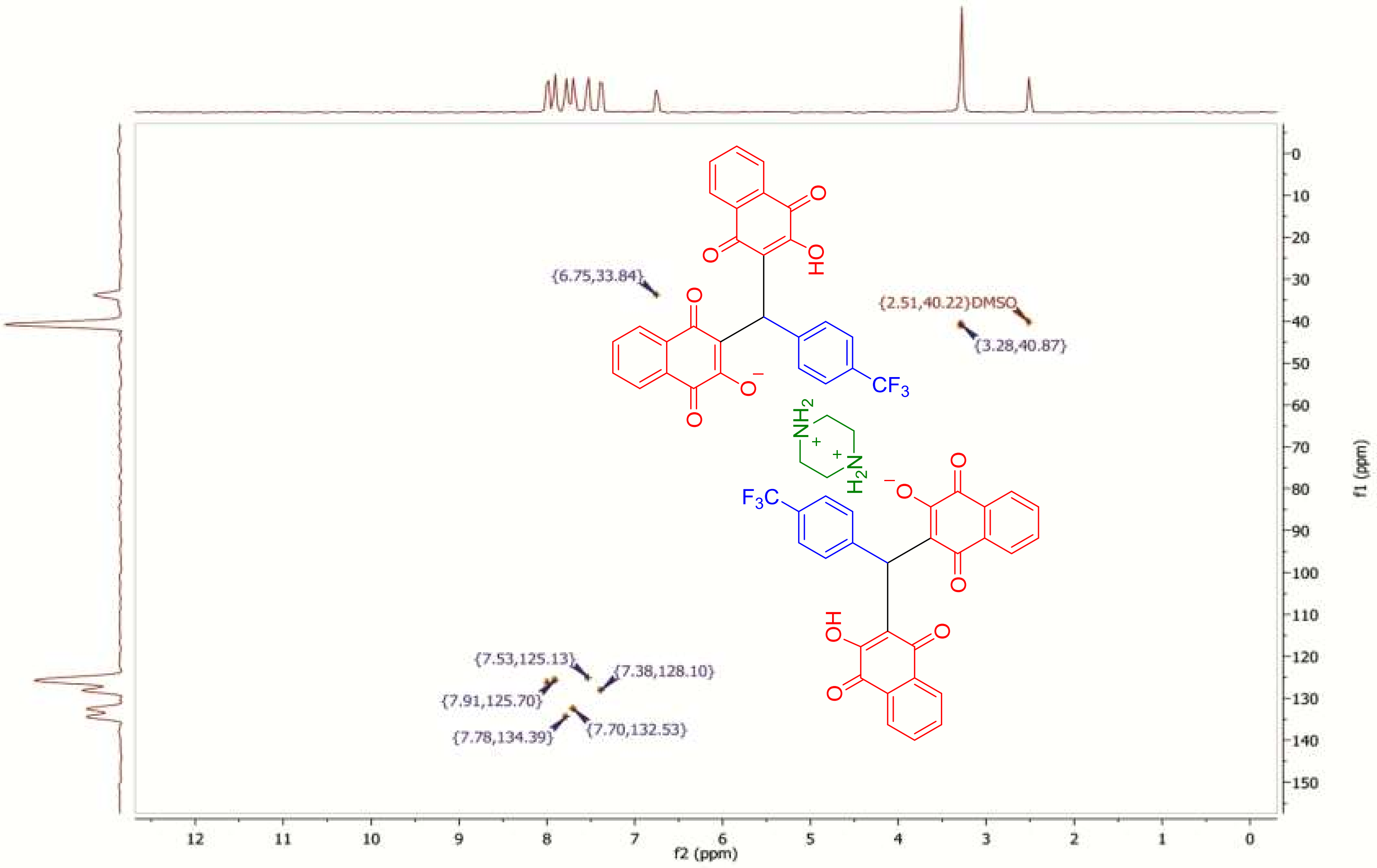

$^{1}H^{13}C$, HSQC-NMR spectrum of piperazine-1,4-diium 3-((1,4-dioxo-1,4-dihydronaphthalen-2-yl)(4-(trifluoromethyl)phenyl)methyl)-1,4-dioxo-1,4-dihydronaphthalen-2-olate

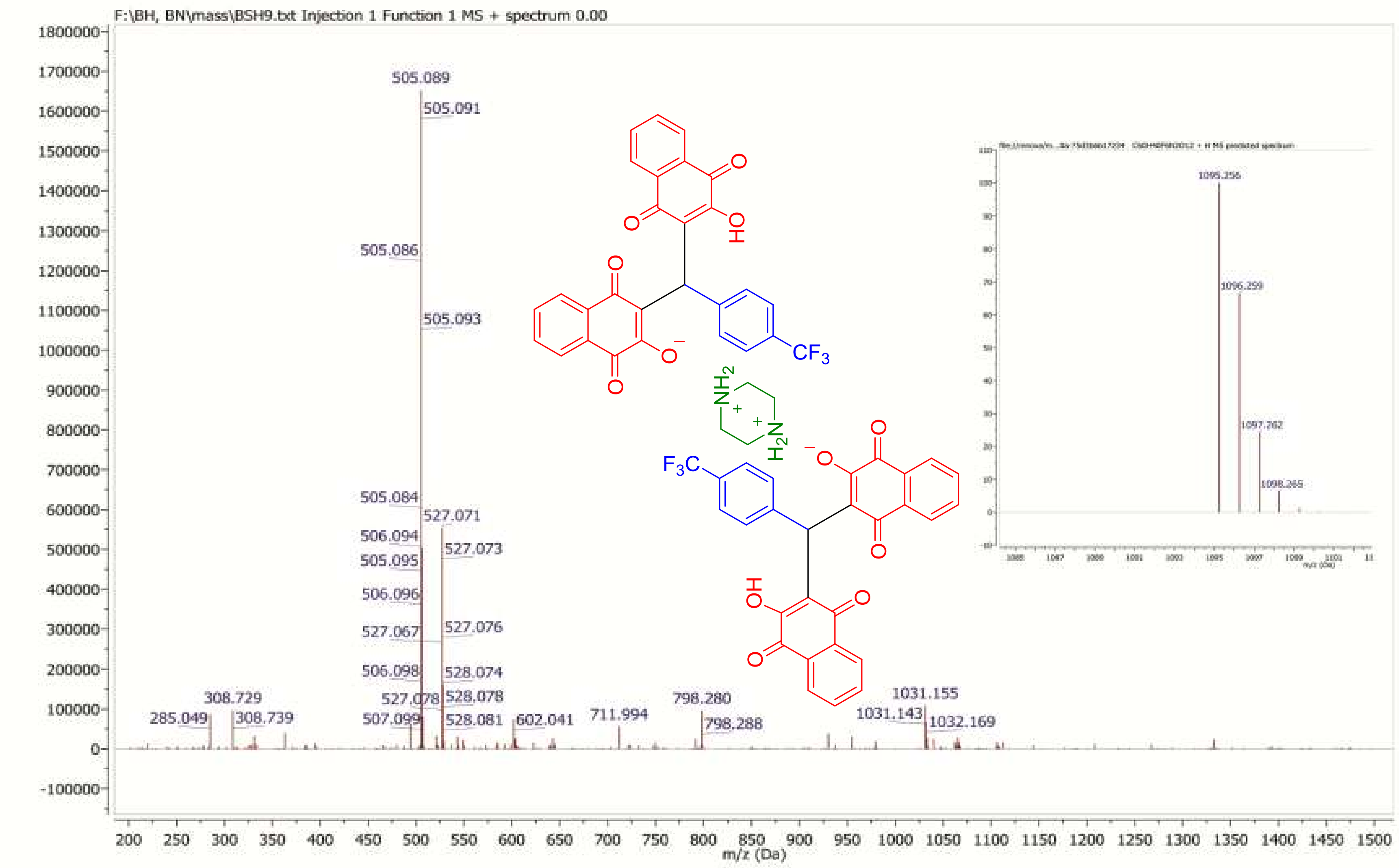

HR-Mass spectrum of piperazine-1,4-diium 3-((1,4-dioxo-1,4-dihydronaphthalen-2-yl)(4-(trifluoromethyl)phenyl)methyl)-1,4-dioxo-1,4-dihydronaphthalen-2-olate

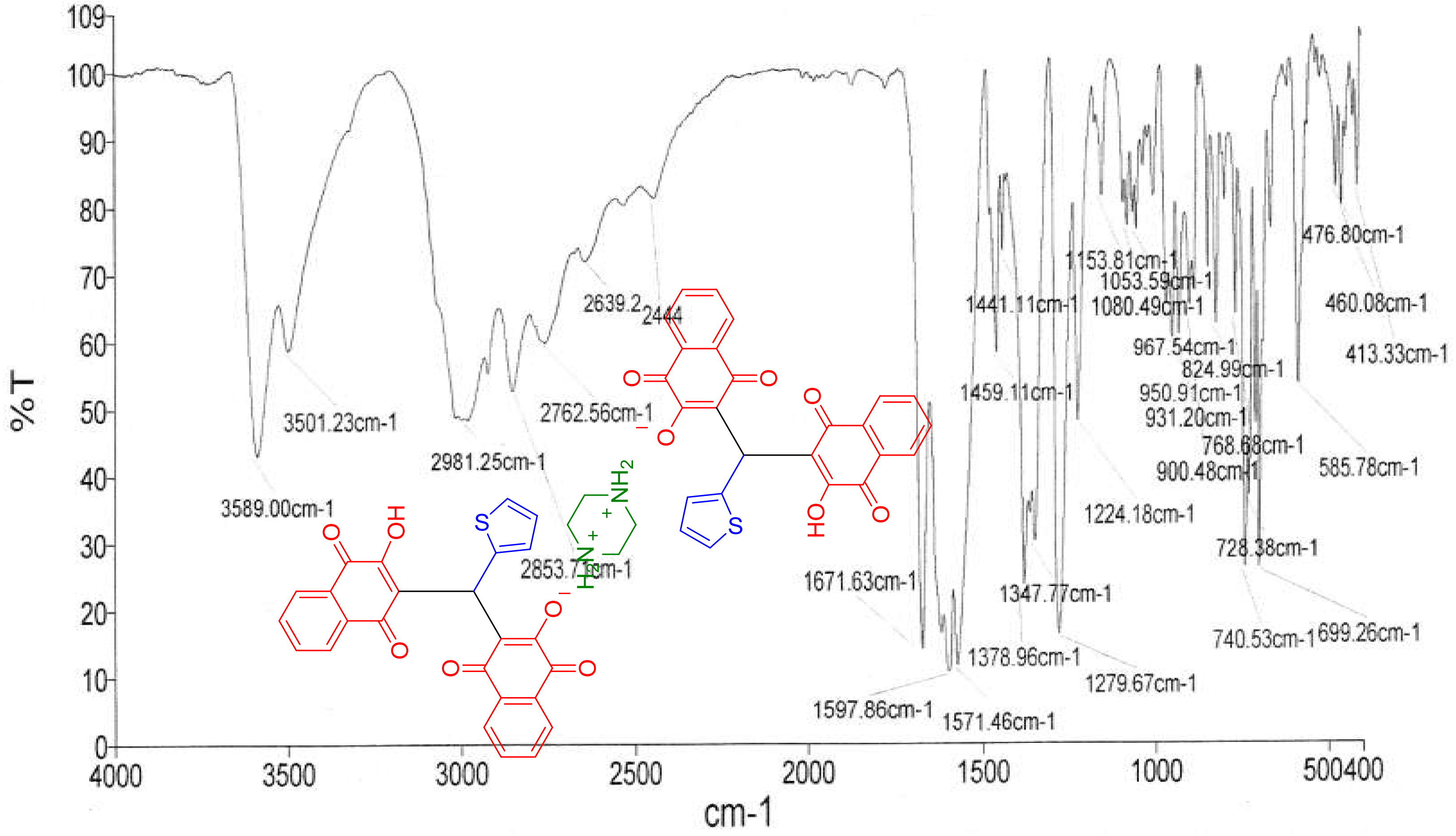

FT-IR spectrum ofpiperazine-1,4-diium 3-((1,4-dioxo-1,4-dihydronaphthalen-2-yl)(thiophen-2-yl)methyl)-1,4-dioxo-1,4-dihydronaphthalen-2-olate

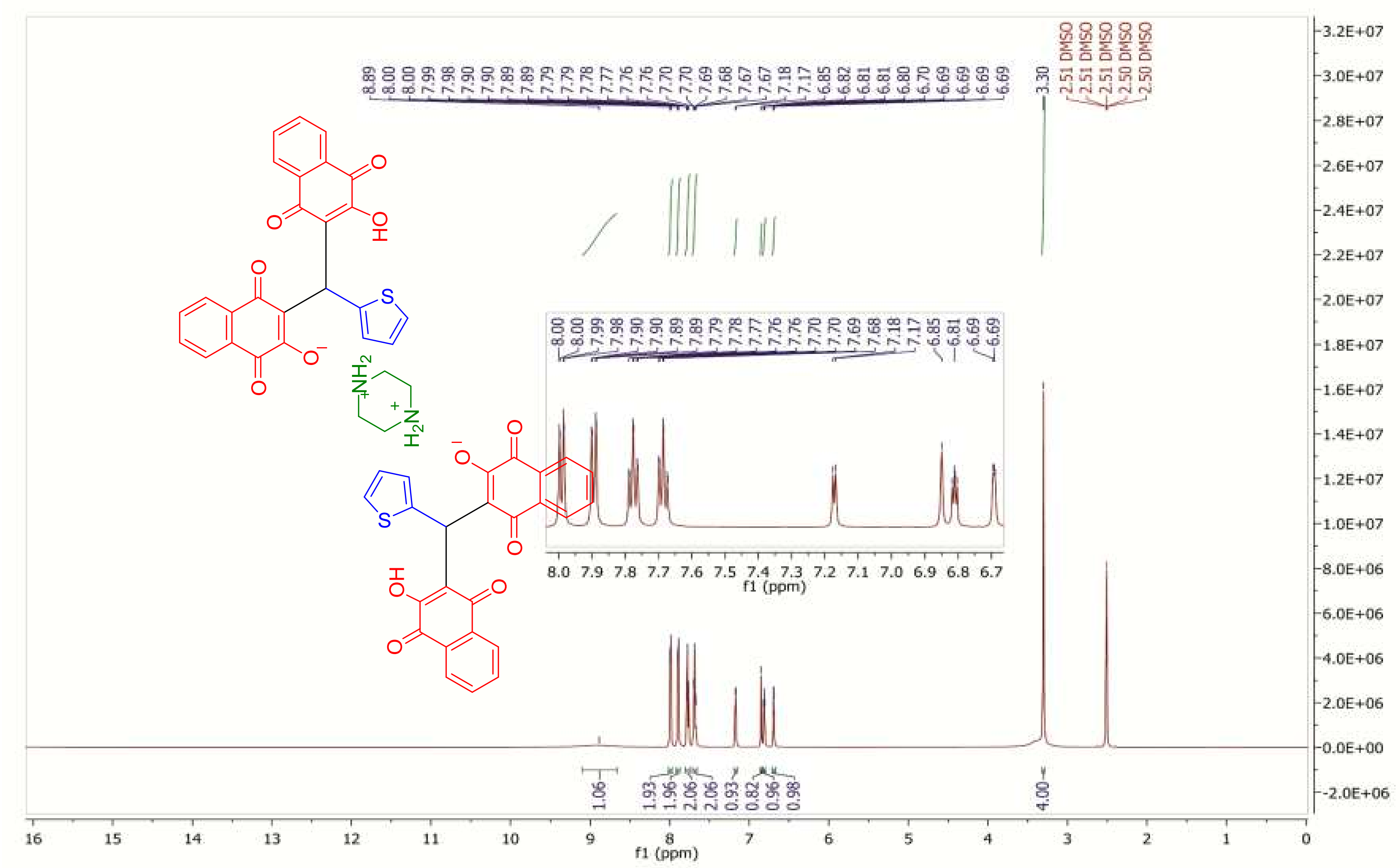

^{1}H NMR spectrum of piperazine-1,4-diium 3-((1,4-dioxo-1,4-dihydronaphthalen-2-yl)(thiophen-2-yl)methyl)-1,4-dioxo-1,4-dihydronaphthalen-2-olate

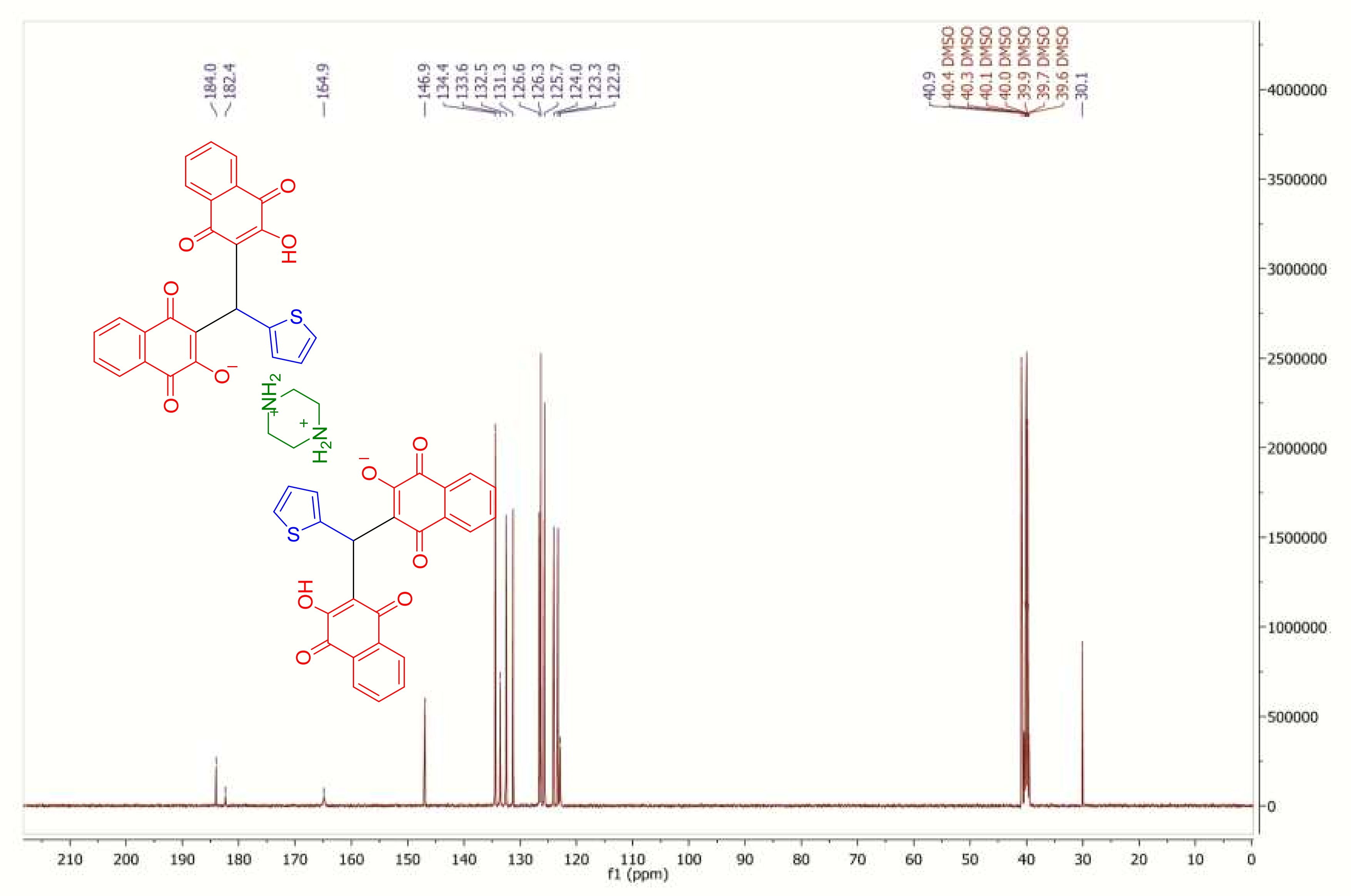

^{13}C NMR spectrum of piperazine-1,4-diium 3-((1,4-dioxo-1,4-dihydronaphthalen-2-yl)(thiophen-2-yl)methyl)-1,4-dioxo-1,4-dihydronaphthalen-2-olate

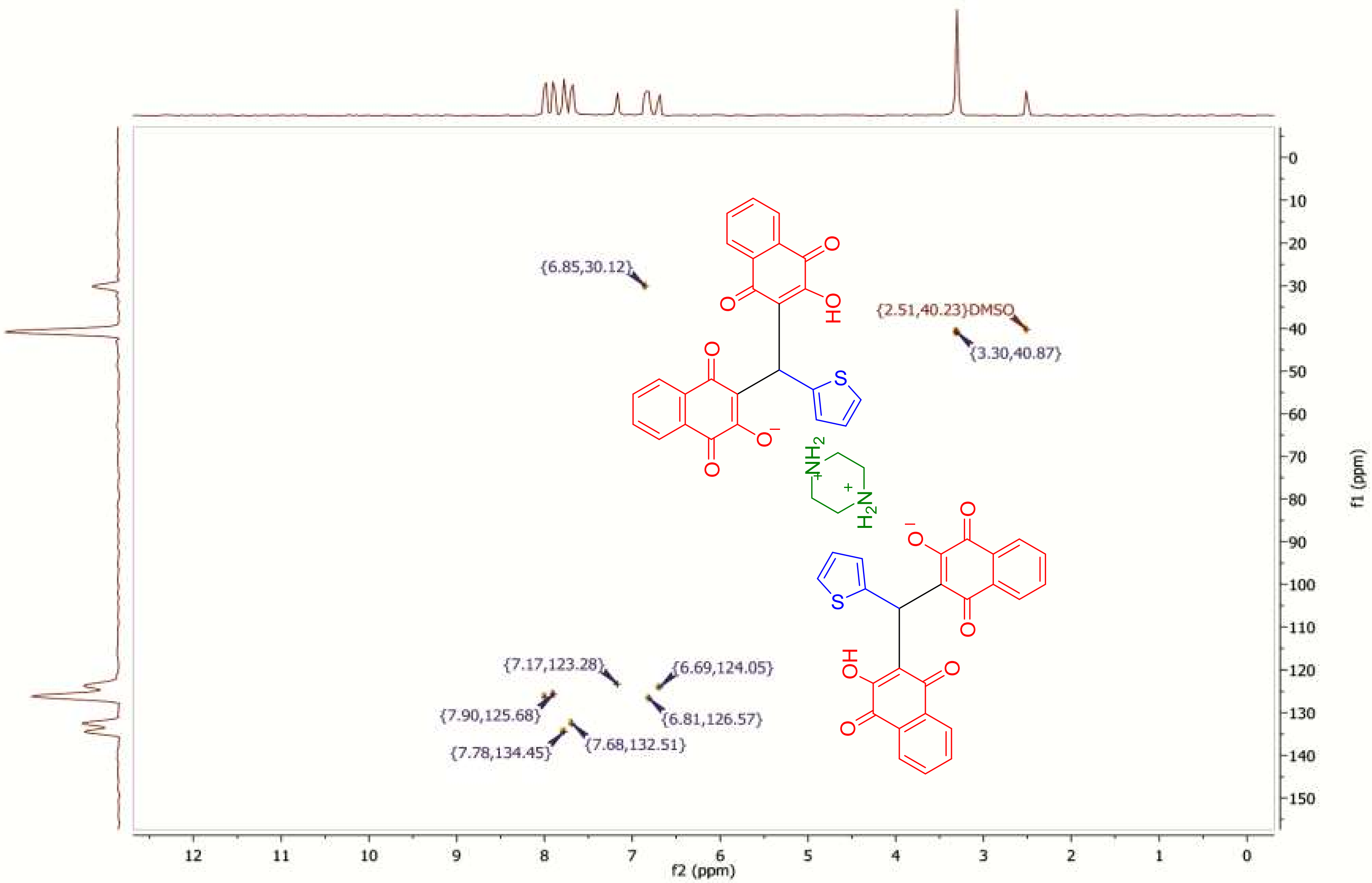

$^{1}H^{13}C$, HSQC-NMR spectrum of piperazine-1,4-diium 3-((1,4-dioxo-1,4-dihydronaphthalen-2-yl)(thiophen-2-yl)methyl)-1,4-dioxo-1,4-dihydronaphthalen-2-olate

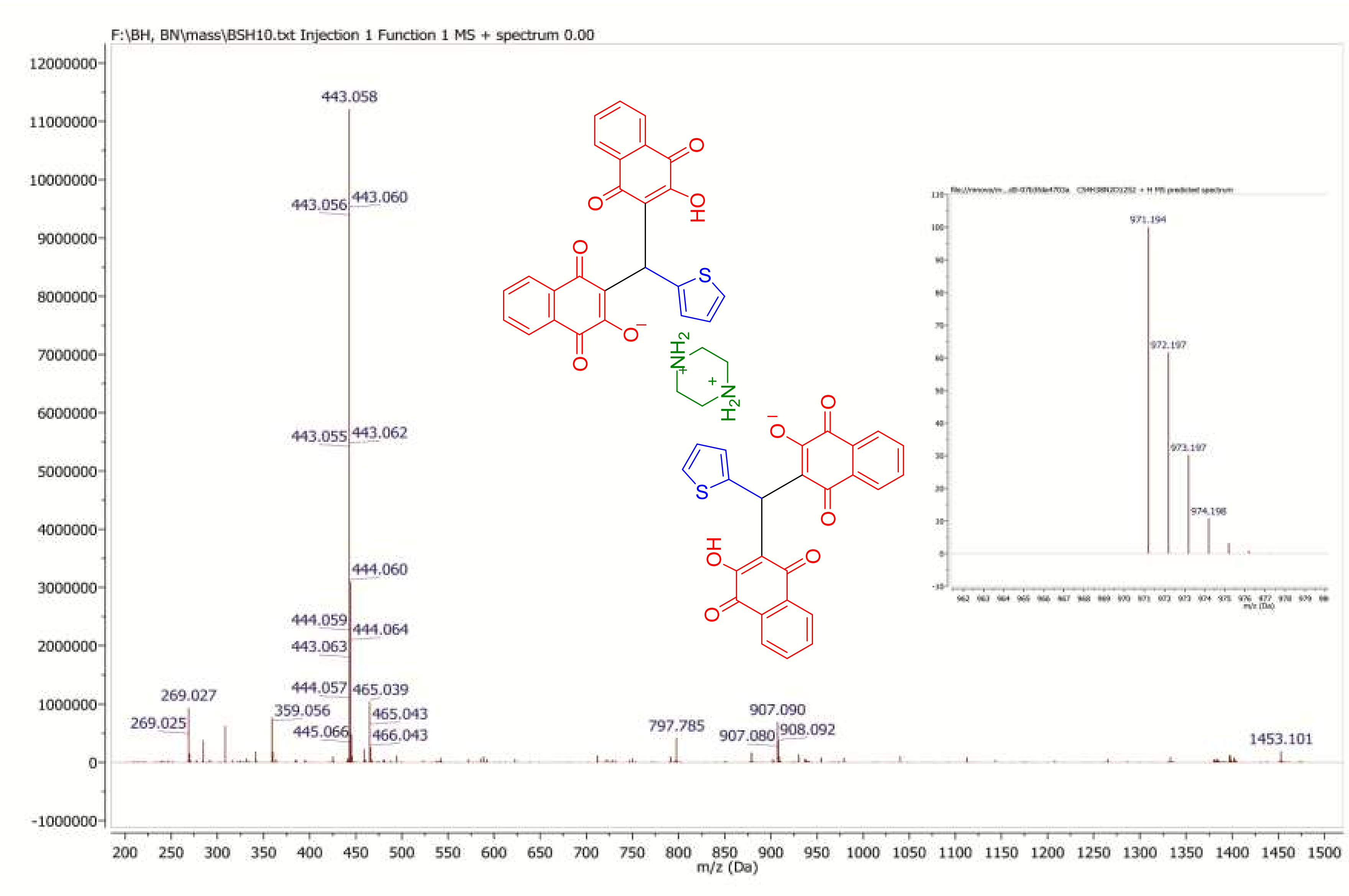

HR-Mass spectrum of piperazine-1,4-diium 3-((1,4-dioxo-1,4-dihydronaphthalen-2-yl)(thiophen-2-yl)methyl)-1,4-dioxo-1,4-dihydronaphthalen-2-olate

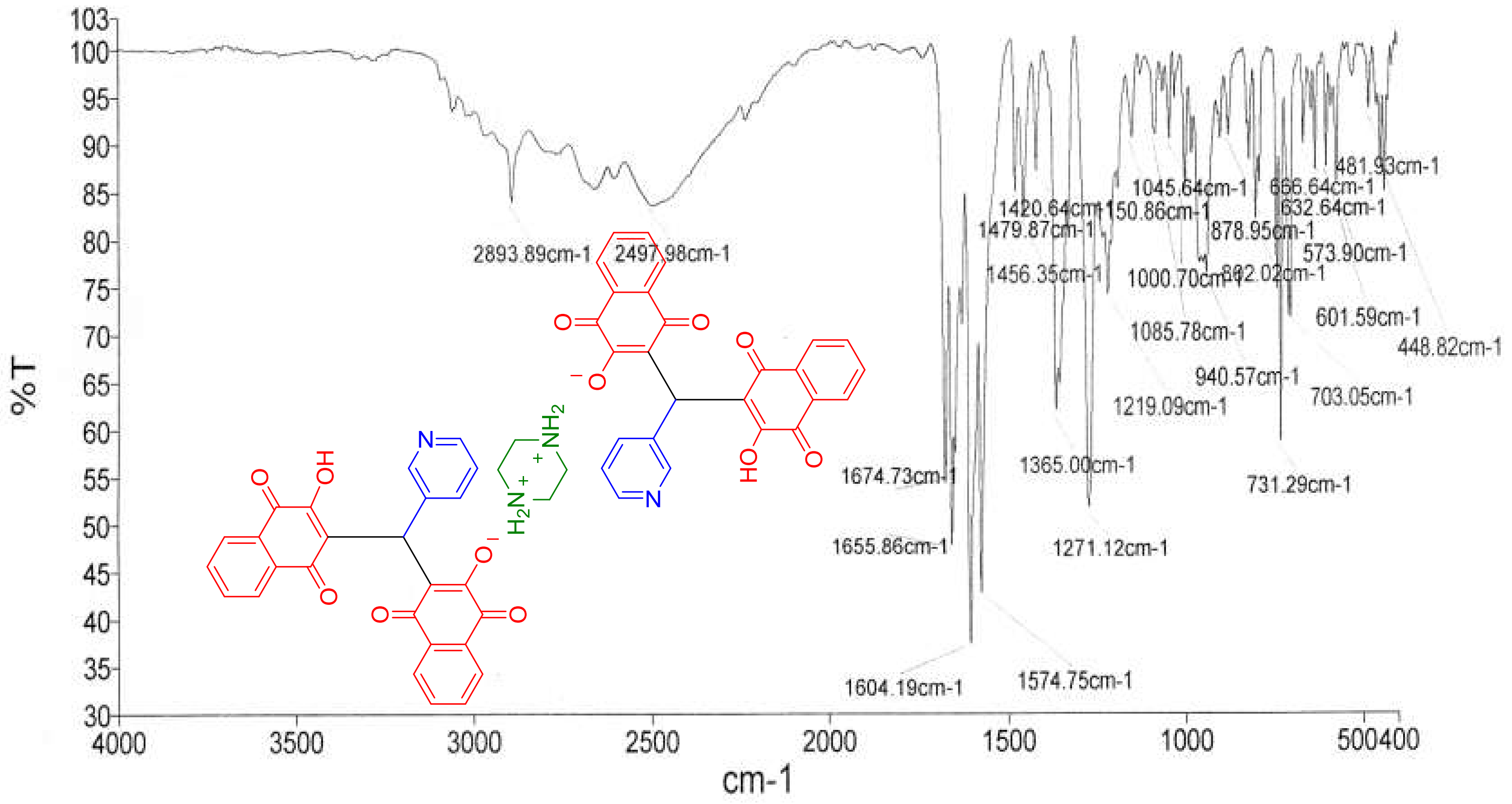

FT-IR spectrum of piperazine-1,4-diium 3-((1,4-dioxo-1,4-dihydronaphthalen-2-yl)(pyridin-3-yl)methyl)-1,4-dioxo-1,4-dihydronaphthalen-2-olate

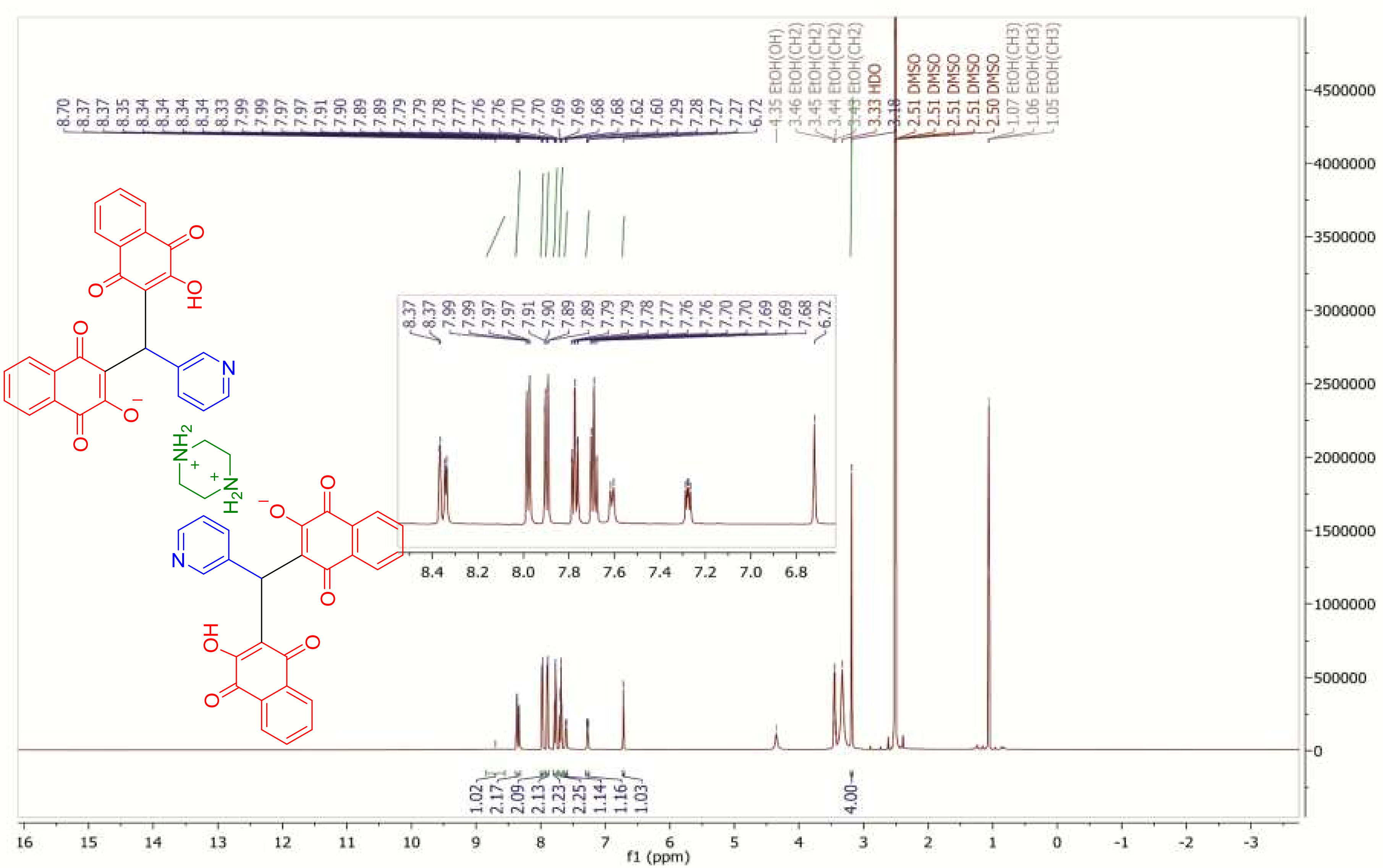

^{1}H NMR spectrum of piperazine-1,4-diium 3-((1,4-dioxo-1,4-dihydronaphthalen-2-yl)(pyridin-3-yl)methyl)-1,4-dioxo-1,4-dihydronaphthalen-2-olate

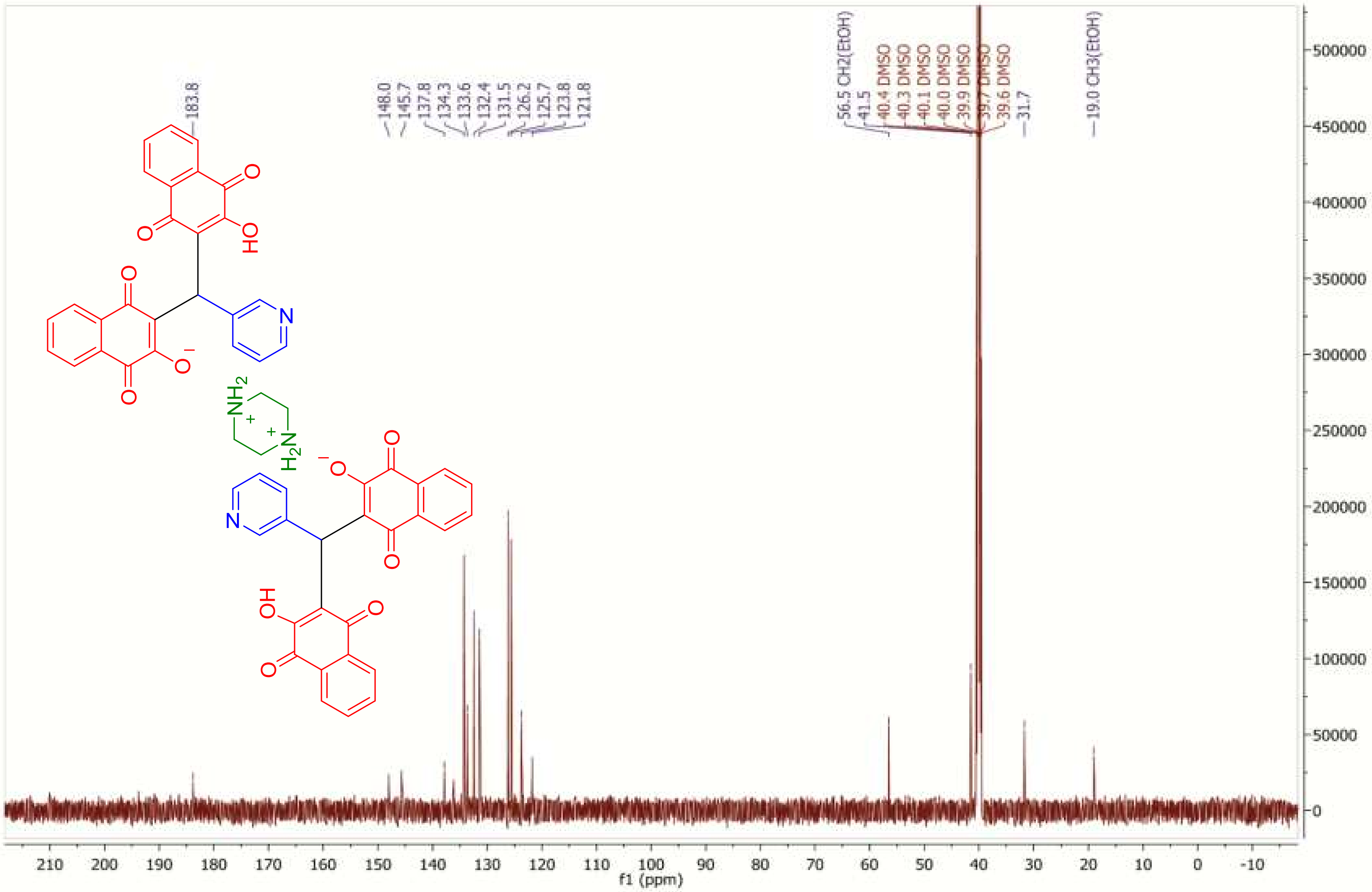

^{13}C NMR spectrum of piperazine-1,4-diium 3-((1,4-dioxo-1,4-dihydronaphthalen-2-yl)(pyridin-3-yl)methyl)-1,4-dioxo-1,4-dihydronaphthalen-2-olate

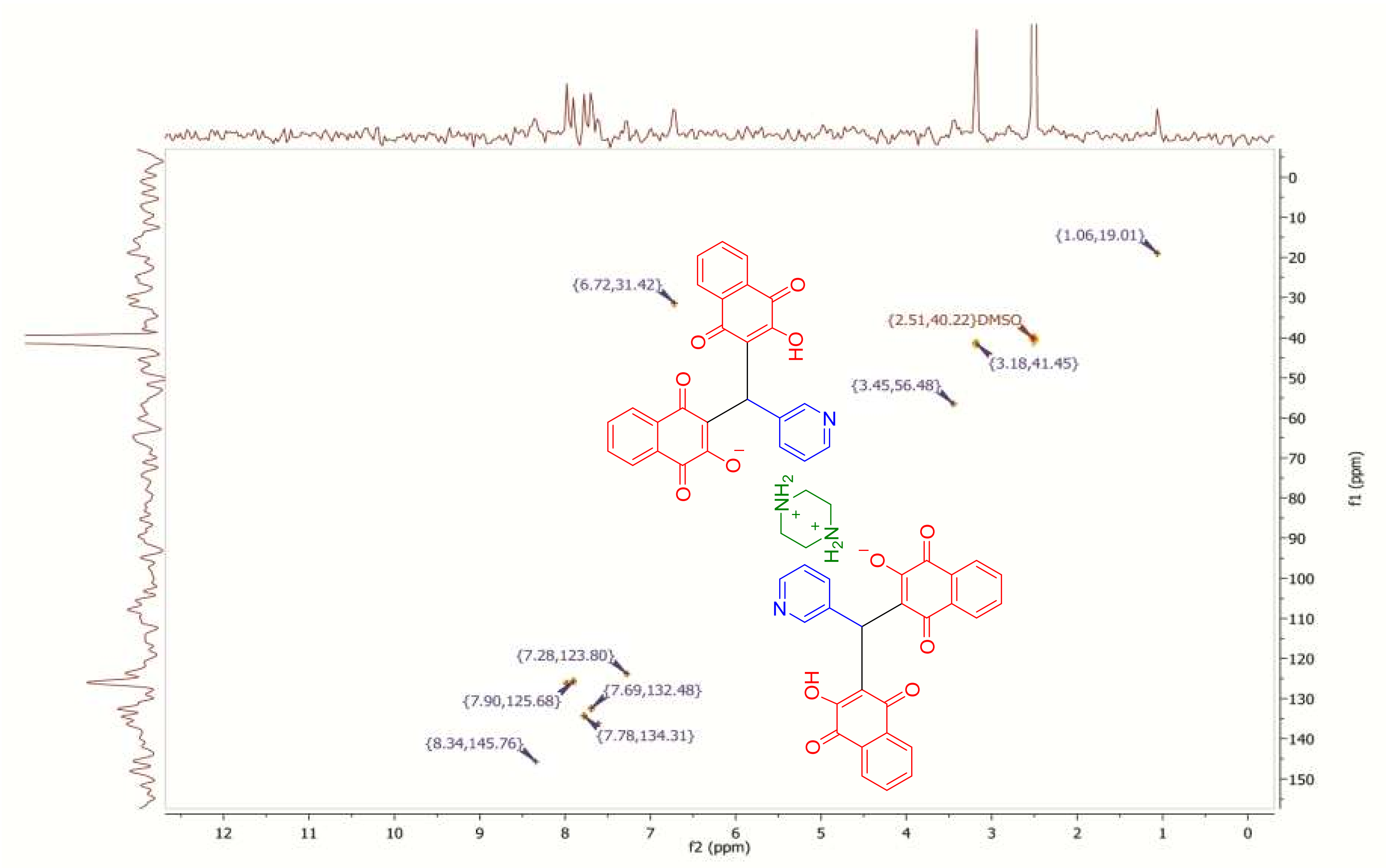

^{1}H^{13}C, HSQC-NMR spectrum of piperazine-1,4-diium 3-((1,4-dioxo-1,4-dihydronaphthalen-2-yl)(pyridin-3-yl)methyl)-1,4-dioxo-1,4-dihydronaphthalen-2-olate

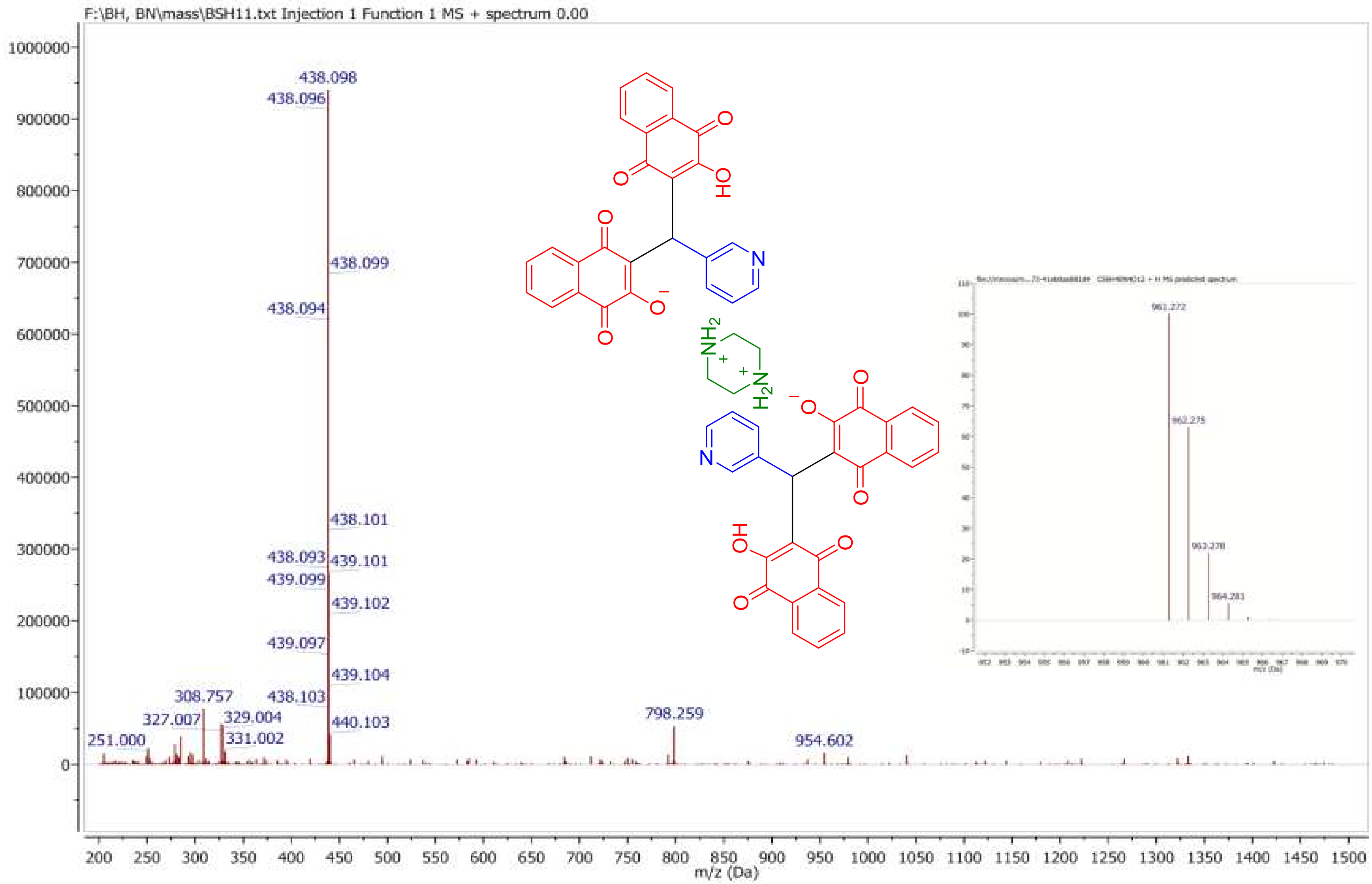

HR-Mass spectrum of piperazine-1,4-diium 3-((1,4-dioxo-1,4-dihydronaphthalen-2-yl)(pyridin-3-yl)methyl)-1,4-dioxo-1,4-dihydronaphthalen-2-olate

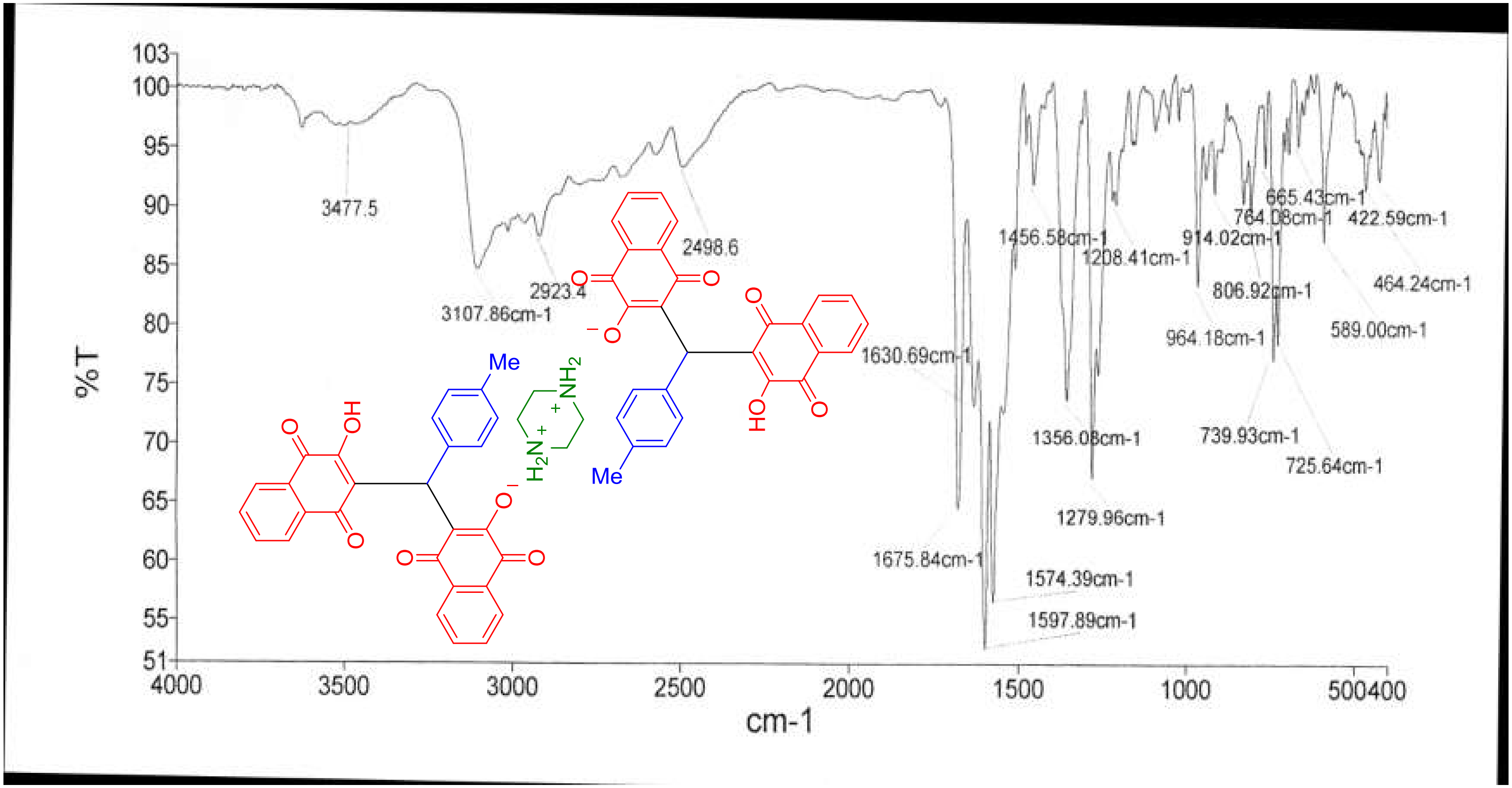

FT-IR spectrum of piperazine-1,4-diium 3-((3-hydroxy-1,4-dioxo-1,4-dihydronaphthalen-2-yl)(p-tolyl)methyl)-1,4-dioxo-1,4-dihydronaphthalen-2-olate

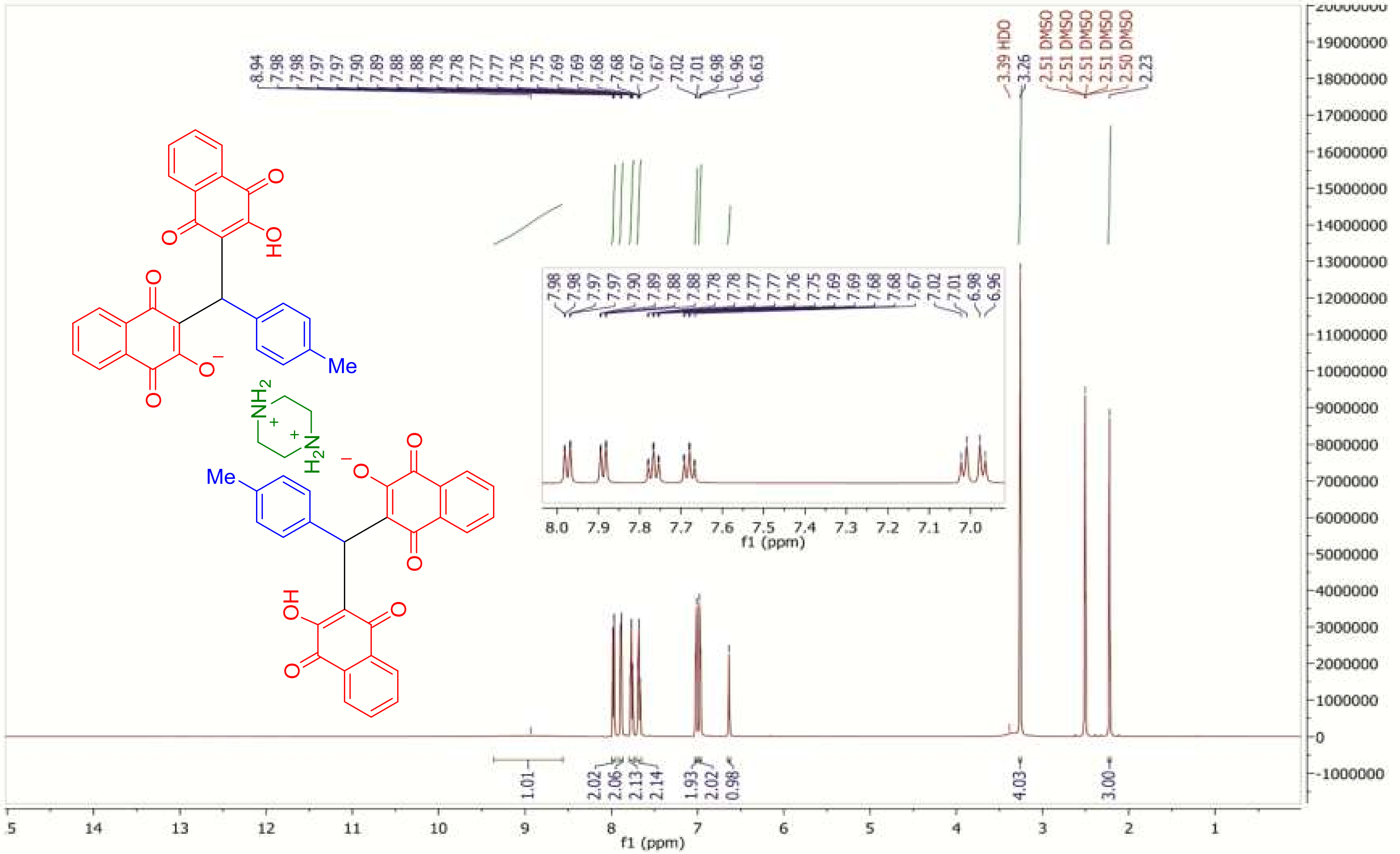

^{1}H NMR spectrum of piperazine-1,4-diium 3-((3-hydroxy-1,4-dioxo-1,4-dihydronaphthalen-2-yl)(p-tolyl)methyl)-1,4-dioxo-1,4-dihydronaphthalen-2-olate

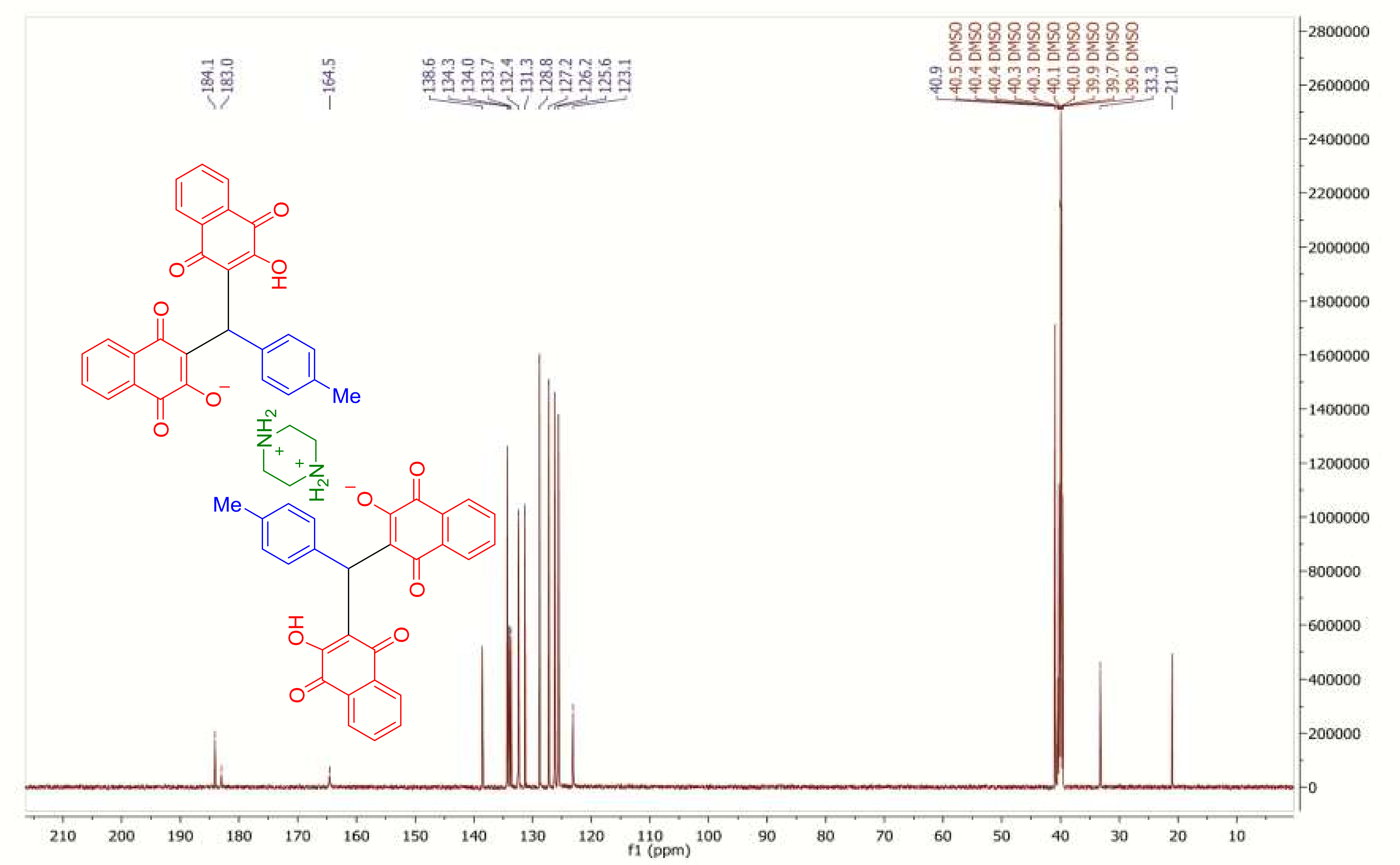

^{13}CNMR spectrum of piperazine-1,4-diium 3-((3-hydroxy-1,4-dioxo-1,4-dihydronaphthalen-2-yl)(p-tolyl)methyl)-1,4-dioxo-1,4-dihydronaphthalen-2-olate

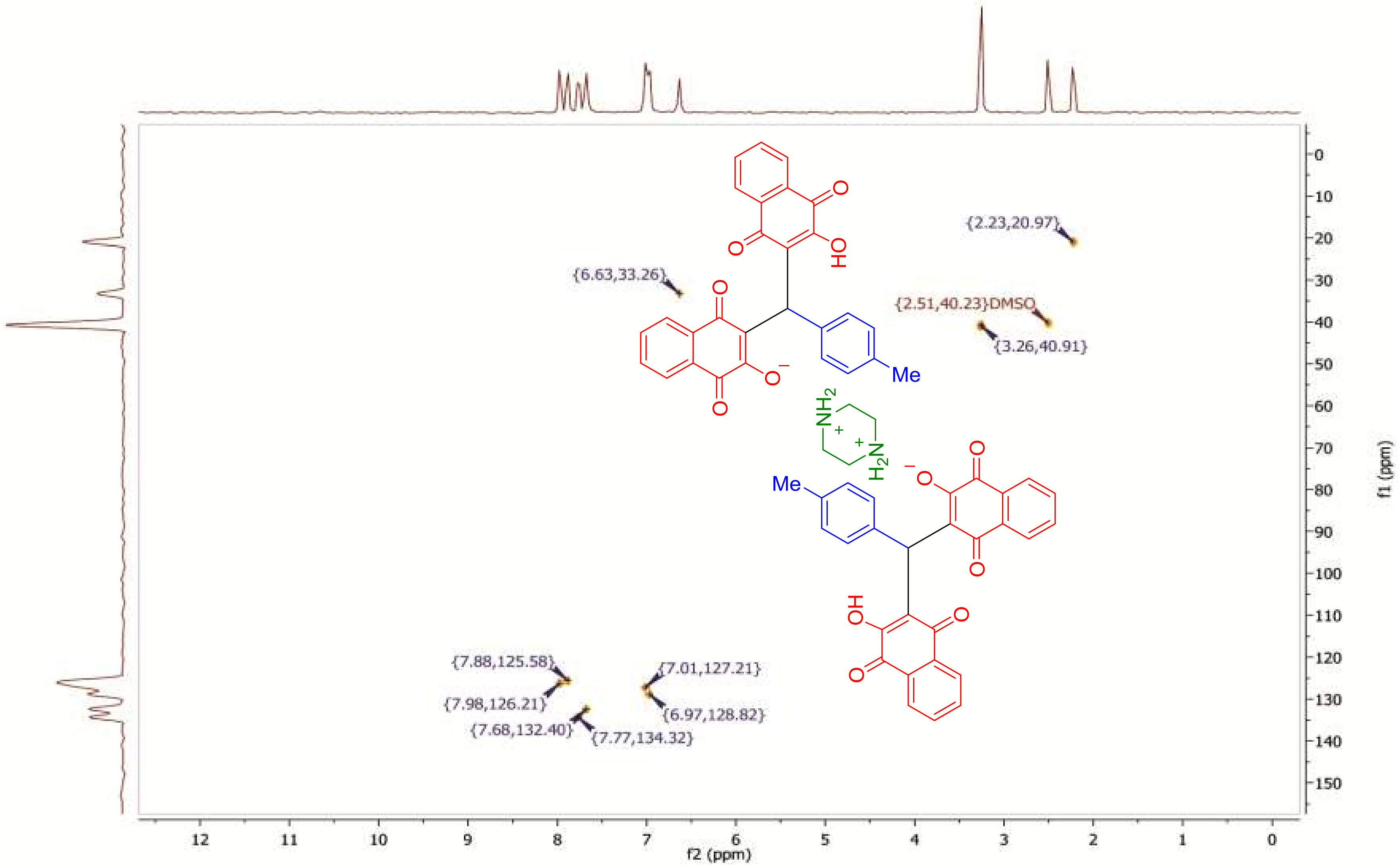

^{1}H^{13}C, HSQC-NMR piperazine-1,4-diium 3-((3-hydroxy-1,4-dioxo-1,4-dihydronaphthalen-2-yl)(p-tolyl)methyl)-1,4-dioxo-1,4-dihydronaphthalen-2-olate

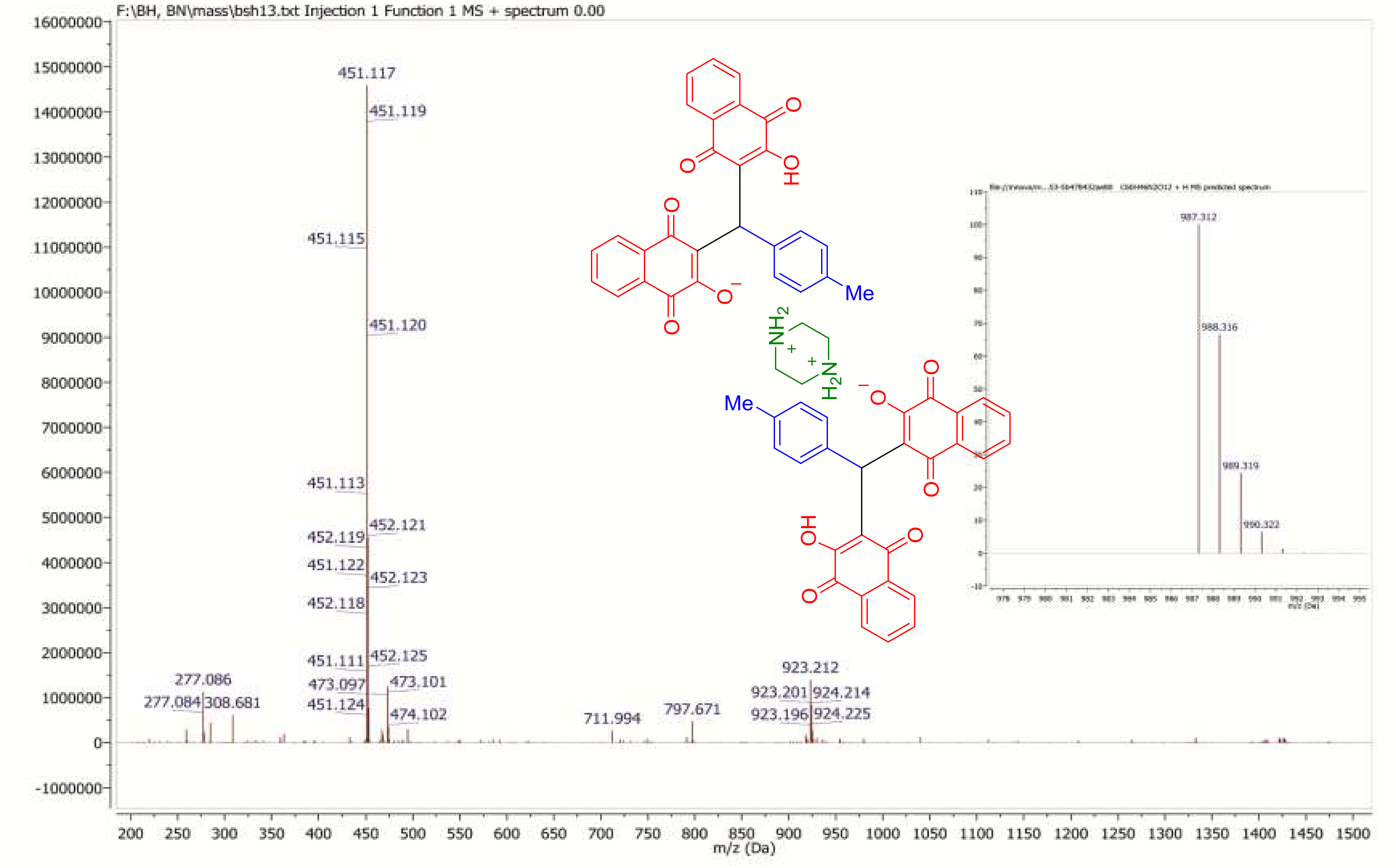

HR-Mass spectrum of piperazine-1,4-diium 3-((3-hydroxy-1,4-dioxo-1,4-dihydronaphthalen-2-yl)(p-tolyl)methyl)-1,4-dioxo-1,4-dihydronaphthalen-2-olate

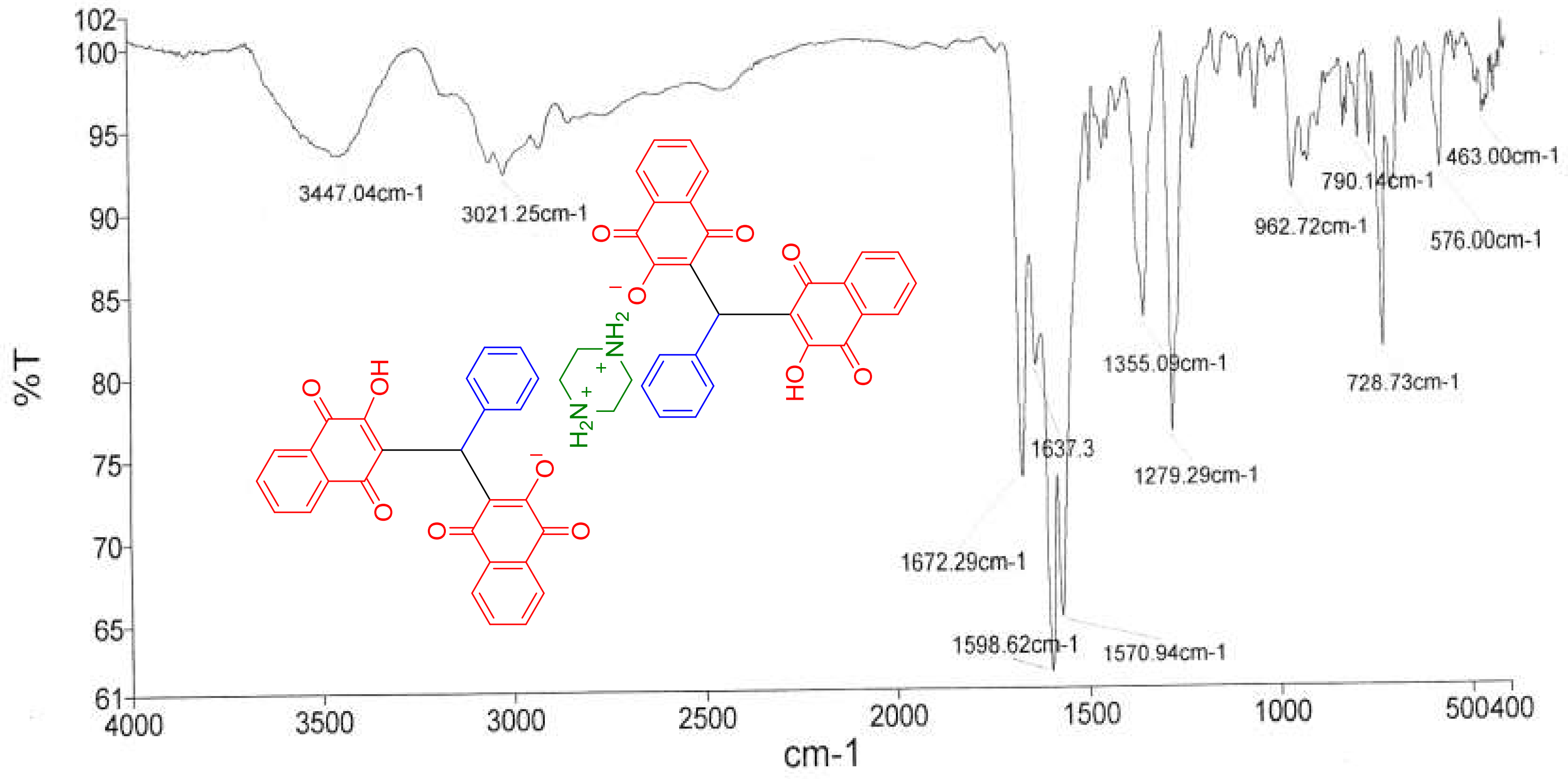

FT-IR spectrum of piperazine-1,4-diium 3-((3-hydroxy-1,4-dioxo-1,4-dihydronaphthalen-2-yl)(phenyl)methyl)-1,4-dioxo-1,4-dihydronaphthalen-2-olate

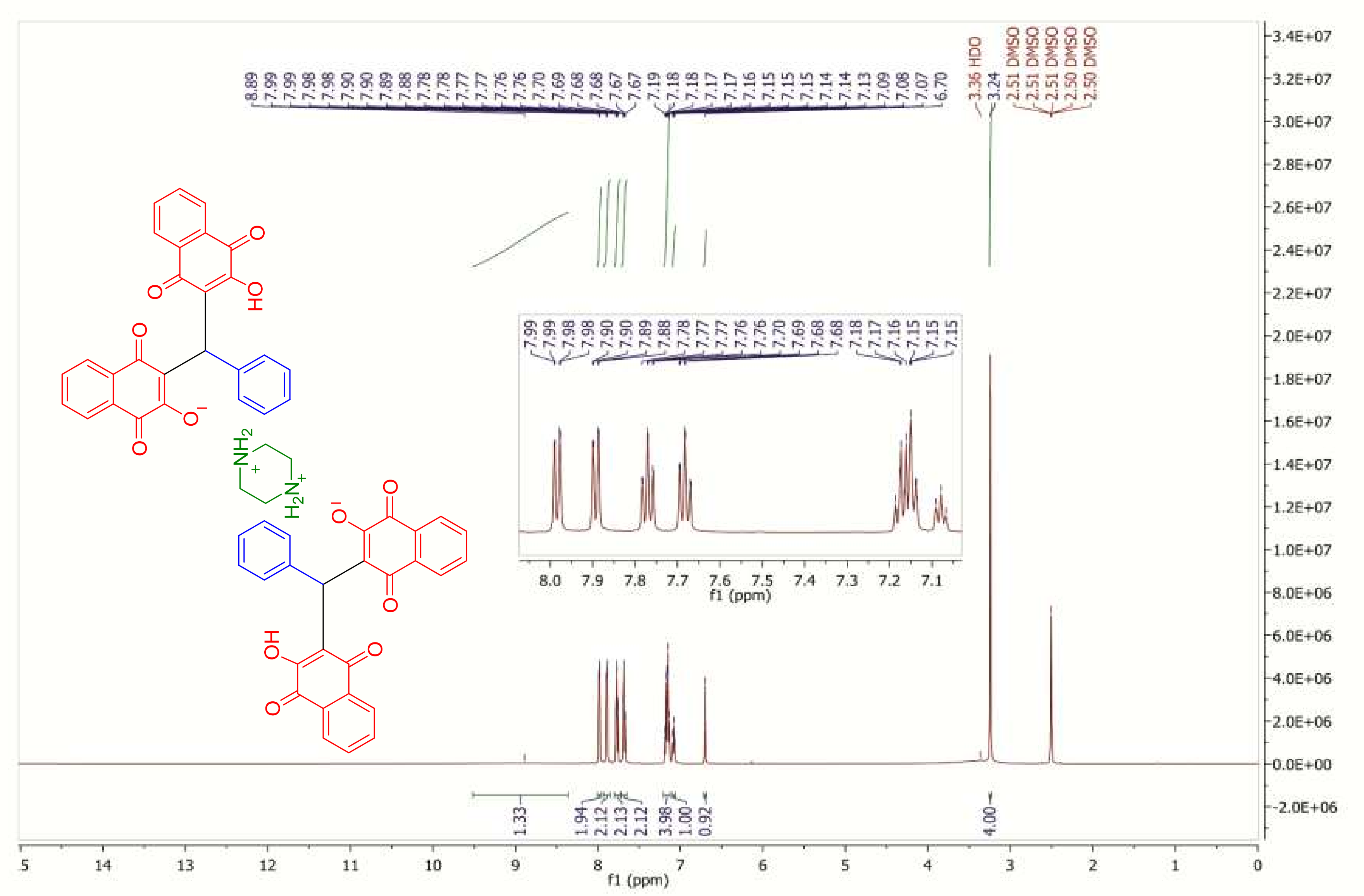

^{1}H NMR spectrum of piperazine-1,4-diium 3-((3-hydroxy-1,4-dioxo-1,4-dihydronaphthalen-2-yl)(phenyl)methyl)-1,4-dioxo-1,4-dihydronaphthalen-2-olate

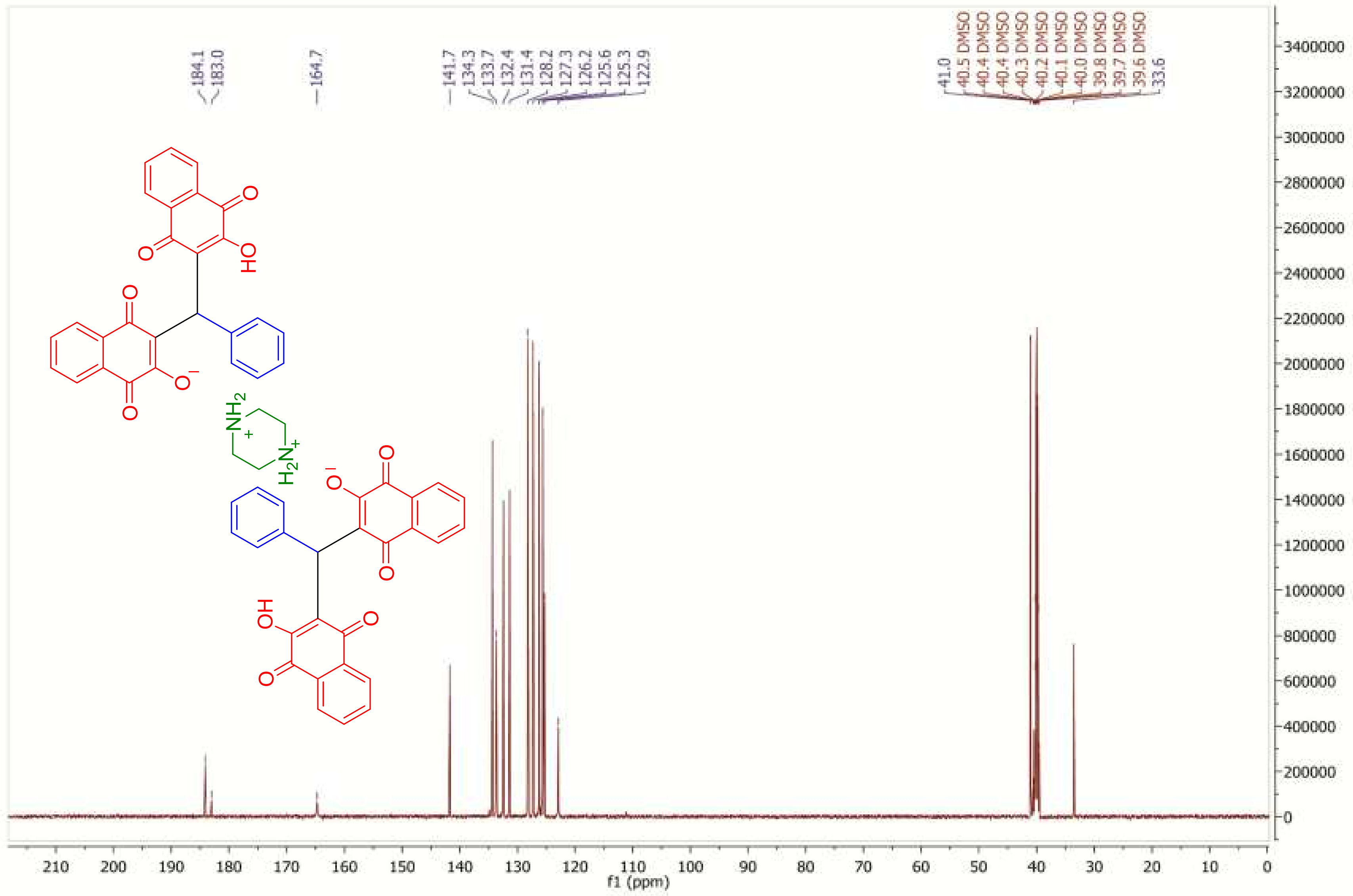

^{13}C NMR spectrum of piperazine-1,4-diium 3-((3-hydroxy-1,4-dioxo-1,4-dihydronaphthalen-2-yl)(phenyl)methyl)-1,4-dioxo-1,4-dihydronaphthalen-2-olate

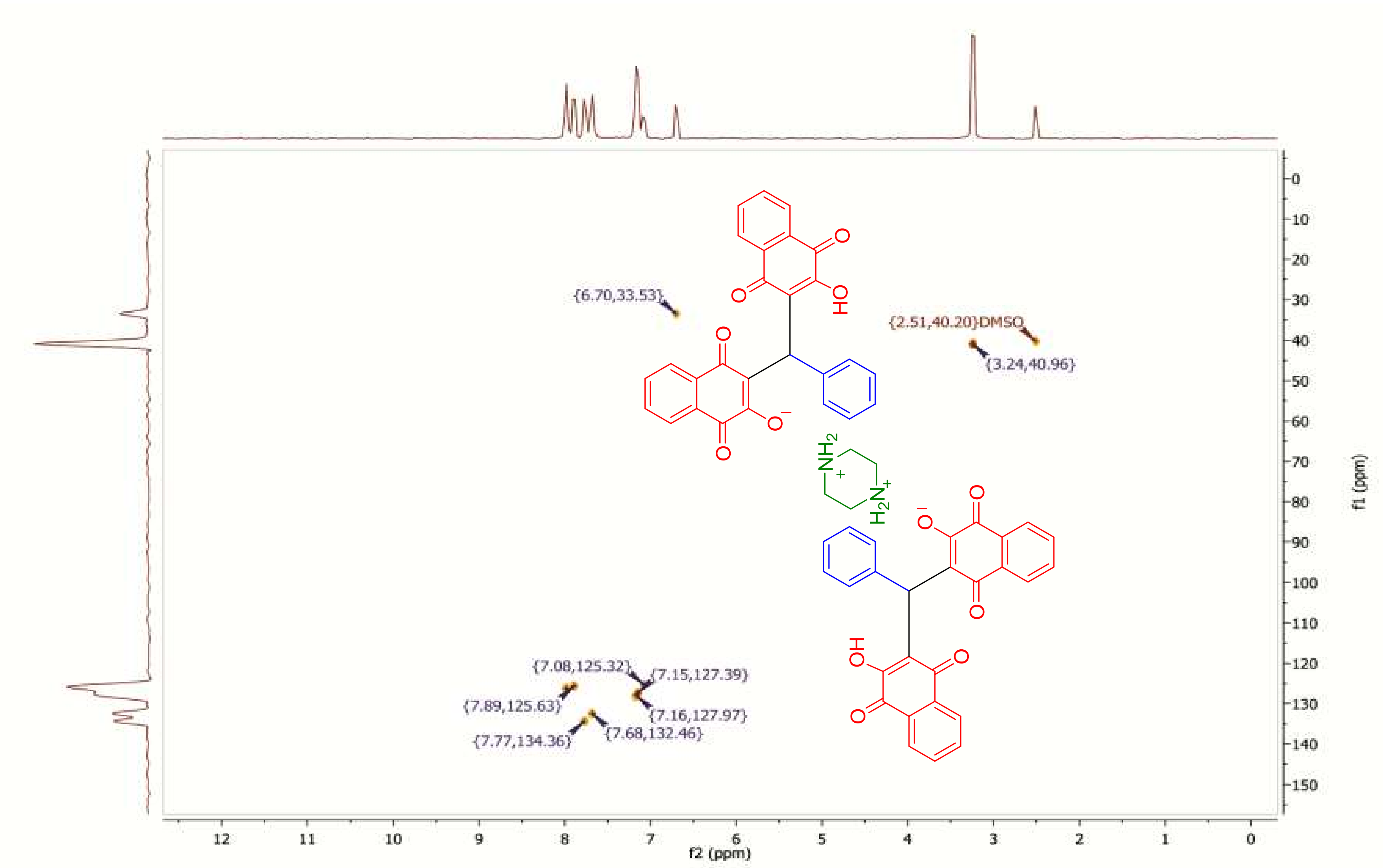

$^1H^{13}C$, HSQC-NMR spectrum of piperazine-1,4-diium 3-((3-hydroxy-1,4-dioxo-1,4-dihydronaphthalen-2-yl)(phenyl)methyl)-1,4-dioxo-1,4-dihydronaphthalen-2-olate

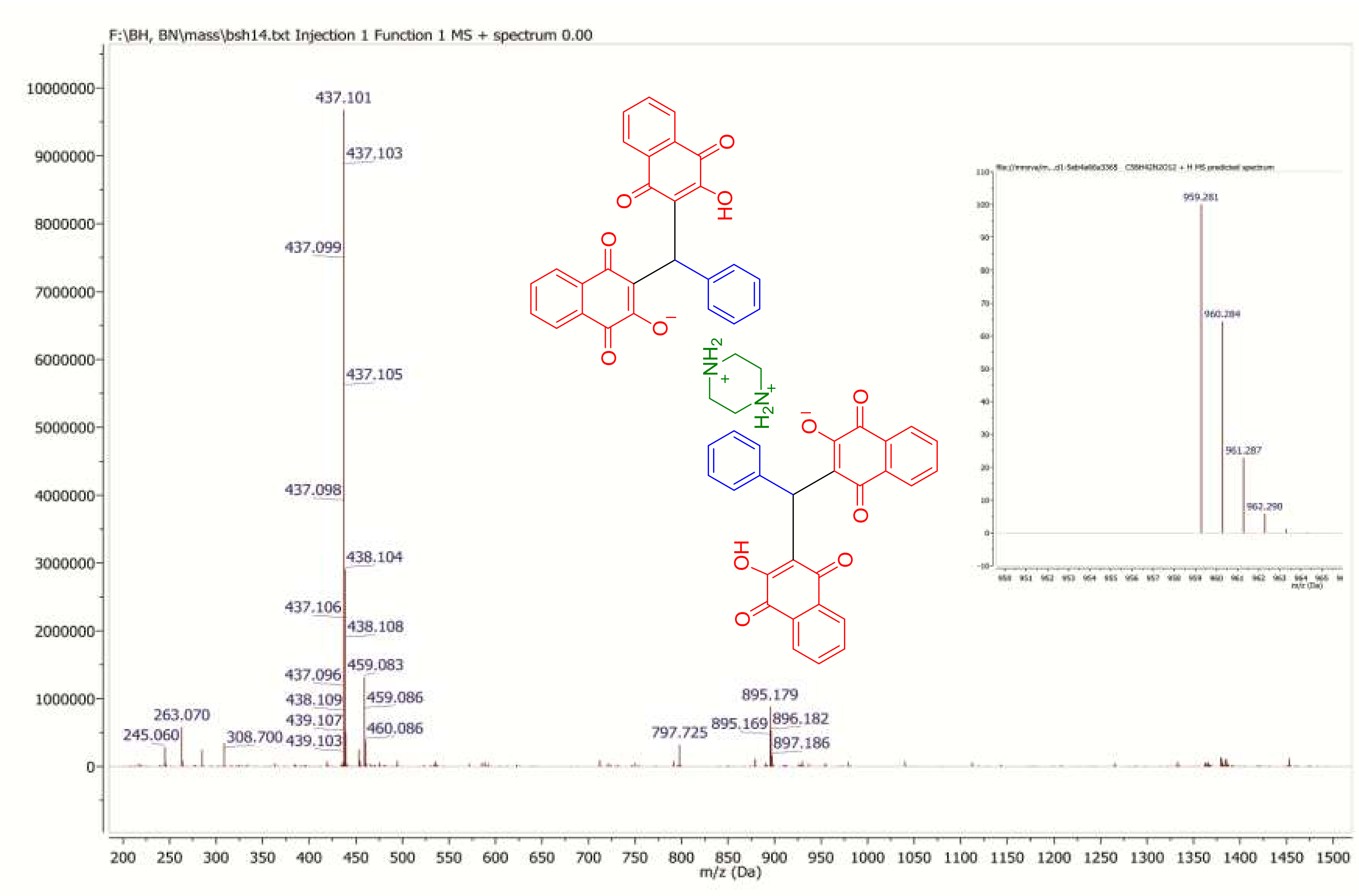

HR-Mass spectrum of piperazine-1,4-diium 3-((3-hydroxy-1,4-dioxo-1,4-dihydronaphthalen-2-yl)(phenyl)methyl)-1,4-dioxo-1,4-dihydronaphthalen-2-olate

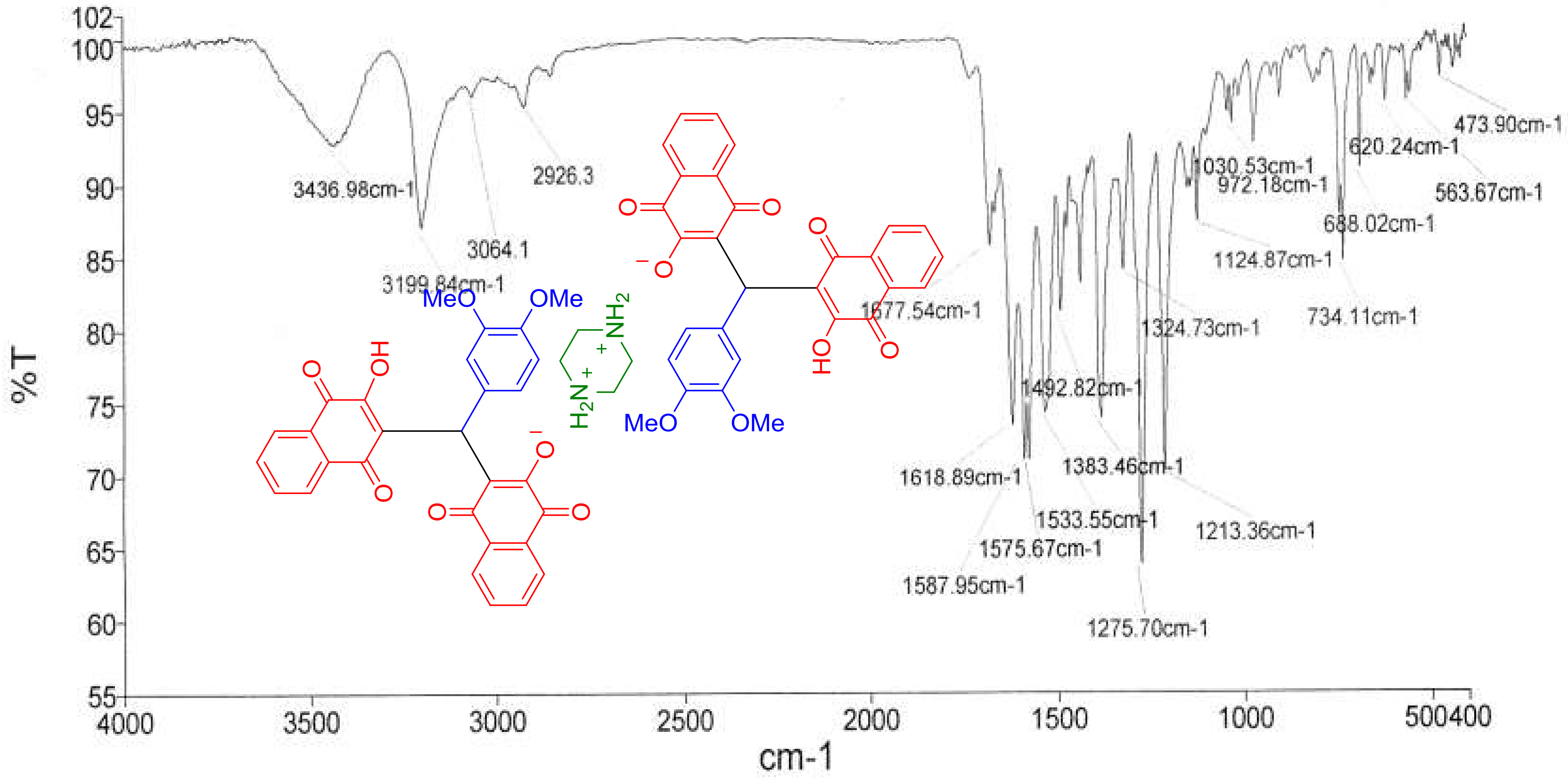

FT-IR spectrum of piperazine-1,4-diium 3-((3,4-dimethoxyphenyl)(3-hydroxy-1,4-dioxo-1,4-dihydronaphthalen-2-yl)methyl)-1,4-dioxo-1,4-dihydronaphthalen-2-olate

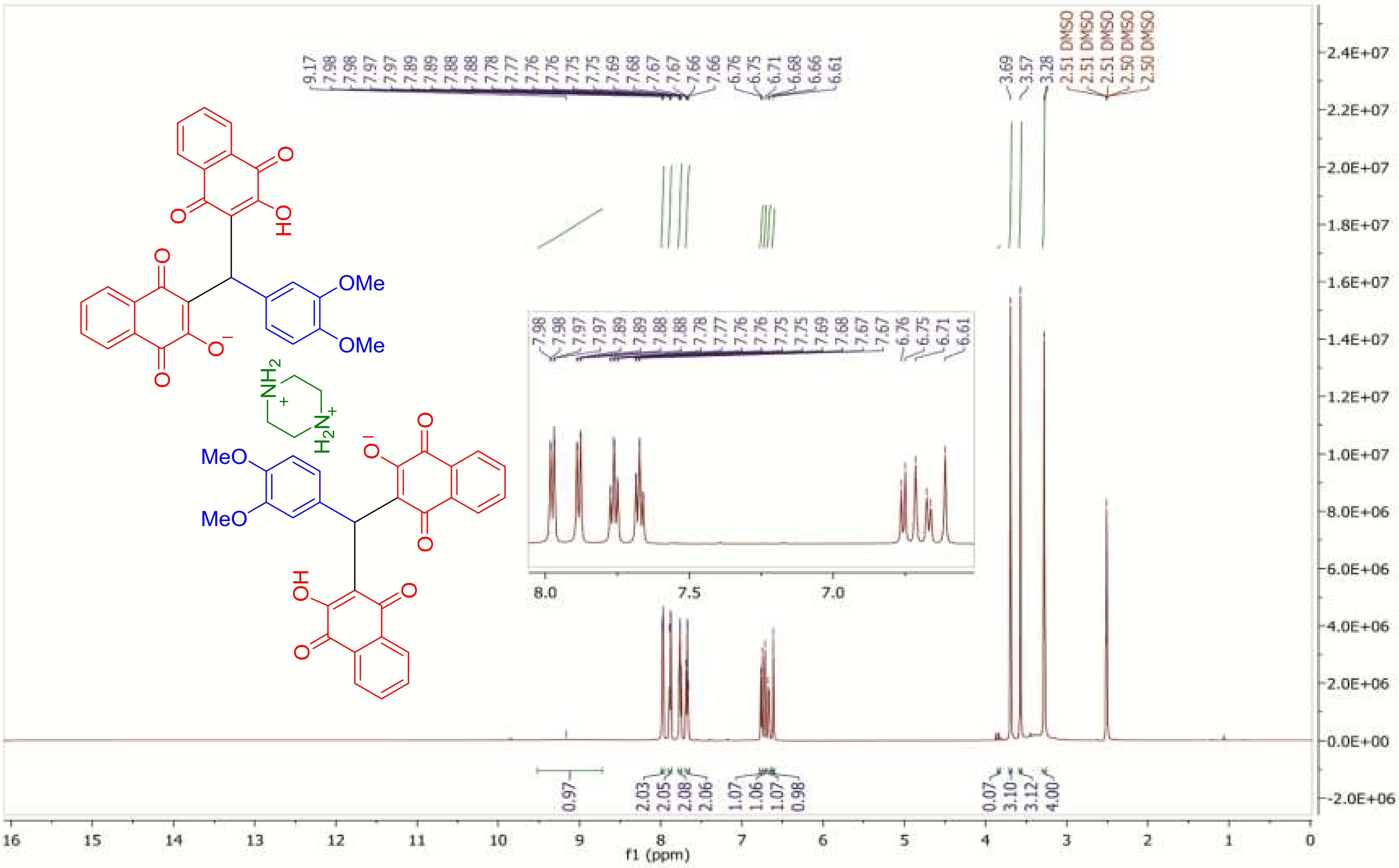

^{1}H NMR spectrum of piperazine-1,4-diium 3-((3,4-dimethoxyphenyl)(3-hydroxy-1,4-dioxo-1,4-dihydronaphthalen-2-yl)methyl)-1,4-dioxo-1,4-dihydronaphthalen-2-olate

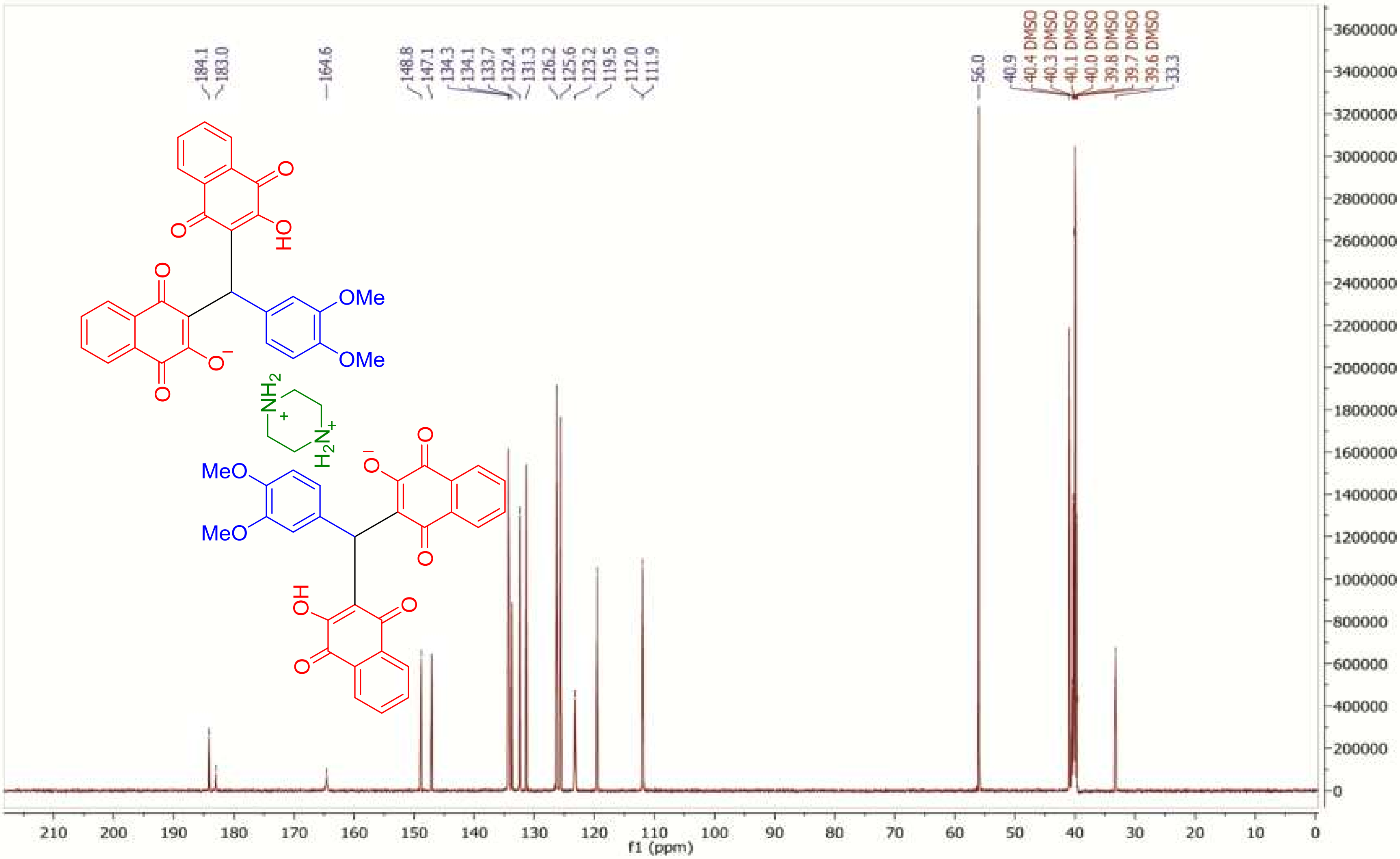

^{13}C NMR spectrum of piperazine-1,4-diium 3-((3,4-dimethoxyphenyl)(3-hydroxy-1,4-dioxo-1,4-dihydronaphthalen-2-yl)methyl)-1,4-dioxo-1,4-dihydronaphthalen-2-olate

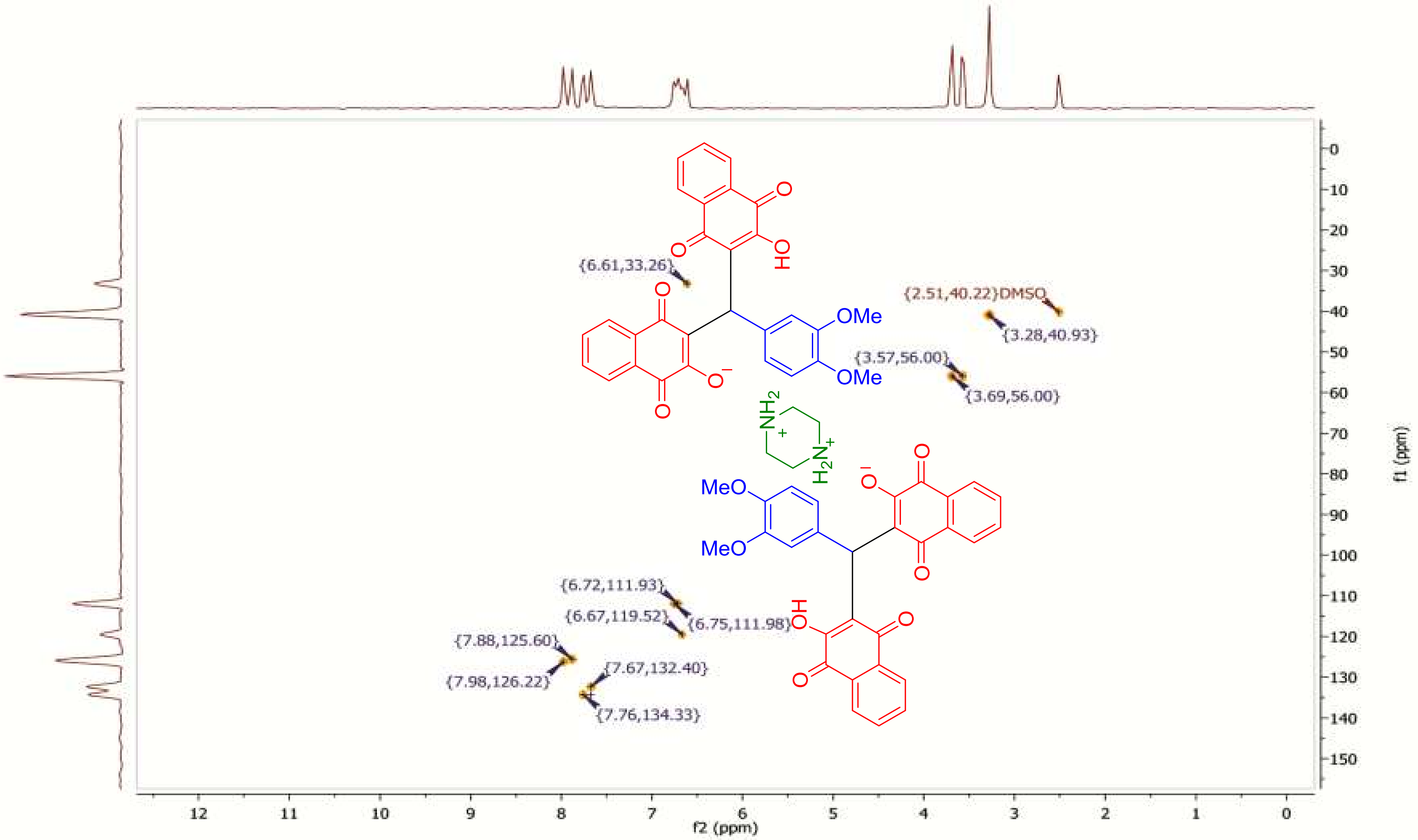

$^1H^{13}C$, HSQC-NMR spectrum of piperazine-1,4-diium 3-((3,4-dimethoxyphenyl)(3-hydroxy-1,4-dioxo-1,4-dihydronaphthalen-2-yl)methyl)-1,4-dioxo-1,4-dihydronaphthalen-2-olate

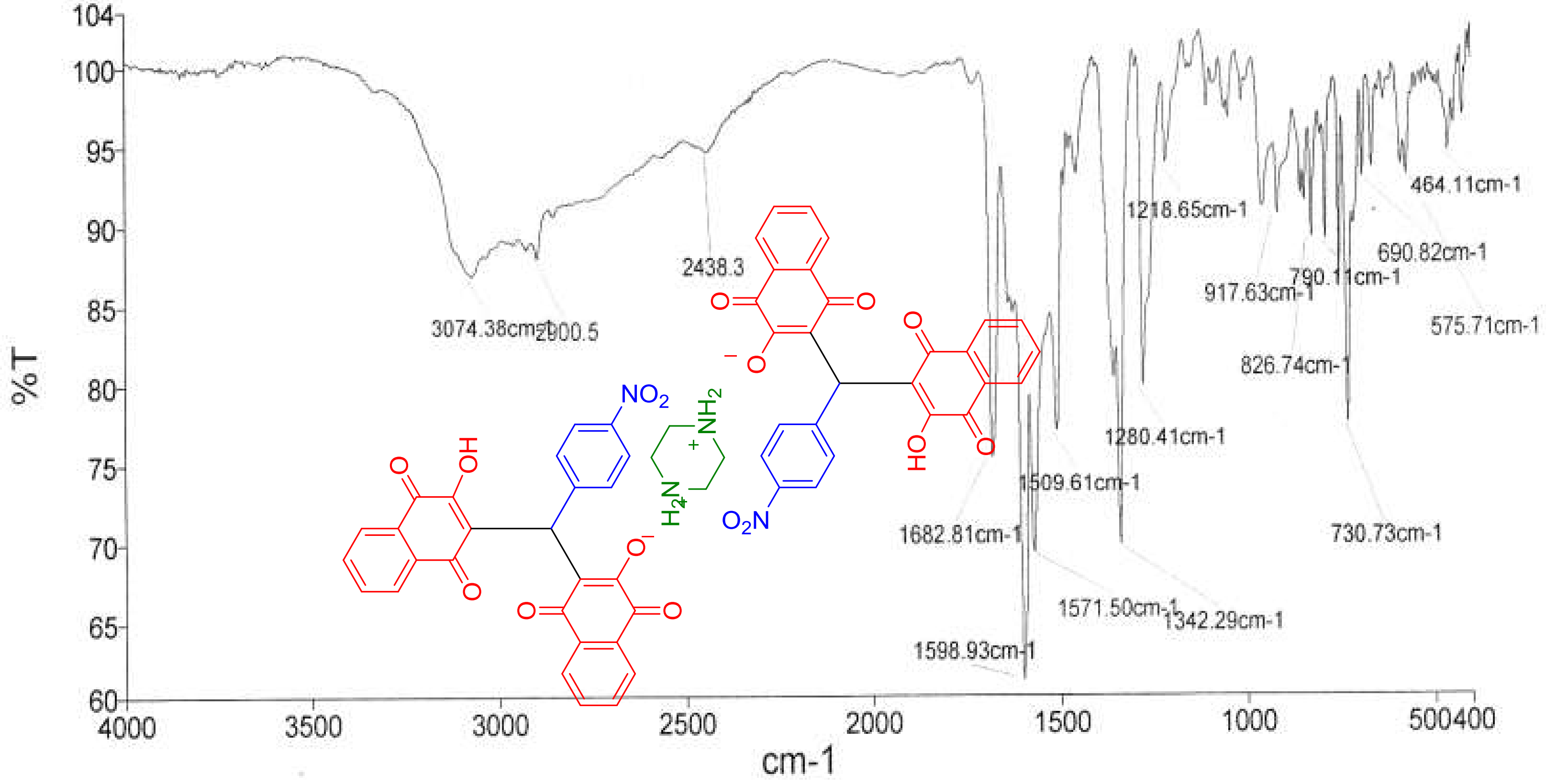

FT-IR spectrum of piperazine-1,4-diium 3-((3-hydroxy-1,4-dioxo-1,4-dihydronaphthalen-2-yl)(4-nitrophenyl)methyl)-1,4-dioxo-1,4-dihydronaphthalen-2-olate

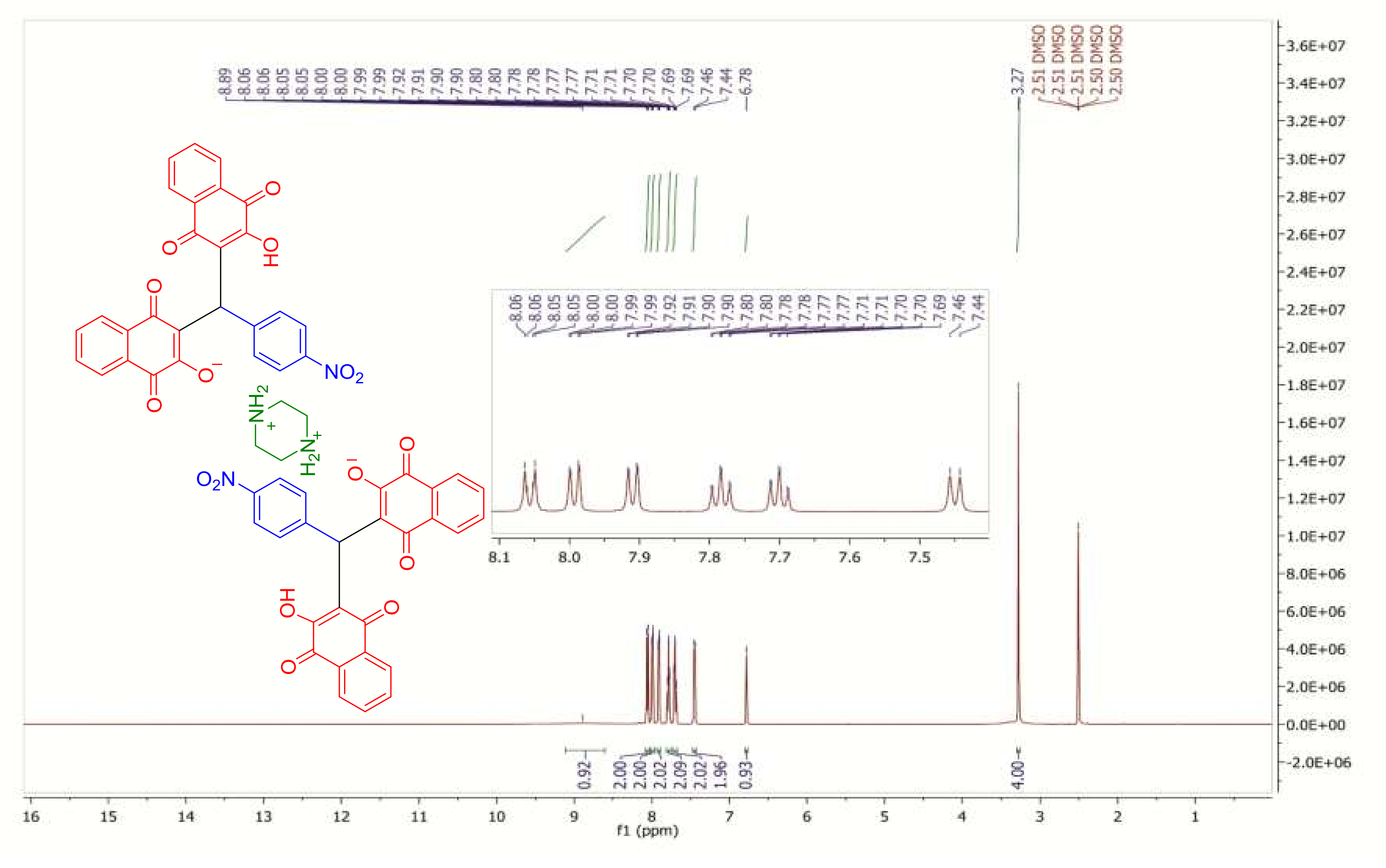

^{1}H NMR spectrum of piperazine-1,4-diium 3-((3-hydroxy-1,4-dioxo-1,4-dihydronaphthalen-2-yl)(4-nitrophenyl)methyl)-1,4-dioxo-1,4-dihydronaphthalen-2-olate

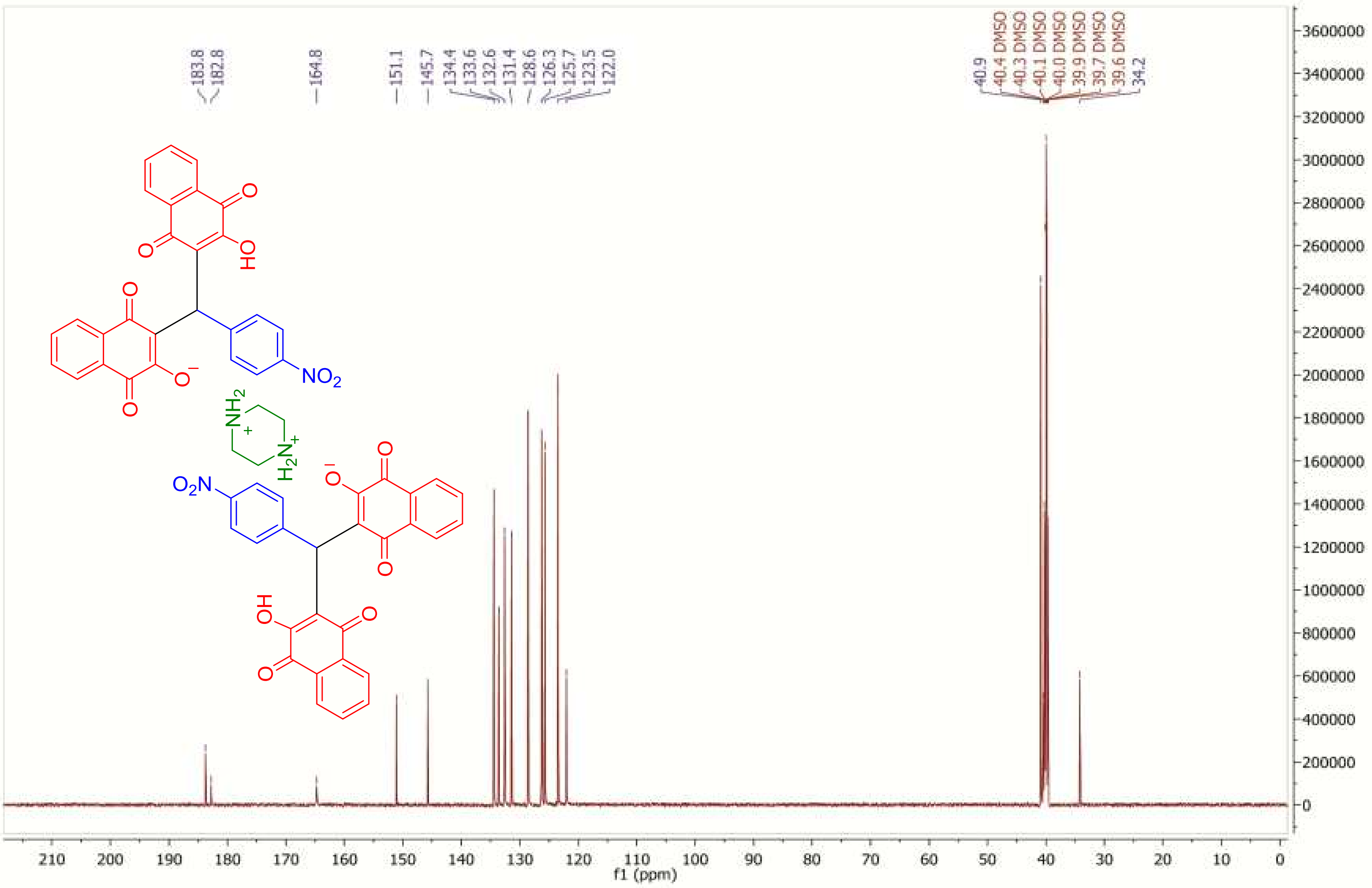

^{13}C NMR spectrum of piperazine-1,4-diium 3-((3-hydroxy-1,4-dioxo-1,4-dihydronaphthalen-2-yl)(4-nitrophenyl)methyl)-1,4-dioxo-1,4-dihydronaphthalen-2-olate

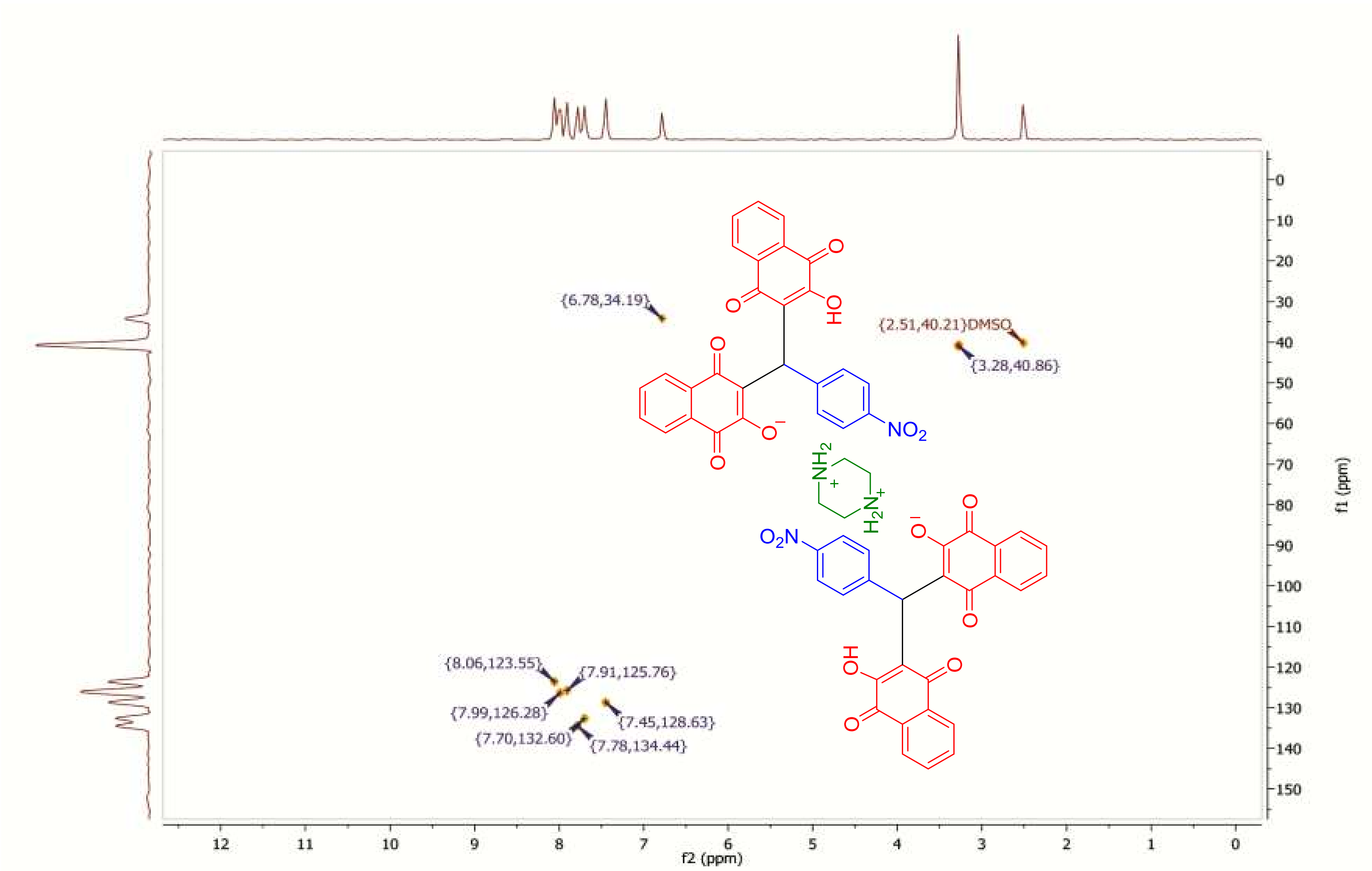

$^{1}H^{13}C$, HSQC-NMR spectrum of piperazine-1,4-diium 3-((3-hydroxy-1,4-dioxo-1,4-dihydronaphthalen-2-yl)(4-nitrophenyl)methyl)-1,4-dioxo-1,4-dihydronaphthalen-2-olate

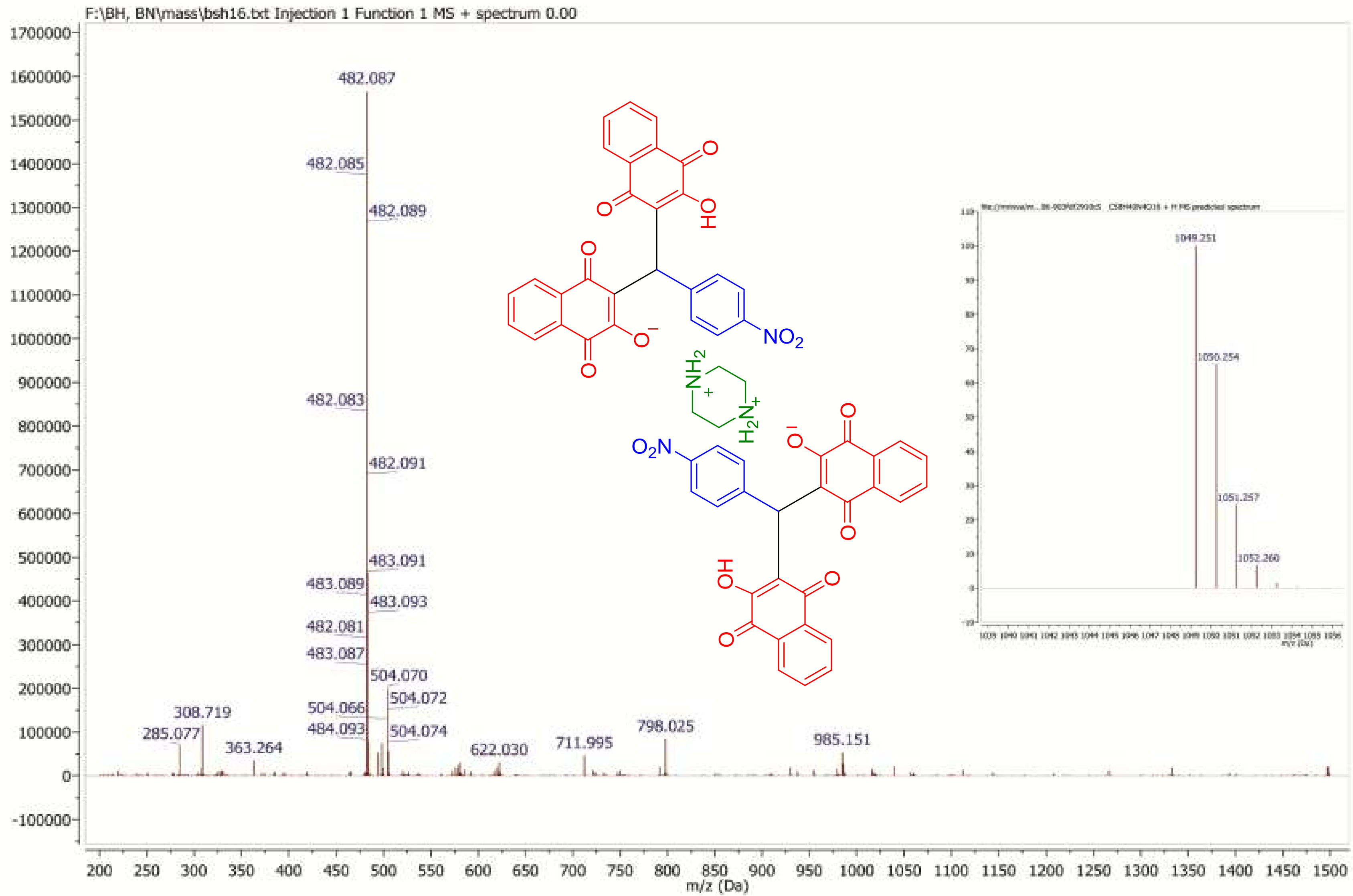

HR-Mass spectrum of piperazine-1,4-diium 3-((3-hydroxy-1,4-dioxo-1,4-dihydronaphthalen-2-yl)(4-nitrophenyl)methyl)-1,4-dioxo-1,4-dihydronaphthalen-2-olate

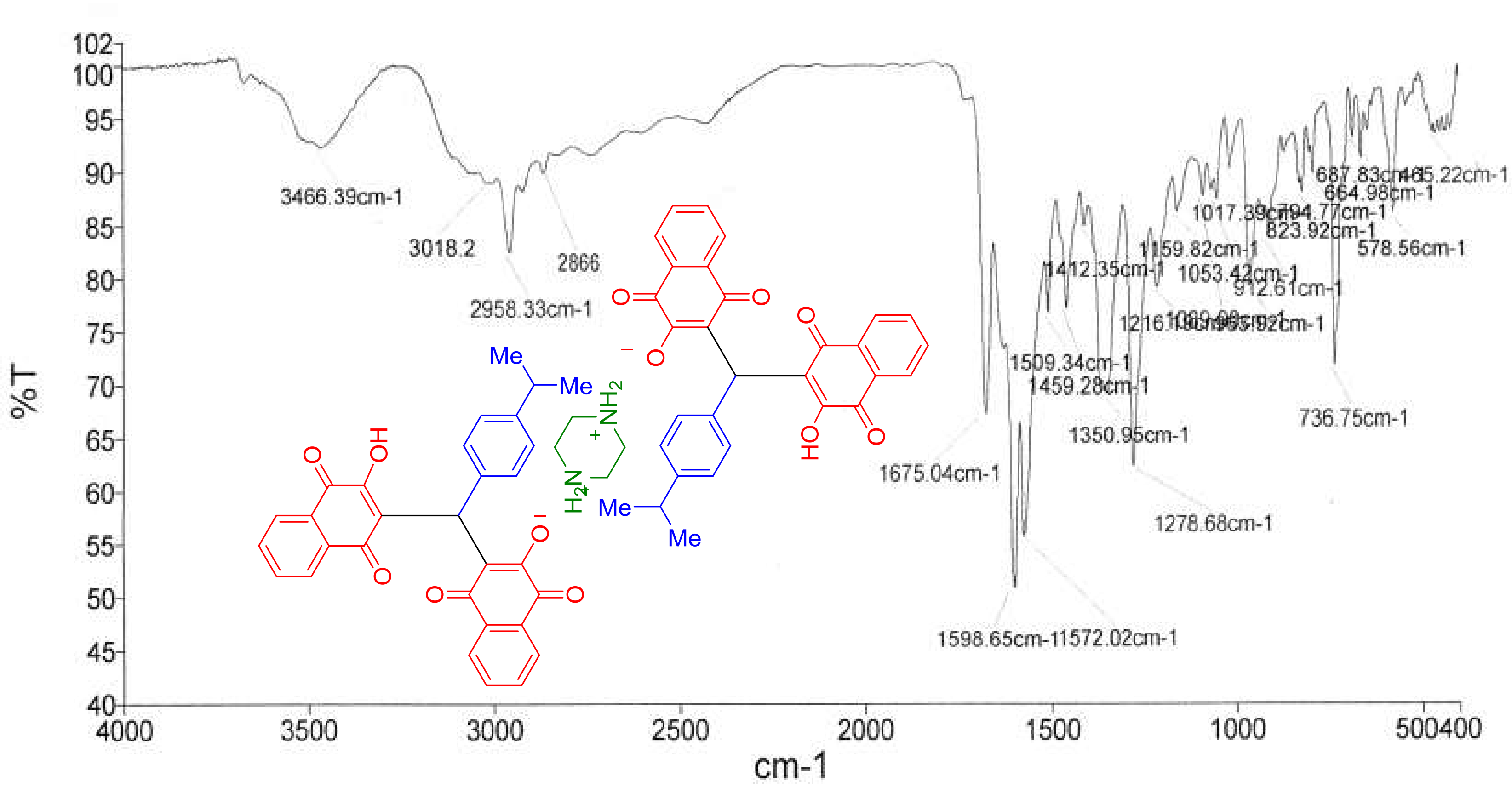

FT-IR spectrum of piperazine-1,4-diium 3-((3-hydroxy-1,4-dioxo-1,4-dihydronaphthalen-2-yl)(4-isopropylphenyl)methyl)-1,4-dioxo-1,4-dihydronaphthalen-2-olate

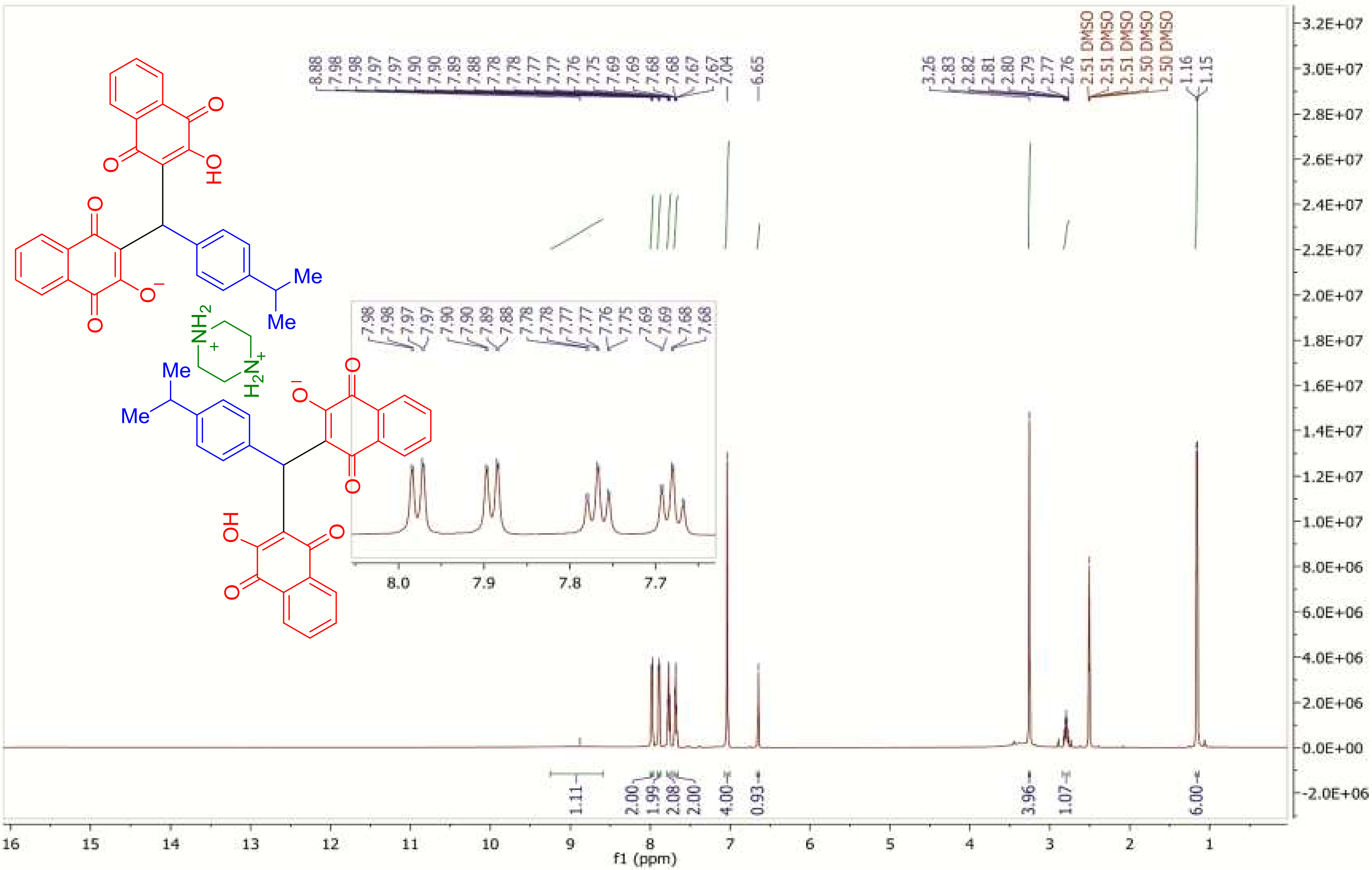

^{1}H NMR spectrum of piperazine-1,4-diium 3-((3-hydroxy-1,4-dioxo-1,4-dihydronaphthalen-2-yl)(4-isopropylphenyl)methyl)-1,4-dioxo-1,4-dihydronaphthalen-2-olate

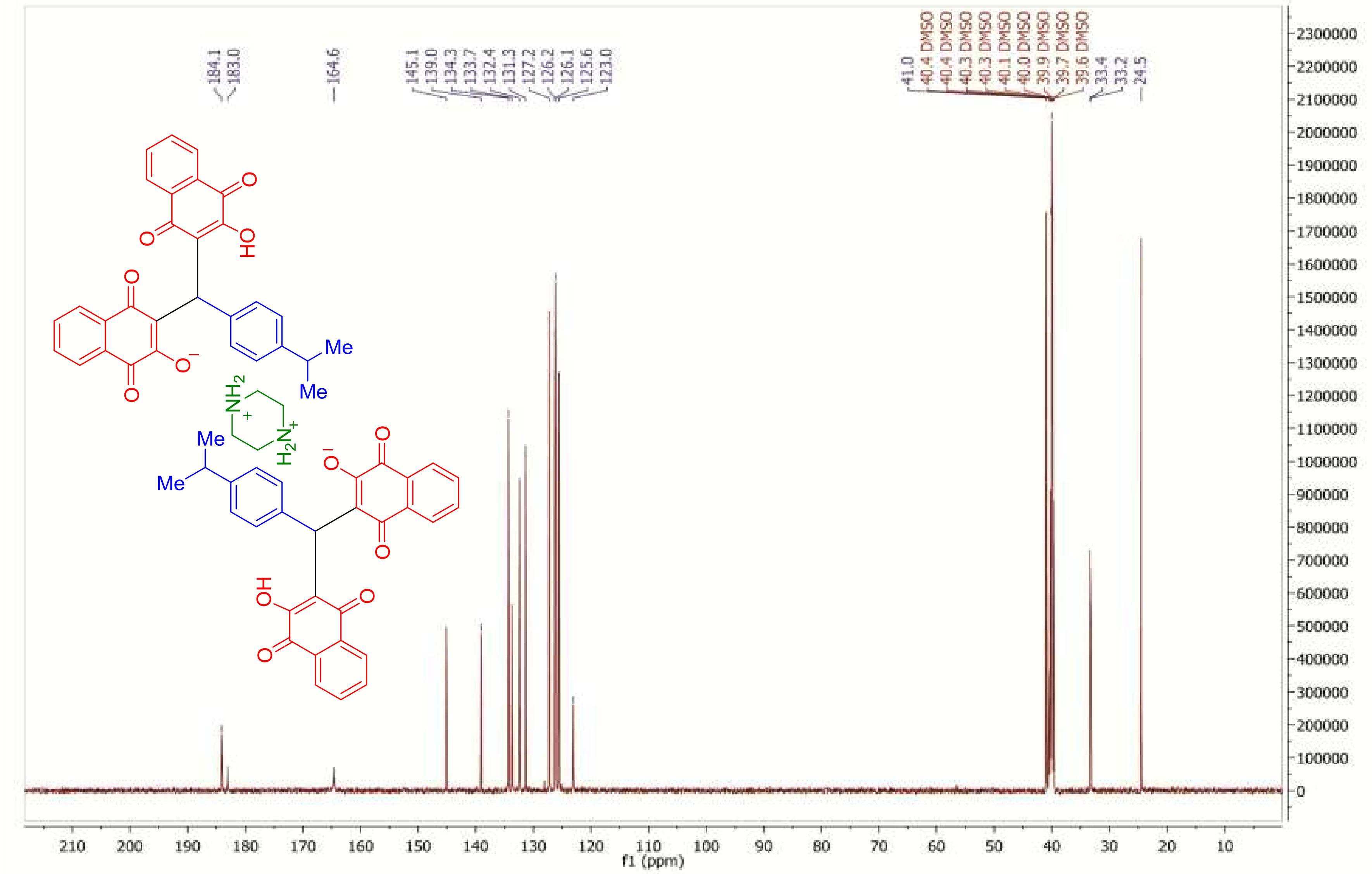

^{13}C NMR spectrum of piperazine-1,4-diium 3-((3-hydroxy-1,4-dioxo-1,4-dihydronaphthalen-2-yl)(4-isopropylphenyl)methyl)-1,4-dioxo-1,4-dihydronaphthalen-2-olate

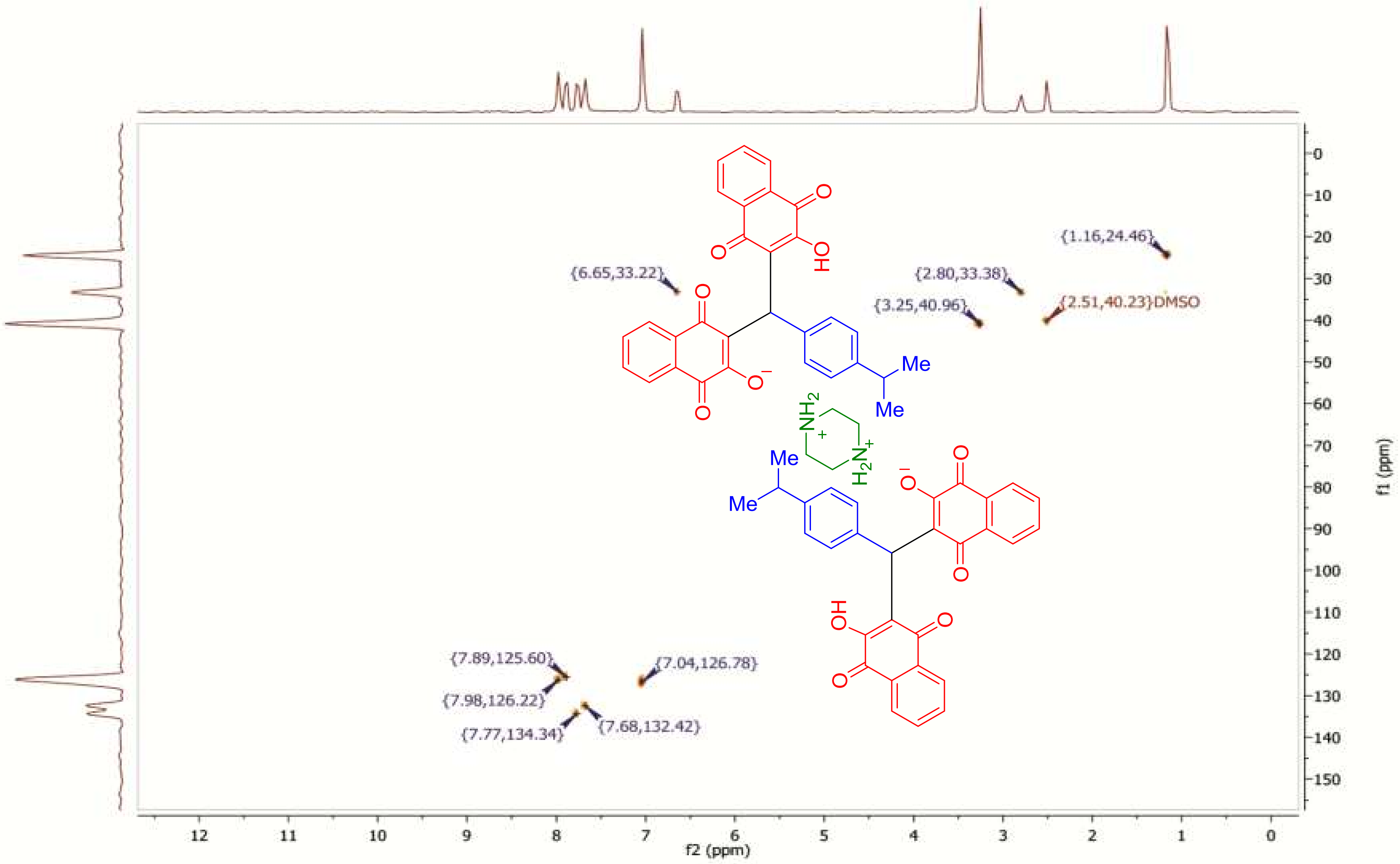

$^{1}H^{13}C$, HSQC-NMR spectrum of piperazine-1,4-diium 3-((3-hydroxy-1,4-dioxo-1,4-dihydronaphthalen-2-yl)(4-isopropylphenyl)methyl)-1,4-dioxo-1,4-dihydronaphthalen-2-olate

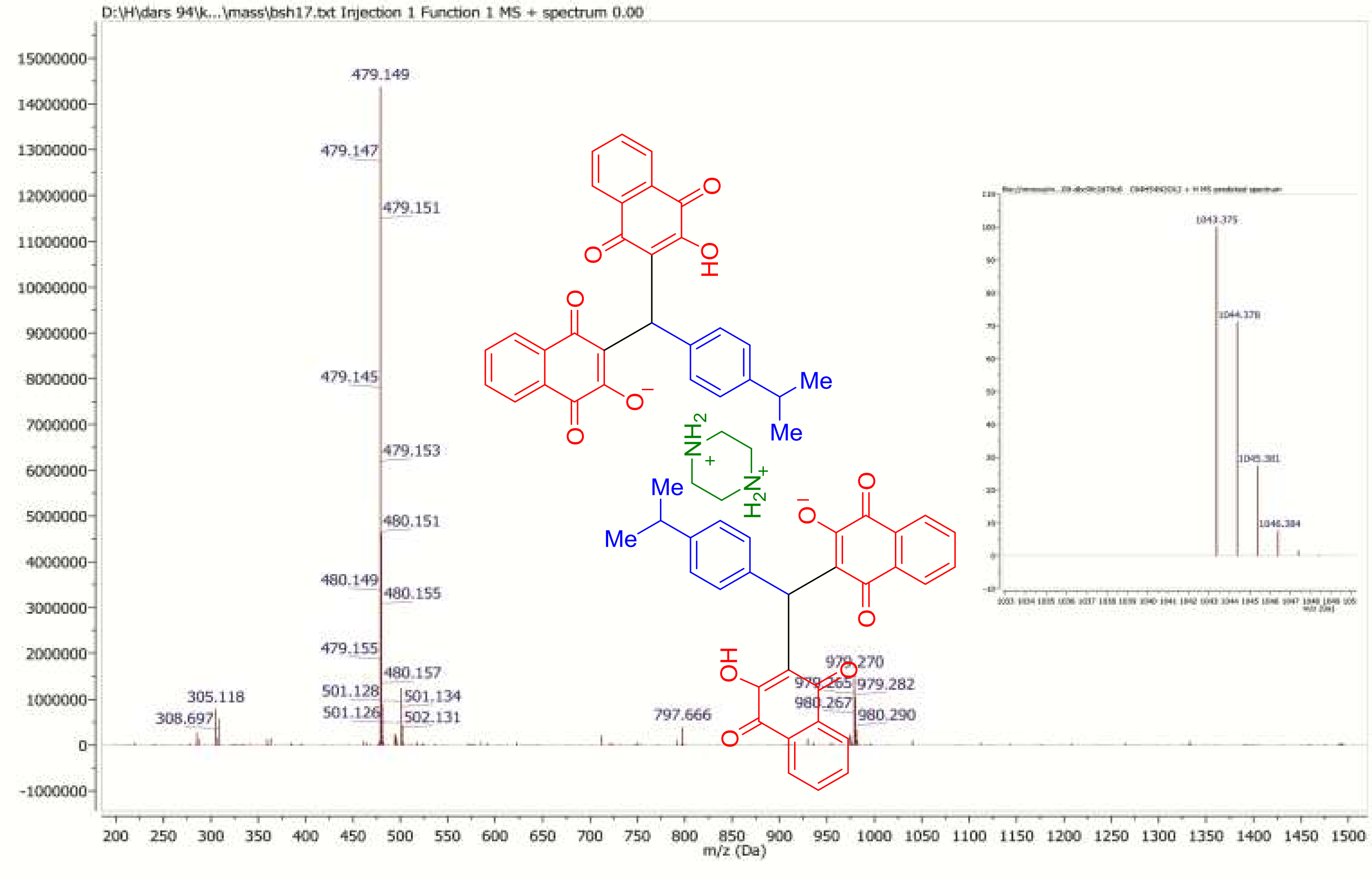

HR-Mass spectrum of piperazine-1,4-diium 3-((3-hydroxy-1,4-dioxo-1,4-dihydronaphthalen-2-yl)(4-isopropylphenyl)methyl)-1,4-dioxo-1,4-dihydronaphthalen-2-olate
